# The *Paramecium* Germline Genome Provides a Niche for Intragenic Parasitic DNA: Evolutionary Dynamics of Internal Eliminated Sequences

**DOI:** 10.1371/journal.pgen.1002984

**Published:** 2012-10-04

**Authors:** Olivier Arnaiz, Nathalie Mathy, Céline Baudry, Sophie Malinsky, Jean-Marc Aury, Cyril Denby Wilkes, Olivier Garnier, Karine Labadie, Benjamin E. Lauderdale, Anne Le Mouël, Antoine Marmignon, Mariusz Nowacki, Julie Poulain, Malgorzata Prajer, Patrick Wincker, Eric Meyer, Sandra Duharcourt, Laurent Duret, Mireille Bétermier, Linda Sperling

**Affiliations:** 1 CNRS UPR3404 Centre de Génétique Moléculaire, Gif-sur-Yvette, France; 2 Département de Biologie, Université Paris-Sud, Orsay, France; 3 CNRS FRC3115, Centre de Recherches de Gif–sur-Yvette, Gif-sur-Yvette, France; 4 Ecole Normale Supérieure, Institut de Biologie de l'ENS, IBENS, Paris, France; 5 INSERM, U1024, Paris, France; 6 CNRS, UMR 8197, Paris, France; 7 Commissariat à l'Energie Atomique (CEA), Institut de Génomique (IG), Genoscope, Evry, France; 8 Methodology Institute, London School of Economics, London, United Kingdom; 9 Institute of Cell Biology, University of Bern, Bern, Switzerland; 10 Department of Experimental Zoology, Institute of Systematics and Evolution of Animals, Polish Academy of Sciences, Krakow, Poland; 11 Centre National de Recherche Scientifique (CNRS), UMR 8030, CP5706, Evry, France; 12 Université d'Evry, Evry, France; 13 Institut Jacques Monod, CNRS, UMR 7592, Université Paris Diderot, Sorbonne Paris Cité, Paris, France; 14 Université de Lyon, Université Lyon 1, CNRS, UMR 5558, Laboratoire de Biométrie et Biologie Evolutive, Villeurbanne, France; Fred Hutchinson Cancer Research Center, United States of America

## Abstract

Insertions of parasitic DNA within coding sequences are usually deleterious and are generally counter-selected during evolution. Thanks to nuclear dimorphism, ciliates provide unique models to study the fate of such insertions. Their germline genome undergoes extensive rearrangements during development of a new somatic macronucleus from the germline micronucleus following sexual events. In *Paramecium*, these rearrangements include precise excision of unique-copy Internal Eliminated Sequences (IES) from the somatic DNA, requiring the activity of a domesticated *piggyBac* transposase, PiggyMac. We have sequenced *Paramecium tetraurelia* germline DNA, establishing a genome-wide catalogue of ∼45,000 IESs, in order to gain insight into their evolutionary origin and excision mechanism. We obtained direct evidence that PiggyMac is required for excision of all IESs. Homology with known *P. tetraurelia* Tc1/mariner transposons, described here, indicates that at least a fraction of IESs derive from these elements. Most IES insertions occurred before a recent whole-genome duplication that preceded diversification of the *P. aurelia* species complex, but IES invasion of the *Paramecium* genome appears to be an ongoing process. Once inserted, IESs decay rapidly by accumulation of deletions and point substitutions. Over 90% of the IESs are shorter than 150 bp and present a remarkable size distribution with a ∼10 bp periodicity, corresponding to the helical repeat of double-stranded DNA and suggesting DNA loop formation during assembly of a transpososome-like excision complex. IESs are equally frequent within and between coding sequences; however, excision is not 100% efficient and there is selective pressure against IES insertions, in particular within highly expressed genes. We discuss the possibility that ancient domestication of a *piggyBac* transposase favored subsequent propagation of transposons throughout the germline by allowing insertions in coding sequences, a fraction of the genome in which parasitic DNA is not usually tolerated.

## Author Summary

Ciliates are unicellular eukaryotes that rearrange their genomes at every sexual generation when a new somatic macronucleus, responsible for gene expression, develops from a copy of the germline micronucleus. In *Paramecium*, assembly of a functional somatic genome requires precise excision of interstitial DNA segments, the Internal Eliminated Sequences (IES), involving a domesticated *piggyBac* transposase, PiggyMac. To study IES origin and evolution, we sequenced germline DNA and identified 45,000 IESs. We found that at least some of these unique-copy elements are decayed Tc1/mariner transposons and that IES insertion is likely an ongoing process. After insertion, elements decay rapidly by accumulation of deletions and substitutions. The 93% of IESs shorter than 150 bp display a remarkable size distribution with a periodicity of 10 bp, the helical repeat of double-stranded DNA, consistent with the idea that evolution has only retained IESs that can form a double-stranded DNA loop during assembly of an excision complex. We propose that the ancient domestication of a *piggyBac* transposase, which provided a precise excision mechanism, enabled transposons to subsequently invade *Paramecium* coding sequences, a fraction of the genome that does not usually tolerate parasitic DNA.

## Introduction

*Paramecium* belongs to the ciliate phylum, a deep radiation of highly diverse unicellular eukaryotes. The hallmark of ciliates is nuclear dimorphism: each unicellular organism harbors two kinds of nuclei with distinct organization and function. A diploid “germline” micronucleus (MIC) undergoes meiosis and transmits the genetic information to the next sexual generation but is not expressed. A polyploid “somatic” macronucleus (MAC) contains a version of the genome streamlined for gene expression and determines the phenotype. A new MAC is formed at each sexual generation by programmed rearrangements of the entire zygotic, germline-derived genome, and the maternal MAC is lost. The MAC genome of *P. tetraurelia* has been sequenced [1] revealing a series of whole genome duplications (WGDs) in the lineage that provide a unique tool for evolutionary analyses.

**Figure 1:**
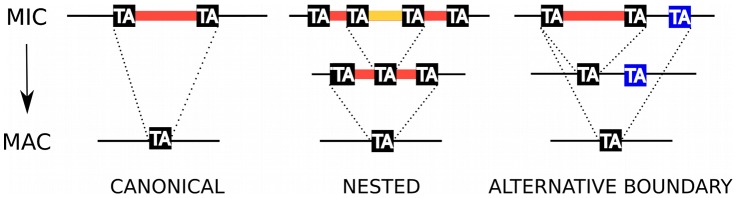
IES excision. Schematic representation of, from left to right, a canonical IES, a nested IES and an IES with an alternative boundary. In the case of the nested IES, the middle line represents either an intermediate in the excision pathway or an alternative final product. In the case of the alternative boundary IES, the middle line represents an alternative final product.

Ciliate genome rearrangements and their epigenetic control by non-coding RNAs have been recently reviewed [2]–[4]. In *Paramecium*, genome rearrangements involve (i) endoreplication of the DNA to about 800 haploid copies, (ii) imprecise elimination of genomic regions that contain, in particular, transposons and other repeated sequences, usually leading to chromosome fragmentation and (iii) elimination of Internal Eliminated Sequences (IES) by a precise mechanism. The accuracy of this process is crucial for IESs located within coding regions, to correctly restore open reading frames. The characterization of fewer than 50 IESs identified by cloning MIC loci [5] showed that they are short (26–883 bp), unique copy elements that are located in both coding and non-coding regions of the genome. The IESs are invariably flanked by two TA dinucleotides whereas only one TA is found at the MAC chromosome junction after IES excision (Figure 1). IESs have also been discovered by *cis*-acting mendelian mutations that prevent their excision, conferring a mutant phenotype [6]–[10]. The mutations in almost all cases were found in one of the flanking TA dinucleotides, which seem to be an absolute sequence requirement for IES excision. Extrapolation of the number of IESs found mainly in surface antigen genes led to the estimation that there could be as many as 50,000 IESs in the *Paramecium* genome. Such massive presence of unique copy IESs inserted in genes is not a characteristic of all ciliates. The estimated 6,000 IESs of the related oligohymenophorean ciliate *Tetrahymena* [11] are excised by an imprecise mechanism [12], are usually multi-copy including recognizable transposons [13]–[15] and are rarely found in coding sequences [16], [17].

Klobutcher and Herrick [18] first reported a weak consensus at the ends of 20 IESs from *Paramecium* surface antigen genes (5′-TAYAGYNR-3′) that resembles the extremities of Tc1/mariner transposons. These authors hypothesized a “transposon link” to explain the origin of IESs, suggesting that they are the decayed relics of a Tc1/mariner transposon invasion and that they are excised from the MAC DNA by a Tc1/mariner transposase encoded by a gene that has become part of the cellular genome [19]. In this model, IES excision represents the exact reversal of Tc1/mariner transposon integration into its TA target site with duplication of the TA dinucleotide, an evolutionary novelty that may have appeared more than once in the ciliate phylum. One problem with the model is that transposition catalyzed by Tc1/mariner transposases usually leaves a 2 or 3 bp “footprint” at the donor site [20] while IES excision is precise.

A decisive step towards understanding the mechanism of IES excision and validating a transposon link for the origin of the IES excision machinery was the identification of a domesticated *piggyBac* transposase in *Paramecium* [21]. Baptized PiggyMac (Pgm), the protein is encoded by the *PGM* gene which is expressed only late in sexual processes, at the time of genome rearrangements. Pgm, localized in the developing new MAC, was found to be required for the excision of all IESs tested and for the imprecise elimination of several regions containing transposons or cellular genes [21]. A similar *piggyBac*-derived transposase is found in *Tetrahymena* and is required for heterochromatin-dependent DNA elimination [22]. Since the *Paramecium* and *Tetrahymena* proteins appear to be monophyletic, based on a broad phylogeny of *piggyBac* transposases (L. Katz and F. Gao, personal communication), the domestication event may have preceded the divergence of these two ciliates, estimated at 500–700 Ma (million years ago) [23]. Most significantly, the *in vivo* geometry of IES excision, initiated by staggered double-strand breaks (DSBs) that generate 4-base 5′ overhangs centered on the TA at both ends of the IES [24], is fully compatible with the *in vitro* reaction catalyzed by a *piggyBac* transposase isolated from an insect [25], whose target site is a 5′-TTAA-3′ tetranucleotide. *piggyBac* elements leave behind no scar when they jump to a new location: only ligation is required to join the fully complementary 5′ overhangs. Limited processing of 5′ and 3′ ends is further required for precise closure of the *Paramecium* IES excision sites since only the TA dinucleotides at the center of the 4-base 5′ overhangs are always complementary [24], [26].

We report here a genomic approach to exhaustively catalogue the IESs in the *Paramecium tetraurelia* germline genome in order to study their evolutionary dynamics and seek evidence for a transposon origin of these elements. We obtained DNA highly enriched in un-rearranged germline sequences, from cells depleted in Pgm by RNA interference. Deep-sequencing of this DNA (hereafter called “PGM DNA”) allowed us to identify a genome-wide set of nearly 45,000 IESs, by comparing contigs assembled using the PGM DNA (hereafter called “PGM contigs”) with the MAC reference genome [1]. The hypothesis that Pgm is required for excision of all IESs was tested by genome-scale sequencing of a source of DNA from purified MICs [27], providing validation of the IES catalogue. The evolutionary dynamics of the IESs was studied by exploiting the series of WGDs that have been characterized in *Paramecium* [1]. The study provides, to our knowledge, the first genome-wide set of IESs, in *Paramecium* or any ciliate, and provides new evidence that IESs have deleterious effects on fitness and that at least a fraction of IESs do derive from Tc1/mariner transposons that have decayed over time. The IES sequences evolve rapidly. The constraints we could detect concern their size distribution, suggestive of the assembly of a transpososome-like excision complex and a weak consensus at their ends, which resembles the extremities of Tc1/mariner elements. We discuss the possibility that ancient domestication of the Pgm transposase favored subsequent propagation of transposons throughout the *Paramecium* germline genome, by providing a mechanism for their precise somatic excision, therefore allowing insertions in coding sequences.

## Results

### IES identification

An overview of the strategy for identification of a genome-wide set of IESs is presented in Figure S1. The first step was next-generation deep sequencing of DNA enriched in un-rearranged sequences, isolated from strain 51 cells that had undergone the sexual process of autogamy after depletion of Pgm protein by RNAi (Figure S2). In the absence of Pgm, the zygotic DNA is amplified but rearrangements are impaired. The sample that was sequenced contained a mixture of 60–65% un-rearranged DNA from the developing new MACs and 35–40% rearranged DNA from the fragments of the maternal MAC still present in the cytoplasm, as judged by Southern blot quantification of MIC and MAC forms at one locus (Figure S3). The PGM sequence reads (Table 1) were mapped to the MAC reference genome of strain 51 (see Materials and Methods), and putative IES insertion sites were defined as sites with a local excess of ends of read alignments (pipeline MIRAA for “Method of Identification by Read Alignment Anomalies”). This excess of ends of alignments arises when a read contains a MIC IES junction, since only part of such a read can align with a MAC chromosome, either starting or ending at the IES insertion site, expected to be a TA dinucleotide. Using MIRAA, we identified 45,739 potential IES insertion sites. Essentially all (99%) of the insertion sites contained a TA dinucleotide, even though this was not assumed by the pipeline.

**Table 1:** Sequencing and mapping statistics.

DNA	Insert size (bp)	Read length (bp)	Reads	Aligned reads	Aligned (%)	Genome coverage (%)
PGM	∼500	108	130,266,728	110,189,736	84.6	99
Lambda-phage	∼200	101	83,149,385	25,949,607	31	44

Paired-end Illumina sequencing was carried out as described in Materials and Methods, and reads were mapped to the *P. tetraurelia* MAC reference genome using the BWA short-read aligner. The genome coverage is the fraction of the genome covered by at least 1 read. The depth of coverage with the PGM DNA is on average 165×. The depth of coverage with the lambda-phage DNA is on average 75× for the part of the genome that is covered. The PGM reads that were not aligned contain *Paramecium* mitochondrial and rDNA sequences, contaminating bacterial sequences as well as sequences present only in the MIC genome. In addition, a large proportion of the unaligned lambda-phage reads are from bacterial contaminants with AT-rich genomes; this DNA was not eliminated by the cesium chloride density gradient separation step of the phage library construction [28].

In order to obtain the sequence of the IESs, the paired-end PGM DNA sequence reads were assembled into contigs (cf. Table S1 for assembly statistics) and compared to the MAC reference genome assembly (pipeline MICA for “Method of Identification by Comparison of Assemblies”). We looked for insertions in the PGM contigs with respect to the MAC reference assembly. Any insertion bounded by TA dinucleotides after local realignment was considered to be an IES. Using this pipeline we identified 44,928 IESs. The fact that 96% (n = 43,220) of the IESs identified by MICA correspond to an IES insertion site identified by MIRAA (Figure S1) testifies to the overall reliability of the procedure. Experimental validation of 6 IESs identified only by MICA and 17 insertion sites identified only by MIRAA was carried out by PCR amplification of an independent preparation of PGM DNA. The results (Table S2) show that the 6 IESs and at least 12 of 17 insertion sites tested do correspond to the presence of an IES. Interestingly, among the IES sites identified only by MIRAA, we found 8 examples of a pair of IESs separated by one or only a few nucleotides (in 5/8 cases, these tandem IESs are located in exons, a proportion similar to that found for the genome-wide IES set, see below). This case is not handled by the MICA pipeline since the initial global alignment with BLAT would have detected a single large insertion that would have been rejected by the local realignment filter, which requires the insertion to be flanked by TA dinucleotides. This is the first report of such closely spaced IESs, although nested IESs (Figure 1) have been previously documented [8].

In order to see whether the set of 44,928 IESs is likely to be exhaustive, we looked for the 53 previously characterized IESs identified directly by cloning MIC loci in *P. tetraurelia* strain 51 cells (Table S3). All 53 previously cloned IESs were found, with the exception of one IES that had been assembled into the MAC reference genome and one IES form that represents use of an alternative boundary. In addition, two small IESs, each of which is nested within a larger IES, were found in PGM DNA but were not identified by our pipeline as IESs. Indeed, nested IESs can only be identified by time-course experiments or if the outer IES is retained in the MAC e.g. as the result of a point mutation [8]. Since 49 of 51 non-nested IESs were identified by MICA, the IES identification procedure has a sensitivity of at least 96%.

The entire IES identification approach is based on the assumption that the excision of all IESs in *Paramecium* requires the Pgm domesticated transposase activity. In order to test this assumption, we sequenced inserts from a lambda-phage library constructed some 20 years ago [28], using DNA from MICs that had been separated from MACs by Percoll gradient centrifugation [27]. This library has been extensively used to clone MIC loci with specific probes. Although the contigs assembled from the phage DNA reads only partially covered the MAC reference genome (Table 1), 98.5% of the 13,377 IESs that could be identified using the phage DNA and the MICA pipeline had also been identified using the PGM DNA. The difference of 1.5% is within the estimated sensitivity of the MICA pipeline. We conclude that all *Paramecium* IESs very likely require Pgm for excision, and that our data set does represent a genome-wide set of *P. tetraurelia* IESs.

### IES distribution in the genome

The genome-wide set of IESs has an overall G+C content of 20%, significantly lower than the 28% G+C content of the MAC reference genome [29] but comparable to the G+C content of intergenic regions (21%). The IESs are found in exons (76.8%), introns (5.4%) and intergenic regions (17.8%), suggesting a nearly random distribution of IESs with respect to genes, since the MAC reference genome is composed of 76% exons, 3.2% introns and 20.8% intergenic DNA [1]. However, IESs are not randomly distributed along the chromosomes. Intriguingly, as shown in Figure S4 for the 8 largest MAC chromosomes, IESs tend to be asymmetrically distributed along MAC chromosomes. The MAC assembly (188 scaffolds >45 Kb constitute 96% of the 72 Mb assembly) contains 115 telomere-capped scaffolds, varying in size from ∼150 Kb to ∼1 Mb, that are considered to represent complete MAC chromosomes. For 70 of these telomere-capped scaffolds, IESs display non-uniform distributions (p<0.002, median scaffold size 417 Kb) while for the remaining 45 telomere-capped scaffolds, the IES distribution is uniform (median scaffold size 275 Kb). Thus the larger the MAC chromosome, the greater the chance of observing a non-uniform IES distribution. The distributions for all scaffolds are easily visualized using the ParameciumDB [30] Genome Browser. The significance of the asymmetry in IES distribution is not clear, but might be related to the global organization of MIC chromosomes, currently unknown (discussed in [29]).

### Germline Tc1/mariner transposons

The genome-wide set of IESs covers 3.55 Mb (mean IES size 79 bp), compared to 72 Mb for the MAC reference genome assembly. The IESs thus add about 5% to the sequence complexity of the part of the MIC genome that is collinear with MAC chromosomes. The total complexity of the PGM contigs (after elimination of contigs with low PGM read coverage and high G+C content, assumed to represent bacterial contamination as confirmed in many cases by BLASTN matches against bacterial genomes) is ∼100 Mb, however the use of a single paired-end sequencing library with small inserts (∼500 bp) may have perturbed assembly of repeated sequences, possibly leading to underestimation of repeated sequence content. We infer that ∼25 Mb of germline-specific DNA corresponds to the imprecisely eliminated regions located outside of the MAC-destined chromosomes i.e. the part of the MIC genome that is not collinear with MAC chromosomes.

We have not further characterized this fraction of the PGM DNA. However, we did identify the first germline *P. tetraurelia* Tc1/mariner transposons (Figure S5), by using the phage-lambda library of MIC DNA [28] to walk past the end of MAC scaffold_51, which bears the subtelomeric 51G surface antigen gene [31]. In all, 5 phage inserts and 4 cloned PCR products corresponding to part or all of different copies of the element downstream of the 51G surface antigen gene, named *Sardine*, were sequenced (EMBL Nucleotide Sequence Database accession numbers HE774468–HE774475) and a consensus for the ∼6.7 Kb transposon was constructed (Figure S5 and Text S1). The ends of the *Sardine* copies contain intact or partially deleted 425 bp terminal inverted repeats (TIRs) which are themselves palindromic, containing a unique, oriented region nested within outer inverted repeats (Figure S5). *Sardine* contains up to 4 ORFs. One ORF is a putative DD35E transposase of the IS*630*-Tc1 family, like the DDE transposases of the TBE and Tec transposons found in stichotrich ciliates [32]. Another ORF, as in Tec transposons [33], encodes a putative tyrosine recombinase. The other two ORFs are hypothetical, though ORF2 shows some similarity (31.7% identity and 55.4% similarity over 202 aa) to the hypothetical ORF1 of the *Tennessee* element from *P. primaurelia* [34]. One of the *Sardine* copies (copy S6) is interrupted by the insertion, within the putative tyrosine recombinase gene, of a different but similar element, named *Thon* (French for “tuna”), which also contains a DD35E transposase, a tyrosine recombinase, possibly the two hypothetical ORFs, and palindromic TIRs of ∼700 bp (Figure S5).

### IES copy number and similarity to transposon sequences

For a handful of IESs, it has been shown experimentally that they are single copy elements [5]. In order to see whether this is generally the case, we looked for all IESs present in more than 1 fully identical copy (100% sequence identity). We found 44,210 IESs to be unique copy (98.4%). We examined all IESs present in 2 or more identical copies and found 39 cases of duplicate IESs as a result of errors in assembly of the MAC reference genome that had led to small, partially redundant scaffolds (4% of the MAC assembly is contained in scaffolds <45 Kb and some of these are partially redundant with the chromosome-size scaffolds [1]). The rest of the 319 IESs found in 2 copies were inserted in homologous genomic sites and appeared to be the result of recent segmental duplication or gene conversion. The 23 cases of IESs found in 3 to 6 copies correspond to expansion or recombination of repeated sequences such as tetratricopeptide repeat (TPR) domains or WD40 repeats.

We performed an all by all sequence comparison of the IESs and of their flanking sequences to see whether we could identify homologous IESs inserted at non-homologous sites in the genome. As shown in Table 2, we were able to identify 8 clusters of 2 to 6 IESs that share significant homology (BLASTN E-value <10^−10^) over at least 85% of their length, inserted in non-homologous sites (cf. Text S2 for the alignments). Moreover, we found significant nucleotide identity (E-value 9×10^−57^ for the best match; nucleotide identity between 68 and 78% for the HSPs) between the IESs of cluster 5 and one of the Tc1/mariner-like transposons identified using the phage library (*Thon*, Figure S5). This is a strong indication that these IESs are derived from recently mobile elements.

**Table 2:** Homologous IESs at non-homologous sites in the genome.

CLUSTER	IES SCAFFOLD	IES POSITION	SIZE (bp)	LOCATION	NUCLEOTIDE MATCH
2	scaffold51_25	381101	209	GSPATP00009750001	
	scaffold51_25	389332	213	intergenic	
3	scaffold51_117	944	608	intergenic	
	scaffold51_160	10020	577	intergenic	
	scaffold51_44	7711	555	intergenic	
5	scaffold51_109	40673	571	intergenic	TIR *Thon*
	scaffold51_128	266698	689	GSPATP00032295001	TIR *Thon*
	scaffold51_131	262422	630	intergenic	TIR *Thon*
	scaffold51_18	127217	770	GSPATP00007326001	TIR *Thon*
	scaffold51_34	280841	512	intergenic	TIR *Thon*
	scaffold51_58	302214	640	intergenic	TIR *Thon*
9	scaffold51_19	475992	666	intergenic	
	scaffold51_96	236752	665	intergenic	
12	scaffold51_124	248174	568	intergenic	
	scaffold51_27	275392	476	GSPATP00010339001	
13	scaffold51_155	211807	458	intergenic	
	scaffold51_20	46790	505	intergenic	
	scaffold51_27	294496	472	GSPATP00010351001	
14	scaffold51_184	21279	1024	GSPATP00038454001	
	scaffold51_21	430950	1038	GSPATP00008497001	
	scaffold51_58	200038	1010	GSPATP00018841001	
15	scaffold51_28	278632	262	GSPATP00010625001	
	scaffold51_4	361312	242	GSPATP00001801001	

A BLASTN internal comparison of all IESs, carried out with an E-value cutoff of 1e-10, was filtered for HSP coverage of at least 85% of the longest IES and for the absence of significant homology between 500 nt of MAC flanking sequence. The IESs were than transitively clustered and aligned using MUSCLE (Text S2). Some clusters were eliminated because of low complexity of the IES sequences. BLASTN homology searches at NCBI and against known Paramecium transposons ([34] and the present manuscript) were carried out using each IES in the clusters as query. *Thon* is a Tc1/mariner-like transposon. BLASTX similarity searches against the non-redundant protein database at NCBI did not yield any significant hits at an E-value cutoff of 0.001. The location of the IES, if in a coding sequence, is provided as a ParameciumDB accession number.

However, the IES sequences of this cluster correspond to a single palindromic TIR. This might reflect assembly problems given use of a single insert size for the paired-end sequencing, either because these IESs contain sequences repeated elsewhere in the genome or because the *Thon* TIRs are large (∼700 bp) and palindromic so that the assembly might have jumped from one TIR to the other deleting the rest of *Thon*. We therefore used a long-range PCR strategy capable of amplifying large DNA fragments containing each of the IESs to verify their size and attempt to obtain sequences (detailed in Text S3). Amplification products of the expected sizes were obtained for all of the IESs from cluster 5, making it unlikely that these IESs correspond to a complete *Thon* element that had failed to be assembled from the paired-end sequencing reads. Three IESs were chosen for sequencing, and the sequences of the corresponding PCR products confirmed the IESs, indicating that they had been correctly assembled. Identification of 6 IESs (at non-homologous genomic sites) that share sequence identity with a *P. tetraurelia* Tc1/mariner solo TIR argues that at least a fraction of IESs do originate from Tc1/mariner-like elements.

We therefore adopted a complementary strategy, using the PFAM-A library of curated protein domains to search for domain signatures in the genome-wide set of IESs. Matches at a BLASTX E-value cutoff of 1 were inspected visually to filter out matches with PFAM-A protein domains from *Paramecium* and matches owing to compositional bias (high A+T content). This left 6 IESs, ranging in size from 2416 to 4154 bp, with a DDE_3 (PFAM accession number 13358) DDE superfamily endonuclease domain characteristic of IS*630*/Tc1 transposons. The peptides encoded by the IESs were subjected to an HMM search of the PFAM-A hmm profiles (http://pfam.sanger.ac.uk/search) for confirmation of the conserved residues and to validate the statistical significance of the match (E-values of 0.02 to 2.1×10^−15^ for the 6 peptides). The IESs were aligned with MUSCLE and a neighbor-joining tree grouped 4 of them together with good bootstrap values (not shown). The 4 IESs were used to search for sequence similarity with the genome-wide set of IESs and this allowed identification of 28 IESs ranging in size from 1251 to 4154 bp (Table S4). The IESs were aligned to provide the consensus sequence for 2 distinct Tc1/mariner-like 3.6 kb transposons from the same new family, baptized *Anchois* (Anchovy). Manually adjusted alignments used to reconstruct the *AnchoisA* and *AnchoisB* elements, consensus sequences and annotation are provided in Text S1.

**Figure 2:**
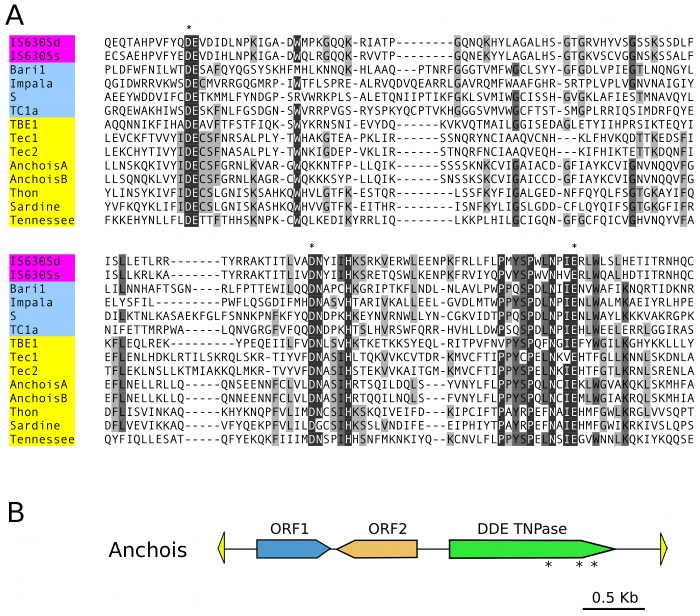
*Anchois* Tc1/mariner family transposon. A) Alignment of the DDE domains of bacterial IS*630* elements (IS*630*Sd, *Salmonella dublin*, GenBank Accession No. A43586; IS*630*Ss, *Shigella sonnei*, X05955), invertebrate and fungal Tc1 transposons (Baril, *D melanogaster*, Q24258; Impala, *Fusarium oxysporum*, AF282722; S, *D* melanogaster, U33463; Tc1, *C elegans*, X01005) and ciliate Tc1/mariner transposons (TBE1, *Oxytricha fallax*, L23169; Tec1 and Tec2, *Euplotes crassus*, L03359 and L03360; Anchois, Thon and Sardine, *Paramecium tetraurelia*, this article; Tennessee, *Paramecium primaurelia*, [34]). Asterisks mark the conserved catalytic DDE residues. B) Schematic diagram of the 3.6 Kb *Anchois* consensus, showing the position and orientation of the 3 ORFs. The yellow triangles represent the ∼22 nt TIRs. Asterisks mark the position of residues of the catalytic DDE triad for the ORF encoding the DDE transposase.

Alignment of the DDE domains of the reconstituted *Anchois* transposons with the DDE domains from bacterial IS*630* elements, invertebrate Tc1 transposons and all known ciliate Tc1/mariner elements indicates that the *Anchois* transposase belongs to the IS*630*/Tc1 subfamily (Figure 2A). Unlike *Thon* and *Sardine* but like the *P. primaurelia Tennessee* element, *Anchois* TIRs are short and lack internal palindromes, moreover *Anchois* does not contain a putative tyrosine recombinase. *Anchois* has 2 hypothetical ORFs in addition to the DDE transposase (Figure 2B; Text S1). The ORF2 of *Anchois* displays homology to ORF2 of *Sardine* (36.2% identity and 56.2% similarity over 210 aa) and to ORF1 of *Tennessee*. Interestingly, for 6 of the 28 IESs that initially identified the copies of *Anchois*, the *Anchois* TIRs do not correspond to the extremities of the IES, raising the possibility of *Anchois* insertions within pre-existing IESs. The discovery of the *Anchois* elements and the fact that several IESs appear to be full-length copies, provides a strong, direct link between IESs and transposons.

### A remarkable IES size distribution

**Figure 3:**
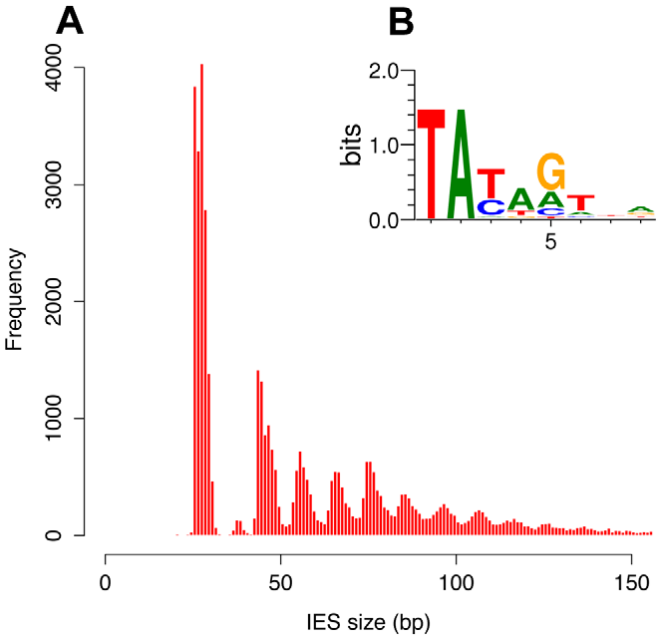
IES sequence properties. A) Histogram of the sizes of the genome-wide set of IESs that are shorter than 150 bp. B) Sequence logo showing information content at each position, corrected for a G+C content of 28%, for the ends of the genome-wide set of IESs.

The size distribution of the genome-wide set of IESs is shown in Figure 3A, for the 93% of the IESs that are shorter than 150 bp. The most remarkable feature is a periodicity of ∼10 bp, which corresponds to the helical repeat of double-stranded DNA. The first peak of the size distribution has maximal amplitude at 26–28 bp and includes 35% of all identified IESs. The abrupt cutoff at 26 bp represents the minimum IES size. A second peak appears to be forbidden and contains only a few IESs. The following peaks are centered at approximately 45–46, 55–56, 65–66 bp etc. and the distance between these peaks is best fit by a 10.2 bp sine wave (not shown). At the far end of the spectrum, 95 of the IESs are between 2 and 5 Kb in size. Similar periodic size distributions are found for IESs inserted in coding sequences and for IESs inserted in non-coding sequences (Figure S6). This indicates that the constraint on the distance between IES ends is an intrinsic property of the IESs and is not related to the locus in which they are inserted in the genome. Whatever their size, the IESs adhere to the weak, Tc1/mariner-like end consensus first reported for 20 IESs located in surface antigen genes [18], as illustrated in Figure 3B for the whole set. Differently sized subsets of the IESs all display essentially the same end consensus (data not shown).

**Figure 4:**
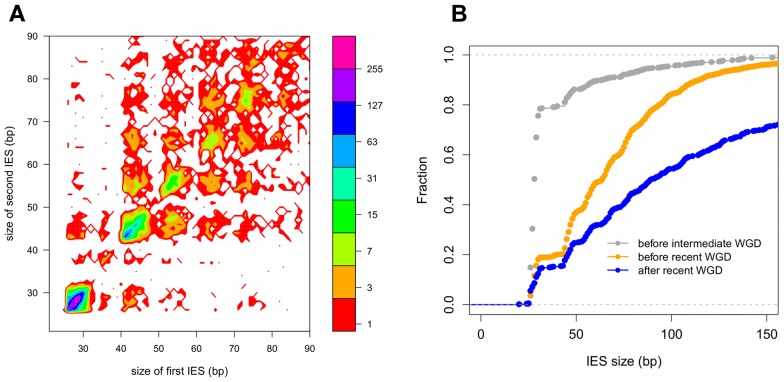
IES conservation in genes related by WGD. A) Filled contour plot of the correlation between the size of IES pairs that have been conserved with respect to the recent WGD. The x axis gives the size in bp of the first IES, the y axis gives the size in bp of the second IES found in the ohnologous gene and the color of each point indicates the number of times that combination of x,y values was found in the data set. The color legend is shown to the right of the figure, the numbers represent counts of the x,y value pairs; the rainbow colors are distributed according to a log2 scale. B) Size distribution of IESs conserved in “quartets” i.e. genes that are still present in 4 copies in the genome after duplication at both the intermediate and the recent WGD events. In order to compare size distributions for different classes of IES, they are represented as experimental cumulative distribution functions. The ripples in each curve correspond to the peaks of a histogram representation as in Figure 3A. The curves are for IESs that must have originated from an ancestral IES acquired before the intermediate WGD (grey, N_1111_ IESs), IESs that must have originated from an ancestral IES acquired before the recent WGD (orange, N_1100_ IESs) and the IESs that might have been acquired since the recent WGD (blue, N_1000_ IESs).

We further examined constraints on IES size and sequence by evaluating IES conservation with respect to the 3 WGDs in the *Paramecium* lineage. We used the large number of paralogs (hereafter termed “ohnologs”) of different ages (Table 3) that could be identified for each of the WGD events [1] to ask whether IESs are present, at the same position relative to the gene coding sequences, in ohnologs of the different WGD events. This analysis makes the assumption that IES insertions are rare events so that if IESs are present at the same position in ohnologous genes, then they must have been acquired before the WGD and can be considered to be “ohnologous” IESs. As shown in Table 3, we found 84.5%, 23.2% and 5.9% conservation of IESs with respect to the recent, intermediate and old WGDs respectively. For comparison, more than 99% intron conservation was found for 1,112 pairs of genes related by the recent WGD [35]. This indicates that the dynamics of IES insertion or loss over evolutionary time is relatively fast compared to that of introns. The only phylogenetic study of IESs, carried out for two loci in a few different stichotrich (formerly called hypotrich) ciliates, which are very distantly related to *Paramecium*, also concluded that the intragenic IESs in those species evolve very rapidly [36]. We found that the ohnologous IESs related by the recent WGD are highly divergent in sequence. In more than 90% of cases, the sequence identity was too low for detection by BLASTN (E-value threshold of 10^−5^). This high level of sequence divergence is consistent with the pattern expected for neutrally-evolving non-coding regions, since the average synonymous substitution rate measured between ohnologous genes derived from the recent WGD is about 1 substitution per site [1]. However, if we compare the lengths of IESs that are conserved with respect to the recent WGD (Figure 4A), for ∼55% of the pairs, both IESs are found in the same peak of the IES size distribution. The honeycomb appearance of the plot (Figure 4A), with off diagonal cells that result from ohnologous IESs in different peaks of the distribution, underscores the strong evolutionary constraint that is exerted on IES size.

**Table 3:** IES conservation in ohnologs produced by the different WGDs.

WGD event	Genes with ohnolog	IESs	Conserved IESs	% conserved
Recent	24052	20623	17430	84.5
Intermediate	12590	11561	2675	23.2
Old	3381	3646	215	5.9

The identification of ohnologs and the reconstitution of the pre-duplication genomes is described in [1]. For the most recent WGD, which preceded the appearance of the *P. aurelia* complex of 15 sibling species [95], 51% of the pre-duplication genes are still present in 2 copies. For the intermediate duplication, 24% of pre-duplication genes are still present in 2 or more copies. For the ancient duplication, which preceded the divergence of *Paramecium* and *Tetrahymena*, 8% of pre-duplication genes are still present in 2 or more copies. The significance of the column headers is as follows. Genes with ohnolog: the number of present day genes with at least one ohnolog from the indicated WGD event. IESs: the number of IESs found in the genes with at least one ohnolog from the indicated WGD event. Conserved IESs: number of IESs found at the same position in at least one other ohnolog, as determined by sequence alignment. The identification of ohnologs is described in [1] and the data are available through ParameciumDB [90]. Note that this analysis only concerns IESs that are within paralogous genes and not IESs found in intergenic regions.

### Dynamics of IES gain and loss

In order to investigate the rate of IES insertions and losses during the evolution of the *Paramecium* lineage, we examined gene families, which we call “quartets”, for which all 4 ohnologs issued from duplication of an ancestral gene at the intermediate and then the recent WGD are still found in the present day genome. Of the 1350 such quartets identified in the MAC genome [1], 878 contain at least one IES in at least one of the 4 duplicated genes. We evaluated the conservation of IESs at the same position with respect to the coding sequence for all members of each quartet (Figure S7), and identified 2126 IES groups, each group containing an IES conserved either in all 4 genes (N_1111_ = 190), in 3 genes (N_1110_ = 64), in 2 genes on the same intermediate WGD branch (N_1100_ = 1304), in 2 genes on different branches (N_1010_ = 10) or in only one of the 4 genes (N_1000_ = 558).

Under the assumption that two IESs present at the same location in ohnologous genes derive from a single ancestral IES (i.e. the probability of two insertion events occurring at the same site after a WGD is considered negligible), and that the rate of IES losses has remained constant, it is possible to estimate the rate of IES gain during the evolution of the *Paramecium* lineage (the model is developed in Text S4). The quartet analysis is fully consistent with a model whereby IES acquisition has been ongoing since before the intermediate WGD (15% of the IESs predating this WGD), with a peak in the period between the intermediate and the recent WGD events: 69% of IESs were acquired during the interval between these two WGDs, vs. 16% during the period since the recent WGD, which corresponds to about the same evolutionary time. Genome-wide IES data for other *Paramecium* species will be necessary in order to test the assumption of a constant rate of IES losses. However, even if we relax this assumption (i.e. rates of IES losses are allowed to vary over time), the model still strongly rejects the hypothesis that all IESs were acquired before the intermediate WGD (cf. Text S4). Thus, with the presently available data and biologically reasonable assumptions, we conclude that IESs have been acquired in all 3 of the time periods delimited by the intermediate and recent WGD events.

We compared the cumulative size distributions of the N_1111_, N_1100_ and N_1000_ IESs (Figure 4B). The N_1111_ IESs, which must have been acquired before the intermediate WGD, are much shorter than the IESs of the two other samples, with almost 80% of the IESs in the first peak, compared to 20% for N_1100_ IESs, which may mainly result from IES acquisition after the intermediate but before the recent WGD, and only 16% for N_1000_ IESs, at least some of which may have been acquired since the recent WGD. In addition, the curves are significantly shifted with respect to each other, in particular, 30% of the N_1000_ IESs are larger than 150 nt, compared to scarcely any IESs larger than 150 nt for the two other samples. This analysis shows that the older an IES, the shorter it is likely to be, consistent with a decay process involving progressive shortening of IESs by accumulation of small deletions, in addition to the accumulation of point mutations.

Quartet analysis is restricted to IESs in genes that have been retained in 4 copies (fewer than 10% of all IESs). Similar distributions of IES size are found if we consider all ohnologous IESs (45% of all IESs, cf. Table 3). IESs conserved with respect to the intermediate WGD (76% of IESs in first peak) are significantly shorter than IESs conserved only with respect to the recent WGD (30% of IESs in the first peak) (data not shown). The size distribution of IESs conserved with respect to the old WGD is poorly determined because of the small number of conserved IESs (Table 3), which are moreover often in genes that have undergone recent gene conversion judging from the nucleotide divergence of the ohnologs (data not shown). It is therefore uncertain that IESs were present in the genome before the old WGD, consistent with the absence of TA-bounded IESs in *Tetrahymena*, which diverged from *Paramecium* after the old WGD event [1].

Since we found essentially no IESs shorter than 26 bp, it seems likely that some mechanism(s) other than decay of the sequence through internal mutations and deletions is responsible for the complete loss of an IES. In order to explore this question, we examined case by case, using both nucleotide and conceptual protein alignments, all of the N_1110_ quartet IES groups (n = 64), which are most parsimoniously explained by insertion of an IES before the intermediate WGD followed by loss of an IES after the recent WGD. We examined the raw read alignments and PGM and phage contigs in order to be sure that there was sufficient read coverage and no evidence suggesting presence of an IES at any site of putative IES loss. We found 4 different explanations for the quartet triplets: precise loss of the fourth IES (n = 17), gain of the third IES by gene conversion between intermediate WGD ohnologs (n = 1), recruitment of the fourth IES into the exon sequence (n = 6), and deletion of the region that encompasses the fourth IES (n = 23), often testifying to the formation of a pseudogene. In addition, we found 5 errors in IES detection (the fourth IES probably exists as it can be found in the phage contigs or is predicted by the MIRAA pipeline). In the remaining cases (n = 12), annotation or alignment problems made it difficult to conclude. The observation of 17 cases of precise loss of an IES from the germline DNA raises the possibility that there is a mechanism for conversion of a MIC locus to the IES-free form using a MAC genome template. However, we cannot rule out the possibility that IESs can be precisely excised from the MIC DNA, and therefore lost, by the same Pgm-dependent mechanism as that involved in MAC genome assembly.

### TA-indels reveal IES excision errors

**Figure 5:**
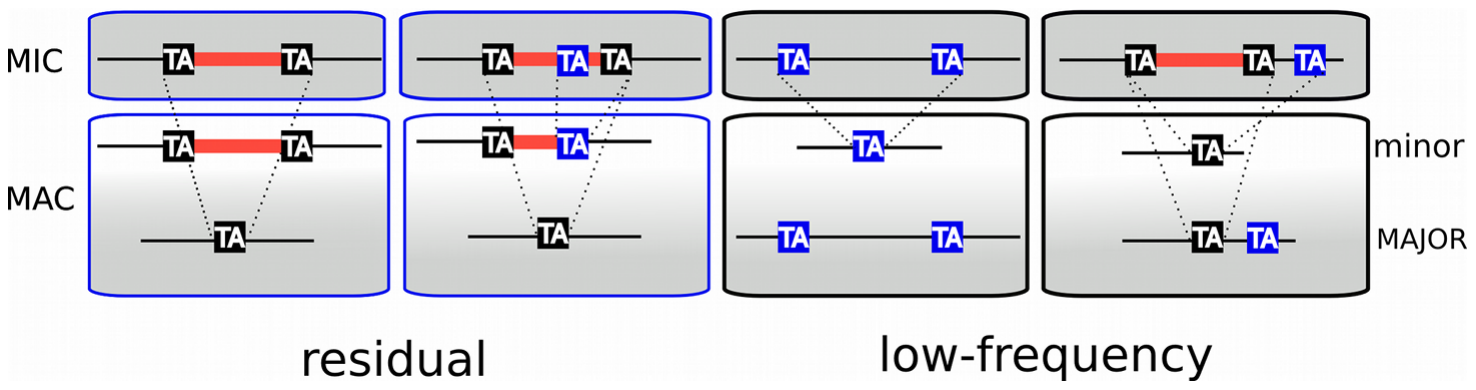
TA-indels are produced by IES excision errors. Schematic representation of the “residual” and “low frequency” TA-indels that were identified by comparing the MAC draft genome assembly (MAJOR form) with the 13× Sanger sequencing reads used to build the assembly [29]. The TA-indels were identified by one or more reads that differed from the assembly (minor form). The residual TA-indels were assumed to be the result of occasional failure to excise an IES and the low-frequency TA-indels to result from excision of MAC-destined sequences. Comparison of the genome-wide set of IESs with the TA-indels revealed that many TA-indels result from the use of alternative IES boundaries situated inside the corresponding IES in the case of residual TA-indels and outside the IES in the case of low-frequency TA-indels. In the schema, TA dinucleotides in black boxes are *bona fide* IES boundaries while TA dinucleotides in blue boxes are alternative IES boundaries.

The analysis of sequence variability in the polyploid (800n) MAC genome, carried out by comparing the MAC assembly representing a “consensus” sequence with the 13× Sanger sequencing reads used to build the assembly, revealed nearly 2000 “TA-indels” that were presumed to be produced by the IES excision machinery and to reflect excision errors [29]. As shown schematically in Figure 5, “residual” TA-indels (n = 739), that were suggested to represent occasional retention of IESs on some macronuclear copies, were absent from the assembly (“major” form in Figure 5), but present in at least one sequence read (“minor” form). For 689 of the residual TA-indels (93%), we found an IES at the corresponding site in the genome. Interestingly, in 134 cases (19.4%), the TA-indel was shorter than the IES and case by case inspection indicated that most of these TA-indels may be products of IES excision that used an alternative IES boundary located within the IES (Figure 5). In this case, the TA-indel would only correspond to part of a larger IES. A few cases of use of an alternative IES boundary that may confer a mutant phenotype have been reported [7], [37].

“Low frequency” TA-indels (n = 1090), previously suggested to represent excision of MAC-destined sequences [29], were present in the assembly (major form, Figure 5), but absent from at least one sequence read (minor form). We could not look for the “low-frequency” TA-indels directly among the genome-wide set of IESs, since they are part of the MAC genome assembly. However, we examined the ends of the low-frequency TA-indels and found 249 cases (23%) where the TA dinucleotide at one of the ends corresponds to the insertion site of an IES in the genome-wide set (Figure 5), indicating that the TA-indel was generated by use of an alternative IES boundary located outside of the IES. The whole of the analysis supports the previous conclusion [29] that TA-indels are products of the IES excision machinery. The high incidence of alternative boundaries in both classes of TA-indels, revealed by comparing them with the genome-wide set of IESs, strengthens the previous conclusion [29] that TA-indels reflect IES excision errors (see below). Thus TA-indels cannot be considered to be IESs in the absence of further experimental support.

### Evidence for selective pressure against IES insertion

**Figure 6:**
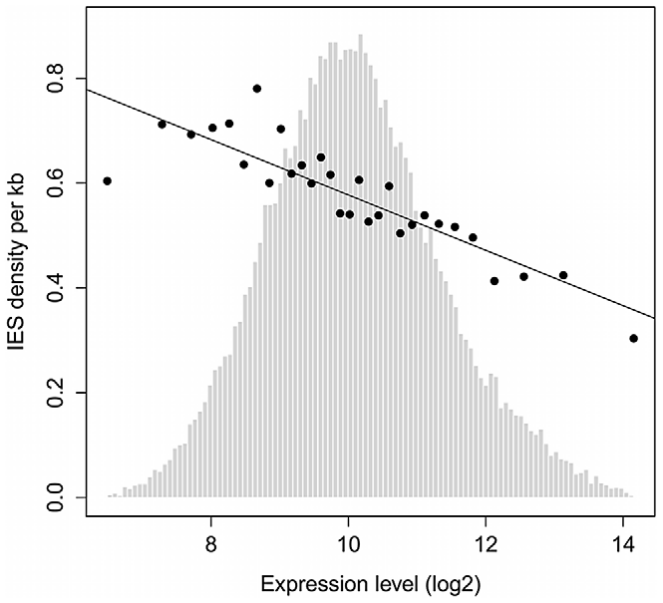
IES density is inversely proportional to gene expression level. Genes were binned according to their median expression level across 58 microarrays representing different cellular and growth conditions as described in [38], [39]. The expression levels were divided into 30 bins as in [38]. The black points show the average IES density (per Kb) of genes in each bin. Linear regression was used to fit the points. Light gray bars show the distribution of genes according to their expression level (before binning).

IESs are tolerated in coding sequences and evolve under a strong constraint on their size and end-consensus, properties that are presumably important for their precise and efficient excision. However, the excision machinery can commit errors, as revealed by TA-indels (cf. above) and by the use of alternative IES boundaries [7], [37]. We therefore looked for evidence that the rate of excision errors is high enough to represent a fitness burden for the organism. First, only 47% of genes contain at least one IES, and the IESs are less represented in strongly expressed genes. Figure 6 shows the density of IESs in genes as a function of gene expression level determined by microarray experiments [38], [39]. The density varies from about 0.7 IESs per Kb (i.e. an IES on average every 1.4 Kb) in genes with low expression to less than 0.3 IESs per Kb (i.e. an IES on average every 3.3 Kb) for the genes with the highest expression. The inverse correlation observed across all levels of expression indicates that IESs are less-well tolerated the more a gene is expressed.

Second, IESs inserted in protein-coding exons display a characteristic bias in their size. There is a statistically significant deficit in IESs whose length is a multiple of 3, compared to IESs found in non-coding regions. Furthermore, this bias is only found for 3n IESs that do not contain a stop codon in phase with the ORF of the upstream coding sequence (Table 4; cf. Table S5 for a more detailed analysis). A similar 3n bias was reported for introns in eukaryotic genomes, and experiments in *Paramecium* showed that the Nonsense Mediated Decay (NMD) pathway destroys mRNAs containing unspliced introns, provided the intron retention leads to a premature stop codon [35]. Retention in mRNA of a 3n stopless intron would not be detected by NMD and therefore could lead to translation of potentially harmful proteins, explaining the deficit in 3n stopless introns. The fact that IESs display a similar deficit suggests that the rate of IES retention is high enough to represent a fitness cost, so that IESs in exons are under selective pressure to be detected by NMD in case they are retained in the MAC genome. We were able to test this hypothesis by looking at the size bias for IESs located in the exons of the 25% of *Paramecium* genes that are the most highly expressed hence subject to the strongest selective pressure. As shown by the last 2 lines of Table 4 (samples Q1 and Q4), the deficit in 3n IESs is the greatest for the IESs found in the most highly expressed genes (28.3%), where IES retention would be the most deleterious.

**Table 4:** Deficit of 3n IESs in coding sequences.

IES Category	Number	3n	non-3n	?^2^	*P*-value
**Non-coding**	10304	3481 (33.78%)	6823 (66.22%)	-	-
**Coding stopwith**	11205	3700 (33.02%)	7505 (66.98%)	2.91	0.08
**Coding stopless**	23339	7095 (30.40%)	16244 (69.60%)	119.42	8.47×10^−28^
**Q1 stopless**	6044	1892 (31.30%)	4152 (68.70%)	16.61	4.59×10^−5^
**Q4 stopless**	5712	1615 (28.27%)	4097 (71.73%)	77.5	1.32×10^−18^

For the calculation of χ^2^, the observed numbers of IESs of length 3n and non-3n inserted in coding sequences are compared to the distribution found for IESs inserted in non-coding sequences under the null hypothesis that IES length is not under constraints related to translation. The null hypothesis is rejected only for those IESs inserted in coding sequences that do not contain a stop codon in frame with the upstream ORF (Sample “Coding stopless”). Microarray experiments [38] were used to group the IESs according to the expression level of the genes in which they are inserted. “Q1” designates IESs in exons of the 25% least expressed genes and “Q4” designates IESs in the exons of the 25% most expressed genes, those subject to the strongest selective pressure. The bias against 3n IESs is stronger in the Q4 sample than in the Q1 sample.

A more detailed analysis of the modulo 3 length distribution for IESs in coding and non-coding sequences, for each peak of the 10 bp periodic size distribution, is provided in Table S5.

## Discussion

### An IES reference set for *P. tetraurelia*

Previous studies of *Paramecium* IESs all relied on a small reference set of about 50 IESs. For the first time in any ciliate genome, in so far as we are aware, we have carried out an exhaustive identification of IESs. Since it is not yet possible to isolate *Paramecium* MICs in the quantity and of the purity required for genomic sequencing, we relied on nuclear DNA isolated from cells depleted in Pgm, the domesticated transposase required for introduction of the DSBs that initiate IES excision [21]. We fortunately were able to use the only genomic library ever made from purified MICs [28] – but heavily contaminated by bacterial DNA – to obtain genome-scale evidence that Pgm is required for excision of all *Paramecium* IESs and to estimate that our IES reference set includes ∼98.5% of all IESs.

Although this IES reference set will prove useful for a variety of studies, it is important to keep two things in mind. First, the IES definition used here is necessarily a genomic definition involving comparison of MIC and MAC sequences. Our procedure does not allow identification of nested IESs (unless the external IES is retained in the MAC), or of any IES located in part of the MIC genome that is not collinear with MAC chromosomes. The complexity of the assembled PGM DNA is almost 100 Mb, although we could not properly assemble repeated sequences. We thus estimate that at least 25% of the germline is not collinear with the MAC chromosomes, and might contain unique copy IESs or transposons, the excision of which could only have been detected if the flanking region were retained in the MAC.

Second, this reference set does not provide information about the variability in IES excision patterns that might exist between different, though genetically identical, cell populations. Many IESs are under maternal, epigenetic control [40], [31], [41]. The genome scanning model [42] posits that every time *Paramecium* undergoes meiosis, the scnRNA pathway compares the maternal MIC, in the form of 25 nt scnRNAs [43], with the maternal MAC, in the form of long non-coding transcripts [44]. The scnRNAs that cannot be subtracted by base pairing with the long maternal transcripts are licensed for transport into the new developing MAC [45] where they target homologous sequences for elimination, probably via deposition of epigenetic marks on the chromatin (cf [3], [4] for recent reviews of genome scanning in *Paramecium* and *Tetrahymena*). The scnRNA pathway in theory provides a powerful defense mechanism against transposons that invade the germline and can explain the molecular basis of alternative MAC rearrangement patterns that are maintained across sexual generations [31], [40], [41], [46], [47]. Hence the following caveat: any genome-wide set of IESs is identified with respect to a particular MAC reference genome sequence. There can be no “universal” IES reference set for the species. Since IESs can be a source of genetic variation as discussed in [48], the IES catalogue we have established will make it possible to study this variation, for example by surveying IES retention in the MACs of geographic isolates and in stocks that have been experimentally subjected to different types of stress.

### Constrained IES size distribution and the IES excision complex

The remarkable sinusoidal distribution of IES sizes retained by evolution reflects strong constraint on the distance between IES ends. We assume that the selection is exerted through the excision mechanism, since the retention of an IES in the MAC can impair gene function. An IES that cannot be efficiently excised is expected to be counter-selected. We propose an interpretation of the IES size distribution based on its similarity with data generated by “helical-twist” experiments, which have provided evidence of DNA looping between distant protein-binding sites in various, mainly prokaryotic, DNA transaction systems (transposition, gene control, replication initiation, site-specific recombination, etc. reviewed in [49]). In these experiments, the distance between transposon ends [50], [51], repressor binding sites [52]–[55] or site-specific recombination sites [56] is varied, on plasmids or on the bacterial chromosome, and the activity of the system is measured *in vivo*. The observed periodicity in the length-dependence of the activity corresponds to the helical repeat of the DNA, since the same face of the double helix must interact with the protein at each end, and given the prohibitive energetic cost of twisting the double helix to fit the binding site to the protein. This is especially true for DNA fragments whose size is close to the persistence length of double stranded DNA (∼150 bp) or shorter. The persistence length, a physical measure of the bending stiffness of a polymer in solution, is the length above which there is no longer a correlation between the orientation of the ends of the molecule. For DNA longer than its persistence length, it becomes possible for the 2 ends to encounter each other to form a loop, without any external intervention.

Almost all (93%) of the IESs in the genome are shorter than the persistence length of DNA. The size distribution, which appears as a series of regularly spaced peaks, can be decomposed into three parts. The largest peak is centered on 28 bp but displays an abrupt minimum size cutoff at 26 bp. A second peak seems to be of forbidden size. Finally, there follow a series of peaks that are best fit by a sine wave with a ∼10.2 bp periodicity. In the helical-twist experiments, the amplitude of the measured biological activity peaks tends to decrease with decreasing distance between interacting sites. However, for the IES size distribution, the decay of IESs over time imposes the opposite tendency: the peak heights increase as IES size decreases.

**Figure 7:**
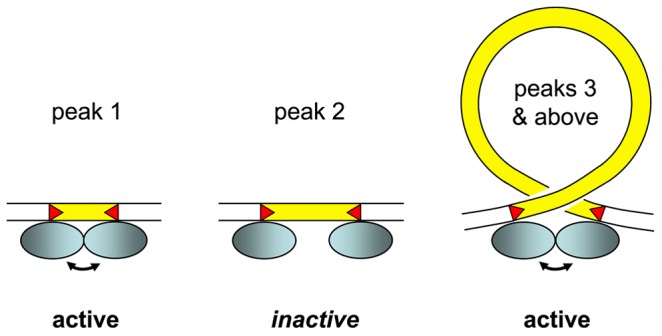
IES size constraint and the assembly of an active excision complex. Our working model is based on the assumption that oligomerization of the IES excisase (most likely the domesticated transposase PiggyMac) on DNA activates catalytic cleavage at IES ends (IESs are drawn in yellow and red triangles highlight the orientation of their ends). In the absence of any information on the stoichiometry of the complex, the excisase is represented by a shaded blue ellipse. For very short IESs from peak 1 (26–30 bp in length), the required contact between protein subunits may be established directly (double-headed arrow) and the complex is active. For IESs longer than 44 bp (peak 3 and above), we propose that looping of the intervening DNA double helix brings IES ends into close proximity and activates DNA cleavage. We have arbitrarily drawn the complex as an antiparallel arrangement of IES ends within a negatively supercoiled loop, but other conformations are possible. IESs from the “forbidden” peak 2 would be too long to allow direct contacts between protein subunits to be established, and too short to form an excision loop.

Our working model for assembly of an active IES excision complex is shown in Figure 7. We propose that, starting at the third peak (44–46 bp), the IESs assemble into the excision complex by forming a double-stranded DNA loop compatible with presentation of the same face of the double helix to the Pgm endonuclease at both IES ends. The near absence of the second peak, the minimum IES size of 26 nt and the 13 bp size of each *piggyBac* TIR [25] lead us to suggest that the IESs in the first peak are able to assemble an active excision complex without formation of a DNA loop. The IESs in the nearly absent second peak would not be efficiently excised, as they would be too short to form a DNA loop and too long to form an active excision complex without a DNA loop.

Molecular analysis of the IES excision mechanism supports the involvement of such a transpososome-type excision complex. First, the domesticated Pgm transposase, which has retained the catalytic site of *piggyBac* transposases [21], is very likely to be the endonuclease responsible for the cleavage reaction, involving the introduction of DSBs at each end of the IES [24]. Second, for IESs larger than 200 bp, covalently closed circular molecules containing the excised IES have been detected as transient intermediates during MAC development [57]. Third, if one end of an IES bears a mendelian mutation in the TA dinucleotide, no DSB occurs at either end of the IES. This indicates that the two IES ends must interact, directly or indirectly, before cleavage can occur [58].

It is worth noting that “canonical” TIRs of cut-and-paste transposons are often bipartite. They are composed of an internal sequence motif recognized and bound by the transposase, and of a few nucleotides at the termini that constitute the DNA cleavage site [59]. The obligatory conservation of a TA dinucleotide at IES ends is indicative of a requirement for DNA cleavage but is not sufficient for specific recognition, even if we take into account the weak consensus over the 6 internal nucleotides. The lack of a sufficiently long conserved motif in IESs makes it unlikely that Pgm recognizes IESs by binding to a specific sequence. For IESs under maternal control [31], it is currently thought that Pgm is recruited to its substrate via epigenetic marks deposited on the chromatin by the scnRNA pathway [3], [21], [42].

The picture of an IES excision complex that emerges from these considerations, which must of course be tested biochemically, requires very short pieces of DNA to form loops (Figure 7). Proteins that bend DNA, such as HMG proteins [60], could be involved. What is quite remarkable here, beyond the fact that evolution has performed such a nice “helical-twist” experiment, is that the DNA loops might be as short as ∼45 bp, shorter than almost any reported case of DNA looping. The minimal *in vivo* value reported for cut-and-paste bacterial transposons is 64–70 bp [50], [51] and this is also the minimum size reported for HMG assisted DNA loop formation *in vitro* [60]. The only indication of shorter loops comes from detection of a minor peak of activity *in vivo* and *in vitro* for ∼50 bp DNA loops in the *E. coli* Hin invertasome, provided that invertasome assembly occurs in the presence of HU, a bacterial nucleoid protein that bends DNA [56]. Given the unusually high A+T content of IESs (80%), local melting might favor the deformations in the double helix required to make the very small looped structures of the postulated IES excision complex.

### Evidence that IESs are remnants of transposons

Ciliate MICs have long been recognized as safe havens for transposons, since removal of the transposons from the somatic DNA during development would decrease the burden on host fitness, as discussed in [19]. Our study provides the first global vision of IESs in any ciliate germline and provides strong support for the “transposon link” hypothesis that present day IESs are remnants of transposons [18], [19].

Although we do not yet have a complete picture of the transposon landscape of the *P. tetraurelia* germline genome, we have identified 3 families of Tc1/mariner elements, with 2 quite different structures. The *Thon* and *Sardine* transposons have long, palindromic TIRs, a tyrosine recombinase and a DDE transposase characteristic of the IS*630*/Tc1 subfamily, with a short spacer (32 aa) between the 2^nd^ and 3^rd^ catalytic residues. This clearly distinguishes these transposons from the *piggyBac* family characterized by a long spacer and a DDD catalytic triad. The IESs related to these elements that we were able to identify appear as solo TIRs. Given the presence of repeated, palindromic subsequences in each TIR, we can speculate that the solo TIRs result from recombination between short direct repeats present within the complex TIRs, as proposed to explain the incidence of solo LTRs derived from LTR retrotransposons in the genomes of some organisms [61], [62]. The other transposon family we have identified, *Anchois*, is characterized by much shorter TIRs which do not contain internal palindromes, a similar DDE transposase and the absence of a tyrosine recombinase. This structure is similar to that of the *P. primaurelia Tennessee* transposon [34]. In the case of *Anchois*, we could find a number of IESs that appear to correspond to the entire transposon or large portions of it, including IESs with a recognizable but degenerate DDE transposase ORF.

It is possible that we have only scratched the tip of the iceberg since the germline genome is expected to contain other mobile elements. Indeed, we were able to identify 8 clusters of homologous IESs inserted at non-homologous genomic sites, suggesting recent mobility, and one of these clusters turned out to consist of IESs that are solo TIRs of the *Thon* element. The other clusters could be the remains of as yet unidentified elements. Both the *Thon* and the *Anchois* IES homologies were detected among the largest IESs in the genome-wide set (i.e. the 380 IESs >500 bp), and for none of them could we detect ohnologous IESs from the recent WGD, an indication that these IESs were recently acquired. Since over 90% of present day IESs have decayed to very short sizes (<150 bp) it is not surprising that internal transposon motifs can no longer be recognized. These very short IESs nonetheless display the short degenerate Tc1/mariner end consensus. The existence of this consensus at IES ends may testify to their evolutionary transposon origin. This end consensus would eventually have become a requirement for efficient cleavage by the IES excision machinery. We can imagine two instances of such convergent evolution: i) other families of mobile elements could be eliminated by the PiggyMac-dependent mechanism and ii) genomic sequences that adhere to the end consensus could be excised just like IESs. We conclude that at least a fraction of IESs are decayed Tc1/mariner transposons, and we consider highly probable that some IESs are derived from other mobile elements.

### IESs are a burden for host fitness

Since IES excision is not 100% efficient, IES insertions are in general deleterious, consistent with the different kinds of selective pressure we have observed: (i) a constrained IES size distribution likely reflecting assembly of the excision complex; (ii) a bias against IESs that do not lead to premature stop codons in case of IES retention in the MAC; (iii) an inverse correlation between IES insertions and gene expression level. IESs can in addition be considered to constitute a mutational burden, in the same way as introns are considered to constitute a mutational burden in intron-rich eukaryotic genomes [63], since IESs are present in large number in *Paramecium*, and any mutation in a flanking TA dinucleotide abolishes IES excision. Nonetheless, the system can give rise to beneficial new functions, as attested by use of the IES excision machinery to provide a regulatory switch for mating type determination (D. Singh, personal communication).

Since IESs are in general deleterious and constitute a fitness burden for the organism, and since we have detected cases of probable clean IES loss from the germline DNA suggesting that a mechanism exists for precise IES excision in the MIC, we may ask why *Paramecium* has any IESs at all. This question can be easily answered if we consider that IESs arise from selfish genetic elements (SGEs, defined as elements – typically transposable elements or viruses – that can enhance their own transmission relative to the rest of the genome, with deleterious or neutral effects for the host [64]). The number of IESs reflects the balance between the number of IES insertions (e.g. invasion by SGEs that subsequently decayed to become unique-copy IESs) and the strength of selection against these insertions, which either prevents fixation of new insertions in the population or favors loss of already fixed insertions. This genetic conflict is mediated by an “arms race” between SGEs and the host as discussed by Werren [64].

### Host defense mechanisms in ciliates

In all kingdoms of life, non-coding RNAs are used to defend host genomes against parasitic nucleic acids, as exemplified in eukaryotes by small RNA pathways involved in protection against viruses or in silencing transposons to ensure integrity of the germline genome [65]–[67]. In ciliates, nuclear dimorphism provides the potential for an additional layer of protection by physically separating the chromosomes that store the genetic information from the rearranged chromosomes that express the genetic information. Additional host defense machinery providing precise excision of transposons/IESs from somatic DNA, might have allowed the invasion of a fraction of the genome in which SGEs are not usually tolerated, namely the coding and regulatory sequences required for gene expression.

In the case of *Paramecium*, Pgm domestication has provided the mechanism for precise excision of TA-bounded insertions from the somatic DNA, allowing transposons/IESs to be cleanly excised from genes in the MAC. Since this would reduce the fitness burden caused by transposition, we presume that it allowed transposons to spread throughout the MIC genome. Recognition of the IESs is however ensured by the scnRNA pathway [3], itself an example of the more ancient mechanism of small RNA-based host immunity against foreign nucleic acids, and this epigenetic recognition may in part explain the less than 100% efficiency of IES excision.

In *Tetrahymena*, which has both a scnRNA pathway and domesticated *piggyBac*-like transposases [4], [22], only excision of intergenic IESs has been studied for the moment and use of heterogeneous cleavage sites was found. This imprecise excision would not be compatible with insertion in genes since gene expression would be compromised. *Tetrahymena* has only about 6,000 IESs and indeed, they are not usually found within genes [17]. Why doesn't *Tetrahymena* have intragenic IESs? We can only speculate that a Tc1/mariner invasion after the divergence of *Paramecium* and *Tetrahymena* was instrumental in the evolution of a precise excision mechanism in *Paramecium*, necessary for spread of these elements throughout the genome. In support of this hypothesis, a recent genome-scale identification of hundreds of *Tetrahymena* IESs [17] revealed a new class of TTAA-bound IESs that are precisely excised. They were found to contribute 3′ exons to genes that are expressed from the zygotic genome during genome rearrangements. These elements might be derived from *piggyBac* transposons, which have TTAA target sites, and perhaps testify to the ancient *piggyBac* invasion that led to domestication of the transposase.

A contrasting situation is found in some stichotrich ciliates. The stichotrich ciliates are very distantly related to the oligohymenophorean ciliates and are characterized by highly fragmented somatic genomes consisting of nanochromosomes that usually bear a single gene. Intragenic IESs are more abundant in the germline genomes of *Oxytricha* and related strichotrichs than in *Paramecium*, with an estimate of at least 150,000 IESs per haploid genome [68]. Both single-copy IESs and transposons are precisely excised and the precise IES excision is assured by guide RNAs transcribed from the maternal MAC [69], which are even capable of re-ordering the scrambled MAC-destined gene segments that occur frequently in *Oxytricha* and related stichotrichs [70]. There is also evidence that the endonuclease required for cleavage in *Oxytricha* is actually a transposase from germline TBE transposons [71]. However, there is currently no evidence for a scnRNA pathway specialized in the control of DNA elimination, although gene silencing by RNAi in *Oxytricha* testifies to the presence of small RNA machinery [69]. Thus the high precision and fidelity of the guide RNA mechanism for genome rearrangements in *Oxytricha* spp. seems to have tipped the balance even further in favor of intragenic IES insertions.

The case of *Euplotes*, a stichotrich ciliate distantly related to *Oxytricha* and probably lacking scrambled genes, merits special attention. Beautiful work carried out by the Jahn and Klobutcher labs in the 1990s showed (i) the existence of high copy number Tc1/mariner elements, Tec1 (2,000 copies per haploid genome) and Tec2 (5,000 copies), as well as lower copy number Tec3 elements (20–30 copies) [33], [72], [73]; (ii) at least a fraction of these Tec elements are precisely excised between TA dinucleotides [74]; (iii) an estimated 20,000 short TA-bounded IESs [33], bearing a Tc1/mariner end consensus just like the *Paramecium* IESs [18], are excised precisely between TA dinucleotides leaving a single TA at each excision site on the MAC destined chromosomes [33] and (iv) molecular characterization of excised circular forms of both Tec elements and short IESs revealed an unusual junction consisting of 2 TA dinucleotides separated by 10 bp of partially heteroduplex DNA, showing that both the Tec transposons and the short IESs are excised by the same mechanism [74], [75]. The mechanism is moreover different from that of precise IES excision in *Paramecium* [24], [57]. Neither the endonuclease responsable for IES cleavage nor the repair pathway has currently been identified in *Euplotes*. It will be fascinating to see whether the same actors, i.e. a domesticated *piggyBac* transposase and the NHEJ (non-homologous end-joining) pathway, are responsible for a mechanism that in its details is not the same as that found in *Paramecium*, or whether completely different cellular machinery has been recruited to carry out the same function i.e. the precise excision from somatic DNA of the Tc1/mariner family Tec transposons and of short TA-bounded IESs presumed to be their relics [19].

In conclusion, different ciliates have evolved different host defenses in response to germline SGE insertions. In all cases that have been examined at the molecular level, maternal non-coding RNAs are involved in programming genome rearrangements. In *Paramecium* and some other lineages, the co-evolution of host defense machinery and SGEs has provided mechanisms for precise somatic excision, uniquely allowing the colonization of coding sequences by Tc1/mariner and likely other transposable elements. This phenomenon is so far only paralleled by the spread of introns into eukaryotic coding sequences, also thought to result from domestication of precise excision machinery, derived in this case from mobile self-splicing ribozymes [76].

## Materials and Methods

### Purification of DNA enriched in un-rearranged sequences from isolated nuclei of cells depleted for PiggyMac

#### Cell growth and autogamy

*Paramecium tetraurelia* strain 51 was used for this study because the available phage-lambda library of purified MIC DNA was made using this strain. Strain 51 only differs at a few loci from strain d4-2 that was used for sequencing the MAC genome [77].

For gene silencing, we used the «feeding» method described in [78]. *Escherichia coli* HT115 [79] harboring plasmid L4440 [80], with the 567-bp *Hind*III-*Nco*I fragment of gene *PGM* inserted between two convergent T7 promoters [21], was induced at 37°C for the production of *PGM* dsRNA in WGP1X medium containing 100 µg/mL ampicillin. As a control, we induced HT115 bacteria for the production of dsRNA homologous to the *ND7* non essential gene (see plasmid description in [81]).

*Paramecium tetraurelia* strain 51new mt8 [24] was grown at 27°C in WGP1X inoculated with *Klebsiella pneumoniae* and supplemented with 0.8 µg/mL β-sistosterol. Following ∼25 divisions, cells were washed and transferred to 4.1 L of freshly induced *E. coli* HT115. Cells were allowed to grow for 8 vegetative divisions, then starved to trigger autogamy. The progression of autogamy was monitored by DAPI staining (Figure S2A) and the viability of sexual progeny was tested to evaluate the efficiency of *PGM*-silencing (Figure S2B).

#### Cell lysis and purification of developing MAC DNA

Following prolonged starvation to favor the degradation of old MAC fragments (day 4 of autogamy), all cultures were filtered through eight layers of sterile gauze. Cells were collected by low-speed centrifugation (285× g for 1 min) and washed twice in 10 mM Tris-HCl pH 7.4. Particular care was taken to eliminate contaminating bacterial biofilms by letting them settle to the bottom of the tubes and removing them with a Pasteur pipette prior to all washing centrifugation steps. The final pellet was diluted 5-fold by addition of lysis buffer (0.25 M sucrose, 10 mM MgCl_2_, 10 mM Tris pH 6.8, 0.2% Nonidet P-40) and processed as described in [82]. All steps were performed at 4°C. Briefly, cells (1 mL) were lysed with 100 strokes of a Potter-Elvehjem homogenizer and washing buffer (0.25 M sucrose, 10 mM MgCl_2_, 10 mM Tris pH 7.4) was added to a final volume of 10 mL. Developing new MACs (together with cell debris, bacterial biofilms and the largest fragments of the old MAC) were collected by centrifugation at 600× g for 1 min and washed 3 times in washing buffer. To remove contaminating bacteria, the pellet was diluted in washing buffer, loaded on top of a 3-mL sucrose layer (2.1 M sucrose, 10 mM MgCl_2_, 10 mM Tris pH 7.4) and centrifuged in a swinging rotor for 1 hr at 210,000× g. The nuclear pellet was collected and diluted 5-fold in 10 mM MgCl_2_ 10 mM Tris pH 7.4 prior to addition of two volumes of proteinase K buffer (0.5 M EDTA pH 9, 1% N-lauryl sarcosine sodium, 1% SDS, 1 mg/mL proteinase K). Following 16-hr incubation at 55°C, genomic DNA was purified as described in [24], with three additional phenol:CHCl_3_ extractions (1∶1), one CHCl_3_ extraction and a final ethanol precipitation [83]. Enrichment for non-excised IESs (IES^+^ forms) was assayed by 1% agarose gel electrophoresis of *Pst*I-restricted DNA and Southern blot hybridization with ^32^P-labeled Gmac probe [21], which corresponds to the MAC sequences just downstream of IES 51G4404 within the surface antigen *G^51^* gene (Figure S3A). To measure the contamination with bacterial DNA, the same blot was dehybridized and probed with a ^32^P-labelled fragment of *K. pneumoniae* 23S rDNA amplified by PCR using primers KP23S-U (5′-AGCGTTCTGTAAGCCTGCGAAGGTG-3′) and KP23S-R (5′-TTCACCTACACACCAGCGTGCCTTC-3′) (Figure S3B). All radioactive signals were scanned and quantified using a Typhoon phosphorimager (Figure S3C).

### Purification of wild-type micronuclear DNA from a lambda-phage library

A lambda-phage library was provided by John Preer. This library had been made from DNA obtained after isolation of stock 51 wild type micronuclei [82] and further purified by cesium chloride density gradient centrifugation to eliminate G+C-rich DNA supposed to represent bacterial contaminants [28]. The library consisted of 70,000 recombinant phages (lambdaGEM11), expected to represent a 7× coverage of the MIC genome. We amplified the original library in 1995 and stored it at 4°C. Phage particles from 1 mL of the reamplified library (approximately 10^5^ particles) were fully recovered by ultracentrifugation (42 min at 113898 g in a TLA-55 rotor; S_lambda particle_ = 410 according to [84]) and concentrated in ∼30 µL. Given the limited amount of material (∼18 pg of 40 Kb phage genomes corresponding to ∼4.5 pg of inserts), the cloned DNA was amplified by PCR using primers located next to the cloning sites (LambdaL2 GGCCTAATACGACTCACTATAGG; LambdaR2 GCCATTTAGGTGACACTATAGAAGAG). Non-genomic sequences should only represent 0.6% of the total PCR-amplified DNA. As PCR inhibitors prevented direct amplification from the concentrated suspension of phage particles, 230 50 µL-PCR reactions were performed from 3 µL of a 30× dilution in SM. The Expand Long-Template PCR System (Roche) was used as recommended by the supplier with 23 amplification cycles, an annealing temperature of 60°C and 12 min for the extension time. PCR reactions were concentrated by ethanol precipitation and ∼35 µg of 9 to 13 Kb PCR products were obtained after purification from 0.6% low-melting-temperature agarose gels and treatment with β-agarase (Sigma).

### DNA sequencing

DNA was sequenced by a paired-end strategy using Illumina GAII and HiSeq next-generation sequencers. The shotgun fragments were ∼500 bp and the paired-end reads 108 nt for DNA enriched in un-rearranged sequences (PGM DNA). The fragments were ∼200 bp and the paired-end reads 101 nt for DNA prepared from the lambda-phage library. In the latter case, short reads that overlapped were merged.

### Short read mapping

All Illumina short reads were mapped to the strain 51 reference genome (see below) using BWA [85] (version 0.5.8). Alignments were indexed using samtools [86] (version 0.1.11).

### Strain 51 reference genome

The *P. tetraurelia* MAC genome [1] was assembled from 13× Sanger sequencing reads from different insert size librairies of strain d4-2 DNA. Strain d4-2 only differs from strain 51 at a few loci. We corrected sequencing errors in the scaffolds using Illumina deep sequencing in two stages, the first stage using the same strain d4-2 DNA sample that had been used for the original Sanger sequencing (84 million 75 nt paired-end reads), the second stage using two different samples of strain 51 MAC DNA (155 million 75 nt paired-end reads). The electronic polishing pipeline used for each stage consisted of the following steps. (i) Gap filling was achieved by assembling the Illumina reads into contigs using the Velvet [87] short read assembler (Kmer = 55 -ins_length 400 -cov_cutoff 3 -scaffolding no). The contigs were mapped to the draft assembly using BLAT [88] and locally realigned with Muscle [89]. If the contigs spanned a sequencing gap, then it was filled. (ii) The Illumina reads were mapped to the draft genome using BWA [85]. (iii) Alignments were indexed using samtools [86]. (iv) Samtools mpileup program and homemade Perl scripts were used to identify all positions covered by at least 10 reads and where at least 80% of the reads did not confirm the reference sequence. (v) The reference sequence was corrected using the list of errors. Steps (ii) through (v) were repeated a few times at the second stage of correction using the strain 51 reads, since BWA mapping has low error tolerance, and more reads could be mapped as the correction progressed. At the end of the process 442 of 861 sequencing gaps were filled, 13,758 substitutions were corrected, 929 deletions of 1–2 nt were filled and 10,339 insertions of 1–2 nt were removed, to yield the strain 51 reference genome that was used for IES identification. The strain 51 reference genome is available via ParameciumDB [90].

### IES identification pipeline

#### MIRAA pipeline

All reads of the DNA enriched in un-rearranged sequences (PGM DNA), were mapped on the *P. tetraurelia* strain 51 reference MAC genome. Alignments indexed with samtools were analysed using custom perl scripts written with the BioPerl library (version 1.6) and the Bio::DB::Sam module (version 1.11). An IES site is characterized by an excess of ends of read alignments since reads that overlap IES junctions only map partially on the MAC genome and stop on the residual TA. These positions are considered to be IES sites if (i) the number of alignment ends is greater than 15 (10% of the average PGM DNA read coverage); (ii) if they are more than 500 bp from the ends of a scaffold, which avoids errors produced by heterogeneity in these regions; (iii) if the read coverage is lower than 300×, to avoid highly repeated sequences.

#### MICA pipeline

The IES detection pipeline consists of the following steps: (i) paired-end read assembly with Velvet [87] (version 1.0.18) using 3 different Kmer values (41, 45 and 55) and the parameters “-scaffolding no -max_coverage 500 -exp_cov auto -ins_length 500 -min_contig_length 100”; (ii) Only contigs with average G+C content less than 0.5 are retained; (iii) repeats are masked with RepeatMasker; (iv) masked contigs are aligned on the reference MAC genome with BLAT (version 34); (v) gaps are realigned locally with Muscle (version 3.7) and a custom Perl script is used to adjust the ends of the alignment. If the alignment is bound by TA dinucleotides, the insert in the contig is considered to be an IES. This pipeline was used to find IESs in the following sets of reads:

1. All the PGM reads after removal of known contaminants (bacteria, rDNA, mitochondrial DNA).
2. All pairs of reads in which at least one read does not align with the MAC reference genome, in order to enrich in MIC reads.
3. All PGM reads after removal of reads that correspond to the potential MAC IES junctions identified by the MIRAA pipeline.
4. Finally, all PGM reads after removal of reads that correspond to a MAC junction identified by the MICA pipeline using the above data sets.

The IES identification pipelines, datasets and overlap between IESs and potential IES sites are summarized in Figure S1. The statistics for each of the assemblies are provided in Table S1.

### IES conservation

Determination of IESs that are conserved in genes duplicated by a WGD event involved identification of the position of the IES with respect to the beginning of the alignment, either using a protein alignment of ohnologs, back translated into nucleotide sequence, or using nucleotide alignment of the 2 genes. In both cases, the alignments were carried out using Muscle [89] (version 3.7). If the relative positions of the IES is the same within a 2 nt tolerance, then the IESs are considered to be conserved.

### Measurement of protein divergence

A phylogenetic tree was computed by concatenation of the alignment of 1350 protein families corresponding to quartets of ohnologs preserved after both the intermediate and recent WGD events. All gap-containing sites were excluded from the alignment, which is therefore robust with respect to possible annotation errors. The tree was constructed using BioNJ [91] with Poisson correction for multiple substitutions. The average length of the 2 branches between the intermediate and recent WGDs is 0.085 substitutions/site. The average length of the 4 branches between the recent WGD and the present is 0.0825 substitutions per site. Assuming a constant substitution rate, we can infer that the time between the intermediate and recent WGD events and between the recent WGD and the present are equivalent, although we cannot date the events since we do not know the substitution rate in *Paramecium*.

### Availability of data

The MAC reference genome used for this study (strain 51) and the genome-wide set of IESs are available at http://paramecium.cgm.cnrs-gif.fr/download/. The IESs have also been integrated into ParameciumDB BioMart complex query interface and the ParameciumDB Genome Browser [90]. The short read datasets have been deposited at the European Nucleotide Archive (Accession numbers ERA137444 and ERA137420).

### Validation by PCR of individual IES or IES insertion sites

Oligonucleotides were designed to flank the IES insertion site at a distance of 150–200 nt to allow detection of amplification products with or without an IES. All PCR amplifications were performed with an Eppendorf personal mastercycler. Standard PCR amplifications were performed with 1 unit of DyNazyme II with reagent concentrations according to instructions provided by Finnzyme (dNTP: 200 µM each, primers: 0.5 µM each) with 50 ng of template DNA. The program used is 2 min at 95°C, 10 cycles of 45 sec at 95°C, 45 sec at annealing temperature, and 1 min at 72°C, 15 cycles of 20 sec at 95°C, 20 sec at annealing temperature and 1 min at 72°C, followed by a final incubation for 3 min at 72°C. Amplified products were analyzed on 3% Nusieve (Lonza) in TBE 1×. Long and AT-rich PCR amplifications were performed with 1 unit of Phusion (Finnzymes) using the following concentrations of reagents (dATP and dTTP: 400 µM each, dCTP and dGTP: 200 µM each, primers: 0.5 µM each) with 50 ng of template DNA. The program used was 1 min at 98°C, 25 cycles of 10 sec at 98°C, 30 sec at annealing temperature and 5 min at 72°C, followed by a final incubation of 2 min at 72°C. Amplified products were analyzed on 1% UltraPure agarose (Invitrogen) in TAE 1×. The template DNA for the amplification reactions was an aliquot of PGM DNA enriched in un-rearranged sequences, prepared as described above.

### Transposon identification

Isolation of inserts from the MIC lambda-phage library [28] was carried out as previously described [31]. Phage inserts and long-range PCR products obtained by amplification of total DNA from vegetative cells were isolated and subjected to Sanger sequencing as in [34]. The lambda-phage inserts and the cloned long-range PCR products used to characterize the *Sardine* and *Thon* transposons have been deposited in the EMBL Nucleotide Sequence Database with accession numbers HE774468–HE774475.

Several IESs with homology to the PFAM DDE_3 domain were used to find other IESs sharing nucleotide identity, leading to a set of 28 IESs that were aligned with Muscle [89] to identify 2 *Anchois* transposons. In a second step, the alignment was refined and manually adjusted in order to reconstruct the *AnchoisA* and *AnchoisB* transposons. These second step alignments were built using IESs along with some PGM contigs that correspond to germline-restricted, imprecisely eliminated regions of the genome containing *Anchois* copies (Text S1).

### Data analysis

Statistical analyses and graphics were performed in the R environment for statistical computing [92] using standard packages, as well as the ape package [93] for phylogenetic analysis. Sequence logos were generated using weblogo software [94].

## Supporting Information

Figure S1 IES identification. A. Schematic representation of the MIRAA pipeline for identification of IES sites by read mapping. B. Schematic representation of the MICA pipeline for identification of IESs by comparison of contigs with the reference genome assembly. C. PGM DNA datasets which were used with the MICA pipeline to identify the genome-wide set of IESs. As explained in Materials and Methods, the 4 datasets are (i) all PGM reads after filtering known contaminants, (ii) all filtered reads with at least one member of the pair that does not match the MAC reference genome, (iii) all filtered reads after removal of the read pairs with a perfect match to a MAC IES juction identified with the MIRAA pipeline and (iv) all filtered reads after removal of the read pairs with a perfect match to a MAC IES junction identified with MICA and the first 3 datasets. D.Venn diagram showing that 96% (n = 43,220) of the IESs identified with MICA correspond to IES insertion sites identified by MIRAA. The MICA pipeline was also used to identify IESs in the phage-lambda inserts: the sequence reads were assembled into 3 sets of contigs with Velvet, using 3 different kmer values (kmer = 45, 51 or 55).
(PDF)

Figure S2 Autogamy time-course of *P. tetraurelia* 51 mt8 submitted to RNAi against *PiggyMac*. A. Cells were transferred at day 0 into 4.1 L of freshly induced feeding bacteria producing dsRNA homologous to a 567-bp region of the *PGM* gene and incubated at 27°C. The progression of autogamy was monitored everyday (D1: day 1, D2: day 2, D3: day 3, D4: day 4) by DAPI staining of cells. V: vegetative cells, F: cells with fragmented old MAC and no clearly visible new developing MACs, A: cells harboring two developing new MACs, C: post-autogamous cells with one new MAC surrounded with fragments of the old MAC. B. Survival of post-autogamous progeny. At day 4, 30 autogamous cells were transferred individually to standard growth medium containing *K. pneumoniae* and incubated at 27°C to follow the resumption of vegetative growth. Survival of the progeny of autogamous cells obtained in standard (Kp) or in control RNAi medium (ND7) was also tested. Wt: normally-growing progeny, sick: slowly-growing cells, often with abnormal swimming behavior.
(PDF)

Figure S3 Purification of IES-enriched genomic DNA from PGM-silenced cells. Autogamous cells were collected at day 4 and genomic DNA was extracted through several cell fractionation steps. Lys.1 and lys.2: independent samples of cells were lysed directly in proteinase K buffer; low sp.: DNA extracted from low speed pellets (600× g for 1 min followed by washing); suc.: DNA extracted from nuclear pellets obtained following centrifugation through a 2.1 M sucrose layer. Each DNA sample was digested by PstI and the digestion fragments were separated on a 1% agarose gel. A. Southern blot hybridization with the Gmac probe (shown as a grey box on the diagram). The position of size markers is shown on the left. IES^−^ and IES^+^ bands were quantified separately. B. Southern blot hybridization with the *K. pneumoniae* 23S rDNA probe. Size markers are shown on the right. All rDNA bands were quantified together. C. Quantification of radioactive signals from the blots shown in A and B. The fraction of IES^+^ form was normalized relative to the sum of IES^−^ and IES^+^ signals (black histograms). Bacterial rDNA was normalized relative to the sum of IES^−^ and IES^+^ signals (grey histograms).
(PDF)

Figure S4 IES distribution on the 8 largest MAC chromosomes. The 8 largest, telomere-capped scaffolds (∼750 Kb to ∼980 Kb in size) were normalized to length 1.0 and some were flipped so that the highest IES density is to the right. The curves represent histograms of IES position on each scaffold after Gaussian smoothing using the R “density” function [92]. IES distribution was evaluated using a Kolmogorov-Smirnov test of the null hypothesis that IESs are uniformly distributed on the scaffold. For the 8 largest scaffolds, the null hypothesis was strongly rejected (p<10^−8^). The same statistical test was carried out for gene distribution on these chromosomes, and the null hypothesis was not rejected, consistent with a uniform distribution of genes on the chromosomes.
(PDF)

Figure S5 *Sardine* and *Thon* Tc1/mariner family transposons. From top to bottom: 1) *Sardine* transposon consensus sequence obtained by alignment of the lambda-phage and PCR copies (the latter were amplified from total DNA of vegetative cells using primers located within the *Sardine* TIRs), showing the presence of palindromic TIRs and 4 putative ORFs, including a DDE transposase and a tyrosine recombinase; 2) lambda-phage with the 51G flank that led to discovery of the *Sardine* element (the region of *de novo* telomere addition at the end of the MAC chromosome, following developmental breakage of the MIC chromosome, is indicated); 3) lambda-phage with the S5 copy of *Sardine*; 4) lambda-phage with the S6 copy of *Sardine*, containing an insertion of a different Tc1/mariner transposon, *Thon*, which has the same general organization as the *Sardine* element; 5) lambda-phage with the S7 copy of *Sardine*; 6) lambda-phage with the S8 copy; 7) PCR products (S46 and S103 copies) with nearly intact ORFs; 8) PCR products (S14 and S106 copies) with nearly intact ORFs. The sequences of the 5 lambda-phages and 4 PCR products have been deposited in the EMBL/GenBANK/DDBJ public nucleotide database with EMBL-Bank accession numbers HE774468–HE774475.
(PDF)

Figure S6 IES size distribution. The histograms represent A) IESs inserted in coding sequences. B) IESs inserted in non-coding sequences. IESs larger than 150 nt are not displayed. The fact that very similar periodic distributions are observed for IESs in both coding and intergenic regions is consistent with the hypothesis that the periodic size constraint is related to the IES excision mechanism. Indeed, IES retention in the MAC could be deleterious either by affecting ORFs (IESs in protein coding sequences) or by affecting regulatory signals (IESs in non-coding sequences).
(PDF)

Figure S7 IES evolution evaluated with quartet IES groups. A) schematic representation of the observable quartet IES groups, arranged from top to bottom according to the number of IESs that are conserved and from left to right, according to the most recent period in which the ancestral IES could have been acquired. B) Schematic representation of the parameters of a statistical model developed to test hypotheses about IES evolution (cf. Text S1). The three time periods delimited by the 2 WGD events and the present time are designated, from the oldest to the most recent, *g_3_*, *g_2_* and *g_1_*. The parameters ρ_3_, ρ_2_ and ρ_1_ are the fraction of IESs that were acquired in each of these time period and the parameters of the form δ_a,b_ are the survival rates for an IES acquired in period *g_a_* during the period *g_b_*. The equations of the model express the observable IES counts as a function of these parameters.
(PDF)

Table S1 Assembly statistics.
(PDF)

Table S2 Molecular validation of some predicted IESs and IES insertion sites.
(PDF)

Table S3 Validation of the genome-wide set of IESs using previously characterized IESs.
(PDF)

Table S4 IESs with homology to *Anchois* transposons.
(PDF)

Table S5 Deficit of 3n IESs in coding sequences, for each peak of the 10 bp periodic size distribution.
(PDF)

Text S1 Transposon sequences. A). The sequences of *Sardine*, *Thon* and *Anchois* transposons reconstituted from manually adjusted multiple alignments of the different decayed copies, cloned from the lambda phage library of MIC DNA (*Sardine*, *Thon*) or found in the PGM DNA assembly (*Anchois*). The sequences of the *Thon* transposon are those of the only known copy, so that ORF annotation (based on homology with the *Sardine* element) is preliminary; the *Thon* ORF1 sequence apparently contains a frameshift. Predicted introns have been removed from the ORF sequences. B) Annotated comparison of *AnchoisA* and *AnchoisB*, showing the position and orientation of the ORFs, with a potential intron in the DDE transposase ORF. C). Manually adjusted alignment used to reconstitute the AnchoisA copy. See Text S4 for the IESs used in the reconstitution. D). Manually adjusted alignment used to reconstitute the *AnchoisB* copy. See Text S4 for the IESs used in the reconstitution. E) IESs used to obtain the final *AnchoisA* and *AnchoisB* consensus sequences based on the manually adjusted alignments in C) and D).
(PDF)

Text S2 Alignment of homologous IESs inserted at non-homologous genomic sites. The IESs of each cluster of homologous IESs (cf. Table 3 and its legend) and 200 bp of 3′ and 5′ flanking sequences were aligned using Muscle [89]. The IESs are in uppercase type and the flanking sequences are in lowercase type. For cluster5, consisting of IESs homologous to a solo TIR of the *Thon* transposon, the consensus sequence and the *Thon* TIR are included in the alignment and the palindromic repeats are highlighted.
(PDF)

Text S3 PCR approach to validate IESs with homology to *Thon* solo TIRs.
(PDF)

Text S4 A maximum likelihood framework for testing hypotheses about IES evolution.
(PDF)

## References

[1] Aury J-M, Jaillon O, Duret L, Noel B, Jubin C, et al (2006) Global trends of whole-genome duplications revealed by the ciliate Paramecium tetraurelia. Nature 444: 171–178 doi:nature05230 17086204 10.1038/nature05230

[2] Chalker DL, Yao M-C (2011) DNA elimination in ciliates: transposon domestication and genome surveillance. Annu Rev Genet 45: 227–246 doi:10.1146/annurev-genet-110410-132432. 21910632 10.1146/annurev-genet-110410-132432

[3] Coyne RS, Lhuillier-Akakpo M, Duharcourt S (2012) RNA-guided DNA rearrangements in ciliates: is the best genome defense a good offense? Biol Cell Accepted manuscript online doi:10.1111/boc.201100057. 10.1111/boc.201100057 22352444

[4] Schoeberl UE, Mochizuki K (2011) Keeping the soma free of transposons: programmed DNA elimination in ciliates. J Biol Chem 286: 37045–37052 doi:10.1074/jbc.R111.276964. 21914793 10.1074/jbc.R111.276964 PMC3199450

[5] Bétermier M (2004) Large-scale genome remodelling by the developmentally programmed elimination of germ line sequences in the ciliate Paramecium. Res Microbiol 155: 399–408. 15207872 10.1016/j.resmic.2004.01.017

[6] Ruiz F, Krzywicka A, Klotz C, Keller A, Cohen J, et al (2000) The SM19 gene, required for duplication of basal bodies in Paramecium, encodes a novel tubulin, eta-tubulin. Curr Biol 10: 1451–1454. 11102808 10.1016/s0960-9822(00)00804-6

[7] Haynes WJ, Ling KY, Preston RR, Saimi Y, Kung C (2000) The cloning and molecular analysis of pawn-B in Paramecium tetraurelia. Genetics 155: 1105–1117. 10880473 10.1093/genetics/155.3.1105 PMC1461171

[8] Mayer KM, Mikami K, Forney JD (1998) A mutation in Paramecium tetraurelia reveals functional and structural features of developmentally excised DNA elements. Genetics 148: 139–149. 9475728 10.1093/genetics/148.1.139 PMC1459799

[9] Mayer KM, Forney JD (1999) A mutation in the flanking 5′-TA-3′ dinucleotide prevents excision of an internal eliminated sequence from the Paramecium tetraurelia genome. Genetics 151: 597–604. 9927454 10.1093/genetics/151.2.597 PMC1460486

[10] Matsuda A, Forney JD (2005) Analysis of Paramecium tetraurelia A-51 surface antigen gene mutants reveals positive-feedback mechanisms for maintenance of expression and temperature-induced activation. Eukaryotic Cell 4: 1613–1619 doi:10.1128/EC.4.10.1613-1619.2005. 16215168 10.1128/EC.4.10.1613-1619.2005 PMC1265893

[11] Yao MC, Choi J, Yokoyama S, Austerberry CF, Yao CH (1984) DNA elimination in Tetrahymena: a developmental process involving extensive breakage and rejoining of DNA at defined sites. Cell 36: 433–440. 6319023 10.1016/0092-8674(84)90236-8

[12] Saveliev SV, Cox MM (2001) Product analysis illuminates the final steps of IES deletion in Tetrahymena thermophila. EMBO J 20: 3251–3261 doi:10.1093/emboj/20.12.3251. 11406601 10.1093/emboj/20.12.3251 PMC150193

[13] Fillingham JS, Thing TA, Vythilingum N, Keuroghlian A, Bruno D, et al (2004) A non-long terminal repeat retrotransposon family is restricted to the germ line micronucleus of the ciliated protozoan Tetrahymena thermophila. Eukaryotic Cell 3: 157–169. 14871946 10.1128/EC.3.1.157-169.2004 PMC329501

[14] Wuitschick JD, Gershan JA, Lochowicz AJ, Li S, Karrer KM (2002) A novel family of mobile genetic elements is limited to the germline genome in Tetrahymena thermophila. Nucleic Acids Res 30: 2524–2537. 12034842 10.1093/nar/30.11.2524 PMC117186

[15] Eisen JA, Coyne RS, Wu M, Wu D, Thiagarajan M, et al (2006) Macronuclear genome sequence of the ciliate Tetrahymena thermophila, a model eukaryote. PLoS Biol 4: e286 doi:10.1371/journal.pbio.0040286. 16933976 10.1371/journal.pbio.0040286 PMC1557398

[16] Yao M-C, Chao J-L (2005) RNA-guided DNA deletion in Tetrahymena: an RNAi-based mechanism for programmed genome rearrangements. Annu Rev Genet 39: 537–559 doi:10.1146/annurev.genet.39.073003.095906. 16285871 10.1146/annurev.genet.39.073003.095906

[17] Fass JN, Joshi NA, Couvillion MT, Bowen J, Gorovsky MA, et al (2011) Genome-Scale Analysis of Programmed DNA Elimination Sites in Tetrahymena thermophila. G3 1: 515–522 doi:10.1534/g3.111.000927. 22384362 10.1534/g3.111.000927 PMC3276166

[18] Klobutcher LA, Herrick G (1995) Consensus inverted terminal repeat sequence of Paramecium IESs: resemblance to termini of Tc1-related and Euplotes Tec transposons. Nucleic Acids Res 23: 2006–2013. 7596830 10.1093/nar/23.11.2006 PMC306976

[19] Klobutcher LA, Herrick G (1997) Developmental genome reorganization in ciliated protozoa: the transposon link. Prog Nucleic Acid Res Mol Biol 56: 1–62. 9187050 10.1016/s0079-6603(08)61001-6

[20] Plasterk RH, Izsvák Z, Ivics Z (1999) Resident aliens: the Tc1/mariner superfamily of transposable elements. Trends Genet 15: 326–332. 10431195 10.1016/s0168-9525(99)01777-1

[21] Baudry C, Malinsky S, Restituito M, Kapusta A, Rosa S, et al (2009) PiggyMac, a domesticated piggyBac transposase involved in programmed genome rearrangements in the ciliate Paramecium tetraurelia. Genes Dev 23: 2478–2483 doi:10.1101/gad.547309. 19884254 10.1101/gad.547309 PMC2779751

[22] Cheng C-Y, Vogt A, Mochizuki K, Yao M-C (2010) A domesticated piggyBac transposase plays key roles in heterochromatin dynamics and DNA cleavage during programmed DNA deletion in Tetrahymena thermophila. Mol Biol Cell 21: 1753–1762 doi:10.1091/mbc.E09-12-1079. 20357003 10.1091/mbc.E09-12-1079 PMC2869380

[23] Parfrey LW, Lahr DJG, Knoll AH, Katz LA (2011) Estimating the timing of early eukaryotic diversification with multigene molecular clocks. Proc Natl Acad Sci USA 108: 13624–13629 doi:10.1073/pnas.1110633108. 21810989 10.1073/pnas.1110633108 PMC3158185

[24] Gratias A, Bétermier M (2003) Processing of double-strand breaks is involved in the precise excision of paramecium internal eliminated sequences. Mol Cell Biol 23: 7152–7162. 14517286 10.1128/MCB.23.20.7152-7162.2003 PMC230320

[25] Mitra R, Fain-Thornton J, Craig NL (2008) piggyBac can bypass DNA synthesis during cut and paste transposition. EMBO J 27: 1097–1109 doi:10.1038/emboj.2008.41. 18354502 10.1038/emboj.2008.41 PMC2323262

[26] Kapusta A, Matsuda A, Marmignon A, Ku M, Silve A, et al (2011) Highly precise and developmentally programmed genome assembly in Paramecium requires ligase IV-dependent end joining. PLoS Genet 7: e1002049 doi:10.1371/journal.pgen.1002049. 21533177 10.1371/journal.pgen.1002049 PMC3077386

[27] Preer LB, Hamilton G, Preer JR Jr (1992) Micronuclear DNA from Paramecium tetraurelia: serotype 51 A gene has internally eliminated sequences. J Protozool 39: 678–682. 1453356 10.1111/j.1550-7408.1992.tb04448.x

[28] Steele CJ, Barkocy-Gallagher GA, Preer LB, Preer JR Jr (1994) Developmentally excised sequences in micronuclear DNA of Paramecium. Proc Natl Acad Sci USA 91: 2255–2259. 8134383 10.1073/pnas.91.6.2255 PMC43349

[29] Duret L, Cohen J, Jubin C, Dessen P, Goût J-F, et al (2008) Analysis of sequence variability in the macronuclear DNA of Paramecium tetraurelia: a somatic view of the germline. Genome Res 18: 585–596 doi:gr.074534.107. 18256234 10.1101/gr.074534.107 PMC2279246

[30] Arnaiz O, Sperling L (2010) ParameciumDB in 2011: new tools and new data for functional and comparative genomics of the model ciliate Paramecium tetraurelia. Nucleic Acids Res Available:http://www.ncbi.nlm.nih.gov.gate1.inist.fr/pubmed/20952411. Accessed 14 December 2010. 10.1093/nar/gkq918 PMC3013783 20952411

[31] Duharcourt S, Keller AM, Meyer E (1998) Homology-dependent maternal inhibition of developmental excision of internal eliminated sequences in Paramecium tetraurelia. Mol Cell Biol 18: 7075–7085. 9819394 10.1128/mcb.18.12.7075 PMC109289

[32] Doak TG, Doerder FP, Jahn CL, Herrick G (1994) A proposed superfamily of transposase genes: transposon-like elements in ciliated protozoa and a common “D35E” motif. Proc Natl Acad Sci USA 91: 942–946. 8302872 10.1073/pnas.91.3.942 PMC521429

[33] Jacobs ME, Sánchez-Blanco A, Katz LA, Klobutcher LA (2003) Tec3, a new developmentally eliminated DNA element in Euplotes crassus. Eukaryotic Cell 2: 103–114. 12582127 10.1128/EC.2.1.103-114.2003 PMC141165

[34] Le Mouël A, Butler A, Caron F, Meyer E (2003) Developmentally regulated chromosome fragmentation linked to imprecise elimination of repeated sequences in paramecia. Eukaryotic Cell 2: 1076–1090. 14555491 10.1128/EC.2.5.1076-1090.2003 PMC219357

[35] Jaillon O, Bouhouche K, Gout J-F, Aury J-M, Noel B, et al (2008) Translational control of intron splicing in eukaryotes. Nature 451: 359–362 doi:nature06495. 18202663 10.1038/nature06495

[36] DuBois ML, Prescott DM (1997) Volatility of internal eliminated segments in germ line genes of hypotrichous ciliates. Mol Cell Biol 17: 326–337. 8972213 10.1128/mcb.17.1.326 PMC231757

[37] Dubrana K, Le Mouël A, Amar L (1997) Deletion endpoint allele-specificity in the developmentally regulated elimination of an internal sequence (IES) in Paramecium. Nucleic Acids Res 25: 2448–2454. 9171098 10.1093/nar/25.12.2448 PMC146731

[38] Gout J-F, Kahn D, Duret L (2010) The relationship among gene expression, the evolution of gene dosage, and the rate of protein evolution. PLoS Genet 6: e1000944 doi:10.1371/journal.pgen.1000944. 20485561 10.1371/journal.pgen.1000944 PMC2869310

[39] Arnaiz O, Gout J-F, Betermier M, Bouhouche K, Cohen J, et al (2010) Gene expression in a paleopolyploid: a transcriptome resource for the ciliate Paramecium tetraurelia. BMC Genomics 11: 547 doi:10.1186/1471-2164-11-547. 20932287 10.1186/1471-2164-11-547 PMC3091696

[40] Duharcourt S, Butler A, Meyer E (1995) Epigenetic self-regulation of developmental excision of an internal eliminated sequence on Paramecium tetraurelia. Genes Dev 9: 2065–2077. 7649484 10.1101/gad.9.16.2065

[41] Meyer E, Keller AM (1996) A Mendelian mutation affecting mating-type determination also affects developmental genomic rearrangements in Paramecium tetraurelia. Genetics 143: 191–202. 8722774 10.1093/genetics/143.1.191 PMC1207253

[42] Duharcourt S, Lepère G, Meyer E (2009) Developmental genome rearrangements in ciliates: a natural genomic subtraction mediated by non-coding transcripts. Trends Genet 25: 344–350 doi:10.1016/j.tig.2009.05.007. 19596481 10.1016/j.tig.2009.05.007

[43] Lepère G, Nowacki M, Serrano V, Gout J-F, Guglielmi G, et al (2009) Silencing-associated and meiosis-specific small RNA pathways in Paramecium tetraurelia. Nucleic Acids Res 37: 903–915 doi:10.1093/nar/gkn1018. 19103667 10.1093/nar/gkn1018 PMC2647294

[44] Lepère G, Bétermier M, Meyer E, Duharcourt S (2008) Maternal noncoding transcripts antagonize the targeting of DNA elimination by scanRNAs in Paramecium tetraurelia. Genes Dev 22: 1501–1512 doi:10.1101/gad.473008. 18519642 10.1101/gad.473008 PMC2418586

[45] Nowacki M, Zagorski-Ostoja W, Meyer E (2005) Nowa1p and Nowa2p: novel putative RNA binding proteins involved in trans-nuclear crosstalk in Paramecium tetraurelia. Curr Biol 15: 1616–1628 doi:10.1016/j.cub.2005.07.033. 16169483 10.1016/j.cub.2005.07.033

[46] Epstein LM, Forney JD (1984) Mendelian and non-mendelian mutations affecting surface antigen expression in Paramecium tetraurelia. Mol Cell Biol 4: 1583–1590. 6092921 10.1128/mcb.4.8.1583-1590.1984 PMC368951

[47] Meyer E (1992) Induction of specific macronuclear developmental mutations by microinjection of a cloned telomeric gene in Paramecium primaurelia. Genes Dev 6: 211–222. 1737617 10.1101/gad.6.2.211

[48] Sperling L (2011) Remembrance of things past retrieved from the Paramecium genome. Res Microbiol 162: 587–597 doi:10.1016/j.resmic.2011.02.012. 21392574 10.1016/j.resmic.2011.02.012

[49] Schleif R (1992) DNA Looping. Annual Review of Biochemistry 61: 199–223 doi:10.1146/annurev.bi.61.070192.001215. 10.1146/annurev.bi.61.070192.001215 1497310

[50] Lane D, Cavaillé J, Chandler M (1994) Induction of the SOS response by IS1 transposase. J Mol Biol 242: 339–350 doi:10.1006/jmbi.1994.1585. 7932694 10.1006/jmbi.1994.1585

[51] Goryshin IYu, Kil YV, Reznikoff WS (1994) DNA length, bending, and twisting constraints on IS50 transposition. Proc Natl Acad Sci USA 91: 10834–10838. 7971970 10.1073/pnas.91.23.10834 PMC45120

[52] Müller J, Oehler S, Müller-Hill B (1996) Repression of lac promoter as a function of distance, phase and quality of an auxiliary lac operator. J Mol Biol 257: 21–29 doi:10.1006/jmbi.1996.0143. 8632456 10.1006/jmbi.1996.0143

[53] Bellomy GR, Mossing MC, Record MT Jr (1988) Physical properties of DNA in vivo as probed by the length dependence of the lac operator looping process. Biochemistry 27: 3900–3906. 3046661 10.1021/bi00411a002

[54] Bond LM, Peters JP, Becker NA, Kahn JD, Maher LJ (2010) Gene repression by minimal lac loops in vivo. Nucleic Acids Research 38: 8072–8082 doi:10.1093/nar/gkq755. 21149272 10.1093/nar/gkq755 PMC3001091

[55] Lee DH, Schleif RF (1989) In vivo DNA loops in araCBAD: size limits and helical repeat. Proceedings of the National Academy of Sciences 86: 476–480. 10.1073/pnas.86.2.476 PMC286493 2643114

[56] Haykinson MJ, Johnson RC (1993) DNA looping and the helical repeat in vitro and in vivo: effect of HU protein and enhancer location on Hin invertasome assembly. EMBO J 12: 2503–2512. 8508775 10.1002/j.1460-2075.1993.tb05905.x PMC413488

[57] Bétermier M, Duharcourt S, Seitz H, Meyer E (2000) Timing of developmentally programmed excision and circularization of Paramecium internal eliminated sequences. Mol Cell Biol 20: 1553–1561. 10669733 10.1128/mcb.20.5.1553-1561.2000 PMC85339

[58] Gratias A, Lepère G, Garnier O, Rosa S, Duharcourt S, et al (2008) Developmentally programmed DNA splicing in Paramecium reveals short-distance crosstalk between DNA cleavage sites. Nucleic Acids Res 36: 3244–3251 doi:10.1093/nar/gkn154. 18420657 10.1093/nar/gkn154 PMC2425466

[59] Chandler M, Mahillon J (2002) Insertion Sequences Revisited. Mobile DNA II. Washington, D.C.: ASM Press. pp. 305–366.

[60] Paull TT, Haykinson MJ, Johnson RC (1993) The nonspecific DNA-binding and -bending proteins HMG1 and HMG2 promote the assembly of complex nucleoprotein structures. Genes Dev 7: 1521–1534. 8339930 10.1101/gad.7.8.1521

[61] Tian Z, Rizzon C, Du J, Zhu L, Bennetzen JL, et al (2009) Do genetic recombination and gene density shape the pattern of DNA elimination in rice long terminal repeat retrotransposons? Genome Res 19: 2221–2230 doi:10.1101/gr.083899.108. 19789376 10.1101/gr.083899.108 PMC2792168

[62] Garfinkel DJ, Nyswaner KM, Stefanisko KM, Chang C, Moore SP (2005) Ty1 copy number dynamics in Saccharomyces. Genetics 169: 1845–1857 doi:10.1534/genetics.104.037317. 15687270 10.1534/genetics.104.037317 PMC1449601

[63] Lynch M (2006) The origins of eukaryotic gene structure. Mol Biol Evol 23: 450–468 doi:10.1093/molbev/msj050. 16280547 10.1093/molbev/msj050

[64] Werren JH (2011) Selfish genetic elements, genetic conflict, and evolutionary innovation. Proc Natl Acad Sci USA 108 Suppl 2: 10863–10870 doi:10.1073/pnas.1102343108. 21690392 10.1073/pnas.1102343108 PMC3131821

[65] Bourc'his D, Voinnet O (2010) A small-RNA perspective on gametogenesis, fertilization, and early zygotic development. Science 330: 617–622 doi:10.1126/science.1194776. 21030645 10.1126/science.1194776

[66] Malone CD, Hannon GJ (2009) Molecular evolution of piRNA and transposon control pathways in Drosophila. Cold Spring Harb Symp Quant Biol 74: 225–234 doi:10.1101/sqb.2009.74.052. 20453205 10.1101/sqb.2009.74.052 PMC3181074

[67] Baulcombe D (2004) RNA silencing in plants. Nature 431: 356–363 doi:10.1038/nature02874. 15372043 10.1038/nature02874

[68] Prescott DM, Prescott JD, Prescott RM (2002) Coding properties of macronuclear DNA molecules in Sterkiella nova (Oxytricha nova). Protist 153: 71–77. 12022278 10.1078/1434-4610-00084

[69] Nowacki M, Vijayan V, Zhou Y, Schotanus K, Doak TG, et al (2008) RNA-mediated epigenetic programming of a genome-rearrangement pathway. Nature 451: 153–158. 18046331 10.1038/nature06452 PMC2647009

[70] Prescott DM (1999) The evolutionary scrambling and developmental unscrambling of germline genes in hypotrichous ciliates. Nucleic Acids Res 27: 1243–1250. 9973610 10.1093/nar/27.5.1243 PMC148308

[71] Nowacki M, Higgins BP, Maquilan GM, Swart EC, Doak TG, et al (2009) A functional role for transposases in a large eukaryotic genome. Science 324: 935–938 doi:10.1126/science.1170023. 19372392 10.1126/science.1170023 PMC3491810

[72] Jahn CL, Klobutcher LA (2002) Genome remodeling in ciliated protozoa. Annu Rev Microbiol 56: 489–520 doi:10.1146/annurev.micro.56.012302.160916. 12142486 10.1146/annurev.micro.56.012302.160916

[73] Jahn CL, Doktor SZ, Frels JS, Jaraczewski JW, Krikau MF (1993) Structures of the Euplotes crassus Tec1 and Tec2 elements: identification of putative transposase coding regions. Gene 133: 71–78. 8224896 10.1016/0378-1119(93)90226-s

[74] Jaraczewski JW, Jahn CL (1993) Elimination of Tec elements involves a novel excision process. Genes Dev 7: 95–105. 8380782 10.1101/gad.7.1.95

[75] Klobutcher LA, Turner LR, LaPlante J (1993) Circular forms of developmentally excised DNA in Euplotes crassus have a heteroduplex junction. Genes Dev 7: 84–94. 8422990 10.1101/gad.7.1.84

[76] Lambowitz AM, Zimmerly S (2011) Group II Introns: Mobile Ribozymes that Invade DNA. Cold Spring Harbor Perspectives in Biology 3 Available:http://cshperspectives.cshlp.org/content/3/8/a003616.abstract 10.1101/cshperspect.a003616 PMC3140690 20463000

[77] Sonneborn TM (1974) Paramecium aurelia. Handbook of Genetics. R. King. New York: Plenum Press, Vol. 11. pp. 469–594.

[78] Galvani A, Sperling L (2002) RNA interference by feeding in Paramecium. Trends Genet 18: 11–12. 11750689 10.1016/s0168-9525(01)02548-3

[79] Timmons L, Court DL, Fire A (2001) Ingestion of bacterially expressed dsRNAs can produce specific and potent genetic interference in Caenorhabditis elegans. Gene 263: 103–112. 11223248 10.1016/s0378-1119(00)00579-5

[80] Timmons L, Fire A (1998) Specific interference by ingested dsRNA. Nature 395: 854 doi:10.1038/27579. 9804418 10.1038/27579

[81] Garnier O, Serrano V, Duharcourt S, Meyer E (2004) RNA-mediated programming of developmental genome rearrangements in Paramecium tetraurelia. Mol Cell Biol 24: 7370–7379 doi:10.1128/MCB.24.17.7370-7379.2004. 15314149 10.1128/MCB.24.17.7370-7379.2004 PMC506981

[82] Preer LB, Hamilton G, Preer JR Jr (1992) Micronuclear DNA from Paramecium tetraurelia: serotype 51 A gene has internally eliminated sequences. J Protozool 39: 678–682. 1453356 10.1111/j.1550-7408.1992.tb04448.x

[83] Sambrook J, Fritsch EF, Maniatis T (1989) Molecular Cloning: A Laboratory Manual. 2nd ed. Cold Spring Harbor Laboratory Pr. 1659 p.

[84] Weigle J (1966) Assembly of phage lambda in vitro. Proc Natl Acad Sci USA 55: 1462–1466. 5227665 10.1073/pnas.55.6.1462 PMC224345

[85] Li H, Durbin R (2009) Fast and accurate short read alignment with Burrows–Wheeler transform. Bioinformatics 25: 1754–1760 doi:10.1093/bioinformatics/btp324. 19451168 10.1093/bioinformatics/btp324 PMC2705234

[86] Li H, Handsaker B, Wysoker A, Fennell T, Ruan J, et al (2009) The Sequence Alignment/Map format and SAMtools. Bioinformatics 25: 2078–2079 doi:10.1093/bioinformatics/btp352. 19505943 10.1093/bioinformatics/btp352 PMC2723002

[87] Zerbino D, Birney E (2008) Velvet: Algorithms for De Novo Short Read Assembly Using De Bruijn Graphs. Genome Res gr.074492.107 doi:10.1101/gr.074492.107. 10.1101/gr.074492.107 PMC2336801 18349386

[88] Kent WJ (2002) BLAT–the BLAST-like alignment tool. Genome Res 12: 656–664 doi:10.1101/gr.229202. 11932250 10.1101/gr.229202 PMC187518

[89] Edgar RC (2004) MUSCLE: multiple sequence alignment with high accuracy and high throughput. Nucleic Acids Res 32: 1792–1797 doi:10.1093/nar/gkh340. 15034147 10.1093/nar/gkh340 PMC390337

[90] Arnaiz O, Sperling L (2011) ParameciumDB in 2011: new tools and new data for functional and comparative genomics of the model ciliate Paramecium tetraurelia. Nucleic Acids Res 39: D632–636 doi:10.1093/nar/gkq918. 20952411 10.1093/nar/gkq918 PMC3013783

[91] Gascuel O (1997) BIONJ: an improved version of the NJ algorithm based on a simple model of sequence data. Mol Biol Evol 14: 685–695. 9254330 10.1093/oxfordjournals.molbev.a025808

[92] R Development Core Team (2011) R: A language and environment for statistical computing. Available:http://www.R-project.org.

[93] Paradis E, Claude J, Strimmer K (2004) APE: Analyses of Phylogenetics and Evolution in R language. Bioinformatics 20: 289–290. 14734327 10.1093/bioinformatics/btg412

[94] Crooks GE, Hon G, Chandonia J-M, Brenner SE (2004) WebLogo: a sequence logo generator. Genome Res 14: 1188–1190 doi:10.1101/gr.849004. 15173120 10.1101/gr.849004 PMC419797

[95] Catania F, Wurmser F, Potekhin AA, Przybos E, Lynch M (2009) Genetic diversity in the Paramecium aurelia species complex. Mol Biol Evol 26: 421–431 doi:10.1093/molbev/msn266. 19023087 10.1093/molbev/msn266 PMC3888249

