# Supplementary material for: The Paramecium Germline Genome Provides a Niche for Intragenic Parasitic DNA: Evolutionary Dynamics of Internal Eliminated Sequences
Source: PLoS Genet. 2012 Oct 4;8(10):e1002984. doi: 10.1371/journal.pgen.1002984 (PMC3464196; doi:10.1371/journal.pgen.1002984)

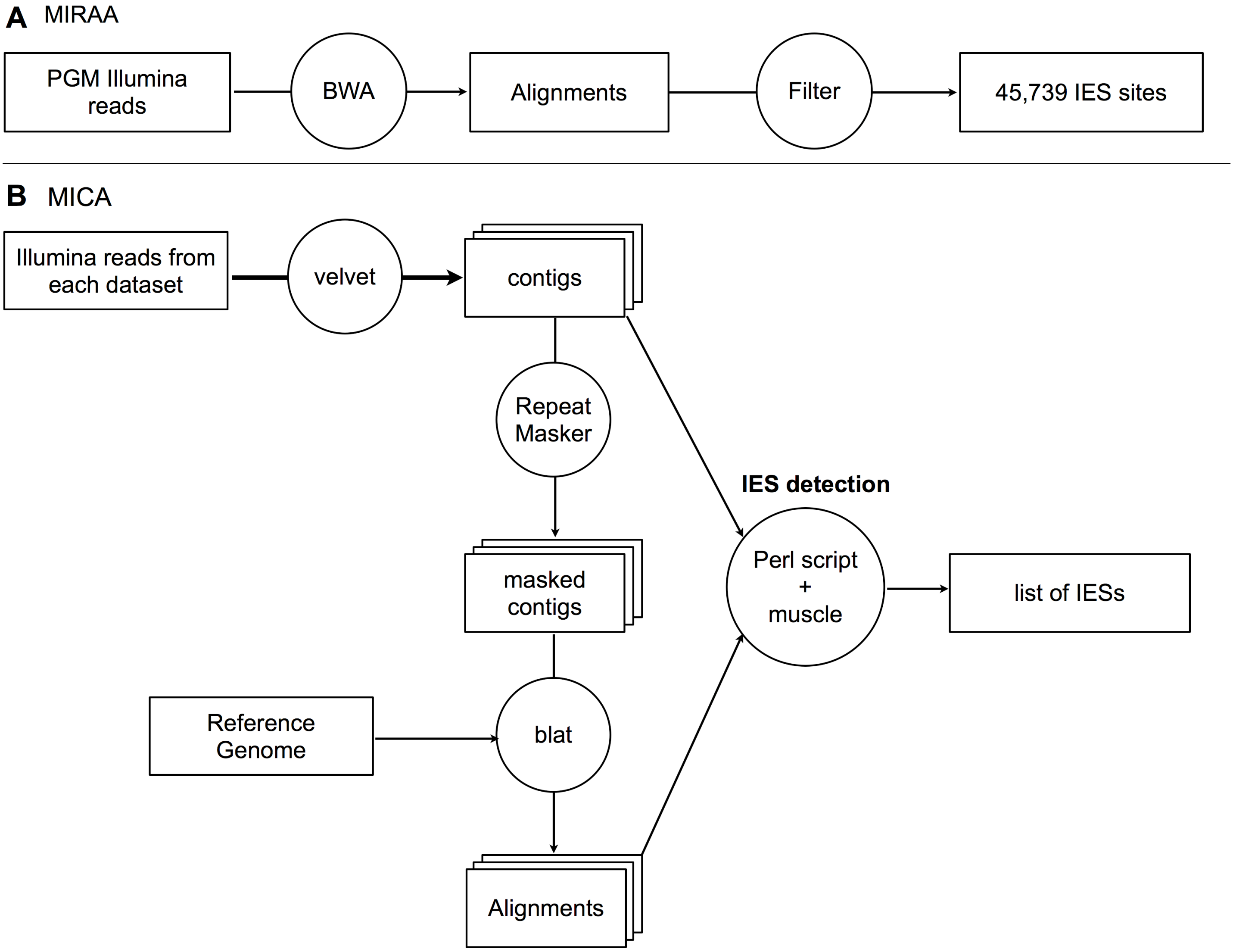

C

Dataset	No. of reads	No. of IESs
All PGM reads	126 M	44,207
Reads at least one no match	32 M	44,211
Reads without MAC junc MIRAA	121 M	44,269
Remove all MAC junc	121 M	43,286
**Merge**	**-**	**44,928**

D

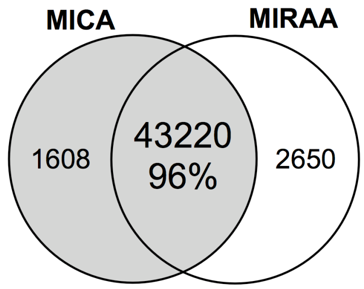

Supplement: Figure S1 — IES identification. A. Schematic representation of the MIRAA pipeline for identification of IES sites by read mapping. B. Schematic representation of the MICA pipeline for identification of IESs by comparison of contigs with the reference genome assembly. C. PGM DNA datasets which were used with the MICA pipeline to identify the genome-wide set of IESs. As explained in Materials and Methods, the 4 datasets are (i) all PGM reads after filtering known contaminants, (ii) all filtered reads with at least one member of the pair that does not match the MAC reference genome, (iii) all filtered reads after removal of the read pairs with a perfect match to a MAC IES juction identified with the MIRAA pipeline and (iv) all filtered reads after removal of the read pairs with a perfect match to a MAC IES junction identified with MICA and the first 3 datasets. D.Venn diagram showing that 96% (n = 43,220) of the IESs identified with MICA correspond to IES insertion sites identified by MIRAA. The MICA pipeline was also used to identify IESs in the phage-lambda inserts: the sequence reads were assembled into 3 sets of contigs with Velvet, using 3 different kmer values (kmer = 45, 51 or 55). (PDF) [file pgen.1002984.s001.pdf]
