# Supplementary figures and images for: The Paramecium Germline Genome Provides a Niche for Intragenic Parasitic DNA: Evolutionary Dynamics of Internal Eliminated Sequences
Source: PLoS Genet. 2012 Oct 4;8(10):e1002984. doi: 10.1371/journal.pgen.1002984 (PMC3464196; doi:10.1371/journal.pgen.1002984)

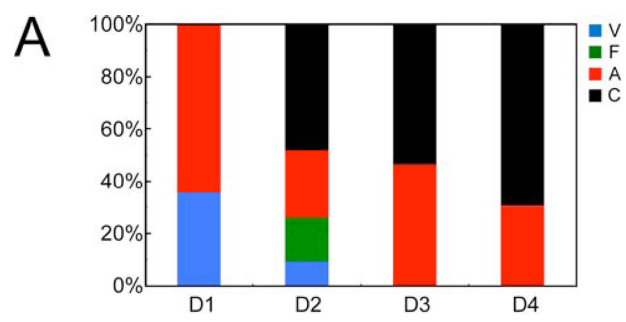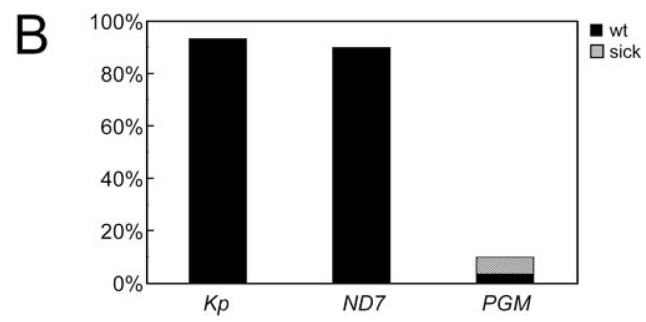

Supplement: Figure S2 — Autogamy time-course of P. tetraurelia 51 mt8 submitted to RNAi against PiggyMac. A. Cells were transferred at day 0 into 4.1 L of freshly induced feeding bacteria producing dsRNA homologous to a 567-bp region of the PGM gene and incubated at 27°C. The progression of autogamy was monitored everyday (D1: day 1, D2: day 2, D3: day 3, D4: day 4) by DAPI staining of cells. V: vegetative cells, F: cells with fragmented old MAC and no clearly visible new developing MACs, A: cells harboring two developing new MACs, C: post-autogamous cells with one new MAC surrounded with fragments of the old MAC. B. Survival of post-autogamous progeny. At day 4, 30 autogamous cells were transferred individually to standard growth medium containing K. pneumoniae and incubated at 27°C to follow the resumption of vegetative growth. Survival of the progeny of autogamous cells obtained in standard (Kp) or in control RNAi medium (ND7) was also tested. Wt: normally-growing progeny, sick: slowly-growing cells, often with abnormal swimming behavior. (PDF) [file pgen.1002984.s002.pdf]

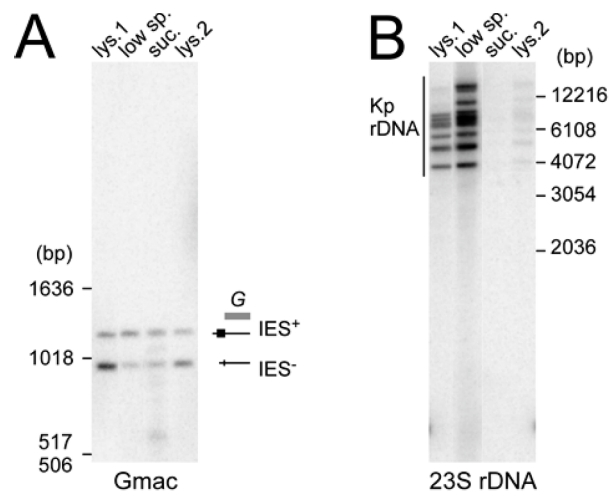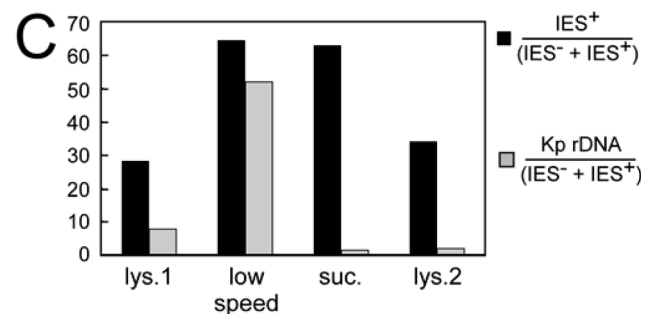

Supplement: Figure S3 — Purification of IES-enriched genomic DNA from PGM-silenced cells. Autogamous cells were collected at day 4 and genomic DNA was extracted through several cell fractionation steps. Lys.1 and lys.2: independent samples of cells were lysed directly in proteinase K buffer; low sp.: DNA extracted from low speed pellets (600× g for 1 min followed by washing); suc.: DNA extracted from nuclear pellets obtained following centrifugation through a 2.1 M sucrose layer. Each DNA sample was digested by PstI and the digestion fragments were separated on a 1% agarose gel. A. Southern blot hybridization with the Gmac probe (shown as a grey box on the diagram). The position of size markers is shown on the left. IES− and IES+ bands were quantified separately. B. Southern blot hybridization with the K. pneumoniae 23S rDNA probe. Size markers are shown on the right. All rDNA bands were quantified together. C. Quantification of radioactive signals from the blots shown in A and B. The fraction of IES+ form was normalized relative to the sum of IES− and IES+ signals (black histograms). Bacterial rDNA was normalized relative to the sum of IES− and IES+ signals (grey histograms). (PDF) [file pgen.1002984.s003.pdf]

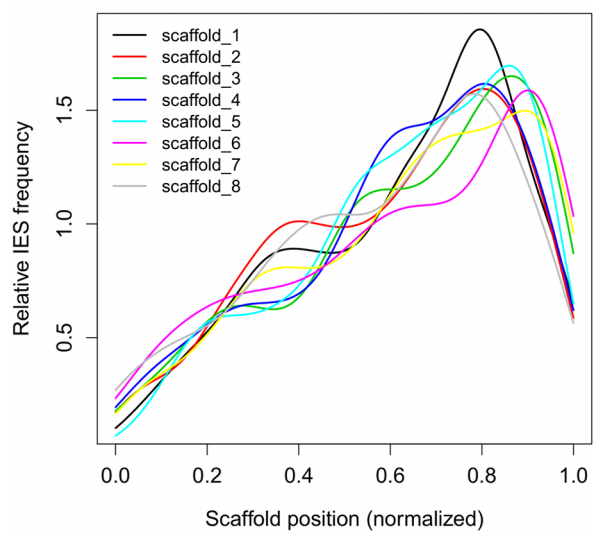

Supplement: Figure S4 — IES distribution on the 8 largest MAC chromosomes. The 8 largest, telomere-capped scaffolds (∼750 Kb to ∼980 Kb in size) were normalized to length 1.0 and some were flipped so that the highest IES density is to the right. The curves represent histograms of IES position on each scaffold after Gaussian smoothing using the R “density” function [92]. IES distribution was evaluated using a Kolmogorov-Smirnov test of the null hypothesis that IESs are uniformly distributed on the scaffold. For the 8 largest scaffolds, the null hypothesis was strongly rejected (p<10−8). The same statistical test was carried out for gene distribution on these chromosomes, and the null hypothesis was not rejected, consistent with a uniform distribution of genes on the chromosomes. (PDF) [file pgen.1002984.s004.pdf]

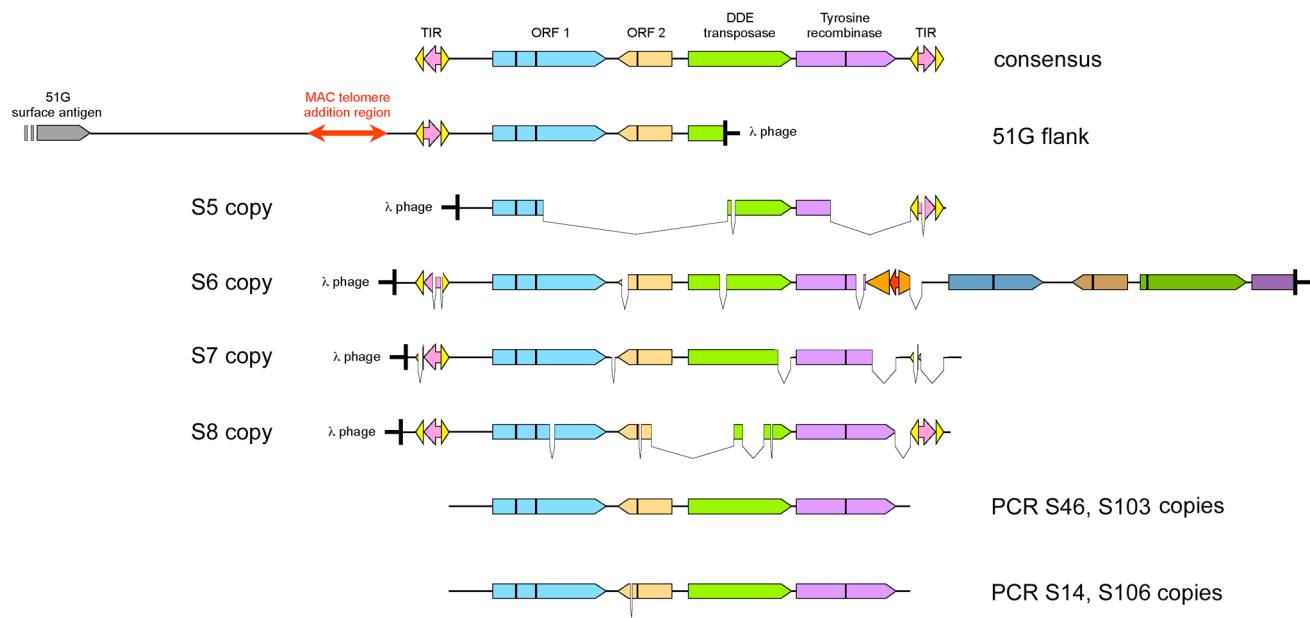

Supplement: Figure S5 — Sardine and Thon Tc1/mariner family transposons. From top to bottom: 1) Sardine transposon consensus sequence obtained by alignment of the lambda-phage and PCR copies (the latter were amplified from total DNA of vegetative cells using primers located within the Sardine TIRs), showing the presence of palindromic TIRs and 4 putative ORFs, including a DDE transposase and a tyrosine recombinase; 2) lambda-phage with the 51G flank that led to discovery of the Sardine element (the region of de novo telomere addition at the end of the MAC chromosome, following developmental breakage of the MIC chromosome, is indicated); 3) lambda-phage with the S5 copy of Sardine; 4) lambda-phage with the S6 copy of Sardine, containing an insertion of a different Tc1/mariner transposon, Thon, which has the same general organization as the Sardine element; 5) lambda-phage with the S7 copy of Sardine; 6) lambda-phage with the S8 copy; 7) PCR products (S46 and S103 copies) with nearly intact ORFs; 8) PCR products (S14 and S106 copies) with nearly intact ORFs. The sequences of the 5 lambda-phages and 4 PCR products have been deposited in the EMBL/GenBANK/DDBJ public nucleotide database with EMBL-Bank accession numbers HE774468–HE774475. (PDF) [file pgen.1002984.s005.pdf]

**A**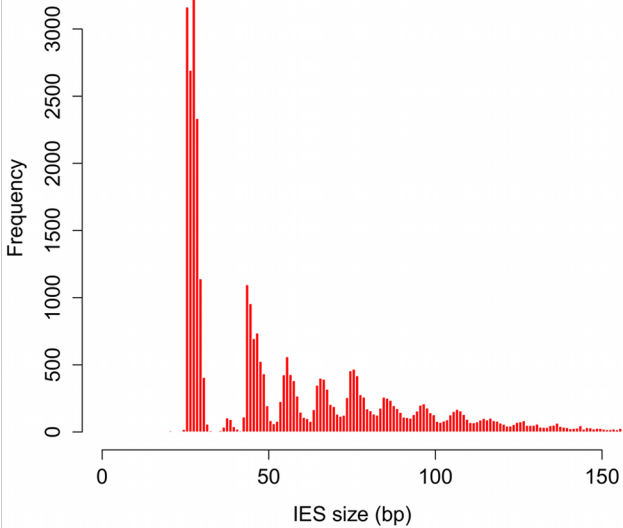**B**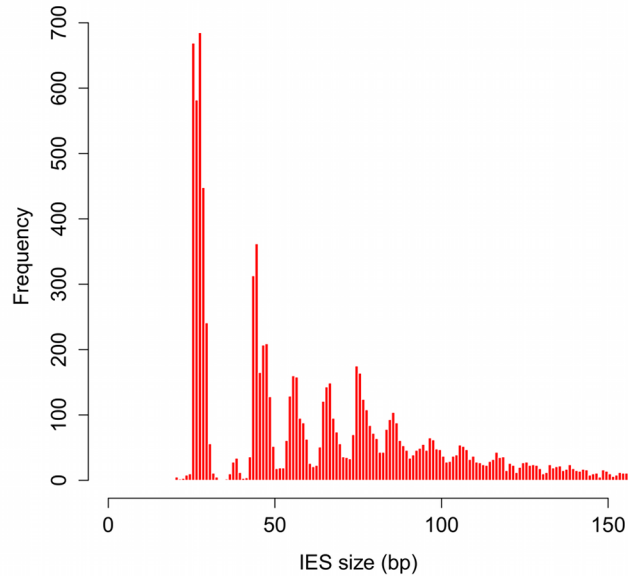

Supplement: Figure S6 — IES size distribution. The histograms represent A) IESs inserted in coding sequences. B) IESs inserted in non-coding sequences. IESs larger than 150 nt are not displayed. The fact that very similar periodic distributions are observed for IESs in both coding and intergenic regions is consistent with the hypothesis that the periodic size constraint is related to the IES excision mechanism. Indeed, IES retention in the MAC could be deleterious either by affecting ORFs (IESs in protein coding sequences) or by affecting regulatory signals (IESs in non-coding sequences). (PDF) [file pgen.1002984.s006.pdf]

**A**

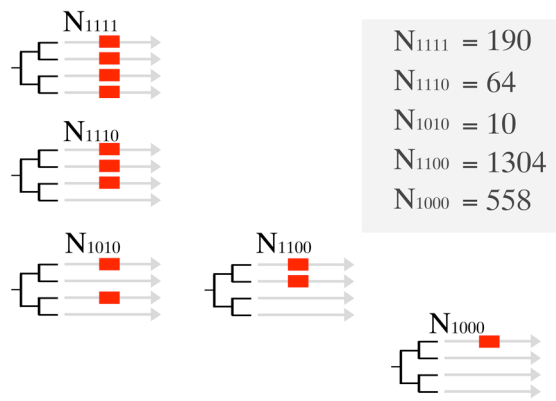

**B**

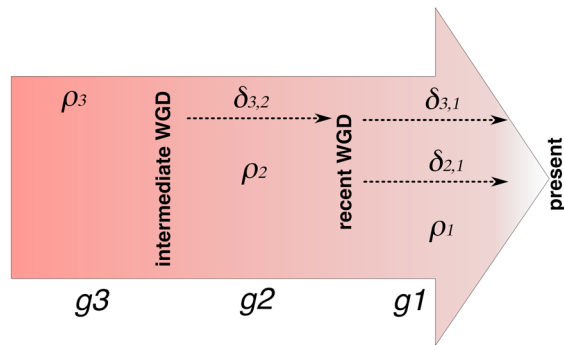

Supplement: Figure S7 — IES evolution evaluated with quartet IES groups. A) schematic representation of the observable quartet IES groups, arranged from top to bottom according to the number of IESs that are conserved and from left to right, according to the most recent period in which the ancestral IES could have been acquired. B) Schematic representation of the parameters of a statistical model developed to test hypotheses about IES evolution (cf. Text S1). The three time periods delimited by the 2 WGD events and the present time are designated, from the oldest to the most recent, g3, g2 and g1. The parameters ρ3, ρ2 and ρ1 are the fraction of IESs that were acquired in each of these time period and the parameters of the form δa,b are the survival rates for an IES acquired in period ga during the period gb. The equations of the model express the observable IES counts as a function of these parameters. (PDF) [file pgen.1002984.s007.pdf]
