# Supplementary material for: The Paramecium Germline Genome Provides a Niche for Intragenic Parasitic DNA: Evolutionary Dynamics of Internal Eliminated Sequences
Source: PLoS Genet. 2012 Oct 4;8(10):e1002984. doi: 10.1371/journal.pgen.1002984 (PMC3464196; doi:10.1371/journal.pgen.1002984)
Supplement: Table S1 — Assembly statistics. (PDF) [file pgen.1002984.s008.pdf]

Dataset	Million reads	Kmer	No. of Contigs	Largest contig (nt)	Complexity (nt)	N50	MAC genome coverage
1	126	45	38714	159958	96838332	18877	96.29%
1	126	51	32891	177346	97451433	26902	96.38%
1	126	55	42969	82851	99085030	7298	97.30%
2	32	45	94491	107860	61535444	1087	49.74%
2	32	51	80141	86502	59448180	1180	46.45%
2	32	55	71678	82851	58477670	1230	44.89%
3	121	45	26721	158845	95582645	24907	95.91%
3	121	51	23091	187719	96119509	29755	95.65%
3	121	55	22059	138361	96437319	25160	96.63%
4	121	55	20728	243532	96552463	32589	96.42%

Table S1. Assembly statistics. The datasets are as follows : 1) All PGM reads; 2) All pairs of PGM reads with at least one read that does not map to the MAC reference genome; 3) All PGM reads after removal of those reads that do not match a putative MAC IES junction identified by the MIRAA pipeline; 4) All PGM reads after removal of those reads that do not match a MAC IES junction identified using the MICA pipeline and the first 9 assemblies. Paired-end assembly was carried out using the Velvet short read assembler (version 1.0.18) with the indicated Kmer value. The MAC genome coverage by the assembled contigs was determined by mapping them to the reference genome with BLAT.
