# Supplementary material for: The Paramecium Germline Genome Provides a Niche for Intragenic Parasitic DNA: Evolutionary Dynamics of Internal Eliminated Sequences
Source: PLoS Genet. 2012 Oct 4;8(10):e1002984. doi: 10.1371/journal.pgen.1002984 (PMC3464196; doi:10.1371/journal.pgen.1002984)
Supplement: Table S2 — Molecular validation of some predicted IESs and IES insertion sites. (PDF) [file pgen.1002984.s009.pdf]

Type	Scaffold	Position	MAC PCR product		MIC PCR product		IES size	Sequenced
			Expected	Observed	Expected	Observed		
MICA & MIRAA	scaffold51_1	292806	369	~375	454	~460	85	no
MICA & MIRAA	scaffold51_10	240455	355	~360	432	~450	77	no
MICA & MIRAA	scaffold51_11	141916	364	~375	573	~560	209	no
MICA & MIRAA	scaffold51_12	366211	350	~350	377	~375	27	MIC
MICA & MIRAA	scaffold51_14	132164	361	~360	408	~410	47	no
MICA & MIRAA	scaffold51_2	623719	348	~350	404	~410	56	no
MICA & MIRAA	scaffold51_3	554680	367	~360	403	~400	36	MIC
MIRAA	scaffold51_1	206570	322	~330	ND	~350	*31-2-28	MIC
MIRAA	scaffold51_11	460330	375	~400	ND	~450	*29-1-29	MIC
MIRAA	scaffold51_13	596382	347	~350	ND	~400	*28-2-30	MIC
MIRAA	scaffold51_14	210046	366	~375	ND	~800	ND	no
MIRAA	scaffold51_15	406155	369	~375	ND	~650	ND	no
MIRAA	scaffold51_16	361775	350	~350	ND	~400	*26-3-26	MIC
MIRAA	scaffold51_9	114280	342	~350	ND	~460	*57-1-46	MIC
MIRAA	scaffold51_56	454715	332	~340	ND	~3200	ND	no
MIRAA	scaffold51_47	324362	358	~360	ND	~410	*47-4-29	MIC
MIRAA	scaffold51_34	257109	394	~400	ND	~650	*106-6-148	MIC
MIRAA	scaffold51_41	194697	378	~400	ND	~475	*28-3-58	MIC
MIRAA	scaffold51_26	330923	345	~360	ND	~500	146	MIC
MIRAA	scaffold51_35	149456	358	~375	ND	none	ND	no
MIRAA	scaffold51_43	182962	366	~400	ND	none	ND	no
MIRAA	scaffold51_37	205471	343	~350	ND	none	ND	no
MIRAA	scaffold51_33	261960	349	~350	ND	none	ND	MAC
MIRAA	scaffold51_28	472294	358	~400	ND	none	ND	no
MICA	scaffold51_179	88164	333	~340	360	~375	27	no
MICA	scaffold51_184	64423	308	~325	353	~350	45	no
MICA	scaffold51_188	26049	351	~360	378	~380	27	no
MICA	scaffold51_188	29158	346	~350	390	~400	44	MIC
MICA	scaffold51_71	364423	373	~390	400	~480	27	no
MICA	scaffold51_87	125232	358	~360	385	~390	27	no

Table S2. Molecular validation of some predicted IESs and IES insertion sites. IES insertion sites predicted by one or both methods (MICA and/or MIRAA) were tested using PCR as described in Materials and Methods. For each tested MICA prediction, the MIC PCR product was of the expected size, and for those that were sequenced, the expected IES sequence was found. For insertion sites predicted only by MIRAA, PCR amplifications show unexpected results : IESs very close one to another, spaced by only one or a few nucleotides, were often found. Out of 17 tested MIRAA predictions, 8 appeared to be such tandem IESs, 1 appeared to be an 146 bp IES, in 5 cases we did not obtain a MIC amplification product, and in the remaining 3 cases, the MIC amplification product was not sequenced. Failure to dectect a MIC product does not exclude the presence of a large IES, given the PCR conditions that were used.

Legend.
* Tandem IESs with size format : IES_1_size – space_size – IES_2_size.
~ Approximate size of the corresponding band in the migration gel.
ND (Expected MIC): we could not predict any size for a MIC amplification because no *a priori* prediction was made on the presence of a MIC sequence.
none (Product MIC): no amplification other than the MAC product could be observed.
ND (IES size): the MIC product was not sequenced or its size could not be predicted.
