# Supplementary material for: The Paramecium Germline Genome Provides a Niche for Intragenic Parasitic DNA: Evolutionary Dynamics of Internal Eliminated Sequences
Source: PLoS Genet. 2012 Oct 4;8(10):e1002984. doi: 10.1371/journal.pgen.1002984 (PMC3464196; doi:10.1371/journal.pgen.1002984)
Supplement: Table S3 — Validation of the genome-wide set of IESs using previously characterized IESs. (PDF) [file pgen.1002984.s010.pdf]

Description	GenBank Accession N°	Scaffold	Position	% ID	known IES length (bp)	IES length (this study) (bp)	COMMENT
ND7_IES4_mic	gi|193480009	scaffold51_5	597595	100	29	29	
ND7_IES3_mic	gi|193480009	scaffold51_5	595021	100	26	26	
ND7_IES2_mic	gi|193480009	scaffold51_5	594919	100	76	76	
ND7_IES1_mic	gi|193480009	scaffold51_5	594675	100	28	28	
IESsm19-576_mic	GB	scaffold51_8	407305	100	66	66	
IES51pwB-658_mic_alternative	gi|10716831	scaffold51_19	587496	100	146	146	
IES51pwB-427_mic	gi|10716831	scaffold51_19	587727	100	44	44	
IES51pwB-2226_mic	GB	scaffold51_19	587126	100	66	66	
IES51PAK11-991_mic	gi|3916119	scaffold51_5	724979	100	28	28	
IES51PAK11-294_mic	gi|3916119	scaffold51_5	725700	100	45	45	
IES51PAK11-1557_mic	gi|3916119	scaffold51_5	724388	100	44	44	
IES51PAK1-991_mic	gi|3894330	scaffold51_16	560295	100	27	27	
IES51PAK1-1557_mic	gi|3894330	scaffold51_16	559704	100	44	44	
IES51PAK1-1036_mic	gi|3894330	scaffold51_16	560250	100	46	46	
IES51ICL1b_mic	gi|1666902	scaffold51_134	33201	100	75	75	
IES51G6447_mic	gi|3452504	scaffold51_51	457658	100	28	28	
IES51G4404_mic	gi|3452504	scaffold51_51	455615	100	222	222	
IES51G2832_mic	gi|3452504	scaffold51_51	454043	100	229	229	
IES51G1832_mic	gi|3452504	scaffold51_51	453043	100	30	30	
IES51G1413_mic	gi|3452504	scaffold51_51	452624	100	52	52	
IES51G-11_mic	gi|3452504	scaffold51_51	451201	100	43	43	
IES51B5464_mic	gi|76589367	scaffold51_143	33517	100	28	28	
IES51B1417_mic	gi|76589367	scaffold51_143	37564	100	44	44	
IES51B-9_mic	gi|76589367	scaffold51_143	38990	100	36	36	
IES51A6649_mic	gi|78707424	scaffold51_106	288995	100	370	370	
IES51A6435_mic	gi|78707424	scaffold51_106	288781	100	28	28	
IES51A4578_mic	gi|78707424	scaffold51_106	286924	100	883	883	
IES51A4404_mic	gi|78707424	scaffold51_106	286750	100	77	77	
IES51A2591_mic	gi|78707424	scaffold51_106	284913	100	370	370	
IES51A1835_mic	gi|78707424	scaffold51_106	284157	100	28	28	
IES51A1416_mic	gi|78707424	scaffold51_106	283738	100	74	74	
IES51A-712_mic	gi|78707424	scaffold51_106	281631	100	77	77	
IES51A-4814_mic	gi|29469820	scaffold51_106	277532	100	28	28	
IES51A-10_mic	gi|78707424	scaffold51_106	282313	100	29	29	
epsilon51D_28bp_mic	gi|1752672	scaffold51_128	10690	100	28	28	
alpha51D_IES5_mic	gi|1752672	scaffold51_159	213381	100	28	28	
alpha51D_IES4_mic	gi|1752672	scaffold51_159	209504	100	44	44	
alpha51D_IES3_mic	gi|1752672	scaffold51_159	207374	100	28	28	

alpha51D_IES1_mic	gi|1752672	scaffold51_159	205692	100	26	26	
51ICL1d_IES3_mic	gi|1667584	scaffold51_124	172613	100	29	29	
51ICL1d_IES2_mic	gi|1667583	scaffold51_124	173737	100	45	45	
51ICL1d_IES1_mic	gi|1667583	scaffold51_124	173840	100	26	26	
**IES51pwB-658_mic**	**gi|10716831**	**scaffold51_19**	**587496**	**100**	**155**	**146**	**alternative boundary form not found**
**IES51A6649_29bp_mic**	**gi|78707424**	**NA**	**NA**	**0**	**29**	**NA**	**nested IES**
**IES51A2591_28bp_mic**	**gi|78707424**	**NA**	**NA**	**0**	**28**	**NA**	**nested IES**
**IES51A15885_mic**	**GB**	**NA**	**NA**	**0**	**54**	**NA**	**assembled in MAC reference**
IES51pwB-383_mic	GB	scaffold51_19	588978	100	47	49	
IES51PAK11-1036_mic	gi|3916119	scaffold51_5	724934	98.1	50	50	
IES51PAK1-538_mic	gi|3894330	scaffold51_16	560748	99.5	206	206	
IES51PAK1-294_mic	gi|3894330	scaffold51_16	561014	98.8	78	78	
IES51B3931_mic	gi|76589367	scaffold51_143	35050	99.5	416	416	
IES51A15637_mic	GB	scaffold51_106	297971	98	47	47	
alpha51D_IES2_mic	gi|1752672	scaffold51_159	206601	96.8	29	28	

Table S3. Validation using previously characterized IESs. Known *P. tetraurelia* strain 51 IESs were taken from GenBank. The IESs with no GenBank Accession Number had been communicated to M.B. and are designated "GB" to indicate that they are included in the compilation in [1]. Known IESs were compared with the genome-wide set of IESs by BLASTN. The four IESs that were not found are in bold. Sequencing errors in IESs deposited in GenBank, or SNPs between different 51 stocks, probably explain identity scores below 100%.

Reference

1. Gratias A, Bétermier M (2001) Developmentally programmed excision of internal DNA sequences in *Paramecium aurelia*. Biochimie 83: 1009–1022.
