# Supplementary material for: The Paramecium Germline Genome Provides a Niche for Intragenic Parasitic DNA: Evolutionary Dynamics of Internal Eliminated Sequences
Source: PLoS Genet. 2012 Oct 4;8(10):e1002984. doi: 10.1371/journal.pgen.1002984 (PMC3464196; doi:10.1371/journal.pgen.1002984)
Supplement: Table S4 — IESs with homology to Anchois transposons. (PDF) [file pgen.1002984.s011.pdf]

IES (ParameciumDB Accession)	Size (nt)	Location of IES	Gene ohnolog(s)	Ohnologous IES	Element	Match %ID	Match Length (nt)
IESPGM.PTET51.1.98.309432	2003	intergenic	-	none	A	72	883
IESPGM.PTET51.1.128.254421	3392	GSPATG00032293001	none	none	A	76	2981
IESPGM.PTET51.1.77.209216	4154	intergenic	-	none	A	75	3714
IESPGM.PTET51.1.103.177611	2462	GSPATG00028199001	Recent WGD	none	B	87	2464
IESPGM.PTET51.1.104.49056	2238	GSPATG00028299001	none	none	B	89	2228
IESPGM.PTET51.1.105.239361	2483	intergenic	-	none	B	80	2166
IESPGM.PTET51.1.120.182371	1769	GSPATG00031026001	Intermediate WGD	none	B	80	1648
IESPGM.PTET51.1.132.167159	1500	intergenic	-	none	B	86	1465
IESPGM.PTET51.1.163.702	1956	GSPATG00036746001	none	none	B	74	1209
IESPGM.PTET51.1.169.56908	3272	GSPATG00037429001	Recent WGD	none	B	87	3272
IESPGM.PTET51.1.173.70900	2714	intergenic	-	none	B	67	1466
IESPGM.PTET51.1.174.130670	3001	GSPATG00037898001	Recent WGD	none	B	75	2955
IESPGM.PTET51.1.181.1750	3001	GSPATG00038327001	none	none	B	74	2411
IESPGM.PTET51.1.214.11549	2317	GSPATG00038730001	none	none	B	81	2159
IESPGM.PTET51.1.24.100577	1340	GSPATG00009289001	none	none	B	75	1265
IESPGM.PTET51.1.28.457973	2468	intergenic	-	none	B	68	895
IESPGM.PTET51.1.29.290535	1722	GSPATG00010910001	Recent WGD	none	B	78	1695
IESPGM.PTET51.1.35.111752	2820	GSPATG00012627001	none	none	B	85	2692
IESPGM.PTET51.1.42.397702	2473	GSPATG00014783001	Recent WGD	none	B	84	2263
IESPGM.PTET51.1.42.72890	3389	intergenic	-	none	B	89	3389
IESPGM.PTET51.1.47.408041	2125	GSPATG00016182001	Intermediate WGD	none	B	79	2125
IESPGM.PTET51.1.50.348282	1251	intergenic	-	none	B	81	1150
IESPGM.PTET51.1.51.131273	2219	GSPATG00017060001	Recent WGD	none	B	76	1245
IESPGM.PTET51.1.57.48117	1257	GSPATG00018530001	Recent WGD	none	B	82	1257
IESPGM.PTET51.1.76.220822	3048	GSPATG00022960001	Recent WGD	none	B	84	2933
IESPGM.PTET51.1.77.311405	3470	intergenic	-	none	B	85	1254
IESPGM.PTET51.1.80.84925	1513	GSPATG00023746001	none	none	B	82	1515
IESPGM.PTET51.1.85.45587	3479	GSPATG00024746001	none	none	B	78	2786

Table S4. IESs used to identify the *Anchois* transposon family. The BLASTN match of each IES with the final consensus of the A or B element, as indicated, is given in the last 2 columns of the table (cf. Text S1 for the alignments used to reconstitute the final *AnchoisA* and *AnchoisB* consensus sequences). Match length is the sum of non-overlapping HSPs. Match %ID is the weighted average percent identity of the HSPs.
