# Supplementary material for: The Paramecium Germline Genome Provides a Niche for Intragenic Parasitic DNA: Evolutionary Dynamics of Internal Eliminated Sequences
Source: PLoS Genet. 2012 Oct 4;8(10):e1002984. doi: 10.1371/journal.pgen.1002984 (PMC3464196; doi:10.1371/journal.pgen.1002984)
Supplement: Table S5 — Deficit of 3n IESs in coding sequences, for each peak of the 10 bp periodic size distribution. (PDF) [file pgen.1002984.s012.pdf]

IES category	Peak	Sizes (nt)	Number of IESs	3n	3n+1	3n+2
**Non-coding**	**-**	**-**	**10304**	**3481 (33.78%)**	**3101 (30.1%)**	**3722 (36.12%)**
	1	0-34	2725	836 (30.68%)	753 (27.63%)	1136 (41.69%)
	2	*34-42*	*93*	*40 (43.01%)*	*22 (23.66%)*	*31 (33.33%)*
	3	42-52	1509	589 (39.03%)	348 (23.06%)	572 (37.91%)
	4	52-63	843	305 (36.18%)	250 (29.66%)	288 (34.16%)
	5	63-73	793	252 (31.78%)	289 (36.44%)	252 (31.78%)
**Coding stopwith**	**-**	**-**	**11205**	**3700 (33.02%)**	**3515 (31.37%)**	**3990 (35.61%)**
	1	0-34	2231	728 (32.63%)	616 (27.61%)	887 (39.76%)
	2	*34-42*	*78*	*25 (32.05%)*	*15 (19.23%)*	*38 (48.72%)*
	3	42-52	1391	459 (33%)	368 (26.46%)	564 (40.55%)
	4	52-63	975	321 (32.92%)	297 (30.46%)	357 (36.62%)
	5	63-73	880	267 (30.34%)	330 (37.5%)	283 (32.16%)
**Coding stopless**	**-**	**-**	**23339**	**7095 (30.4%)**	**7049 (30.2%)**	**9195 (39.4%)**
	1	0-34	10943	3123 (28.54%)	3144 (28.73%)	4676 (42.73%)
	2	*34-42*	*240*	*88 (36.67%)*	*61 (25.42%)*	*91 (37.92%)*
	3	42-52	3510	1108 (31.57%)	936 (26.67%)	1466 (41.77%)
	4	52-63	1834	561 (30.59%)	621 (33.86%)	652 (35.55%)
	5	63-73	1520	457 (30.07%)	545 (35.86%)	518 (34.08%)

Table S5. Deficit of 3n IESs in coding sequences evaluated by peak of the 10 bp periodic size distribution. The distribution of IES length modulo 3 is shown for IESs in non-coding (intron + intergenic) and coding (exon) regions of the genome (cf. Table 4). The coding IESs are further separated according to whether or not they contain a stop codon in phase with the upstream exon sequence. The modulo 3 counts are also presented for each of the first 5 peaks of the 10 bp periodic size distribution; the 2nd "forbidden" peak (see Discussion), containing very few IESs, is in italics.

The 10 bp periodicity in the IES size distribution, a strong constraint probably imposed by IES excision geometry (see Discussion), causes a distortion of the modulo 3 length distribution, both in coding and non-coding sequences. The relative proportion of 3n, 3n+1 and 3n+2 IESs differs from 1/3 for each class and varies among the different peaks of the 10 bp periodicity, leading to an overall excess of 3n+2 IESs. However, comparison of the 3n values for each peak shows in all cases a lower percentage of 3n IESs in coding ("stopless" counts) than in non-coding regions of the genome. Furthermore, there is a lower percentage of 3n "stopless" IESs than 3n "stopwith" IESs for all of the peaks except the second one (in italics), which contains very few IESs.

The two constraints on IES size appear to be independent. First, the same 10 bp periodicity is found both for IESs inserted in coding and in non-coding sequences (Fig. S6). Second, the analysis of each individual peak of the 10 bp periodicity shows that in all cases, the frequency of 3n IESs is lower for stopless IESs in coding sequences compared to IESs in non-coding sequences. The observed deficit of 3n stopless IESs in exons is therefore not an indirect consequence of the 10 bp periodicity in the IES size distribution.
