# Supplementary material for: The Paramecium Germline Genome Provides a Niche for Intragenic Parasitic DNA: Evolutionary Dynamics of Internal Eliminated Sequences
Source: PLoS Genet. 2012 Oct 4;8(10):e1002984. doi: 10.1371/journal.pgen.1002984 (PMC3464196; doi:10.1371/journal.pgen.1002984)
Supplement: Text S1 — Transposon sequences. A). The sequences of Sardine, Thon and Anchois transposons reconstituted from manually adjusted multiple alignments of the different decayed copies, cloned from the lambda phage library of MIC DNA (Sardine, Thon) or found in the PGM DNA assembly (Anchois). The sequences of the Thon transposon are those of the only known copy, so that ORF annotation (based on homology with the Sardine element) is preliminary; the Thon ORF1 sequence apparently contains a frameshift. Predicted introns have been removed from the ORF sequences. B) Annotated comparison of AnchoisA and AnchoisB, showing the position and orientation of the ORFs, with a potential intron in the DDE transposase ORF. C). Manually adjusted alignment used to reconstitute the AnchoisA copy. See Text S4 for the IESs used in the reconstitution. D). Manually adjusted alignment used to reconstitute the AnchoisB copy. See Text S4 for the IESs used in the reconstitution. E) IESs used to obtain the final AnchoisA and AnchoisB consensus sequences based on the manually adjusted alignments in C) and D). (PDF) [file pgen.1002984.s013.pdf]

Text S1. Transposon sequences.

A) The sequences of *Sardine, Thon* and *Anchois* transposons reconstituted from manually adjusted multiple alignments of different decayed copies, cloned from the lambda phage library of MIC DNA (*Sardine*, *Thon)* or found in the PGM DNA assembly (*Anchois*). The sequences of the *Thon* transposon are those of the only known copy, so that ORF annotation (based on homology with the *Sardine* element) is preliminary; the *Thon* ORF1 sequence apparently contains a frameshift. Predicted introns have been removed from the ORF sequences.

B) Annotated comparison of *AnchoisA* and *AnchoisB,* showing the position and orientation of the ORFs, with a potential intron in the DDE transposase ORF.

C) Manually adjusted alignment used to reconstitute the *AnchoisA* element. Some PGM contig regions that correspond to imprecisely excised germline DNA have been included in this alignment.

D) Manually adjusted alignment used to reconstitute the *AnchoisB* element. Some PGM contig regions that correspond to imprecisely excised germline DNA have been included in this alignment.

E) IESs used to reconstitute *AnchoisA* and *AnchoisB* consensus sequences. The table provides the correspondence between the ParameciumDB IES accessions and the labels used in the alignments in C) and D). In a number of cases, “Alignment Length” is 2 nt longer than “IES Size”, since both flanking TA dinucleotides are included in the alignment. Some IESs used for the initial alignment and identification of *Anchois* (cf. Table S4) were not included in the final, manually adjusted alignments shown here.

A

```
>Sardine consensus (6,673 nt with duplicated TAs)
TATAGCTGTTTGCTTAAAAAATATATAATTGATTTCATAATATAATAATTAAATTTATAT
TGAGTCAATATAATAAGCCTATTATATTGACTCAATATAAATGATTTATACTACAGAAAA
TTTATATTATGGTgAAAAAGAAATTAATCTTTTTCACCAAATATTTAATTAATTAATACA
TAAATTCAAAAAATTATTCTTCAATTCTTTTTACAAAAATGCATTGTGAAAAAGAATGAT
CTTTTTCACAATATAATTAATTGAATGGTGAAAAAGAACTATTCTTTTTCACAGTTTAAT
TTTTATCATAATACAAATCAATTATATTGAGTCAATATAATAGGCTTATTATATTGACTC
AATATAAATTTAATTATTATATTATGAAATCAATTATATATTTTTTAAGCAAACAGCTAT
GTCTTTTTAAATATTAAGGATTtAATTAGTTTAGTTAATTTCTATTGCAAAATTGTGTTT
AACTATCTATCTCTACGAAACTTTTGCTAGTATATATAACCTTCATTAATTTATATTTTA
TACAATGAGATCAATTTCATTTAATTCTTTCATTTGAATCATAGATTAGTCGATAACTTT
TTATAGATAGATTATTCACTTTGCTTTTGAAGCTCAAAACCATTATATAATAAGTATAGA
ATAATTCTTAATTATTAATGTAAATTTATATGATAATCTAACACAGTTTCTCAAACTAAT
AAACTAATTAATTCATTCAACAGATTATCCTCAAACATTCTATGATTCTATCATATAATA
TTATTAATTTTATTACTTTCTCAACCGTTTCTTAATTTACTGATTTTCACCTTATTTTTA
TCTTATTATATAAATGTTATCTTTATTCTATTTATTTAAAATAGTTGAGGGCCTCGATTA
ACAACCATAAAATTTTTTTGTAGTAAGACCATAATATTTGCAATTCATATTTTTTTTTTG
TATTAATTATTTATTCAAATGAATTAAAACAATTTATTTTTAAAACTTTAGTTTTTTAAA
TATGTTCCAAACACCTACAATTAATGTTTGATATGTAATGAATTAGTTTGCTTGTAAGAA
ACAAATCAATAATTTGAAAAAAAAATAAATGAACACCTCCTATCCTTTAATCATCTTAAA
GCACAAAAAGAAATTCTAGACCAAACTGGATTCCTTTAGAACTAGACATCTATAAATCAT
GAGAGCGAAAGACAATCTTATCCACTATATGATCTTCAAATTGCCAGAGAATTAAATTAA
ATTGGTATGAAATCCATAAATGATTGCGATTAGGTCTAAATCTTTACTTTTAGAATTGCA
TATTAAAACTTGATAATTTTCTTTCTAAAAAGCAATCAACATAAAAGGAATACGGGATTA
TCTTAAAAAGGTTTTATCATATGCTTTTTATCAATTAAACAAATTAAATTTCAGATTAAA
TTATAATAAAGTTTCTTTAGAATGAAGAGTATCGACATGATAGTATTTCAGCACTAAGAC
TTTTAGCAAAATAAGCTGGTATTTATAATTAATAAATTAATAAGGCCATTAAATATCACT
TTAGGTTTAAAATAATATTCAGATTGAAAAAAAAAATTAATTAAAAAAGAATAAAAAAAA
AGCTCAATTTGAATTGCTTTGTTGTATATAGTTTTTAGAATTATTATTAAAAAATCTGAA
TTACAATTTGGCTTTTCTTTTGCTTTTACTTTTTGAGTTGGATTTAGAACTAAGTTGTAT
TTCAATCCTTACATTTCAGGATGTTTTATCAGATAAAATCGAAATAAACAAAGAGAAGTT
TGATAAATTAAGAACAAAAACGGTGCTTATTGGTAAATCATTATAATAGGCGGCAAAAAT
AACATTAAAAGTTTTAGGACTACCTTTAAGTTCACCTATTTTTCCTTTAATTAGAAATTA
TAGAGATATACAAAGTCTATTAGAAAAACTTAAAAAAGAATATAAGTTTTAACTTGAAGC
ATATAATGAGGAGACCAAATAATTTATATAGAATTTTCATCTTTCAGTTATAAAATTTAT
AAAGAAATCGATCTACCCTTCAAAATAAATATAGTCATTTTTGGAAAGCTAAGTAGCAAA
TCATAAAAATAGCAATAAATAATCTGATGCAGCTTTTCCAGCTCTATTTGAAATtTTCTT
CGCGTATTTTGAATCCATAAAATAAAAATAAATAATTGGGAATTCAAAAGTTAATTCAAT
TATTAATAATAATGTTTTTAAAAAGAGGAGTAAATCTGAATTATAAATTATAAGAAATAG
AAACATAGAATAAATGAGAGAAGCAACTtAAATTGTTGTACCTTTAGAGCAATTCTGTTA
AGATTAACAGTACCAAAATCATGTATATAAACTAATTGAATAAATAGAATTCATTAATTG
GCAATTAAGTCCTTGAGAAAAAcATCAAAGTAGATAAATCCTAATAAAACTCATTAAATT
AGGATCAATCTTTAATAATTTCTGATTGAAAACAAAGAATAAGAAATTATTTATTAGAAG
TAATAATATAAAACAATAATTTTTAAACaATAACTATGTAATAATTTATGTTCATTCCCC
TTCGATATCCTTTATTTTGTTTTCAATTAACTTTAATTCTTTTTCATAATCATCAATTAT
TTTTAGCTTTCTGTCCATTTTACTTTCTAAAGTTTTCTTCTTCAAAATTTTCTTTTGTTT
ATCTGCTTCCATTTATTTATCTTTTGATATACGAAAAAATCTTTTAACATTTGTTTTCTT
ACCCATTGAAAAATCTAACTCTTTAATCTTTCTAAAAATTAAATTTCATAAATACTTGAT
CTGATCTTGAGTGAAGATCTTCATATCATACTTTGAATCGGTTTTATATTTTTGAAAGAA
AGAATGATTCGGTTTTAAAGTCTTTAAAACGTTTATAATCCAATCTTTGTCAGGTTGGTG
CTTTTAATCTATTCCCCAAGTTTTTTATTATATTGGTATTTTTTGGACCTCCTCTATTAT
TTCGTATTTTTTCACTGCCTTAAGTAGTACCCCAATTTAAACTTCAGATAAATTAGGGAC
ATAGATTTTCTCAAAAAGTACACCCAATAAATATTCTGATGTAATAATCGAAGATGTCAA
TTTTGGTAAATATTTCCCTTAAATCAGCAATCCATAATAAGCTGATTCCAAGGAAGGTAT
TAATTAATTAGCATCTGCTCGAGATATTTTGAATTCATTCTCTTCTTCTTAAAATATATT
TTACATTTTATTTTCACTTCAAAAAATAATTAATTGAATAAACATTTCAATTAAATATTT
CCACTTAAATATGCATGGTAAATGTAAAAATTCAAAATCACGAAGACACTTCCTTATTTT
TAAACTAAAGATTATTTTTAATCGATTTCAAATAAATTTTTAATTTAATTCTTTCTTGAT
GTTTAGCAAACAATAAGGAACGCACTCGAAATGCAAGTGCTGCAAAGACATTATAAAATA
ATAAGTCTTAGTTTGTAATCATATTCCTAATCTCTAGAATAATAAAATCGATAAGAAAGT
TAAATTGAATACGAATGCTAGCCTATGCTTACTAGACATCCGATTTGCAATTATAGTTGC
CTTGATTTCAAAAAATACATCTCAGGTTCAGAAAAAAATCGAAATTACTTTCCCTAAAGTC
CTACCTTTACCATTGAAGAAGTGTAAACAATTAATTCAATCAATCATCACACATAAAGAA
GAGATAATTGAAAAAAAAAAAACCAAAACAAAACCTAAAAGCTTGAGTATAAGAAGGAGA
```

TATTTTAAAAGTGTTTTTCCTCTGACTTAGGATTAAAGAGAAAAAATTGTAGAACTCCAA
CTATTAAAATATAGAAATTCTGTCATCTCTGCTATTCTATGCATTAGCCCCTCATAAGTT
AGATAAAATACAATTAGATCAACAATTATTAAATATAAAGAACAAATTAAGATTACTAGA
ACTTAGAGTGATTGGTTGATAGAATAAGCTTCATTTTAATCAAATAGAAGTCTATTTAAC
GCTCGCTAATTCTTATCCGAATTCATTGAATAATTTCCAAATTAAAGAATATCTATTTAA
TAATTTCGTAAAATTTTACACGGTTTAAATATTAGGTATAGAATTCCTTCTCTCAAACCA
TTACAATTTTTTAATAAATATGGAAAATCTGAAGAAATGAACAATTTTGTAGCTAAGTTA
TGCGGATATGTATTTAAATAATATAAATTAATTTTTATTGATGAATGCTCTTTGGGAAAC
ATTTCAAAAGCTTCTCATAAATAATGGCATGTTGTTGGAACATTTAAAGAAATTACACAT
AGAATTTCAAATTTTAAATATCTAATAGGATCATTGGGAGATGATTGCTTTTTATAATAT
TAAATTTTTTCAGGCACTGGAAAGGGCTTTATATTTAGAGATTTTTTAGTTGAAGTAATT
AAAAAAGCTTAAGTTTTCTATTAGGAGAAACCCTTTGTTTTAATTTTAGATGGCTGCAGT
ATTCATAAATCAAGTTTAGTGAATGATATATTTGAAGAAATTCCTCACATTTATACTCCA
GCTTATAGACCAGAGTTTAATGCAATCGAACATATGTTTGGCTGGATTAAGAGAAAAATA
GTTTCTTTGTAACCAAGTATAACTTCATAATTCATTTATCAAATTCATTAAACAATTAAT
TAAATAGACGATAATTTAATGTAAAACTGGTGTGGAAGTGTTTATAGACCAATACTTAAT
GAATTATAATGGATTTGAACTTTTTAATTATTATAATAAACTAAATTTTTGATTAAATTT
AAAATATATATGAATGAAAAGTCAAATTAAGACGGCTCTGCACAGTTTGAGACTAACAAC
AATATTAATTCTTCAAAATTAAGGGATCTTTTAGACTAGTATGATTATTTTGCTATTTGC
AAAGTTTGTAAACTTCAGGTCTTGTTTACATTCGTAAATTAATTAGTAATCCATTACATA
GAGTGTTCATCTTTTTCTAATTAATCCAAAGAAAAAGGATTGATTGTTGAGACAAATTAA
AATGACTATTTAAATCCAGAGTTTGATTACAATACTCCATTTTTTATTTAGGAAATCAAT
CATACAAATTTTCTTTAATCAATTCTTAATGTTGGAAATTTGTCAATCAGCAGCAAGACT
TAGTATTAATCAGCATTATTGAAATTACTTAAGAATGTTGAATAGAAGGATTAAAACAAA
CTATGTGGATTGGATTTGATAAAATTAATGCAGGATTAATGCACAAATTTAACTTAAGTG
AATGCTGTTATTCGTTTATAAAATACAATGCATATCCCATAATCTTTAGAATTCAAGTAA
AAAGTGAGATTATAAAAAAAATTAATCAAAGAAAAAGTTGAAAAAAGGCCTCATCGTTCA
TGGGATTTGAATTACATTTAAGAAGTAATATTAAACTTATACTTAGTTACTAAACGAATT
GTTTGTTTACAATAGGGGATTTCTGGCCTTCAGACTCTTTATCCTATATGAGCTGGCTGC
ACGAATTTCAGAATTAGCATAAATTACTTTTTAAATGGTGGTTAATGGATTTATAAACTA
TATACCACAAAAATAAAAAAGACTTGGTAAAATTGCAGAAAGAAAGTTTCAAATAACAAC
AAGCGTGCAATTATAAACACAAGAGTTTGTAAAAAGAAATTCAATTTGTTTGTTATAAAC
TTTAGATATTGGATTACATAAGCAATCTATTAAATCATAATCTTTTCATAAATAATTGAA
ATATGCTTTAAAATTAATAGAAAATAGAAAGGTTAATCTTTCAAATGAGTATAAAGAGTT
CTTATAAAATTTTTAATATCATGATTTGAGATATTCAAAATTGCAAGAAATGAATAAAAG
TGGAATTGACGAGAAAATAATATAGACATTTGTGAAGCATTCCAATAGTAAAACAACATA
ATTGTATTTGTAAAATTAAAATATAAATGAAATCGTTAATTTTAATACGATAAACTAAAA
TAAATGCAAAGAAAAATTCTATGTATCACCAAGTCTTATTTTAAATTTTTTAGGTGAAAA
TGATTTTGAATGATATTATATATATGTTATAAGTGCATAATGAgTAAAATTTAATGAATT
AAAATGATTATACTAAAAATTCACATTCATTCAAATAAGTTCACATTCAAATGAATTATA
GGATGATAACTATTATGTTTTTAAGCGATAATATATTATTATAATGTATTTAAAATAGAA
GAGAATTAAAGACATAGCTGTTTGCTTAAAAAATATATAATTGATTTCATAATATAATAA
TTAAATTTATATTGAGTCAATATAATAAGCCTATTATATTGACTCAATATAATTGATTTG
TATTATGATAAAAATTAAACTGTGAAAAAGAATAGTTCTTTTTCACCATTCAATTAATTA
TATTGTGAAAAAGATCATTCTTTTTCACAATGCATTTTTGTAAAAAGAATTGAAGAATAA
TTTTTTGAATTTATGTATTAATTAATTAAATATTTGGTGAAAAAGATTAATTTCTTTTTc
ACCATAATATAAATTTTCTGTAGTATAAATCATTTATATTGAGTCAATATAATAGGCTTA
TTATATTGACTCAATATAAATTTAATTATTATATTATGAAATCAATTATATATTTTTTAA
GCAAACAGCTATA
>Sardine consensus DDE cds
ATGCAAGTGCTGCAAAGACATTATAAAATAATAAGTCTTAGTTTGTAATCATATTCCTAA
TCTCTAGAATAATAAAATCGATAAGAAAGTTAAATTGAATACGAATGCTAGCCTATGCTT
ACTAGACATCCGATTTGCAATTATAGTTGCCTTGATTTCAAAAAATACATCTCAGGTTCA
GAAAAAATCGAAATTACTTTCCCTAAAGTCCTACCTTTACCATTGAAGAAGTGTAAACAA
TTAATTCAATCAATCATCACACATAAAGAAGAGATAATTGAAAAAAAAAAAACCAAAACA
AAACCTAAAAGCTTGAGTATAAGAAGGAGATATTTTAAAAGTGTTTTTCCTCTGACTTAG
GATTAAAGAGAAAAAATTGTAGAACTCCAACTATTAAAATATAGAAATTCTGTCATCTCT
GCTATTCTATGCATTAGCCCCTCATAAGTTAGATAAAATACAATTAGATCAACAATTATT
AAATATAAAGAACAAATTAAGATTACTAGAACTTAGAGTGATTGGTTGATAGAATAAGCT
TCATTTTAATCAAATAGAAGTCTATTTAACGCTCGCTAATTCTTATCCGAATTCATTGAA
TAATTTCCAAATTAAAGAATATCTATTTAATAATTTCGTAAAATTTTACACGGTTTAAAT
ATTAGGTATAGAATTCCTTCTCTCAAACCATTACAATTTTTTAATAAATATGGAAAATCT
GAAGAAATGAACAATTTTGTAGCTAAGTTATGCGGATATGTATTTAAATAATATAAATTA
ATTTTTATTGATGAATGCTCTTTGGGAAACATTTCAAAAGCTTCTCATAAATAATGGCAT

GTTGTTGGAACATTTAAAGAAATTACACATAGAATTTCAAATTTTAAATATCTAATAGGA
TCATTGGGAGATGATTGCTTTTTATAATATTAAATTTTTTCAGGCACTGGAAAGGGCTTT
ATATTTAGAGATTTTTTAGTTGAAGTAATTAAAAAAGCTTAAGTTTTCTATTAGGAGAAA
CCCTTTGTTTTAATTTTAGATGGCTGCAGTATTCATAAATCAAGTTTAGTGAATGATATA
TTTGAAGAAATTCCTCACATTTATACTCCAGCTTATAGACCAGAGTTTAATGCAATCGAA
CATATGTTTGGCTGGATTAAGAGAAAAATAGTTTCTTTGTAACCAAGTATAACTTCATAA
TTCATTTATCAAATTCATTAAACAATTAATTAAATAGACGATAATTTAATGTAAAACTGG
TGTGGAAGTGTTTATAGACCAATACTTAATGAATTATAATGGATTTGA
>Sardine consensus DDE pep
MQVLQRHYKIISLSLQSYSQSLEQQNRQESQIEYECQPMLTRHPICNYSCLDFKKYISGS
EKIEITFPKVLPLPLKKCKQLIQSIITHKEEIIEKKKTKTKPKSLSIRRRYFKSVFPLTQ
DQREKIVELQLLKYRNSVISAILCISPSQVRQNTIRSTIIKYKEQIKITRTQSDWLIEQA
SFQSNRSLFNARQFLSEFIEQFPNQRISIQQFRKILHGLNIRYRIPSLKPLQFFNKYGKS
EEMNNFVAKLCGYVFKQYKLIFIDECSLGNISKASHKQWHVVGTFKEITHRISNFKYLIG
SLGDDCFLQYQIFSGTGKGFIFRDFLVEVIKKAQVFYQEKPFVLILDGCSIHKSSLVNDI
FEEIPHIYTPAYRPEFNAIEHMFGWIKRKIVSLQPSITSQFIYQIHQTINQIDDNLMQNW
CGSVYRPILNELQWI
>Sardine consensus Y-recombinase cds
ATGAATGAAAAGTCAAATTAAGACGGCTCTGCACAGTTTGAGACTAACAACAATATTAAT
TCTTCAAAATTAAGGGATCTTTTAGACTAGTATGATTATTTTGCTATTTGCAAAGTTTGT
AAACTTCAGGTCTTGTTTACATTCGTAAATTAATTAGTAATCCATTACATAGAGTGTTCA
TCTTTTTCTAATTAATCCAAAGAAAAAGGATTGATTGTTGAGACAAATTAAAATGACTAT
TTAAATCCAGAGTTTGATTACAATACTCCATTTTTTATTTAGGAAATCAATCATACAAAT
TTTCTTTAATCAATTCTTAATGTTGGAAATTTGTCAATCAGCAGCAAGACTTAGTATTAA
TCAGCATTATTGAAATTACTcAAGAATGTTGAATAGAAGGATTAAAACAAACTATGTGGA
TTGGATTTGATAAAATTAATGCAGGATTAATGCACAAATTTAACTTAAGTGAATGCTGTT
ATTCGTTTATAAAATACAATGCATATCCCATAATCTTTAGAATTCAAGTAAAAAGTGAGA
TTATAAAAAAAATTAATCAAAGAAAAAGTTGAAAAAAGGCCTCATCGTTCATGGGATTTG
AATTACATTTAAGAATTACTAAACGAATTGTTTGTTTACAATAGGGGATTTCTGGCCTTC
AGACTCTTTATCCTATATGAGCTGGCTGCACGAATTTCAGAATTAGCATAAATTACTTTT
TAAATGGTGGTTAATGGATTTATAAACTATATACCACAAAAATAAAAAAGACTTGGTAAA
ATTGCAGAAAGAAAGTTTCAAATAACAACAAGCGTGCAATTATAAACACAAGAGTTTGTA
AAAAGAAATTCAATTTGTTTGTTATAAACTTTAGATATTGGATTACATAAGCAATCTATT
AAATCATAATCTTTTCATAAATAATTGAAATATGCTTTAAAATTAATAGAAAATAGAAAG
GTTAATCTTTCAAATGAGTATAAAGAGTTCTTATAAAATTTTTAATATCATGATTTGAGA
TATTCAAAATTGCAAGAAATGAATAAAAGTGGAATTGACGAGAAAATAATATAGACATTT
GTGAAGCATTCCAATAGTAAAACAACATAATTGTATTTGTAAAATTAAAATATAAATGAA
ATCGTTAATTTTAATACGATAAACTAAAATAAATGCAAAGAAAAATTCTATGTATCACCA
AGTCTTATTTTAAATTTTTTAGGTGAAAATGATTTTGAATGA
>Sardine consensus Y-recombinase pep
MNEKSNQDGSAQFETNNNINSSKLRDLLDQYDYFAICKVCKLQVLFTFVNQLVIHYIECS
SFSNQSKEKGLIVETNQNDYLNPEFDYNTPFFIQEINHTNFLQSILNVGNLSISSKTQYQ
SALLKLLKNVEQKDQNKLCGLDLIKLMQDQCTNLTQVNAVIRLQNTMHIPQSLEFKQKVR
LQKKLIKEKVEKRPHRSWDLNYIQELLNELFVYNRGFLAFRLFILYELAARISELAQITF
QMVVNGFINYIPQKQKRLGKIAERKFQITTSVQLQTQEFVKRNSICLLQTLDIGLHKQSI
KSQSFHKQLKYALKLIENRKVNLSNEYKEFLQNFQYHDLRYSKLQEMNKSGIDEKIIQTF
VKHSNSKTTQLYLQNQNINEIVNFNTINQNKCKEKFYVSPSLILNFLGENDFE
>Sardine consensus ORF1 cds
ATGAATTAAAACAATTTATTTTTAAAACTTTAGTTTTTTAAATATGTTCCAAACACCTAC
AATTAATGTTTGATATGTAATGAATTAGTTTGCTTGTAAGAAACAAATCAATAATTTGAA
AAAAAAATAAATGAACACCTCCTATCCTTTAATCATCTTAAAGCACAAAAAGAAATTCTA
GACCAAACTGGATTCCTTTAGAACTAGACATCTATAAATCATGAGAGCGAAAGACAATCT
TATCCACTATATGATCTTCAAATTGCCAGAGAATTAAATTAAATTGGTCTAAATCTTTAC
TTTTAGAATTGCATATTAAAACTTGATAATTTTCTTTCTAAAAAGCAATCAACATAAAAG
GAATACGGGATTATCTTAAAAAGGTTTTATCATATGCTTTTTATCAATTAAACAAATTAA
ATTTCAGATTAAATTATAATAAAGTTTCTTTAGAATGAAGAGTATCGACATGATAGTATT
TCAGCACTAAGACTTTTAGCAAAATAAGCTGGCCATTAAATATCACTTTAGGTTTAAAAT
AATATTCAGATTGAAAAAAAAAAATTAATTAAAAAAGAATAAAAAAAAAAGCTCAATTTGAA
TTGCTTTGTTGTATATAGTTTTTAGAATTATTATTAAAAAATCTGAATTACAATTTGGCT
TTTCTTTTGCTTTTACTTTTTGAGTTGGATTTAGAACTAAGTTGTATTTCAATCCTTACA
TTTCAGGATGTTTTATCAGATAAAATCGAAATAAACAAAGAGAAGTTTGATAAATTAAGA
ACAAAAACGGTGCTTATTGGTAAATCATTATAATAGGCGGCAAAAATAACATTAAAAGTT
TTAGGACTACCTTTAAGTTCACCTATTTTTCCTTTAATTAGAAATTATAGAGATATACAA
AGTCTATTAGAAAAACTTAAAAAAGAATATAAGTTTTAACTTGAAGCATATAATGAGGAG

ACCAAATAATTTATATAGAATTTTCATCTTTCAGTTATAAAATTTATAAAGAAATCGATC
TACCCTTCAAAATAAATATAGTCATTTTTGGAAAGCTAAGTAGCAAATCATAAAAATAGC
AATAAATAATCTGATGCAGCTTTTCCAGCTCTATTTGAAATtTTCTTCGCGTATTTTGAA
TCCATAAAATAAAAATAAATAATTGGGAATTCAAAAGTTAATTCAATTATTAATAATAAT
GTTTTTAAAAAGAGGAGTAAATCTGAATTATAAATTATAAGAAATAGAAACATAGAATAA
ATGAGAGAAGCAACTtAAATTGTTGTACCTTTAGAGCAATTCTGTTAAGATTAACAGTAC
CAAAATCATGTATATAAACTAATTGAATAAATAGAATTCATTAATTGGCAATTAAGTCCT
TGA
>Sardine consensus ORF1 pep
MNQNNLFLKLQFFKYVPNTYNQCLICNELVCLQETNQQFEKKINEHLLSFNHLKAQKEIL
DQTGFLQNQTSINHESERQSYPLYDLQIARELNQIGLNLYFQNCILKLDNFLSKKQSTQK
EYGIILKRFYHMLFINQTNQISDQIIIKFLQNEEYRHDSISALRLLAKQAGHQISLQVQN
NIQIEKKNQLKKNKKKAQFELLCCIQFLELLLKNLNYNLAFLLLLLFELDLELSCISILT
FQDVLSDKIEINKEKFDKLRTKTVLIGKSLQQAAKITLKVLGLPLSSPIFPLIRNYRDIQ
SLLEKLKKEYKFQLEAYNEETKQFIQNFHLSVIKFIKKSIYPSKQIQSFLESQVANHKNS
NKQSDAAFPALFEIFFAYFESIKQKQIIGNSKVNSIINNNVFKKRSKSELQIIRNRNIEQ
MREATQIVVPLEQFCQDQQYQNHVYKLIEQIEFINWQLSP
>Sardine consensus ORF2 cds
ATGTAAAATATATTTTAAGAAGAAGAGAATGAATTCAAAATATCTCGAGCAGATGCTAAT
TAATTAATACCTTCCTTGGAATCAGCTTATTATGGATTGCTGATTTAAGGGAAATATTTA
CCAAAATTGACATCTTCGATTATTACATCAGAATATTTATTGGGTGTACTTTTTGAGAAA
ATCTATGTCCCTAATTTATCTGAAGTTTAAATTGGGGTACTACTTAAGGCAGTGAAAAAA
TACGAAATAATAGAGGAGGTCCAAAAAATACCAATATAATAAAAAACTTGGGGAATAGAT
TAAAAGCACCAACCTGACAAAGATTGGATTATAAACGTTTTAAAGACTTTAAAACCGAAT
CATTCTTTCTTTCAAAAATATAAAACCGATTCAAAGTATGATATGAAGATCTTCACTCAA
GATCAGATCAAAAAGATTAAAGAGTTAGATTTTTCAATGGGTAAGAAAACAAATGTTAAA
AGATTTTTTCGTATATCAAAAGATAAATAAATGGAAGCAGATAAACAAAAGAAAATTTTG
AAGAAGAAAACTTTAGAAAGTAAAATGGACAGAAAGCTAAAAATAATTGATGATTATGAA
AAAGAATTAAAGTTAATTGAAAACAAAATAAAGGATATCGAAGGGGAATGA
>Sardine consensus ORF2 pep
MQNIFQEEENEFKISRADANQLIPSLESAYYGLLIQGKYLPKLTSSIITSEYLLGVLFEK
IYVPNLSEVQIGVLLKAVKKYEIIEEVQKIPIQQKTWGIDQKHQPDKDWIINVLKTLKPN
HSFFQKYKTDSKYDMKIFTQDQIKKIKELDFSMGKKTNVKRFFRISKDKQMEADKQKKIL
KKKTLESKMDRKLKIIDDYEKELKLIENKIKDIEGE
>Thon (partial copy ; 5,262 nt with upstream TA)
TATAGCTGATTTCATCAGCAAAATATACTTAATATTACTACAATATATATAAATTCAATT
AAAATTTTGAATTAAAGTTAATATATTGATAATGTATTAAGTCAATAAAATTCAACTATT
TTATTGACTTAATATAAAGTTTCTATTGGTGAAAAGATAAATATCTTTCACGGTGAAAAG
AAAATTTTTCTTTTCACAATACTTTTTTACACAAAACCAAAGAACAATTTTTTGATTTGT
AAATATAATAATATAAAAATTATGGTGAAAAGAAAAAATTTCTTTTCACCAAATAATATG
CTTTTATAGAAACCAAAAATAACACATAGAAATGTGTTAATACTATATTTCTTCTATATA
TAATCAAAAATTaagatgtttgtaatgattcctgaacttttagaaaaataaaacgaatta
ttggtgaaaagaaatttttttcttTTCaCCATAATTtTtATATTatcAtATATaCAAATCA
AAAAATTGtTCTTTGTTTTtGtGTAAAAAAGTATTGTGAAAAGAAAAATtTTCTtTTCaC
cGTGAAAGATATTTATCTTTTCacCAATAGAAacCtTTATAtTAAGtTTGTGGAAGTGAT
TTTTTTCTCCTAATTATTTTTTTCTTATTCCTATTATTGAATGAAAATTTATTGATTGAG
AATCCATTTTCCATCATTATTGAATTTTTCCAAGTTTCAAGAGTCCATTTTTAGTTTTTG
CAAAATGGAAATGAATGTTGAAATTTATTTAATGGGAAAACTCACGATATCCTTTAGCTT
GTTTTATAATGATTTATCACCTATTCATCTAAATGATATTAAATTCAGTTCGATAATCAT
CAAACAATGATGCATTTATTATGTCGTAAATTTTTTTGTAATAATCCGATCATTAAAGCA
TTAATTTTAATAATGAATGATCAGAATATCAACAACAAATGTCAGTATTTTAAACAGAAT
ACAGAGACAGAATATAAATGTTTAATTTGCTTTAAAACAATGATCATTTAATAAAGGCCA
TCCATTGATTCATAATTTAATGATCATTTTTTATCCAAAGCGCATATGATTAGTTAATTT
TAAATTTTGCTTGAACTTGGGTATTTTGATCAAGTATCCTTTGCATGTGATTTGTAAATA
AATTCGGGCAAAGAAATACAATTATTTAAGTTATAGTAAGCAAAGAATCTCAAAAATTAA
AGCAAAGAATGTAAGATTTTACATATTTAGATCTGAATTGCTATTTTCAAAAATGCACGA
AACAAATTAATGGAATTGATCGAAAAATGTTGGCAAGTCAAAAAAACATATCGCATAATA
CTTAAACGCTTTTATAATTTTTTATTTGAATTGGAAGCGGATTCTTATGAAGAGGATTTG
GTCTTACGATTTTTATAAAGAGAAAAATTTAAACAGAATAGTATATCTGCTATGCAATTC
CTTTCAAAAGCTATTTGTAAATGTCCTTAAAACTATTTAGGCTAAGCCTTATCTGGTGAA
TTCATTGATAAAAATTAAAAACTAAAAGTAGATTAATAAACTTAAAATAGAGAAAAGGCC
CAAGTTAAGTATAAATTGTGTAAATAACTTATGGCGTTTCTATTCAACAATTCATAAGAA
AGATTAGTCTTTCTGATATTTATATTGTTTTAATTTGAAATTGATCTAAAAGTTATCCCG
CAACTTAAGTATTAGGATATCTTGAATAATACTATTCAGAAGTCTACAAATAATGGTTAA

AAGTCAAGTGAATAGATTTTGAAAATAAATAACTCGATATGTTAATTGATAATATAATTT
TTGGAAAAATCTAAACTCGATACGACTACTTTTGTGTTACCTGAGGTTCGCAGGATAAGA
GATTTGTAATCGCTTCTATGTTCAATTAAAACAGAATTTGTTTAAAAATAGAGAAGCACA
ATGACACGCTTGAAGAATTTATCTAAAACTTCACCTTTTCAAAGATAAAATTTATAAAAA
ACTATTAATTAGTGAGAAAAACAAGTAGACTGATAAGTAAAAGAATATATCTATTAATAA
AGCGAACAGTTTATGGTAGTACCATTCCCCGATTTATTAAAACTGTTTATATTTTTGTTT
GAAGAAGAAAAAACTCAGTGCTAGACTTAAAATATTTTAAACTCAAACTCAACTTAATAA
AAAGCTAAGAGAAGCAAATCTTAGTTGGAATTATAACGATAAAAAAACAAGGTCCAAATG
ATTTAAATTTCTAATCATATTATCGTTTTGGATGATAGCGATAATGAAAATAAATTCATG
TATGTATTAGCTTTATTCTTTTATAGTCCTATCAACTCTTAAACATAAGGAACTTTTCAT
TAGCCTAATTTACATTCAGAAAATGAAATTCAATCAATTTCTCAAAGATAAAATAATTTG
TAAACGACTGATTAAGATTGAGCATAATTTAAGGAAGGAAATATTTATGAATTTAAAAAA
CTTTATTATCATTTGTTCATGACCATAAAGGCTGTTTCAATTACCAGTAATATCCTTAAT
CCTATCTTCGATAAATTAAATATCTTTTTCACATTCCTCAATAAGTTTGTGTTTGCGATC
CATCTTAACCTCTAAATTTTTTTtCTTCAAATTCTTTCTAAATTATTCTGCTTCTTATTG
TTTCTCTTTAAAAATTCTGAAGAATCTTTTCACACTTGTCTTTTTCCCCATAAATCCAAT
TCTTTTATTTTACTAAAATTTTTCAAATACTATTACTTGTATTATTCTAAGGCAAGCAAT
TTCATATCAAAATTTGACTCTTATTTGTACTTTTAAAAGAAAATATGATTTGGTTTAAGT
GTTTTTAACACATTAATAATCCAGTTCTTATCAGGAGTTCTTTTGGATTCAATTCCCCAA
ATTTATTATTGAAGAGGAATCTTAATTAATTCTTGTATTAATTCATGCTTTGTTACAGAC
TTTAGCAACACACCTATTTAGACTTCATCTAATTTGGTCGCATATATTTTATCAAATAAA
ATGCCTAATAAGTATTCCGACGTTATAATAGATGAAGTCATCTTTGGCAGATATTTTCCT
TACATCAACAATCCAAAATATGCTGATTCTAGTGATGGTATTAATTAATTGGCGTCTTAC
CTTGTTAATTTCATATATTCCTCTTCCTAAAGAATATTTTGCATAATATTTGAAATAATT
ATATATAAAATCTTGATTTTCAGCATTTTGTTAAAGTGTAATTAATTGGAAATATAAAAA
ATGTAAAAATTTGTGATTGACGAAGCTGCTACGTATTTTTTATATAAAACATTATTTCAC
CAAATTCAAGAATTATTTTATTTATTATGAATTTGGATATCAATAATCATGCAAAACAAT
AAGATTCAATTTCTAAGCATAAATTTACAAAATAATAAGCTTTGAAATGTATATTATATT
GAAATATTGGTAGCATAAAGAATAGAAGAAGAATCTACTATTGTGTATGAATGTTAACCC
ATACTTGGGTTTCATCCTACTTGTAATGAATAATGCTTAACTTTTGTTCAAAAGATAGCA
AATTAAGAAAAAATGAACGATTTTAGAATTGAAAAGCTCTAATTCTCATCACTAATCTAG
AGTAGGACTGTATTAAAAAAGTTTGTTGTTAAACAATATTAAGAAAAAGACATGAAACAA
ACTAAATTATTGTTGAAAAGGAAAAATTAAATTTGCAAGAAGAGAGCTACTCCATTAACA
TTTGATAAGCGAAAGGATATCTAGGAATTATAAATGAAACAATATAAAAATTCAGTAATA
TCAGAGATCCTAGGAATTAGCACATAATAAGTGAGACAAAATCATATTAGACTTTAAATA
AGTAAATCAAAAGTAGAAATCAAAATTACAAAAAGGTAGCAAAATTGGTTGATAGAAAAT
GGTTCATAATAATCAAATTCGATATTATTTAATGCTCGATTTTTTCTAGCAGAATTTTAA
ACTTAATTTCCAAAGCAACAAATTACAATCTCTCAGTTTCGAAAGGTTCTACATTAGTGC
AATTTAAAATATAGAATTCCTTCTTTGAAACCATTCAGTTTTTTTTATAAAGCTGGAAAA
GATGAAGAAACAAAAATTTATATCTCAAATTTGTAGTTATCTCATAAATTCATATAAAAT
TGTGTTTATTGATGAATGTTCATTAGGGAATATATCCAAATCTGCCCATAAATAGTGGCA
TGTTTTAGGAACTTTCAAAGAGTCTACTTAAAGGTTATCGAGTTTTAAATACTTTATCGG
AGCATTAGGTGAGGATAACTTTTTCTAATATTAACTGTTTTCTGGGACTGGTAAAGCTTA
CATATTTCAGGACTTTTTAATATCAGTTATTAACAAAGCTCAAAAACACTATAAAAATCA
ACCGTTTGTCTTAATTATGGATAATTGTAGTATCCATAAATCAAAATAAATTGTAGAAAT
CTTTGATAAAATTCCTTGTATTTTTACACCTGCTTATAGGCCAGAATTTAATGCGATTGA
GCATATGTTTGGATGGTTAAAGAGAGGTCTAGTTGTATCTTAGCCAACTTCCACAAACCA
ATTTATATATTAAATCCATAGAATTATGAATTCTATTACAGAGAATTTAATGTAAAATTG
GTGTTAGAGTATTTACAAACCATTGCTTAAAGAATTGGATTATTTATGAGTACATTTTAA
AAAGAAATTATTTAATTACAATTAACTATTTTTATTATGATTCAATAATTAAAATGCATT
TATCAACCAAATAAGAACAAGTTTCAAGAAATCTTAATTCTGAATTGATTTTCGACAATG
TACTAAATTCTTATAAGTACAATACTATTTGCAAAAAGTGTAATCAAGAAATTCCTTTAA
CAGAGATAAATCATTTGATCATTCATTAAATTGATTGTCAATAGAATGCTCATTGTATTT
CCCAAGATAATTAAAtAAGCGAATAaaTTCaaCCCTGAACCaTTGAGAAATCGatGGGTC
tTGAAAATTGGCAAGCTACTCAgTGTCaAGAAAAAaaaaaataaAAAGTAATTTAATAAC
TTCTTGATATTGGAAAGTTATCAAAAGAATGTtAAAATCTCTAAAGACCTGCATTAATAA
AGTTATTTAAAAATTTCGATGATTGTAATCTTCTCAATTTGTAGGCTTAACAACTAAAAA
ATATTGTGAAATATTAATGTAAAAGTACAAATTAAGTAATTAAACAGTAAAAGAAAATTT
TAAAAGAAGGGATTCTCAAACGACCTCATCGATCATGGGATC
>Thon DDE cds
ATGAATTtGGATATCAATAATCATGCAAAaCAATAAGATTCAATtTCTAAGCATAAATtT
ACAAAATAATAAGCTtTGAAATCATAAAGAATAGAAGAAGAATCTACTATTGTGTATGAA
TGTTAACCCATACTTGGGTTTCATCCTACTTGTAATGAATAATGCTTAACTTTTGTTCAA
AAGATAGCAAATTAAGAAAAAATGAACGATTTTAGAATTGAAAAGCTCTAATTCTCATCA

CTAATCTAGAGTAGGACTGTATTAAAAAAGTTTGTTGTTAAACAATATTAAGAAAAAGAC
ATGAAACAAACTAAATTATTGTTGAAAAGGAAAAATTAAATTTGCAAGAAGAGAGCTACT
CCATTAACATTTGATAAGCGAAAGGATATCTAGGAATTATAAATGAAACAATATAAAAAT
TCAGTAATATCAGAGATCCTAGGAATTAGCACATAATAAGTGAGACAAAATCATATTAGA
CTTTAAATAAGTAAATCAAAAGTAGAAATCAAAATTACAAAAAGGTAGCAAAATTGGTTG
ATAGAAAATGGTTCATAATAATCAAATTCGATATTATTTAATGCTCGATcTTTTCTAGCA
GAATTTTAAACTTAATTTCCAAAGCAACAAATTACAATCTCTCAGTTTCGAAAGGTTCTA
CATTAGTGCAATTTAAAATATAGAATTCCTTCTTTGAAACCATTCAGTTTTTTTATAAA
GCTGGAAAAGATGAAGAAACAAAaTTTATATCTCAAATTTGTAGTTATCTCATAAATTCA
TATAAAATTGTGTTTATTGATGAATGTTCATTAGGGAATATATCCAAATCTGCCCATAAA
TAGTGGCATGTTTTAGGAACTTTCAAAGAGTCTACTTAAAGGTTATCGAGTTTTAAATAC
TTTATCGGAGCATTAGGTGAGGATAACTTTTTCTAATATTAACTGTTTTCTGGGACTGGT
AAAGCTTACATATTTCAGGACTTTTTAATATCAGTTATTAACAAAGCTCAAAAACACTAT
AAAAATCAACCGTTTGTCTTAATTATGGATAATTGTAGTATCCATAAATCAAAATAAATT
GTAGAAATCTTTGATAAAATTCCTTGTATTTTTACACCTGCTTATAGGCCAGAATTTAAT
GCGATTGAGCATATGTTTGGATGGTTAAAGAGAGGTCTAGTTGTATCTTAGCCAACTTCC
ACAAACCAATTTATATATTAAATCCATAGAATTATGAATTCTATTACAGAGAATTTAATG
TAAAATTGGTGTTAGAGTATTTACAAACCATTGCTTAAAGAATTGGATTATTTATGA
>Thon DDE pep
MNLDINNHAKQQDSISKHKFTKQQALKSQRIEEESTIVYECQPILGFHPTCNEQCLTFVQ
KIANQEKMNDFRIEKLQFSSLIQSRTVLKKFVVKQYQEKDMKQTKLLLKRKNQICKKRAT
PLTFDKRKDIQELQMKQYKNSVISEILGISTQQVRQNHIRLQISKSKVEIKITKRQQNWL
IENGSQQSNSILFNARSFLAEFQTQFPKQQITISQFRKVLHQCNLKYRIPSLKPFSFFYK
AGKDEETKFISQICSYLINSYKIVFIDECSLGNISKSAHKQWHVLGTFKESTQRLSSFKY
FIGALGEDNFFQYQLFSGTGKAYIFQDFLISVINKAQKHYKNQPFVLIMDNCSIHKSKQI
VEIFDKIPCIFTPAYRPEFNAIEHMFGWLKRGLVVSQPTSTNQFIYQIHRIMNSITENLM
QNWCQSIYKPLLKELDYL
>Thon Y-recombinase cds (partial)
ATGCATTTATCAACCAAATAAGAACAAGTTTCAAGAAATCTTAATTCTGAATTGATTTTC
GACAATGTACTAAATTCTTATAAGTACAATACTATTTGCAAAAAGTGTAATCAAGAAATT
CCTTTAACAGAGATAAATCATTTGATCATTCATTAAATTGATTGTCAATAGAATGCTCAT
TGTATTTCCCAAGATAATTAAAtAAGCGAATAaaTTCaaCCCTGAACCaTTGAGAAATCG
atGGGTCtTGAAAATTGGCAAGCTACTCAgTGTCaAGAAAAAaaaaaataaAAAGTAATT
TAATAACTTCTTGATATTGGAAAGTTATCAAAAGAATGTtAAAATCTCTAAAGACCTGCA
TTAATAAAGTTATTTAAAAATTTCGATGATTGTAATCTTCTCAATTTGTAGGCTTAACAA
CTAAAAAATATTGTGAAATATTAATGTAAAAGTACAAATTAAGTAATTAAACAGTAAAAG
AAAATTTTAAAAGAAGGGATTCTCAAACGACCTCATCGATCATGGGATC
>Thon Y-recombinase pep (partial)
MHLSTKQEQVSRNLNSELIFDNVLNSYKYNTICKKCNQEIPLTEINHLIIHQIDCQQNAH
CISQDNQISEQIQP*TIEKSMGLENWQATQCQEKKKQKVIQQLLDIGKLSKECQNLQRPA
LIKLFKNFDDCNLLNLQAQQLKNIVKYQCKSTNQVIKQQKKILKEGILKRPHRSWD
>Thon ORF1 cds (putative -1 frameshift at position 973)
ATGAATGATCAGAATATCAACAACAAATGTCAGTATTTTAAACAGAATACAGAGACAGAA
TATAAATGTTTAATTTGCTTTAAAACAATGATCATTTAATAAAGGCCATCCATTGATTCA
TAATTTAATGATCATTTTTTATCCAAAGCGCATATGATTAGTTAATTTTAAATTTTGCTT
GAACTTGGGTATTTTGATCAAGTATCCTTTGCATGTGATTTGTAAATAAATTCGGGCAAA
GAAATACAATTATTTAAGTTATAGTAAGCAAAGAATCTCAAAAATTAAAGCAAAGAATGT
AAGATTTTACATATTTAGATCTaAATTGCTATTTTCAAAAATGCACGAAACAAATTAATG
GAATTGATCGAAAAATGTTGGCAAGTCAAAAAAACATATCGCATAATACTTAAACGCTTT
TATAATTTTTTATTTGAATTGGAAGCGGATTCTTATGAAGAGGATTTGGTCTTACGATTT
TTATAAAGAGAAAAATTTAAACAGAATAGTATATCTGCTATGCAATTCCTTTCAAAAGCT
ATTTGCTAAGCCTTATCTGGTGAATTCATTGATAAAAATTAAAAACTAAAAGTAGATTAA
TAAACTTAAAATAGAGAAAAGGCCCAAGTTAAGTATAAATTGTGTAAATAACTTATGGCG
TTTCTATTCAACAATTCATAAGAAAGATTAGTCTTTCTGATATTTATATTGTTTTAATTT
GAAATTGATCTAAAAGTTATCCCGCAACTTAAGTATTAGGATATCTTGAATAATACTATT
CAGAAGTCTACAAATAATGGTTAAAAGTCAAGTGAATAGATTTTGAAAATAAATAACTCG
ATATGTTAATTGATAATATAATTTTTGGAAAAATCTAAACTCGATACGACTACTTTTGTG
TTACCTGAGGTTCGCAGGATAAGAGATTTGTAATCGCTTCTATGTTCAATTAAAACAGAA
TTTGTTTAAAAATAGAGAAGCACAATGACACGCTTGAAGAATTTATCTAAAACTTCACCT
TTTCAAAGATAAAATTTATAAAAAACTATTAATTAGTGAGAAAAACAAGTAGACTGATAA
GTAAAAGAATATATCTATTAATAAAGCGAACAGTTTATGGTAGTACCATTCCCCGATTTA
TTAAAACTGTTTATATTTTTGTTTGA
>Thon ORF1 pep (putative -1 frameshift corrected)
MNDQNINNKCQYFKQNTETEYKCLICFKTMIIQQRPSIDSQFNDHFLSKAHMISQFQILL

ELGYFDQVSFACDLQINSGKEIQLFKLQQAKNLKNQSKECKILHIQIQIAIFKNARNKLM
ELIEKCWQVKKTYRIILKRFYNFLFELEADSYEEDLVLRFLQREKFKQNSISAMQFLSKA
ICQALSGEFIDKNQKLKVDQQTQNREKAQVKYKLCKQLMAFLFNNSQERLVFLIFILFQF
EIDLKVIPQLKYQDILNNTIQKSTNNGQKSSEQILKINNSICQLIIQFLEKSKLDTTTFV
LPEVRRIRDLQSLLCSIKTEFVQKIEKHNDTLEEFIQNFTFSKIKFIKNYQLVRKTSRLI
SKRIYLLIKRTVYGSTIPRFIKTVYIFV
>Thon ORF2 cds
ATGCAAAATATTCTTTAGGAAGAGGAATATATGAAATTAACAAGGTAAGACGCCAATTAA
TTAATACCATCACTAGAATCAGCATATTTTGGATTGTTGATGTAAGGAAAATATCTGCCA
AAGATGACTTCATCTATTATAACGTCGGAATACTTATTAGGCATTTTATTTGATAAAATA
TATGCGcCCAAATTAGATGAAGTCTAAATAGGTGTGTTGCTAAAGTCTGTAACAAAGCAT
GAATTAATACAAGAATTAATTAAGATTCCTCTTCAATAATAAATTTGGGGAATTGAATCC
AAAAGAACTCCTGATAAGAACTGGATTATTAATGTGTTAAAAACACTTAAACCAAATCAT
ATTTTCTTTTAAAAGTACAAATAAGAGTCAAATTTTGATATGAAATTGCTTGCCTTAGAA
TAATACAATAAAATAAAAGAATTGGATTTtATGGGGAAAAAGACAAGTGTGAAAAGATTC
TTCAGAATTTTTAAAGAGAAACAATAAGAAGCAGAATAATTTAGAAAGAATTTGAAGAAA
AAAAaTTTAGAGGTTAAGATGGATCGCAAACACAAACTTATTGAGGAATGTGAAaAAGAT
ATTTAATTTATCGaAGATAGgATTaAGgATATTACTGGTAATTGA
>Thon ORF2 pep
MQNILQEEEYMKLTRQDANQLIPSLESAYFGLLMQGKYLPKMTSSIITSEYLLGILFDKI
YAPKLDEVQIGVLLKSVTKHELIQELIKIPLQQQIWGIESKRTPDKNWIINVLKTLKPNH
IFFQKYKQESNFDMKLLALEQYNKIKELDFMGKKTSVKRFFRIFKEKQQEAEQFRKNLKK
KNLEVKMDRKHKLIEECEKDIQFIEDRIKDITGN
>AnchoisA consensus (3,599 nt with duplicated TAs)
TATAGTCCAAAATTATTGCAGATTTAAGTTGCTTTGAGTTGCAAAAATTAACTTTTTTTT
GCTGAAGCAGAAAGAAACAAGAAATAAATTGATATATTATGAATAAGAATTTTTAATATT
TTTTTCCCAATGTTGATTCTCTCCCTAAATTTGCATTAATATATTTCCTGCTGTTAATTT
GCTGTTATAATAGTTTATAAATATTCAGATAAAATAAAAAAAATAAATTAGTGCAATTTA
ATTGCAGAAAATATTTTCCAAGAGAATTTAAATTAGATAAAATCTGCCTGGCTATTTTTC
GTTTTTCAATATTATCATCAAAATAAATGTCTGAAAAGGTTTCCAGCTAATCTATAACTT
GCAAACATTGCTCAACTTCATTATTTGGACAATCGAATATCAAGCTTCATCAATTTGTTT
GCGTTGAACAAATTTAGAAAAATATGGAAGAAGCTTTAAATATTGGAGGAGGGAAAAATC
AACGAAGAAGAAACAAAAAATGCATTGAAAGAATTTTACAGCAGGGTTTGGAAAACTTCC
AATAGCACTAAATCAAAAGAAATCAACAAAGTGCACAAAGAAATTCGAAATTAGATATCA
AAATTAGCGACCAAAATTTTAAGCAACAGCAAATAAATATTTTAGAGAAAAAGGAATAGC
AAGAAAGCAATTAGAAAGATAAACAATATGAAGAAATATTGTCAGAGAGCAAGCAAGAAT
TTGAGCAGAACATTATTTTAAGAAAAGTGAAGAGAGTGGTAGAGAAGTGGAAGTGCTAAA
GATGTATAAAAGAGTTCATGAGGGAGCAGGATGGGTATCGGCACTTTGAGTTCCATCATA
AGGATTTGAATGAAGAAGAGAAAAAGTTCTTTGACTCGAGAAAGAAGAAAAAAGGTGCTG
AAGCAGAATAGTAGCAGGATGAAAAATGAGATACCATAAAATATATTAAAAAATGCATGA
ATAATTTTCTACTTCTTTTTCATAATAATCAATTTCTTCTTTAGAATTGCTGTCTCTATT
CTACTTCCTCTTAAATTCCTTAATTCACTATTTCTAATTCTTCAGTTAACTTTGATAATT
TTAGCAACTTTTTTGCTTAAATGAGTTCTTTGCGAGCTAACTTTTTCTTAGCTGCATTTT
ATTTAAATATCTCAACTTACTCCTTTGTTCTGCGAAAATATCTCAGCCTTTTATTTCTTC
CGATAGAAGTGTCTAATTAGCGAATCAACCTTATGTTGATAAATATTTTACATTAAATCA
TCCTGCGTGAGATTTGATTAATCCTGCACTTTAGGgtTTTAAATAAAGAATGATTTGGTT
CTATTGTTTTAAGAACATTAACTGCCCATGTTTTATCAGGGTAGTTTCCATCAGAAAAGC
CAAGCTCTTAACCATTAAGCTTCTTTTTAATTTCATGTAAAATTTCTTCTTTAGTAGCTG
ATCTCAGCAGTTATCCTATTTGAACTTTTTTTGATTCTAAGCCAAAGCATTATTTAGTAG
CAACTTTGAGCAAATATTACGCTGTTACACCTGAAGAATGAATTGCAGGTAAATAATATC
CTGCCAAGATTAAGCCATAGTACATAGATTCAACAGAACCAGTTGCTTGATTAGCTTAGA
AAATGGAAATCATGATGAATTTCGAAAGTTTGTTTAATTATTCAAACAATGCGATAGTAA
ATTTTTATTGAAAAAAAAATTCTTTTTTAACCAATCAATTTCAAAGCAATTGATTTTAAA
AGCAGAAAACAATTTAGAATAAATTAAAGAAAAATATTAAATGATTTATAATTTTAATGC
AACTTAAATACTGCTTTTTTATTGAATTATATTTATAATGAGGATTTTTTGTTTTGAATT
GGAGAAAATATTTTGATTCAATTTTGATTATTGATTCGAATTTTAGATTATAGCTCCAAT
TTTTATGCAAGAAATTTAATTTTTCCACATTTAATGTAAATAGAAACTCAAGCTTCTTCA
TCTCTGTACATATATTATTTGCTATAGCAGGCCATATGCTTAAACAACAATATGTAGACA
CATATGCGATGATCATTGCTAATAATTTTACTAAACCTATGTTTATGAAAAAAGACTTTT
AATCAAAACCAAATAGCAAATGCTGGAACTGCTTACTCTAGAGGATGCTCATAAAATTAT
AAAACAAAAATTAGAATCCACAAGGGAAGTAAAGTTGAGAAAAAAATATAAACAAATTTGA
GAAAAAAAGCAACTTCAAATCATTAACTTTAAAATTGCAGAAAATTGCTTTAGAATTAGG
AAATTCGGTAAAACCTGCAATTTTGGCAGGATATTTAGGAATTTCTATTGGAAGAGCTAA
ATATTTATAAAAGTATGTTtTACAGCAACCTGATTTACAAAGACAATAACTTGCTTTTAA

ATTTGATGCATAACATAAATTTTGGATTTAAAAATTTGTAGCTCAAGAAAATGTTGCTCG
CTTGAGCAGTGCAAAATAAATCTATTAAGAATTTATAGCAAATTTTCATATTTGCAAATA
GCAACTGCAGTTTCATTAATTTTTTAAGTAGTTTAAACAGCTTTGGTTATATACCTATCG
CTTATTTAGTTGCGTTAATTTTAAATCTCAAACTAAATGTTCTTTAGACAGAAGGTTTGA
ATTTGCaAAATAATTAGCTACTCTTTTAAATTCAAAATAAAAAATTGTTTACATTGATGA
GTGCAGTTTTGGGAGAAATTTAAAAGTTGCAAGAGGTTGGTAAAAAAAGAACACTTTTCC
ATTATTGCAAATTAAATCAAGCTCATCAAAAAATAAATGTGTTATTGGAGCTATAGCTTG
TGACGGGTTTATTGCTTACAAATGTGTTATAGGAAATGTTAATCAATAGGTTTTTGGAGA
ATTTTTGAATGAACTTCTAAGGCTGCTTTAGCAGAATAGTGAAGAAAATAATTTTTGCTT
GGTGCTGGACAATGCATCTATCCATCGCACATCACAAATTTTGGATTAATTGTCTTATGT
GAACTATTTATTTCTCCCACCATATAGTCCTTAGCTTAATTGCATTGAAAAGCTATGGGG
AGTAGCAAAACAAAAGCTCTCCAAAATGCACTTCGCAACAACATTTTAAGAATTTCACAA
CATTCATTTTGTGCTTAACTCAATTACAGCTAATTAAGTGCAAAATATTTGCACCTTTAA
CTTGAAATTCTTGCAAGCTTGCTTACATAGACAGCAGTTTTGATTAATTATATTTTCATT
TAAACCATAAAATATTCAATGCAAGCAATTAACAATAAAAATTCTCTACTCTTTTCTTAT
AGCGACAGTGTGCAAAAGCAGACTTTCGTCAGTTATACTTAACACTATCGAGCAGATATT
AATAAGAGCAGAGTTTCGAATCGGGGGACAGGATGGGATCTAAAACACTCGCTCCTTAAC
CTTTCCAGCCTATTCTCAATACTATTAGACTCTGATTTAATTATATATTTTAAAATTTGA
TAAAATTAAATTATGGGTTTGGGGTAATTCATTAAATATTATATAATTATATATAATTTA
TATTATTATAAATTTATATAATCCTGCAAAAGCAGAAAGTCAAATTGTATTTGACTATA
>AnchoisA consensus DDE cds
ATGTAAATAGAAACTCAAGCTTCTTCATCTCTCAGGCCATATGCTTAAACAACAATATGT
AGACACATATGCGATGATCATTGCTAATAATTTTACTAAACCTATGTTTATGAAAAAAGA
CTTTTAATCAAAACCAAATAGCAAATGCTGGAACTGCTTACTCTAGAGGATGCTCATAAA
ATTATAAAACAAAAATTAGAATCCACAAGGGAAGTAAAGTTGAGAAAAAATATAAACAAA
TTTGAGAAAAAAAGCAACTTCAAATCATTAACTTTAAAATTGCAGAAAATTGCTTTAGAA
TTAGGAAATTCGGTAAAACCTGCAATTTTGGCAGGATATTTAGGAATTTCTATTGGAAGA
GCTAAATATTTATAAAAGTATGTTtTACAGCAACCTGATTTACAAAGACAATAACTTGCT
TTTAAATTTGATGCATAACATAAATTTTGGATTTAAAAATTTGTAGCTCAAGAAAATGTT
GCTCGCTTGAGCAGTGCAAAATAAATCTATTAAGAATTTATAGCAAATTTTCATATTTGC
AAATAGCAACTGCAGTTTCATTAATTTTTTAAGTAGTTTAAACAGCTTTGGTTATATACC
TATCGCTTATTTAGTTGCGTTAATTTTAAATCTCAAACTAAATGTTCTTTAGACAGAAGG
TTTGAATTTGCaAAATAATTAGCTACTCTTTTAAATTCAAAATAAAAAATTGTTTACATT
GATGAGTGCAGTTTTGGGAGAAATTTAAAAGTTGCAAGAGGTTGGTAAAAAAAGAACACT
TTTCCATTATTGCAAATTAAATCAAGCTCATCAAAAAATAAATGTGTTATTGGAGCTATA
GCTTGTGACGGGTTTATTGCTTACAAATGTGTTATAGGAAATGTTAATCAATAGGTTTTT
GGAGAATTTTTGAATGAACTTCTAAGGCTGCTTTAGCAGAATAGTGAAGAAAATAATTTT
TGCTTGGTGCTGGACAATGCATCTATCCATCGCACATCACAAATTTTGGATTAATTGTCT
TATGTGAACTATTTATTTCTCCCACCATATAGTCCTTAGCTTAATTGCATTGAAAAGCTA
TGGGGAGTAGCAAAACAAAAGCTCTCCAAAATGCACTTCGCAACAACATTTTAAGAATTT
CACAACATTCATTTTGTGCTTAACTCAATTACAGCTAATTAAGTGCAAAATATTTGCACC
TTTAACTTGAAATTCTTGCAAGCTTGCTTACATAGACAGCAGTTTTGA
>AnchoisA consensus DDE pep
MQIETQASSSLRPYAQTTICRHICDDHCQQFYQTYVYEKRLLIKTKQQMLELLTLEDAHK
IIKQKLESTREVKLRKNINKFEKKSNFKSLTLKLQKIALELGNSVKPAILAGYLGISIGR
AKYLQKYVLQQPDLQRQQLAFKFDAQHKFWIQKFVAQENVARLSSAKQIYQEFIANFHIC
KQQLQFHQFFKQFKQLWLYTYRLFSCVNFKSQTKCSLDRRFEFAKQLATLLNSKQKIVYI
DECSFGRNLKVARGWQKKNTFPLLQIKSSSSKNKCVIGAIACDGFIAYKCVIGNVNQQVF
GEFLNELLRLLQQNSEENNFCLVLDNASIHRTSQILDQLSYVNYLFLPPYSPQLNCIEKL
WGVAKQKLSKMHFATTFQEFHNIHFVLNSITANQVQNICTFNLKFLQACLHRQQF
>AnchoisA consensus ORF1 cds
ATGTCTGAAAAGGTTTCCAGCTAATCTATAACTTGCAAACATTGCTCAACTTCATTATTT
GGACAATCGAATATCAAGCTTCATCAATTTGTTTGCGTTGAACAAATTTAGAAAAATATG
GAAGAAGCTTTAAATATTGGAGGAGGGAAAAATCAACGAAGAAGAAACAAAAAATGCATT
GAAAGAATTTTACAGCAGGGTTTGGAAAACTTCCAATAGCACTAAATCAAAAGAAATCAA
CAAAGTGCACAAAGAAATTCGAAATTAGATATCAAAATTAGCGACCAAAATTTTAAGCAA
CAGCAAATAAATATTTTAGAGAAAAAGGAATAGCAAGAAAGCAATTAGAAAGATAAACAA
TATGAAGAAATATTGTCAGAGAGCAAGCAAGAATTTGAGCAGAACATTATTTTAAGAAAA
GTGAAGAGAGTGGTAGAGAAGTGGAAGTGCTAAAGATGTATAAAAGAGTTCATGAGGGAG
CAGGATGGGTATCGGCACTTTGAGTTCCATCATAAGGATTTGAATGAAGAAGAGAAAAAG
TTCTTTGACTCGAGAAAGAAGAAAAAAGGTGCTGAAGCAGAATAGTAGCAGGATGAAAAA
TGA
>AnchoisA consensus ORF1 pep
MSEKVSSQSITCKHCSTSLFGQSNIKLHQFVCVEQIQKNMEEALNIGGGKNQRRRNKKCI

ERILQQGLENFQQHQIKRNQQSAQRNSKLDIKISDQNFKQQQINILEKKEQQESNQKDKQ
YEEILSESKQEFEQNIILRKVKRVVEKWKCQRCIKEFMREQDGYRHFEFHHKDLNEEEKK
FFDSRKKKKGAEAEQQQDEK
>AnchoisA consensus ORF2 cds
ATGATTTCCATTTTCTAAGCTAATCAAGCAACTGGTTCTGTTGAATCTATGTACTATGGC
TTAATCTTGGCAGGATATTATTTACCTGCAATTCATTCTTCAGGTGTAACAGCGTAATAT
TTGCTCAAAGTTGCTACTAAATAATGCTTTGGCTTAGAATCAAAAAAAGTTCAAATAGGA
TAACTGCTGAGATCAGCTACTAAAGAAGAAATTTTACATGAAATTAAAAAGAAGCTTAAT
GGTTAAGAGCTTGGCTTTTCTGATGGAAACTACCCTGATAAAACATGGGCAGTTAATGTT
CTTAAAACAATAGAACCAAATCATTCTTTATTTAAAacCCTAAAGTGCAGGATTAATCAA
ATCTCACGCAGGATGATTTAATGTAAAATATTTATCAACATAAGGTTGATTCGCTAATTA
GACACTTCTATCGGAAGAAATAAAAGGCTGAGATATTTTCGCAGAACAAAGGAGTAAGTT
GAGATATTTAAATAAAATGCAGCTAAGAAAAAGTTAGCTCGCAAAGAACTCATTTAAGCA
AAAAAGTTGCTAAAATTATCAAAGTTAACTGAAGAATTAGAAATAGTGAATTAAGGAATT
TAAGAGGAAGTAGAATAGAGACAGCAATTCTAAAGAAGAAATTGA
>AnchoisA consensus ORF2 pep
MISIFQANQATGSVESMYYGLILAGYYLPAIHSSGVTAQYLLKVATKQCFGLESKKVQIG
QLLRSATKEEILHEIKKKLNGQELGFSDGNYPDKTWAVNVLKTIEPNHSLFKTLKCRINQ
ISRRMIQCKIFINIRLIRQLDTSIGRNKRLRYFRRTKEQVEIFKQNAAKKKLARKELIQA
KKLLKLSKLTEELEIVNQGIQEEVEQRQQFQRRN
>AnchoisB consensus (3,621 nt with duplicated TAs)
TATAGTCGAAAACAATTTCAGCCAACATTCACTTTGAGTTGCAAAATTCAATTTTATTTT
GCTATTGCAAAAGCCAAGTAAAAATTGAACCAAGTTTATTTAAAAGCAGGTTTGAAGGAA
ATTTTTGATAATAATTTTTCTTATAATTAATTTAACTTCTAATATTTTTTGTGCACTTTT
ATATATATTGCTTATAATAATATATTTTGCATTATATGATATATAAAGCAAAGTAACGCA
ATTTTATCGCAGCAAATTTTTTTCAGGATATTTTATTAATTTAAATTATGTATGTCTATT
TTTTGTTTTCATTGTTATTTTCAAAATGTCTGAAATATAACCCAACTAGCCCATAACTTG
CCAATATTGCCAGAAAGTATTGTTTGGGTAATTGAACATCAAGGCCCATCGTTTTATTTG
TATTGAACAATTGAAGAAAAATTTGGAATTAGAATTATAGGTTGGCTAAGGAAAAAATCA
ACGCAGGAGAACAAAAAAGTTTATAGAAAAAGTATTGTAGCAAGGTTTAGCAAACTTCCA
ATAAAATGAATCTAAAAGAAATTCAGATCATAAGCAAAGGACCTAACACTTAGATGAAAA
AAATGCTAAAGACAATATTTAGCAATAGCAATTAAAAATTTCAGAAAACTAGCAACAGCA
GGAAATAAAATAAACAGAAAAATAGGCTGAAGAAATAGTAGCAGAAAAAAATCAAGATGT
AGAGCAGAATATTATTTTGAAGAAAGTTAAGAGAATAGTTGAGAAATGGAAATGCTAGAA
ATGCATTAAAGAATTCATAAGGGAACAGGATGGATATCGACATTTTGAGCACCATCATAA
AGATTGTAACGAAGAGGAGAAAAAATTCTTTGAGCAGAGAAGAAAGAAAAAAGTTGCAGA
ATCATAGCAATAGCAAGAAGAAAAATGATATATATATAAATATAAAATTAGAGAATATTT
GATCTTTTTGTTTTGTTTTATTAATAATTATTCAATTTCTTCTTTAAAACGACTGCGCTT
ATTAAACTTCTTCTTAAATTCCTTAATTTATTTATTCTAACTCTTCAGTTAACTTTGATA
ATTTAAGCAATTTTTTTGCTTAAATAAGCTCTTTGCGAGCCTGCTTTTTCTTTGCTGCAT
CTTATTTAAATTTCTCAACTTGCTCCTTCGTTCTGCGGAAATATCTCAACTTTTTGTTTC
TTCCAATAGATGTATCTAATTAACGAATCAAACTTTAATTGAAATAACTGTTACATTAGA
TCATCTTGCGTAAGATTTGATTAATCTTGCACTTtgGGTTCTTGAATAAAGAATGATTCG
GATCTATCGTTTTAAGAATATTGACTGCCCATGTTTTATCAGGGTAGTTTCCATCAGCAA
AGCCAAGCTCTTAGCCATTTAGTTTCTTTTAAATCTCGTGTAAAATTTCCTCTTTAGTTG
CTGATCTTAGCAATTATCCTATTTGAACTTTTTTTGATTCTAAGCTATAGCATTTTTTTG
TAGCAACTCTGAGCAAATATTACGCTGTTACACCTGAAGAATGGATCGCGGGCAAATAAT
ATCCCGCTAAGATTAATCCATAATACATAGATTCAACAGAACCAGTTGCTTAATTTGCTT
GTAAAATGGAAATCATAAAGAATTCTAAATCTTTGTTCAATTAATTTAAAAATGCAAAAG
CAAAATTTATATTGAAAAAAAAATTCTTTTCCAACCAATCAAATTGAAAGCAATTTATCG
CAAATTAAAAAATATAAATAATAAAGAATAAAACAAACAAATTAATTATTATTTTTTAAA
TATAAATTATAACTAGCATTCTTATTGGATTATTTTTAAATTCAAGATTTTTTTCAAAAA
TTGACAAAATATTTTTCCATTCAATTGTTATTATTGGATGTTGGTATGAATTTTAGAATA
TGAAATCTTATTTATTGCAAATAATTCTATTATTTCATAATTTAATGTAAATAGAAACTC
CAGCTTCAACTTCTTCTCTGTAATAATATTATTTGCTATAGCAGGCCTTACACTTAAACT
ACATTATGTAGACATACATGCAATGATGACTGTCAATAATTTTATTAAACCTATGTGTAT
GAAAAAAAAAAAATTTTCATCAAAACAAAACAGCAATTGCAGGAATTGATTACTTTGGAT
GATGCGCTTAAAATTATATAGCAAAAATTATAAACAAAAAGGGAAGAAACGCTAAGAAAA
AAGATTAACAAACCTAAGAGAGCAAATATATTTAAATCTTAACTAACTTTCAAGTTAAAG
TAAATTGCTTAAGAATTAGGAACTTCTATAAAACCTGCAATTTTAGCAGGATATTTAGGA
ATTTCAATTGGAAGGGCTAAATATCTATAAAAGTAAGTTCGAGAGTATCCCGATTCTCTG
AGTCAATAATTTGCTTTTTCATTTGATGAATAGCACAAATTATGGATTGAAAAATTTGTA
GCTTAAGATAATGTTGCACACTTGAGCAGTGCAAAACAAATATATTAAGAATTCATTGTA
AATTTTCATATTAATCAATAACAACTGCAATTTCATTCATTTTTTTAGTGGTTTAAGCAA

CTTGGGTTGTATACTTATCGCTTACTTAGTTGCTTTAATTTTAAATCTCAAACCAAATGC
TCTTTAGAAAGAAGATTTGAATTTGCAAAATAATTAACTACTCTTCTAAGTTAAAATTAA
AAATTAGTCTACATTGATGAATGTAGCTTTGGGAGAAACTTAAAAGCTGGACGATGCTGG
CAAAAAAAGAATAGTTATCCATTGCTGTAAATAAAATCGAACTCCTCAAAAAGTAAATGT
GTAATTGGTGCTATCTGTTGTGATGGGTTTATTGCTTATAAATGTGTTATCGGAAATGTA
AATCAATAGGTATTTGGAGAATTTTTGAATGAACTTTTAAAGCTGCTTTAGCAAAATAAT
GAAGAAAATAATTTTTGCTTAGTGCTGGATAATGCCTCCATCCACCGAACATAATAAATT
CTGAATCAATTATCATTTGTTAATTATTTATTTCTTCCTCCATATAGCCCTTAGCTTAAC
TGCATCGAAATGCTGTGGGGAATTGCTAAGCGATAGCTCTCTAAAATGCATATTGCAACT
ACTGCCTAAGAATTTTATAATATACATGTTGTGCTTAACTCTATTACAACAACTCAAGTT
CAAAACATCTGCACTTCAAACCTAAAATTCCTAGAAGCTTGCTTGTTACGAAAATAATTC
TGATGCTATTATATACAATTTAAATTTTAATATAAAGTTTCAATAGTAAATGAGCAAAAA
GTCTCCGATTCTTTCGAATAGCGATGGTGTAGCAACTGCAGATTGTTAGTCTGGAATGCT
TTAACACTTTCAAGCAGATTTTAGTCGAAGCAGAGTTTCGAATTGGGGGACCGAAAGGGA
GCTAAATCACTCGCTCCTTAATCTATCCAGCCAACTCTCAATATAACAAGCCTTTCATTT
TTATTTATAGTTTTTTGATTATAAAAATAAAATTATGGGTTTGTGTGATTCATGAGTGAT
TATATATAAAAATAGATTTATATTATAAAAGAAATAATATCAGGAAGTCTGAAGAAGTAG
ATGAAATTGTATTGGACTATA
>AnchoisB consensus DDE cds
ATGTAAATAGAAACTCCAGCTTCAACTTCTTCTCTCAGGCCTTACACTTAAACTACATTA
TGTAGACATACATGCAATGATGACTGTCAATAATTTTATTAAACCTATGTGTATGAAAAA
AAAAAAATTTCATCAAAACAAAACAGCAATTGCAGGAATTGATTACTTTGGATGATGCG
CTTAAAATTATATAGCAAAAATTATAAACAAAAAGGGAAGAAACGCTAAGAAAAAAGATT
AACAAACCTAAGAGAGCAAATATATTTAAATCTTAACTAACTTTCAAGTTAAAGTAAATT
GCTTAAGAATTAGGAACTTCTATAAAACCTGCAATTTTAGCAGGATATTTAGGAATTTCA
ATTGGAAGGGCTAAATATCTATAAAAGTAAGTTCGAGAGTATCCCGATTCTCTGAGTCAA
TAATTTGCTTTTTCATTTGATGAATAGCACAAATTATGGATTGAAAAATTTGTAGCTTAA
GATAATGTTGCACACTTGAGCAGTGCAAAACAAATATATTAAGAATTCATTGTAAATTTT
CATATTAATCAATAACAACTGCAATTTCATTCATTTTTTTAGTGGTTTAAGCAACTTGGG
TTGTATACTTATCGCTTACTTAGTTGCTTTAATTTTAAATCTCAAACCAAATGCTCTTTA
GAAAGAAGATTTGAATTTGCAAAATAATTAACTACTCTTCTAAGTTAAAATTAAAAATTA
GTCTACATTGATGAATGTAGCTTTGGGAGAAACTTAAAAGCTGGACGATGCTGGCAAAAA
AAGAATAGTTATCCATTGCTGTAAATAAAATCGAACTCCTCAAAAAGTAAATGTGTAATT
GGTGCTATCTGTTGTGATGGGTTTATTGCTTATAAATGTGTTATCGGAAATGTAAATCAA
TAGGTATTTGGAGAATTTTTGAATGAACTTTTAAAGCTGCTTTAGCAAAATAATGAAGAA
AATAATTTTTGCTTAGTGCTGGATAATGCCTCCATCCACCGAACATAATAAATTCTGAAT
CAATTATCATTTGTTAATTATTTATTTCTTCCTCCATATAGCCCTTAGCTTAACTGCATC
GAAATGCTGTGGGGAATTGCTAAGCGATAGCTCTCTAAAATGCATATTGCAACTACTGCC
TAAGAATTTTATAATATACATGTTGTGCTTAACTCTATTACAACAACTCAAGTTCAAAAC
ATCTGCACTTCAAACCTAAAATTCCTAGAAGCTTGCTTGTTACGAAAATAATTCTGA
>AnchoisB consensus DDE pep
MQIETPASTSSLRPYTQTTLCRHTCNDDCQQFYQTYVYEKKKIFIKTKQQLQELITLDDA
LKIIQQKLQTKREETLRKKINKPKRANIFKSQLTFKLKQIAQELGTSIKPAILAGYLGIS
IGRAKYLQKQVREYPDSLSQQFAFSFDEQHKLWIEKFVAQDNVAHLSSAKQIYQEFIVNF
HINQQQLQFHSFFQWFKQLGLYTYRLLSCFNFKSQTKCSLERRFEFAKQLTTLLSQNQKL
VYIDECSFGRNLKAGRCWQKKNSYPLLQIKSNSSKSKCVIGAICCDGFIAYKCVIGNVNQ
QVFGEFLNELLKLLQQNNEENNFCLVLDNASIHRTQQILNQLSFVNYLFLPPYSPQLNCI
EMLWGIAKRQLSKMHIATTAQEFYNIHVVLNSITTTQVQNICTSNLKFLEACLLRKQF
>AnchoisB consensus ORF1 cds
ATGTCTGAAATATAACCCAACTAGCCCATAACTTGCCAATATTGCCAGAAAGTATTGTTT
GGGTAATTGAACATCAAGGCCCATCGTTTTATTTGTATTGAACAATTGAAGAAAAATTTG
GAATTAGAATTATAGGTTGGCTAAGGAAAAAATCAACGCAGGAGAACAAAAAAGTTTATA
GAAAAAGTATTGTAGCAAGGTTTAGCAAACTTCCAATAAAATGAATCTAAAAGAAATTCA
GATCATAAGCAAAGGACCTAACACTTAGATGAAAAAAATGCTAAAGACAATATTTAGCAA
TAGCAATTAAAAATTTCAGAAAACTAGCAACAGCAGGAAATAAAATAAACAGAAAAATAG
GCTGAAGAAATAGTAGCAGAAAAAAATCAAGATGTAGAGCAGAATATTATTTTGAAGAAA
GTTAAGAGAATAGTTGAGAAATGGAAATGCTAGAAATGCATTAAAGAATTCATAAGGGAA
CAGGATGGATATCGACATTTTGAGCACCATCATAAAGATTGTAACGAAGAGGAGAAAAAA
TTCTTTGAGCAGAGAAGAAAGAAAAAAGTTGCAGAATCATAGCAATAGCAAGAAGAAAAA
TGA
>AnchoisB consensus ORF1 pep
MSEIQPNQPITCQYCQKVLFGQLNIKAHRFICIEQLKKNLELELQVGQGKNQRRRTKKFI
EKVLQQGLANFQQNESKRNSDHKQRTQHLDEKNAKDNIQQQQLKISENQQQQEIKQTEKQ
AEEIVAEKNQDVEQNIILKKVKRIVEKWKCQKCIKEFIREQDGYRHFEHHHKDCNEEEKK

FFEQRRKKKVAESQQQQEEK
>AnchoisB consensus ORF2 cds
ATGATTTCCATTTTACAAGCAAATTAAGCAACTGGTTCTGTTGAATCTATGTATTATGGA
TTAATCTTAGCGGGATATTATTTGCCCGCGATCCATTCTTCAGGTGTAACAGCGTAATAT
TTGCTCAGAGTTGCTACAAAAAAATGCTATAGCTTAGAATCAAAAAAAGTTCAAATAGGA
TAATTGCTAAGATCAGCAACTAAAGAGGAAATTTTACACGAGATTTAAAAGAAACTAAAT
GGCTAAGAGCTTGGCTTTGCTGATGGAAACTACCCTGATAAAACATGGGCAGTCAATATT
CTTAAAACGATAGATCCGAATCATTCTTTATTCAAGAACCcaAAGTGCAAGATTAATCAA
ATCTTACGCAAGATGATCTAATGTAACAGTTATTTCAATTAAAGTTTGATTCGTTAATTA
GATACATCTATTGGAAGAAACAAAAAGTTGAGATATTTCCGCAGAACGAAGGAGCAAGTT
GAGAAATTTAAATAAGATGCAGCAAAGAAAAAGCAGGCTCGCAAAGAGCTTATTTAAGCA
AAAAAATTGCTTAAATTATCAAAGTTAACTGAAGAGTTAGAATAAATAAATTAAGGAATT
TAAGAAGAAGTTTAATAAGCGCAGTCGTTTTAAAGAAGAAATTGA
>AnchoisB consensus ORF2 pep
MISILQANQATGSVESMYYGLILAGYYLPAIHSSGVTAQYLLRVATKKCYSLESKKVQIG
QLLRSATKEEILHEIQKKLNGQELGFADGNYPDKTWAVNILKTIDPNHSLFKNPKCKINQ
ILRKMIQCNSYFNQSLIRQLDTSIGRNKKLRYFRRTKEQVEKFKQDAAKKKQARKELIQA
KKLLKLSKLTEELEQINQGIQEEVQQAQSFQRRN

```
B AnchoisA     1 TATAGTCCAAAATTATTGCAGATTTAAGTTGCTTTGAGTTGCAAAAATTAACTTTTTTTTGCTGAAGCAGAAAGAAACAA
  AnchoisB     1 TATAGTCGAAAACAATTTCAGCCAACATTCACTTTGAGTTGCAAAATTCAATTTTATTTTGCTATTGCAAAAGCCAAGTA

  AnchoisA    81 GAAAT------AAATTGATATA-------TTATGAATAAGAATTTTTAATATT--TTTTTCCCAATGTTGATTCTCTCCC
  AnchoisB    81 AAAATTGAACCAAGTTTATTTAAAAGCAGGTTTGAAGGA-AATTTTTGATAATAATTTTTCTTATAATTAATT-------

  AnchoisA   146 TAAATTTGCATTAATATATTTCCTGCTGTTAATTTGCTGTTATA---ATAGTTTATAA----ATATTCAG----ATAAAA
  AnchoisB   153 TAACTT----CTAATATTTTTTGTGC---------ACTTTTATATATATTGCTTATAATAATATATTTTGCATTATATGA

  AnchoisA   215 TAAAAAAAATAAATTAGTGCAATTTAATTGCAGAAAATATTTTCCAAGAGAATTTA--AATTAGATAAAATCTGCCTGGC
  AnchoisB   220 TATATAAAGCAAAGTAACGCAATTTTATCGCAGCAAATTTTTTTCAGGATATTTTATTAATT---TAAATTATGTATGTC

                                                   ORF1 > M  S  E  K  V  S  S  Q  S  I  T  C  K  H  C
  AnchoisA   293 TATTTTTCGTTTTTCAATATTATCATCAAAATAAATGTCTGAAAAGGTTTCCAGCTAATCTATAACTTGCAAACATTGCT
  AnchoisB   297 TATTTTTTG-TTTTCATTGTTATTTTCA----AAATGTCTGAAATATAACCCAACTAGCCCATAACTTGCCAATATTGCC
                                                   ORF1 > M  S  E  I  Q  P  N  Q  P  I  T  C  Q  Y  C

                  S  T  S  L  F  G  Q  S  N  I  K  L  H  Q  F  V  C  V  E  Q  I  Q  K  N  M  E  E
  AnchoisA   373 CAACTTCATTATTTGGACAATCGAATATCAAGCTTCATCAATTTGTTTGCGTTGAACAAATTTAGAAAAATATGGAAGAA
  AnchoisB   372 AGAAAGTATTGTTTGGGTAATTGAACATCAAGGCCCATCGTTTTATTTGTATTGAACAATTGAAGAAAAATTTGGAATTA
                  Q  K  V  L  F  G  Q  L  N  I  K  A  H  R  F  I  C  I  E  Q  L  K  K  N  L  E  L

                  A  L  N  I  G  G  G  K  N  Q  R  R  R  N  K  K  C  I  E  R  I  L  Q  Q  G  L  E
  AnchoisA   453 GCTTTAAATATTGGAGGAGGGAAAAATCAACGAAGAAGAAACAAAAAAATGCATTGAAAGAATTTTACAGCAGGGTTTGGA
  AnchoisB   452 GAATTATAGGTTGGCTAAGGAAAAAATCAACGCAGGAGAACAAAAAAGTTTATAGAAAAAGTATTGTAGCAAGGTTTAGC
                  E  L  Q  V  G  Q  G  K  N  Q  R  R  R  T  K  K  F  I  E  K  V  L  Q  Q  G  L  A

                  N  F  Q  Q  H  Q  I  K  R  N  Q  Q  S  A  Q  R  N  S  K  L  D  I  K  I  S  D
  AnchoisA   533 AAACTTCCAATAGCACTAAATCAAAAGAAATCAACAAAGTGCACAAAGAAATTCGAAATTAGATATCAAAATTAGCGACC
  AnchoisB   532 AAACTTCCAATAAAATGAATCTAAAAGAAATTCAGATCATAAGCAAAGGACCTAACACTTAGATGAAAAAAATGCTAAAG
                  N  F  Q  Q  N  E  S  K  R  N  S  D  H  K  Q  R  T  Q  H  L  D  E  K  N  A  K

                  Q  N  F  K  Q  Q  Q  I  N  I  L  E  K  K  E  Q  Q  E  S  N  Q  K  D  K  Q  Y  E
  AnchoisA   613 AAAATTTTAAGCAACAGCAAATAAATATTTTAGAGAAAAAGGAATAGCAAGAAAGCAATTAGAAAGATAAACAATATGAA
  AnchoisB   612 ACAATATTTAGCAATAGCAATTAAAAATTTCAGAAAACTAGCAACAGCAGGAAATAAAATAAACAGAAAAATAGGCTGAA
                  D  N  I  Q  Q  Q  Q  L  K  I  S  E  N  Q  Q  Q  Q  E  I  K  Q  T  E  K  Q  A  E

                  E  I  L  S  E  S  K  Q  E  F  E  Q  N  I  I  L  R  K  V  K  R  V  V  E  K  W  K
  AnchoisA   693 GAAATATTGTCAGAGAGCAAGCAAGAATTTGAGCAGAACATTATTTTAAGAAAAGTGAAGAGAGTGGTAGAGAAGTGGAA
  AnchoisB   692 GAAATAGTAGCAGAAAAAAATCAAGATGTAGAGCAGAATATTATTTTGAAGAAAGTTAAGAGAATAGTTGAGAAATGGAA
                  E  I  V  A  E  K  N  Q  D  V  E  Q  N  I  I  L  K  K  V  K  R  I  V  E  K  W  K

                  C  Q  R  C  I  K  E  F  M  R  E  Q  D  G  Y  R  H  F  E  F  H  H  K  D  L  N
  AnchoisA   773 GTGCTAAAGATGTATAAAAGAGTTCATGAGGGAGCAGGATGGGTATCGGCACTTTGAGTTCCATCATAAGGATTTGAATG
  AnchoisB   772 ATGCTAGAAATGCATTAAAGAATTCATAAGGGAACAGGATGGATATCGACATTTTGAGCACCATCATAAAGATTGTAACG
                  C  Q  K  C  I  K  E  F  I  R  E  Q  D  G  Y  R  H  F  E  H  H  H  K  D  C  N

                 E  E  E  K  K  F  F  D  S  R  K  K  K  K  G  A  E  A  E  Q  Q  Q  D  E  K  * > ORF1
  AnchoisA   853 AAGAAGAGAAAAAGTTCTTTGACTCGAGAAAGAAGAAAAAAGGTGCTGAAGCAGAATAGTAGCAGGATGAAAAATGAGAT
  AnchoisB   852 AAGAGGAGAAAAAATTCTTTGAGCAGAGAAGAAAGAAAAAAGTTGCAGAATCATAGCAATAGCAAGAAGAAAAATGATAT
                 E  E  E  K  K  F  F  E  Q  R  R  K  K  K  V  A  E  S  Q  Q  Q  Q  E  E  K  * > ORF1

                                                                       ORF2 < *  N  R  R  Q  F
  AnchoisA   933 ACCATAAAATAT---ATTAAAAAATGCATGAATAATTTTCTACTTCTTTTTCATAATAA----TCAATTTCTTCTTTAGA
  AnchoisB   932 ATATATAAATATAAAATTAGAGAATATTTG---ATCTTTTTGTTTTGTTTTATTAATAATTATTCAATTTCTTCTTTAAA
                                                                       ORF2 < *  N  R  R  Q  F

                  Q  Q  R  Q  E  V  E  E  Q  I  G  Q  N  V  I  E  L  E  E  T  L  K  S  L  K  L
  AnchoisA  1006 ATTGCTGTCTCTATTCTACTTCCTCTTAAATTCCTTAATTCACTATTTCTAATTCTTCAGTTAACTTTGATAATTTTAGC
  AnchoisB  1009 ACGACTGCGCTTATTAAACTTCTTCTTAAATTCCTTAATTTATTTATTCTAACTCTTCAGTTAACTTTGATAATTTAAGC
                  S  Q  A  Q  Q  V  E  E  Q  I  G  Q  N  I  Q  E  L  E  E  T  L  K  S  L  K  L

                 L  K  K  A  Q  I  L  E  K  R  A  L  K  K  K  A  A  N  Q  K  F  I  E  V  Q  E  K
  AnchoisA  1086 AACTTTTTTGCTTAAATGAGTTCTTTGCGAGCTAACTTTTTCTTAGCTGCATTTTATTTAAATATCTCAACTTACTCCTT
  AnchoisB  1089 AATTTTTTTGCTTAAATAAGCTCTTTGCGAGCCTGCTTTTTCTTTGCTGCATCTTATTTAAATTTCTCAACTTGCTCCTT
                 L  K  K  A  Q  I  L  E  K  R  A  Q  K  K  K  A  A  D  Q  K  F  K  E  V  Q  E  K

                  T  R  R  F  Y  R  L  R  K  N  R  G  I  S  T  D  L  Q  R  I  L  R  I  N  I  F  I
  AnchoisA  1166 TGTTCTGCGAAAATATCTCAGCCTTTTATTTCTTCCGATAGAAGTGTCTAATTAGCGAATCAACCTTATGTTGATAAATA
  AnchoisB  1169 CGTTCTGCGGAAATATCTCAACTTTTTGTTTCTTCCAATAGATGTATCTAATTAACGAATCAAACTTTAATTGAAATAAC
                  T  R  R  F  Y  R  L  K  K  N  R  G  I  S  T  D  L  Q  R  I  L  S  Q  N  F  Y  S

                  K  C  Q  I  M  R  R  S  I  Q  N  I  R  C  K  L  T  K  F  L  S  H  N  P  E  I
  AnchoisA  1246 TTTTACATTAAATCATCCTGCGTGAGATTTGATTAATCCTGCACTTTAGGGTTTTAAATAAAGAATGATTTGGTTCTATT
  AnchoisB  1249 TGTTACATTAGATCATCTTGCGTAAGATTTGATTAATCTTGCACTTTGGGTTCTTGAATAAAGAATGATTCGGATCTATC
                  N  C  Q  I  M  K  R  L  I  Q  N  I  K  C  K  P  N  K  F  L  S  H  N  P  D  I

                 T  K  L  V  N  V  A  W  T  K  D  P  Y  N  G  D  S  F  G  L  E  Q  G  N  L  K  K
  AnchoisA  1326 GTTTTAAGAACATTAACTGCCCATGTTTTATCAGGGTAGTTTCCATCAGAAAAGCCAAGCTCTTAACCATTAAGCTTCTT
  AnchoisB  1329 GTTTTAAGAATATTGACTGCCCATGTTTTATCAGGGTAGTTTCCATCAGCAAAGCCAAGCTCTTAGCCATTTAGTTTCTT
                 T  K  L  I  N  V  A  W  T  K  D  P  Y  N  G  D  A  F  G  L  E  Q  G  N  L  K  K

                  K  I  E  H  L  I  E  E  K  T  A  S  R  L  L  Q  G  I  Q  V  K  K  S  E  L  G  F
  AnchoisA  1406 TTTAATTTCATGTAAAATTTCTTCTTTAGTAGCTGATCTCAGCAGTTATCCTATTTGAACTTTTTTTGATTCTAAGCCAA
  AnchoisB  1409 TTAAATCTCGTGTAAAATTTCCTCTTTAGTTGCTGATCTTAGCAATTATCCTATTTGAACTTTTTTTGATTCTAAGCTAT
                  Q  I  E  H  L  I  E  E  K  T  A  S  R  L  L  Q  G  I  Q  V  K  K  S  E  L  S  Y
```

```
                C  Q  K  T  A  V  K  L  L  Y  Q  A  T  V  G  S  S  H  I  A  P  L  Y  Y  G  A
AnchoisA 1486 AGCATTATTTAGTAGCAACTTTGAGCAAATATTACGCTGTTACACCTGAAGAATGAATTGCAGGTAAATAATATCCTGCC
AnchoisB 1489 AGCATTTTTTTGTAGCAACTCTGAGCAAATATTACGCTGTTACACCTGAAGAATGGATCGCGGGCAAATAATATCCCGCT
                C  K  K  T  A  V  R  L  L  Y  Q  A  T  V  G  S  S  H  I  A  P  L  Y  Y  G  A

              L  I  L  G  Y  Y  M  S  E  V  S  G  T  A  Q  N  A  Q  F  I  S  I  M < ORF2
AnchoisA 1566 AAGATTAAGCCATAGTACATAGATTCAACAGAACCAGTTGCTTGATTAGCTTAGAAAATGGAAATCATGATGAATTTCGA
AnchoisB 1569 AAGATTAATCCATAATACATAGATTCAACAGAACCAGTTGCTTAATTTGCTTGTAAAATGGAAATCATAAAGAATTCTAA
              L  I  L  G  Y  Y  M  S  E  V  S  G  T  A  Q  N  A  Q  L  I  S  I  M < ORF2

AnchoisA 1646 AAGTTTGTTTAATTATTCAAACAATGCGATAGT-AAATTTTTATTGAAAAAAAAATTCTTTTTTAACCAATCAATTTCAA
AnchoisB 1649 ATCTTTGTTCAATTAATTTAAAAATGCAAAAGCAAAATTTATATTGAAAAAAAAATTCTTTTCCAACCAATCAAATTGAA

AnchoisA 1725 AGCAATTGATT-----TTAAAAGCAGAAAACAATTTAGAATAAATTAAAGAAAAATATTAAATGATTTATAATTTTAATG
AnchoisB 1729 AGCAATTTATCGCAAATTAAAAAATATAAATAATAAAGAATAAAACAAACAAATTAATTA------TTATTTTTTAAATA

AnchoisA 1800 CAACTTAAATACTGCTTTTTTATTGAATTATATTTATAATGAGGATTTTTTGTTTTGAATTGGAGAAAATATTTTG--AT
AnchoisB 1803 TAAATTATAACTAGCATTCTTATTGGATTATTTTTAAATTCAAGATTTTT--TTCAAAAATTGACAAAATATTTTTCCAT

AnchoisA 1878 TCAATTTTGATTA-------TTGATTCGAATTTTAGATTAT-AGCTCCAATTTTTATGCAAGAAATTTAATTTTTCCAC-
AnchoisB 1881 TCAATTGTTATTATTGGATGTTGGTATGAATTTTAGAATATGAAATCTTATTTAT-TGCAAATAATTCTATTATTTCATA

              DDE > M  Q  I  E  T  Q  A     S  S  S  L                       R  P  Y  A  Q  T
AnchoisA 1949 ATTTAATGTAAATAGAAACTCAAGCTT---CTTCATCTCTGTACATATATTATTTGCTATAGCAGGCCATATGCTTAAAC
AnchoisB 1960 ATTTAATGTAAATAGAAACTCCAGCTTCAACTTCTTCTCTGTAATAATATTATTTGCTATAGCAGGCCTTACACTTAAAC
              DDE > M  Q  I  E  T  P  A  S  T  S  S  L                       R  P  Y  T  Q  T

                T  I  C  R  H  I  C  D  D  H  C  Q  Q  F  Y  Q  T  Y  V  Y     E  K  R  L  L
AnchoisA 2026 AACAATATGTAGACACATATGCGATGATCATTGCTAATAATTTTACTAAACCTATGTTTATG---AAAAAAGACTTTTAA
AnchoisB 2040 TACATTATGTAGACATACATGCAATGATGACTGTCAATAATTTTATTAAACCTATGTGTATGAAAAAAAAAAAATTTTCA
                T  L  C  R  H  T  C  N  D  D  C  Q  Q  F  Y  Q  T  Y  V  Y  E  K  K  K  I  F

              I  K  T  K  Q  Q  M  L  E  L  L  T  L  E  D  A  H  K  I  I  K  Q  K  L  E  S  T
AnchoisA 2103 TCAAAACCAAATAGCAAATGCTGGAACTGCTTACTCTAGAGGATGCTCATAAAATTATAAAACAAAAATTAGAATCCACA
AnchoisB 2120 TCAAAACAAAACAGCAATTGCAGGAATTGATTACTTTGGATGATGCGCTTAAAATTATATAGCAAAAATTATAAACAAAA
              I  K  T  K  Q  Q  L  Q  E  L  I  T  L  D  D  A  L  K  I  I  Q  Q  K  L  Q  T  K

                R  E  V  K  L  R  K  N  I  N  K  F  E  K  K  S  N  F  K  S     L  T  L  K  L  Q
AnchoisA 2183 AGGGAAGTAAAGTTGAGAAAAAATATAAACAAATTTGAGAAAAAAAGCAACTTCAAATC---ATTAACTTTAAAATTGCA
AnchoisB 2200 AGGGAAGAAACGCTAAGAAAAAAGATTAACAAACCTAAGAGAGCAAATATATTTAAATCTTAACTAACTTTCAAGTTAAA
                R  E  E  T  L  R  K  K  I  N  K  P  K  R  A  N  I  F  K  S  Q  L  T  F  K  L  K

                K  I  A  L  E  L  G  N  S  V  K  P  A  I  L  A  G  Y  L  G  I  S  I  G  R  A
AnchoisA 2260 GAAAATTGCTTTAGAATTAGGAAATTCGGTAAAACCTGCAATTTTGGCAGGATATTTAGGAATTTCTATTGGAAGAGCTA
AnchoisB 2280 GTAAATTGCTTAAGAATTAGGAACTTCTATAAAACCTGCAATTTTAGCAGGATATTTAGGAATTTCAATTGGAAGGGCTA
                Q  I  A  Q  E  L  G  T  S  I  K  P  A  I  L  A  G  Y  L  G  I  S  I  G  R  A

              K  Y  L  Q  K  Y  V  L  Q  Q  P  D  L  Q  R  Q  Q  L  A  F  K  F  D  A  Q  H  K
AnchoisA 2340 AATATTTATAAAAGTATGTTTTACAGCAACCTGATTTACAAAGACAATAACTTGCTTTTAAATTTGATGCATAACATAAA
AnchoisB 2360 AATATCTATAAAAGTAAGTTCGAGAGTATCCCGATTCTCTGAGTCAATAATTTGCTTTTTCATTTGATGAATAGCACAAA
              K  Y  L  Q  K  Q  V  R  E  Y  P  D  S  L  S  Q  Q  F  A  F  S  F  D  E  Q  H  K

               F  W  I  Q  K  F  V  A  Q  E  N  V  A  R  L  S  S  A  K  Q  I  Y  Q  E  F  I  A
AnchoisA 2420 TTTTGGATTTAAAAATTTGTAGCTCAAGAAAATGTTGCTCGCTTGAGCAGTGCAAAATAAATCTATTAAGAATTTATAGC
AnchoisB 2440 TTATGGATTGAAAAATTTGTAGCTTAAGATAATGTTGCACACTTGAGCAGTGCAAAACAAATATATTAAGAATTCATTGT
               L  W  I  E  K  F  V  A  Q  D  N  V  A  H  L  S  S  A  K  Q  I  Y  Q  E  F  I  V

                N  F  H  I  C  K  Q  Q  L  Q  F  H  Q  F  F  K  Q  F  K  Q  L  W  L  Y  T  Y
AnchoisA 2500 AAATTTTCATATTTGCAAATAGCAACTGCAGTTTCATTAATTTTTTAAGTAGTTTAAACAGCTTTGGTTATATACCTATC
AnchoisB 2520 AAATTTTCATATTAATCAATAACAACTGCAATTTCATTCATTTTTTTAGTGGTTTAAGCAACTTGGGTTGTATACTTATC
                N  F  H  I  N  Q  Q  Q  L  Q  F  H  S  F  F  Q  W  F  K  Q  L  G  L  Y  T  Y

              R  L  F  S  C  V  N  F  K  S  Q  T  K  C  S  L  D  R  R  F  E  F  A  K  Q  L  A
AnchoisA 2580 GCTTATTTAGTTGCGTTAATTTTAAATCTCAAACTAAATGTTCTTTAGACAGAAGGTTTGAATTTGCAAAATAATTAGCT
AnchoisB 2600 GCTTACTTAGTTGCTTTAATTTTAAATCTCAAACCAAATGCTCTTTAGAAAGAAGATTTGAATTTGCAAAATAATTAACT
              R  L  L  S  C  F  N  F  K  S  Q  T  K  C  S  L  E  R  R  F  E  F  A  K  Q  L  T

                T  L  L  N  S  K  Q  K  I  V  Y  I  D  E  C  S  F  G  R  N  L  K  V  A  R  G  W
AnchoisA 2660 ACTCTTTTAAATTCAAAATAAAAAATTGTTTACATTGATGAGTGCAGTTTTGGGAGAAATTTAAAAGTTGCAAGAGGTTG
AnchoisB 2680 ACTCTTCTAAGTTAAAATTAAAAATTAGTCTACATTGATGAATGTAGCTTTGGGAGAAACTTAAAAGCTGGACGATGCTG
                T  L  L  S  Q  N  Q  K  L  V  Y  I  D  E  C  S  F  G  R  N  L  K  A  G  R  C  W

                Q  K  K  N  T  F  P  L  L  Q  I  K  S  S  S  S  S  K  N  K  C  V  I  G  A  I  A
AnchoisA 2740 GTAAAAAAAGAACACTTTTCCATTATTGCAAATTAAATCAAGCTCATCAAAAAATAAATGTGTTATTGGAGCTATAGCTT
AnchoisB 2760 GCAAAAAAAGAATAGTTATCCATTGCTGTAAATAAAATCGAACTCCTCAAAAAGTAAATGTGTAATTGGTGCTATCTGTT
                Q  K  K  N  S  Y  P  L  L  Q  I  K  S  N  S  S  K  S  K  C  V  I  G  A  I  C

              C  D  G  F  I  A  Y  K  C  V  I  G  N  V  N  Q  Q  V  F  G  E  F  L  N  E  L  L
AnchoisA 2820 GTGACGGGTTTATTGCTTACAAATGTGTTATAGGAAATGTTAATCAATAGGTTTTTGGAGAATTTTTGAATGAACTTCTA
AnchoisB 2840 GTGATGGGTTTATTGCTTATAAATGTGTTATCGGAAATGTAAATCAATAGGTATTTGGAGAATTTTTGAATGAACTTTTA
              C  D  G  F  I  A  Y  K  C  V  I  G  N  V  N  Q  Q  V  F  G  E  F  L  N  E  L  L

                R  L  L  Q  Q  N  S  E  E  N  N  F  C  L  V  L  D  N  A  S  I  H  R  T  S  Q  I
AnchoisA 2900 AGGCTGCTTTAGCAGAATAGTGAAGAAAATAATTTTTGCTTGGTGCTGGACAATGCATCTATCCATCGCACATCACAAAT
AnchoisB 2920 AAGCTGCTTTAGCAAAATAATGAAGAAAATAATTTTTGCTTAGTGCTGGATAATGCCTCCATCCACCGAACATAATAAAT
                K  L  L  Q  Q  N  N  E  E  N  N  F  C  L  V  L  D  N  A  S  I  H  R  T  Q  Q  I
```

```
                L  D  Q  L  S  Y  V  N  Y  L  F  L  P  P  Y  S  P  Q  L  N  C  I  E  K  L  W
AnchoisA 2980 TTTGGATTAATTGTCTTATGTGAACTATTTATTTCTCCCACCATATAGTCCTTAGCTTAATTGCATTGAAAAGCTATGGG
AnchoisB 3000 TCTGAATCAATTATCATTTGTTAATTATTTATTTCTTCCTCCATATAGCCCTTAGCTTAACTGCATCGAAATGCTGTGGG
                L  N  Q  L  S  F  V  N  Y  L  F  L  P  P  Y  S  P  Q  L  N  C  I  E  M  L  W

              G  V  A  K  Q  K  L  S  K  M  H  F  A  T  T  F  Q  E  F  H  N  I  H  F  V  L  N
AnchoisA 3060 GAGTAGCAAAACAAAAGCTCTCCAAAATGCACTTCGCAACAACATTTTAAGAATTTCACAACATTCATTTTGTGCTTAAC
AnchoisB 3080 GAATTGCTAAGCGATAGCTCTCTAAAATGCATATTGCAACTACTGCCTAAGAATTTTATAATATACATGTTGTGCTTAAC
              G  I  A  K  R  Q  L  S  K  M  H  I  A  T  T  A  Q  E  F  Y  N  I  H  V  V  L  N

               S  I  T  A  N  Q  V  Q  N  I  C  T  F  N  L  K  F  L  Q  A  C  L  H  R  Q  Q  F
AnchoisA 3140 TCAATTACAGCTAATTAAGTGCAAAATATTTGCACCTTTAACTTGAAATTCTTGCAAGCTTGCTTACATAGACAGCAGTT
AnchoisB 3160 TCTATTACAACAACTCAAGTTCAAAACATCTGCACTTCAAACCTAAAATTCCTAGAAGCTTGCTTGTTACGAAAATAATT
               S  I  T  T  T  Q  V  Q  N  I  C  T  S  N  L  K  F  L  E  A  C  L  L  R  K  Q  F

                * > DDE
AnchoisA 3220 TTGAT-TAATTATATTTTCATTTAAACCATAAAATA----TTCAATGCAAGCAATTAACAATAAAAATTCTCTACTCTTT
AnchoisB 3240 CTGATGCTATTATA-TACAATTTAAATTTTAATATAAAGTTTCAAT---AGTAAATG--AGCAAAAAGTCTCCGATTCTT
                * > DDE

AnchoisA 3295 TCTTATAGCGACAGTGT-GCAAAAGCAGACT-TTCGTCAGTTATAC-TTAACACTATCGAGCAGATATTAATAAGAGCAG
AnchoisB 3314 TCGAATAGCGATGGTGTAGCAACTGCAGATTGTTAGTCTGGAATGCTTTAACACTTTCAAGCAGATTTTAGTCGAAGCAG

AnchoisA 3372 AGTTTCGAATCGGGGGACAGGATGGGATCTAAAACACTCGCTCCTTAACCTTTCCAGCCTATTCTCAATACTATTAGACT
AnchoisB 3394 AGTTTCGAATTGGGGGACCGAAAGGGAGCTAAATCACTCGCTCCTTAATCTATCCAGCCAACTCTCAATATAACAAGCCT

AnchoisA 3452 CTGATTTAATTATATATTTTAAAATTTGAT----AAAATTAAATTATGGGTTTGGGGTAATTCATTAAATATTATATAAT
AnchoisB 3474 TTCATTTTTATTTATAGTTT----TTTGATTATAAAAATAAAATTATGGGTTT-GTGTGATTCATGAGTGATTATATATA

AnchoisA 3528 TATATATAATTTATATTATTATA-AATTTATAT----AATCCTGCAAAAGCAGAAAGTCAAATTGTATTTGACTATA
AnchoisB 3549 AAAATA-GATTTATATTATAAAAGAAATAATATCAGGAAGTCTGAAGAAGTAGA---TGAAATTGTATTGGACTATA
```

C

```
CTG40749b        -------------------------------------------------------------------------------
IES77            TATAGTCTAAAATTATTGAAGATTCAAATTGCTTTGAGTTGCAAA----AATGA--CTTTTTTTGCTGAAGCAGAAAGAA
CTG23427         -------------------------------------------------------------------------------
CTG35975         TATAGTCGAAAATTAATGAAGATTTAAGTTGTTTTGAGTTGCAAAAAATTAATTAATTTTTTTTTCCTGAAGGAGAAAGAA
CTG21499         TATAGTCCAAAATTATAGCAGATT------ACTTTGAGTTGCAAA----AATTAATGTTTTTTAGCTGAAGCAGAAAGAT
CTG40749         TATAGTCCAAAATTATTGCAGATTTAAGTTGCTTGGAGTTGAAAA----AATTCATTATTTTTTGCTGAAGCAGAAAGA-
IES128           TATAGTCCAAAATTATAGCAGATTCAAGTTGCATTGAGTTGCAAA----AATTAACTTTTGTTGCTGAAGCAGAAAGAA
CTG28766         TATAGTCCAAAATTATTGCAGATTTAAGTTGCTTTGA------------GATTAACTTTTT-TTGCTGAAGTAGAAAGAA
IES98            TATAGACCAAAATTAATGCAGATTTAAGTTGCTTTGCGTTGCAAA----ATTTAACTTTTTTTTGCTGAAGCAAAAAGAA
CTG10063         -------------------------------------------------------------------------------
AnchoisA       1 TATAGTCCAAAATTATTGCAGATTTAAGTTGCTTTGAGTTGCAAA----AATTAACTTTTTTTGCTGAAGCAGAAAGAA

CTG40749b        -------------------------------------------------------------------------------
IES77            ACAAGA-----AATAAATTAATATAGTACGAATAAGAATTTTTAATATTTTTTTCCCAATTTTGATTTTCTCCTTAAATT
CTG23427         -------------------------------------------------------------------------------
CTG35975         ACGAGAATATTAATTAATTGATAGATTATGAATAAGAATTTTTAATATTTTTT-CTGAATGTTGATTCT----CTACATT
CTG21499         ACAAGG-----AATCAATTTAAATGTTATGAATAAGAATTTTTAATATTTTTCC--AATATTGATTCTCTCCCTAAATC
CTG40749         ACAAGA-----AATAAATTGATATATTAAGAATAAGAATTTTTAATATTTTTTTCCCAATGTTGATTGTCT-CCTAAATT
IES128           ACAAGA-----AATAAATTGATATATTATGAATAAGAATTTTTAATATTTTTTCCC--ACGTTGATTCTCTCCCTAAATT
CTG28766         ACAAGA-----AATAATATGATATATTATGAATAAGAATGTTTAGTATTTTTTTCACAATTTTAATGGTCTCCCTAAATT
IES98            ACTAGA-----AGTAAATTGATATATTATGAATAAGAATTTTCATTATTTTTTTCCGAATGTTGATTCT---CCTAAATT
CTG10063         -------------------------------------------------------------------------------
AnchoisA      77 ACAAGA-----AATAAATTGATATATTATGAATAAGAATTTTTAATATTTTTTTCCCAATGTTGATTCTCTCCCTAAATT

CTG40749b        -------------------------------------------------------------------------------
IES77            TGCATTAATATATTTCCTGCTGTTAATTTGCTGTTATATTAGTTTATAAATATTCAGATAATATAAAAAAAATAAATTAG
CTG23427         -------------------------------------------------------------------------------
CTG35975         TGCATTAATATAATTCCTGCTGTTGATTTGTTGTTACAATAGCTTGT--ATATTCAGATAAAATAAAAAAAATAAATTAG
CTG21499         TGCATTAATCTATTTCCTGCTATTAATTTGCCGTTATAATAGTTTATAAATATTCAGATAAAATAAAAAAAATAATTTAG
CTG40749         TGCATTATTATATTTCCTGCTCTTGATTTGTTGTTATAATAGTTTATAAATATTCAGATAAAGTAAAAAATATAAAGTAG
IES128           TGCATTAATATATTTCCCGCAGTTAATTTGCTGTTATAATAGTTTATAAATATTCAGATAAAATAAAAAA-ATAAATTAA
CTG28766         TGAATTAATATATTTCCCGCTGATAATTTGCTATTATAATAGTTTATAAATATTTAGACAAAATAAAATAAATAA-TTCG
IES98            TTCATTAATATATTTCCTGCTGTTAATTTGCTAT-----TAGTTTATAAATATTCAGATAATATAAAAAAATAAAAATAG
CTG10063         -------------------------------------------------------------------------------
AnchoisA     152 TGCATTAATATATTTCCTGCTGTTAATTTGCTGTTATAATAGTTTATAAATATTCAGATAAAATAAAAAAAATAAATTAG

CTG40749b        -------------------------------------------------------------------------------
IES77            TGCAATTTAATTGTAGAAAGT-TTTTCTAAGAGAATTTTAATTAAATAAA-ATCTGCCTG-----CAAATTATCG--TTT
CTG23427         -------------------------------------------------------------------------------
CTG35975         TGCGATTTAATCGCAGACAATATTTTTCAAGAGAATTTAAATTAAATAAA-ATCATCCTA----CCTATTTT--GGTTTT
CTG21499         TGCAATTATATTGCAGAAAATATTTTCCAAGAGAATTTAAATTAGAGAAATATCTGCCTG----CCTATTTTTCG-TTTT
CTG40749         TGCAATTTTATTGCAGAAAATATTTTCCAAGAGAATTTAAATTAGACAAA-ATCTGCCTG----GCTATTTTTCG-TTTT
IES128           TGCCATT-AATTGCAGAAAATATTTTCCAAGAGAATTTAAATTAGATAAA-ATCTGCCTGCCTAGCTATTTTTCG-TTTT
CTG28766         TGTAAAATGATTGCAGAATATAGTTTCCAAGAGAATTTAAATTAGATAAA-ATCTGCCTG----ACTATTTTTCGTTTTT
IES98            TGCAATTTAATTGCAGAAAATTTTTCCCAAAAGAATTTAAAATAGGTAAA-ATATTTCTG----GCTATTTTTCG-TTTT
CTG10063         -------------------------------------------------------------------------------
AnchoisA     232 TGCAATTTAATTGCAGAAAATATTTTCCAAGAGAATTTAAATTAGATAAA-ATCTGCCTG----GCTATTTTTCG-TTTT

CTG40749b        -------------------------------------------------------------------------------
IES77            TCAATATTATCATCAAAATAAATGTCTGAAAAGGTTTCCAGCTAATTTATAACTTATAAATATTGCTCAAC--TTCATTA
CTG23427         -------------------------------------------------------------------------------
CTG35975         CCAATATTACCATCATAATA---------------------------------------------GCTCAATATTTCATTA
CTG21499         TCAATATTTTCATCAAA-----TGTCTGAAAAGGTTTCCAGCTAATCTACCACTTGCAAACATTGCTCAAC--TTCATTA
CTG40749         TCAATATTTTCATTAAAATAATTGTCTGAAAAGGTTTCCAGCTAATCTATAACTTGCAAAATTGCTCAAC--TTCATTA
IES128           TCTACATTATCATCAAAA-AAATGTCTG-AAAGGTTTCCAGCTAATCTATAACTTGCAAAGATTGCTCAAC--TTCATTA
CTG28766         TCAATATAATTATCAAAATAAATGTCTGAAAAGGTTTCCAGCTAATCCATAACTTGCAAACATTGCTTAAC--TTCATTA
IES98            TCAATGTTATCATCAAAATAAATGTCTGAAAAAGTTTCCAGCTAATCTATAA----------------------------
CTG10063         -------------------------------------------------------------------------------
AnchoisA     306 TCAATATTATCATCAAAATAAATGTCTGAAAAGGTTTCCAGCTAATCTATAACTTGCAAACATTGCTCAAC--TTCATTA

CTG40749b        -------------------------------------------------------------------------------
IES77            TTTGGACAATCAAATATCAGGCTTCATCAATTTGTTTGCGTTGAAC-AAATTTAGAAAAATATCAAAGAAGATTAAAATA
CTG23427         -------------------------------------------------------------------------------
CTG35975         TTTGGACAATCGAATAACTAGCTTTATCAATTTATTTGATGTGAAC-AAATTTAGAAAAATATGGATGTGGCTTTAAATA
CTG21499         TTTGGACAATCGAATATTAAGCTTCATCAATTTGTTTGCGTTGAACAAAATTTAGAAAAATATGGAAGAAG-TTTAAATA
CTG40749         TTGGGACAATCAAATATCATGCTTCATCAATTTGTTTGCGTTGTAC-AAATTTAGAAAAATATGGAAGAAAC--------
IES128           TTTGGACAATCGAATATCACGCTTCATCAATTTGTTTACGTGCAAA-AAATTTAGAAAAATATGGAAGAAGCTTTAAATA
CTG28766         TCCTGACAATCGAATATCAAACTTCGTCAATTTGTTGGAGTTGAAC-AAATTTAGAAAAATGTGGAAGAAGCTTTAA---
IES98            ------------------------------------------------------------------GC-TTAAATG
CTG10063         -------------------------------------------------------------------------------
AnchoisA     384 TTTGGACAATCGAATATCAAGCTTCATCAATTTGTTTGCGTTGAAC-AAATTTAGAAAAATATGGAAGAAGCTTTAAATA

CTG40749b        -------------------------------------------------------------------------------
IES77            TTGGAGGAGGGAAAAATCAATGAAGATGAAACAAAAAATGCA-TTGAAAGAATTTTACAGAAGGGTTTGTA-AAACTTCC
CTG23427         -------------------------------------------------------------------------------
CTG35975         TTGGAGTAGGGAAAAATCAAAATGAAGAAAC-AAAAATGCA-TTGAAAG-ATTTTATAGCA-GGTTTGGA-AAACTTCC
CTG21499         TTGGAGGAGGAAAAAAATCAACGAAGTAGAAACAAAAAATGCA-TTGAAAATATTTTACAGCAGGGTTTTGA-AAACTTCC
CTG40749         ---------------------------------AAAACTGCATTTGAAAGAATTTTACTGCAGGGTTTTGATAAACCTCC
IES128           TTGGAGGAGGGAAAAATCAACGAAGAGAAA--AAAAATGCA-TGGAAAGAATTTTACAGCAGGGTTTGGA-AAACTTCC
CTG28766         ------GGAGGGAAAATCAATGAAGAGGAAG------------------------------------------------
IES98            TTGGCGGAGGG-AAAATCAACAAAGAAGAAACAAAAAATTA--TTGAAAAAATTTAC-GCAACGTTTGAAAAAATTTCC
CTG10063         -------------------------------------------------------------------------------
AnchoisA     463 TTGGAGGAGGGAAAAATCAACGAAGAAGAAACAAAAAATGCA-TTGAAAGAATTTTACAGCAGGGTTTGGA-AAACTTCC
```

```
CTG40749b      --------------------------------------------------------------------------------
IES77          AA-TAGCACT-----------------------------------------------------------------AAAAT
CTG23427       --------------------------------------------------------------------------------
CTG35975       AA-TACAACTAAATCAAAAG---------AAAGTGCAC-TAAGAAATTCAAAAT--------------AGCAACCAAAAT
CTG21499       AA-TAGCGCT----------------------------------------------------------------------
CTG40749       AA-TAGCACTAAATCAAAAAAAATCAAC-AAAGTGCACAAAAGAAATTCGAAATTAGATTTCAAGATTAGCGACCAAAAT
IES128         AA-TAACACTAAATCAAAAGAAATAAAC-AAAGTGCAC-AAAGAAATTCGAAATTAGATATCAAAATTAGTGATCAAAAT
CTG28766       --------------------------------------------------------------------------------
IES98          AAATAGTGCTAAATCAGAAGAAATCAACAAAAGTGCAT-AAAGAAATTCAAAATTAGA-------------GACCAAAAT
CTG10063       --------------------------------------------------------------------------------
AnchoisA   541 AA-TAGCACTAAATCAAAAGAAATCAAC-AAAGTGCAC-AAAGAAATTCGAAATTAGATATCAAAATTAGCGACCAAAAT

CTG40749b      --------------------------------------------------------------------------------
IES77          TTTCAGCAACAGCAAATAAATATTTTAGAGAAAACGGAATAGCAAGAAAGCAATTAGAAAGATAAACAATATGAAGAAAT
CTG23427       --------------------------------------------------------------------------------
CTG35975       TTTAAGCGACAACAAATAAATATTTTAGAGAAAAAGGAAGAGTA------------------------------------
CTG21499       --------------------------------------------------------------------------------
CTG40749       TTTATGCAACAGCAAATAAATATTTTAGATAAAAAGAAATAGCAAGAAAGCAATAAGAAAGATAAACAACATTAAGAAAT
IES128         TTTAAGCAACAGCAAATAAATATTTTAGAGAAAAAGGAATAGCAAGAAA-CAATTAGAAAGATAAACAATATGAAGAAA-
CTG28766       ------------------------------------------------AGCAATTAGAAAGAT-TACAATATGAAGAAAT
IES98          TTTCAACGACAGCAAATAAATATTTTAGAGAAAAAGGAAAAGCAAAAAAGCAATTAGAAAGATAAACAATATGAAGAAAT
CTG10063       --------------------------------------------------------------------------------
AnchoisA   618 TTTAAGCAACAGCAAATAAATATTTTAGAGAAAAAGGAATAGCAAGAAAGCAATTAGAAAGATAAACAATATGAAGAAAT

CTG40749b      --------------------------------------------------------------------------------
IES77          ATTGTCAGAGTGCAAGCAAGAATTTGAGCAGAACATTATTTTAAGAAAAGTGAAGAGAGTGGTAGAGAAGTGGAAGTGAT
CTG23427       --------------------------------------------------------------------------------
CTG35975       --------------------------------------------------------------------------------
CTG21499       --------------------------------------------------------------------------------
CTG40749       ACTGTCAGAGAGCAAGCAAGAATTGGAGCAGAACATTATTTTAAGAAAAGTGAAGAGAGTAGTAGAGAAGTGGAAGTGCT
IES128         ATTGTCAGAGA---GCAAGAATTTGAGCAGAACATTATTCTAAGAAAAGTGAAGAGAGTGGTAGAGAAGTGGAAGTGCT
CTG28766       ATTGTTAGAGAGCAAGCAAGTATTTGAGTAGAGTATTACTTTAAGAAAAGTGA------------------GGAAGTGCT
IES98          ATTGTTAGAGAACAAGCAAAAATTTGAACAGAACATTATTTTAACAAAAGTGAAGAGAGT-GTAGAGAAGTGGAAGTGCT
CTG10063       --------------------------------------------------------------------------------
AnchoisA   698 ATTGTCAGAGAGCAAGCAAGAATTTGAGCAGAACATTATTTTAAGAAAAGTGAAGAGAGTGGTAGAGAAGTGGAAGTGCT

CTG40749b      --------------------------------------------------------------------------------
IES77          AAAGATATATTAAAGAGTTTATGAGG---------------CGGCACTTTGAGTTCCATCATGAGGATTTGAATGAAAAA
CTG23427       --------------------------------------------------------------------------------
CTG35975       --------------------------------------------------------------------------------
CTG21499       --------------------------------------------------------------------------------
CTG40749       AAAGATTTATAAAAGAGTTCATGAGGGAGCAGGATGGGTATCAGCACTTTTAGTTCCATCATAAGAATATGAATGAAGAA
IES128         AAAGATGTATAAAAAAGTTCATGAGGGAGCAGGATGGGTATCGGCACTTTGAGTTCCATCATAAGGATTTGAATGAAGAT
CTG28766       AAAGATGTATAAAAGAGTATATGAGGGAGCTGGATGGGTCTCTTCAATTTGAGTTCCATTATAAAAATTTGAATGAAGAA
IES98          AAAGATGTAT-AAAGATTTCATGAGGGAGCAGGATGGGTATCGGCACTTCGAATTGCATCATAAAGATTTGAATGAAGAA
CTG10063       --------------------------------------------------------------------------------
AnchoisA   778 AAAGATGTATAAAAGAGTTCATGAGGGAGCAGGATGGGTATCGGCACTTTGAGTTCCATCATAAGGATTTGAATGAAGAA

CTG40749b      --------------------------------------------------------------------------------
IES77          GAGAAAAAATTCTTTGACTCGAGGAAGAAGAAAAAAAGTGCTGAAGCAGAATAGTAGCAGGATAAATA------------
CTG23427       --------------------------------------------------------------------------------
CTG35975       --------------------------------------------------------------------------------
CTG21499       --------------------------------------------------------------------------------
CTG40749       GAGA-AATGTTCTTTGACTCGAG---GAAGAAAAAAGGTGCTGAAGCAGAATAGTGGGAGGATGAAAAATGAGATACAAA
IES128         GAGAAAAAGCTCTTGAACTCGAGAAAGAAGAAAAA-AGTGCTGAAGCAGAACAGTAGCAGTATGAAAAATGAGATACCAT
CTG28766       GAGAGAAAGTTCTTTAACTCTAGAAAAAGGAAAAA-GGTGCTGAAGCAGAATAGTAGCAGGATGAAAA-TGAGATACCAT
IES98          GAGATAAAGTTCTTTGACTAGAGAAAGAAGAAAAATGATGCTGAAGCAGAATAGTAGCAGGATGAAAAATGAGATACCAT
CTG10063       --------------------------------------------------------------------------------
AnchoisA   858 GAGAAAAAGTTCTTTGACTCGAGAAAGAAGAAAAAAGGTGCTGAAGCAGAATAGTAGCAGGATGAAAAATGAGATACCAT

CTG40749b      --------------------------------------------------------------------------------
IES77          ---------------------------AATTTTCTACTTC--TTTTTCATAATAATCAATTTCTTC-TTTAGAATTGCTG
CTG23427       --------------------------------------------------------------------------------
CTG35975       --------------------------------------------------------------------------------
CTG21499       --------------------------------------------------------------------------------
CTG40749       AACATATATTAAAAA-ATGCATA-AATAATTTTCTACTTC--TTTTTCATAAGAATCAATTTCTTCTTTTAGAATTGCTG
IES128         AAAATATATAAAAAA--TGCATG-AATAATATTATACTTCTTTTTTTCATAATAAACAATTTCTTC-TTTAGAATTGCTG
CTG28766       ATAATATATAAAAAATATGCATGAAATAATTTTCTACTTA--TTTTTCATAATGATCAATTTCTTC-TTTAGAATTGCTA
IES98          AGAATATATTAAAAA-ATGCATG-AATAATTTTCTACTTC--TTTTTCATAATAATCAATTACTTC-TTTAGAATTTCTG
CTG10063       --------------------------------------------------------------------------------
AnchoisA   938 AAAATATATTAAAAA-ATGCATG-AATAATTTTCTACTTC--TTTTTCATAATAATCAATTTCTTC-TTTAGAATTGCTG

CTG40749b      --------------------------------------------------------------------------------
IES77          TCTCTATTCTATTTCCTCTTAAATTCCTTAATTCATTATTTCTAATTCTTCAGTTAACTTTGATATTTTTAGCAAC--AT
CTG23427       --------------------------------------------------------------------------------
CTG35975       --------------------------------------------------------------------------------
CTG21499       --------------------------------------------------------------------------------
CTG40749       TCTCTATTCTGCCTCCTCTTAACCTCCTTAATTCACTATTTCCAATTCTTCAGTTAACTTTGATAATTTCAGCACC---T
IES128         TCTCTATTCTACTTCCTCTTAAATTCCTTAATTCACTATTTCTAATTCTTCAGTTTACTTTGATAATCTTAGCAAC--TT
CTG28766       TCTCTATTCTACTTCCTCTTAAA-TCCTTAATTCACTATTTCTAATTCTTTAGTTAACTTTGATAATTTCAGCAACTTTT
IES98          TCTCTATTCTACTCCCTCTTAAATTCCTTAATTCACTTTTTCTAATTTTTCAGTTAACTTT-------------------
CTG10063       --------------------------------------------------------------------------------
AnchoisA  1013 TCTCTATTCTACTTCCTCTTAAATTCCTTAATTCACTATTTCTAATTCTTCAGTTAACTTTGATAATTTTAGCAAC--TT
```

```
CTG40749b        --------------------------------------------------------------------------------
IES77            TTTTGCTTAAATAAGTTCTTTGCGAGC----------TAAGCAGCATTTTATTTAAATATCTCAACTTACTCCTTTATTC
CTG23427         --------------------------------------------------------------------------------
CTG35975         --------------------------------------------------------------------------------
CTG21499         --------------------------------------------------------------------------------
CTG40749         TTTTGCCTAAATGAG----------------------TTAGCTGCATTTTATTTAAATA-----------------GTTC
IES128           TTTTGCTTAAATGAGTTCTTTGCGAGCTGACTTTTTCTTAGCTGCATTTTATTTAAATATCTCAACTTACTCATTTGTTC
CTG28766         TTTTGCTGAAACAAGTTCTTTGCGAGCTAACTTTTTCTTAGCTGCATTTTATTTAAATATACCCACTTACTCCTTTGTTA
IES98            ----------------------------------TTCTTAGCTGCATTTTATTTAAATATATCAACTTACTCCTTTGTTC
CTG10063         --------------------------------------------------------------------------------
AnchoisA    1091 TTTTGCTTAAATGAGTTCTTTGCGAGCTAACTTTTTCTTAGCTGCATTTTATTTAAATATCTCAACTTACTCCTTTGTTC

CTG40749b        --------------------------------------------------------------------------------
IES77            TGC-CAAATATCTTGGCCTTTTATTTCTTCCGATAGAAGTGTCTAATTAGCGAATCAATCTTGTGTTGATAAATATTTTA
CTG23427         --------------------------------------------------------------------------------
CTG35975         --------------------------------------------------------------------------------
CTG21499         --------------------------------------------------------------------------------
CTG40749         TTCGAAAATATCTCAGCCTTTTATTTCTTTCGATCGAAGTGTTTAATTAGCGAATCAACCTTATGTTGATCAATATTTTA
IES128           TGCAAAAATATCTCAG-CTTTTATTTCTTCCGATAGAAGTTTCTAATTGGCGAATCAACCTTATATTGATAAATATCTTA
CTG28766         TGCAAAAATATCTCAGCCTTCTATTTCTT-CGATAGAAGTGTTTAATTAGCGAATCAACCTTATGTTGATAAATATTTTA
IES98            TGCGAAAATATCTCAGCCTTCTATTTCTTCCGATAGAAGTGTCTAATTAGCGAATAGAACTTATGGTAATAAATATTTTA
CTG10063         --------------------------------------------------------------------------------
AnchoisA    1171 TGCGAAAATATCTCAGCCTTTTATTTCTTCCGATAGAAGTGTCTAATTAGCGAATCAACCTTATGTTGATAAATATTTTA

CTG40749b        --------------------------------------------------------------------------------
IES77            CATTAAATCATACTGCGTGAGATTTGATTAATCCTGC-CATTAGGGTTTGTAAATAAGGAGTGATTTGGTTCTATTGTTT
CTG23427         --------------------------------------------------------------------------------
CTG35975         --------------------------------------------------------------------------------
CTG21499         --------------------------------------------------------------------------------
CTG40749         CATTAAATCATCCAGCGTGAGATTTGA-TAATCTTGT-----AGGGTTTTTAAATAAA-AATGATTTGGTTCTATTGTTT
IES128           CATTAAATCATCCTTTGTGAGATTTGATTAATCCTGCACTTTACGGTTT-TAAATAAAGAATTATTTGGTTCTATTGTTT
CTG28766         CATTAAATCATCCTATGTGAGATTTTATTAATCCTTCACTTTAGGGTTTTTAAATAAAGAATATTTTGATTCTATTGTTT
IES98            CATT-AATCATCCTGCGTGAGATTTGATTAATCCTGC-------------------------------------------
CTG10063         --------------------------------------------------------------------------------
AnchoisA    1251 CATTAAATCATCCTGCGTGAGATTTGATTAATCCTGCACTTTAGGGTTT-TAAATAAAGAATGATTTGGTTCTATTGTTT

CTG40749b        --------------------------------------------------------------------------------
IES77            TAAGAAAATTAACTGCCAATGTTTTATCAAGGTAGCTTTCCATCAGAAAAGCCAAGCTCTTAACAATTAAAATTCTTTTT
CTG23427         --------------------------------------------------------------------------------
CTG35975         --------------------------------------------------------------------------------
CTG21499         --------------------------------------------------------------------------------
CTG40749         TAAGAACGTTAACTGCCCATGTTTTATCAAGATAA-TTTCCATCAGAAATG-CAAGCTCTTAACCATTAAGCTTCGTTTT
IES128           TAAGAACATTAACTGCCCATGTTTTATCAGGGTAG-TTTCCATCAGAAAAGCCAAGCTCTTAACCATTAAGCTTGTTTTT
CTG28766         TAAGAACATTAACTGCCCATG-TTTAACAGGGTAG-TTTCCATCAGAGAAGCCAAGCTCTTAATCATTAAGCTT-TTTTT
IES98            --------------------------------------------------------------------------------
CTG10063         --------------------------------------------------------------------------------
AnchoisA    1330 TAAGAACATTAACTGCCCATGTTTTATCAGGGTAG-TTTCCATCAGAAAAGCCAAGCTCTTAACCATTAAGCTTCTTTTT

CTG40749b        --------------------------------------------------------------------------------
IES77            ATTTTCATGTAAAATTTCTTATTTGGTAGCTGATCTCAGCAGTTATCCTATTTGAACTTTTTTTGATTCTAAGCCAAAGT
CTG23427         --------------------------------------------------------------------------------
CTG35975         --------------------------------------------------------------------------------
CTG21499         --------------------------------------------------------------------------------
CTG40749         AATTTCATATAAAATTTCTTCTTTAGTAG-TGATCTCAGAAGTCATCCAATTTCATCTTTTTTTGATTCCAAGCCAAACA
IES128           AATTTCATGTAAAATTTCTTCTTT-GTATCTGATCTCAGCAGTTATCCTATTTCAACTTTTTTTGATTCTAAGCCAAAGC
CTG28766         AATTTCATGTAAAATTTCTTCTTTATTGGCTGAGCTCAACAGTTATCCTATTTAAACTTTTTTCT-CTCTAAGCCAAAGC
IES98            ---------------------------------------------TCCTATTTGA--TTTTTTTGACTCTAAGACAACGC
CTG10063         --------------------------------------------------------------------------------
AnchoisA    1409 AATTTCATGTAAAATTTCTTCTTTAGTAGCTGATCTCAGCAGTTATCCTATTTGAACTTTTTTTGATTCTAAGCCAAAGC

CTG40749b        --------------------------------------------------------------------------------
IES77            ATGATTTAGTATCAACTTTGAGCAAATAATACGCTGTCACACCTTAAGAATGAATTGCAGGTAAATACTAACCTGCCAAG
CTG23427         --------------------------------------------------------------------------------
CTG35975         --------------------------------------------------------------------------------
CTG21499         --------------------------------------------------------------------------------
CTG40749         ATT------------TTTTGAGCAAATATTATGCTGTTACACCTGAGGAATGAATTGTGGGTATATAATATCATACGAAG
IES128           ATT--TTAGTAGCAACTTTGAGCAAATATTACGCTTTTACATCTGAAGAATGAATTGCAGGTAAATAATATCCTGCC-AG
CTG28766         ATTTATTAATAGCAACTTTGAGCAAATATTAC-CTTTTACACCTGAAGGATGAATTGCATTAGAGTAATATCCTTTCAAG
IES98            ATT-TTTAGTAGCAACTTTGATCAAAGATTACGCTGTTACACCTGAAGAAATAA-----------TATCCCCCTGCCAAG
CTG10063         --------------------------------------------------------------------------------
AnchoisA    1489 ATTATTTAGTAGCAACTTTGAGCAAATATTACGCTGTTACACCTGAAGAATGAATTGCAGGTAAATAATATCCTGCCAAG

CTG40749b        --------------------------------------------------------------------------------
IES77            ATTAAGCCAAAGTACATGGATTCAACAAAACCAGTTGCTTGATTAGCTTAG-AAATGGAAATCATGATG-AATTTTGGAA
CTG23427         --------------------------------------------------------------------------------
CTG35975         --------------------------------------------------------------------------------
CTG21499         --------------------------------------------------------------------------------
CTG40749         ATTAAGCCATTGTACATAGATTTAACAGAACCAATCACTTGATTAGTTTACAAAATGGAAATCATGATG-AATTTCGAAA
IES128           ATTAACCAATAGTACATAGATTCAACAGAACCAGTTGTTTGATTAGCTTAGAAAACGGAAATCAAGATG-AATTTCGAAA
CTG28766         ACTAAGCTATAATACATAAAATCAAGGGAACCAGTTGAATGATTAGC-----AAATGGAAATCATGATGAAATTTCAATA
IES98            ATTAAGCCA-----------------------------------------------------------------------
CTG10063         --------------------------------------------------------------------------------
AnchoisA    1569 ATTAAGCCATAGTACATAGATTCAACAGAACCAGTTGCTTGATTAGCTTAGAAAATGGAAATCATGATG-AATTTCGAAA
```

```
CTG40749b        --------------------------------------------------------------------------------
IES77            GCATGTTTAATTATTCAAACAACGCGATAGTAAATTTTTATTGA-AAAATAAGTTCTTTTTTAACCAATCAATTTGAAAG
CTG23427         --------------------------------------------------------------------------------
CTG35975         --------------------------------------------------------------------------------
CTG21499         --------------------------------------------------------------------------------
CTG40749         GTTTGTTTAAGTACTTGAACAATGTGATAGTAAATTTTTATTGA-AAAAAATATTCTGTTTTAACCAATCAATTTCAAAG
IES128           GTTTGTTTAATTATTAAAACAATGCGATAGTAAATTTTTATTGAAAAAAAAAAATCTTTTTTTACCAATCAATTTCAAAG
CTG28766         GTTTGTTTAATTATTCAAACAATGCAATA---------------------------------------------------
IES98            --------------------------------------------------------------------------------
CTG10063         --------------------------------------------------------------------------------
AnchoisA    1648 GTTTGTTTAATTATTCAAACAATGCGATAGTAAATTTTTATTGA-AAAAAAAATTCTTTTTTAACCAATCAATTTCAAAG

CTG40749b        --------------------------------------------------------------------------------
IES77            C-AATTGATTTT-TAAAGCAGAAAACAATTTAGAATAAATTAAAGAAAAATACTTAAATGATTTATAATTT-AATACAAA
CTG23427         --------------------------------------------------------------------------------
CTG35975         --------------------------------------------------------------------------------
CTG21499         --------------------------------------------------------------------------------
CTG40749         C-AATTGATTTTAAAAAGCAGAAAACAATTTAGAATAAATTAAGAAAAATTA-TTAAATGATTAATAATT----------
IES128           CAAATTAATTTT-AAAAGCAGAAAACAATTTAGAATAAATTAAAGAAAAATA-ATAAATGATTTATAATTTTAATGCAAC
CTG28766         --------------------------------------------------------------------------------
IES98            --------------------------------------------------------------------------------
CTG10063         --------------------------------------------------------------------------------
AnchoisA    1727 C-AATTGATTTT-AAAAGCAGAAAACAATTTAGAATAAATTAAAGAAAAATA-TTAAATGATTTATAATTTTAATGCAAC

CTG40749b        --------------------------------------------------------------------------------
IES77            TTAAATACTGCTTTTTTATTGGATTATATTTATAGTGAGAATTTT---TTTTGAATTAAAGTAAAATATTTTGATTAAAT
CTG23427         --------------------------------------------------------------------------------
CTG35975         --------------------------------------------------------------------------------
CTG21499         --------------------------------------------------------------------------------
CTG40749         --------------------------------------------------------------------------------
IES128           TTA----------TTTTATTGAATTATATTTATAATGAGGATTTTTTGTTTTGAATTGGAG-AAAATATTTTGATTCAAT
CTG28766         --------------------------------------------------------------------------------
IES98            --------------------------------------------------------------------------------
CTG10063         --------------------------------------------------------------------------------
AnchoisA    1804 TTAAATACTGCTTTTTTATTGAATTATATTTATAATGAGGATTTTTTGTTTTGAATTGGAG-AAAATATTTTGATTCAAT

CTG40749b        --------------------------------------------------------------------------------
IES77            TTT---------------TTTTTTATTGGCGCTCAAATTTTTATGCCAGAAATTTAATTTTTCCACATTTAATGTAAATA
CTG23427         --------------------------------------------------------------------------------
CTG35975         --------------------------------------------------------------------------------
CTG21499         --------------------------------------------------------------------------------
CTG40749         --------------------------------------------------------------------------------
IES128           TTTGATTATTGATTCGAATTTTAGATTATAGCTCCAATTTTTATGCAAGAAATTTAATTTTTCCACATTTAATGTAAATA
CTG28766         --------------------------------------------------------------------------------
IES98            --------------------------------------------------------------------------------
CTG10063         --------------------------------------------------------------------------------
AnchoisA    1883 TTTGATTATTGATTCGAATTTTAGATTATAGCTCCAATTTTTATGCAAGAAATTTAATTTTTCCACATTTAATGTAAATA

CTG40749b        --------------------------------------------------------------------------------
IES77            GAAACTCCAGCTTCTTCATTAGTGTACATATATTATTTGCTATAGCAGGCCTTATGCTTAAATAACAATATTTATACACA
CTG23427         --------------------------------------------------------------------------------
CTG35975         --------------------------------------------------------------------------------
CTG21499         --------------------------------------------------------------------------------
CTG40749         --------------------------------------------------------------------------------
IES128           GAAACTCAAGCTTCTTCATCTCTGTACATATATTATTTGCTATAGCAGGCCATATACCTAAACAACAATATGAAGACACA
CTG28766         --------------------------------------------------------------------------------
IES98            --------------------------------------------------------------------------------
CTG10063         --------AGCTTCTTCATCTCTGTACATATATTATTTGCTATAGCAGGCCATATGCTTAAACAACAATATGTAGACACA
AnchoisA    1963 GAAACTCAAGCTTCTTCATCTCTGTACATATATTATTTGCTATAGCAGGCCATATGCTTAAACAACAATATGTAGACACA

CTG40749b        --------------------------------------------------------------------------------
IES77            TATGCGATGATCATTGCTAATAATTT-ACTAAACCTATGTTTATGAAAAAAAAATTTTAATCAAAACCAAATAGCAAATG
CTG23427         --------------------------------------------------------------------------------
CTG35975         --------------------------------------------------------------------------------
CTG21499         --------------------------------------------------------------------------------
CTG40749         --------------------------------------------------------------------------------
IES128           TTTGCAATGATCATTGCTAATAATTTTACTAAACCTATGTTTCTGAAAAAAGACTTTTAATCAAAACCAAATAGCAAATG
CTG28766         --------------------------------------------------------------------------------
IES98            --------------------------------------------------------------------------------
CTG10063         TATGCGATGATCATTGCTAATAATTTTACTAAACCTATACTTATGAAAAAAGACTTTTAATCAAAGCCAAGTATCAAATG
AnchoisA    2043 TATGCGATGATCATTGCTAATAATTTTACTAAACCTATGTTTATGAAAAAAGACTTTTAATCAAAACCAAATAGCAAATG

CTG40749b        --------------------------------------------------------------------------------
IES77            CTGGAATTGTTTACTCTAGAGGATGCTCATAAAATTATAAAACAAAA-TTAGATTCCAGAAGGGAAGTAAAGTTGAGAAA
CTG23427         --------------------------------------------------------------------------------
CTG35975         --------------------------------------------------------------------------------
CTG21499         --------------------------------------------------------------------------------
CTG40749         --------------------------------------------------------------------------------
IES128           CTAGAACTGCTTACTTTAAAGGATGCTCATAAAATTATAAAACAAAG--TAGAATCCACAAGGGAAGTAAAATTGAGAAA
CTG28766         --------------------------------------------------------------------------------
IES98            --------------------------------------------------------------------------------
CTG10063         TTGGAACTGCTTACTCTAGGTGATGCTCATAAAATTATAAAACAAAAATTAGAATCCACAAGGGAAGTAAAGTTTAGAAA
AnchoisA    2123 CTGGAACTGCTTACTCTAGAGGATGCTCATAAAATTATAAAACAAAAATTAGAATCCACAAGGGAAGTAAAGTTGAGAAA
```

```
CTG40749b         --------------------------------------------------------------------------------
IES77             AAATATGAACAAATTTGAGAAAAAAAGCAACTTCAAATCATTAACTTAAAAAAATTTTGCAGAAAA----TTGC-TTAGA
CTG23427          --------------------------------------------------------------------------------
CTG35975          --------------------------------------------------------------------------------
CTG21499          ---------------------------CAAAATCAAATCATTAACTTTAAAA----TTGCAGAAAATAAATTGATTTAGA
CTG40749          --------------------------------------------------------------------------------
IES128            AAA-ATAAA-AAATTTGAGAAAAA-AGCAACTTCGAATCATTAATTTTAAAA----TTATAGAAAA----TTGCTTTAGA
CTG28766          --------------------------------------------------------------------------------
IES98             --------------------------------------------------------------------------------
CTG10063          AAA----------------------AGCAATTTCAAATCATTAACTTTAAAA----TTGCAGAAAA----TATCTTTAGA
AnchoisA     2203 AAATATAAACAAATTTGAGAAAAAAAGCAACTTCAAATCATTAACTTTAAAA----TTGCAGAAAA----TTGCTTTAGA

CTG40749b         --------------------------------------------------------------------------------
IES77             ATTAGGAAATTCGGTAAAACCTGCAA---TTTTGGCA-GGATATTTAGGAATTTCTCTTGGAATAGCTAAGGATTTATAA
CTG23427          --------------------------------------------------------------------------------
CTG35975          --------------------------------------------------------------------------------
CTG21499          ACTAGAAAATTCGGTAAAACTTAAAATTTTTTTGGCA-GGATATTTAGGAATTTCTATTGGAAGAACTAATTATTGAGAA
CTG40749          --------------------------------------------------------------------------------
IES128            ATCAGGAAATTTTGTAAAACCTGCAA---TTTTGGCAAGGATATTTAGGAATTTCTATTATGAGAGCTAAATATTTATAA
CTG28766          --------------------------------------------------------------------------------
IES98             --------------------------------------------------------------------------------
CTG10063          ATGAGGAAATTCGGTAAAATCTGGAA---TTTTGGCA-GGATATCTAGGAA-TTCTATT---------------------
AnchoisA     2275 ATTAGGAAATTCGGTAAAACCTGCAA---TTTTGGCA-GGATATTTAGGAATTTCTATTGGAAGAGCTAAATATTTATAA

CTG40749b         --------------------------------------------------------------------------------
IES77             ATGTATGTTTTACAGCAACCTAATATTTACAAAGACAATAACTTGCTTT----ATTGATGCATAACAAAAATTTTTGATT
CTG23427          --------------------------------------------------------------------------------
CTG35975          --------------------------------------------------------------------------------
CTG21499          AATGATGTT-TACA-CAACCTG--ATTTACAAGGACAATAACTTGCTTTTACATTTGATGCTTAACATAAATTTAGGA-T
CTG40749          --------------------------------------------------------------------------------
IES128            AAGTATGTT-TACAGCAAGCTG--ATTAACAAAGACAATAACTTGCTTTTAAATTTGATGCATAACATAAAATGTGGATT
CTG28766          --------------------------------------------------------------------------------
IES98             --------------------------------------------------------------------------------
CTG10063          --------------------------------------------------------------------------------
AnchoisA     2351 AAGTATGTTTTACAGCAACCTG--ATTTACAAAGACAATAACTTGCTTTTAAATTTGATGCATAACATAAATTTTGGATT

CTG40749b         --------------------------------------------------------------------------------
IES77             T--AAATTCGTAGCTCAAGTAAATTTTGCTCGCTTAGGCAGTGTAAAATAAATCTATTAAAAACTTAGAGCAAATTTTCA
CTG23427          --------------------------------------------------------------------------------
CTG35975          --------------------------------------------------------------------------------
CTG21499          TAAAAATTTGTATCTAAAGATAATGTTACTC--TTGAGCAGTGCAAAATAAATCTATTAAGAA-TTGTAGCAAATTTTCA
CTG40749          --------------------------------------------------------------------------------
IES128            TAAAAATTTGTAGCTCAAGAAAATATTGCTCTTTTGAGCAGTGCAAAATAAATCTATTAAGAATTTATAGCAAATTTTCA
CTG28766          --------------------------------------------------------------------------------
IES98             --------------------------------------------------------------------------------
CTG10063          --------------------------------------------------------------------------------
AnchoisA     2429 TAAAAATTTGTAGCTCAAGAAAATGTTGCTCGCTTGAGCAGTGCAAAATAAATCTATTAAGAATTTATAGCAAATTTTCA

CTG40749b         --------------------------------------------------------------------------------
IES77             TATTTGCAACTAGCTACCGCAG-TTCATTATTTTTTTTAGTAGTTTAAACTACTTTGGCTTTGTACCTATCGCTTATTTA
CTG23427          --------------------------------------------------------------------------------
CTG35975          --------------------------------------------------------------------------------
CTG21499          TATTTGCAAATAGCAACAGCAGTTTCATTAAATTTTTAGATAATTTAAACAGCTTGGGTTATATACATGTCGCTTATTTA
CTG40749          --------------------------------------------------------------------------------
IES128            TATTTGCAAATAGCAACTGCAGTTTCATTAATTTTTTAAGTAGTTTAAACAGCTTTGGTCATATACCTATCGATTATTTT
CTG28766          --------------------------------------------------------------------------------
IES98             --------------------------------------------------------------------------------
CTG10063          --------------------------------------------------------------------------------
AnchoisA     2509 TATTTGCAAATAGCAACTGCAGTTTCATTAATTTTTTAAGTAGTTTAAACAGCTTTGGTTATATACCTATCGCTTATTTA

CTG40749b         --------------------------------------------------------------------------------
IES77             GTTGCGTTAATTTTAAATCTCAAATTAAATGTTCTTTAGACAGGAGGTTTGAATTTGC-ATACAATTAGCTACTCTTTTA
CTG23427          --------------------------------------------------------------------------------
CTG35975          --------------------------------------------------------------------------------
CTG21499          GTTGCGTTAATTTTAAATCTCAAAATTAATGTTCTTTAGACAGAAGGTTTGAATTTGCAAAATAATTAGCTACTCTTTTA
CTG40749          --------------------------------------------------------------------------------
IES128            GTTGCGTTAA-TTTAAGTCTCAAACTAAATGTTCTTTAGACAGAAGGTTTGATTTTGC-AAATAATTAGCTACTCTTTTA
CTG28766          -----------------------------------------------------------------TTATCTACTCTTTTA
IES98             --------------------------------------------------------------------------------
CTG10063          --------------------------------------------------------------------------------
AnchoisA     2589 GTTGCGTTAATTTTAAATCTCAAACTAAATGTTCTTTAGACAGAAGGTTTGAATTTGCAAAATAATTAGCTACTCTTTTA

CTG40749b         --------------------------------------------------------------------------------
IES77             AATTCAAGATAAAAAATGGTTTACATTAATGAGTGCAGTTTTTGGAGACATTTATTAGTTGCAAGAGGTTGTTAATAAAA
CTG23427          --------------------------------------------------------------------------------
CTG35975          --------------------------------------------------------------------------------
CTG21499          ATTC--AATTAAAAAATTGTTTACATTGATGAGTGCAG-TTTGGGAGATATTTAAAAATTGCAAAAGGTTAGTAAAAAAA
CTG40749          --------------------------------------------------------------------------------
IES128            AATTAAAAATAAAAAATTGTTTACATTGCTGAGTGCGCTTTTGGGAGAAATTTAAAAGTTGCAAGAGGTTGGTAAAAAAA
CTG28766          AATTCAAAATAAAAAATTGCTTACATTGATAAGTGCAGTTTTGGCAGAAATTTA-AAGTTGCAAGAGGTTGGTAAAAAAT
IES98             --------------------------------------------------------------------------------
CTG10063          --------------------------------------------------------------------------------
AnchoisA     2669 AATTCAAAATAAAAAATTGTTTACATTGATGAGTGCAGTTTTGGGAGAAATTTAAAAGTTGCAAGAGGTTGGTAAAAAAA
```

```
CTG40749b        ----------------TTGCAAATTAAATCAAGCTCATCAAAAAATAAATGTGTTATTGGAGCTATATCTTGTGACGTGT
IES77            GAACACTTTTCCATTATTGCAAATTAAATCAAGCTCATCAAAAAATAAATGTGTTATTGGAGCCATAGCTTGTGACTGGT
CTG23427         --------------------------------------------------------------------------------
CTG35975         --------------------------------------------------------------------------------
CTG21499         -AACACGTTTCCATAATTGCAAATTAAATCAAGCTCATCATAAAATAAATATATTATTGGAGCTATAGCTTGTAAAGGGT
CTG40749         --------------------------------------------------------------------------------
IES128           GGGCACTTT-CCATTTTTGCAAATTAAATCAAGCTCATC-AAAAATCAATATGTTATTGGAGCTATAGCTTGTGACGGGT
CTG28766         GA-CGCTTTTCCATTATTGCAAATTAAATCAAGCTCATC-AAAAATAAATTTGTTATAAGAACTATAGCTTGTTATAGGT
IES98            --------------------------------------------------------------------------------
CTG10063         --------------------------------------------------------------------------------
AnchoisA    2749 GAACACTTTTCCATTATTGCAAATTAAATCAAGCTCATCAAAAAATAAATGTGTTATTGGAGCTATAGCTTGTGACGGGT

CTG40749b        TTATTGCTTAT-AATGTGTTATAGGAAATGTT-ATCAATAGGTTTTGGGAGAATTTTT-GAATGAACCTCTAAGGCTGCT
IES77            TTATTGCTTACAAATGTATTATAGGAAATGTTTATCAATAGGTTTTTAGAGAATTTTT-GAATGAACTTCTAAGGCTCCT
CTG23427         --------------------------------------------------------------------------------
CTG35975         --------------------------------------------------------------------------------
CTG21499         TTATAGCTTAAAAATGTGTTATAGG-AATGTTAATCAATAGGATTTTGGAGAATATTT-TAATGAACTTTTAAGGCTGCT
CTG40749         --------------------------------------------------------------------------------
IES128           TTAATACTTACAAATGTGTTATAGGAAATGTTAATCAATAGGTGTTTGGAGAATTTTTTGAATGAACTTCTAA-GCTGCT
CTG28766         TTATTGCTTGCAAATGTG--------AATTTTAATCGATAGGGTTTTGGAGAATTTTT-GAATGAACTTCTAAAGCTGAT
IES98            --------------------------------------------------------------------------------
CTG10063         --------------------------------------------------------------------------------
AnchoisA    2829 TTATTGCTTACAAATGTGTTATAGGAAATGTTAATCAATAGGTTTTTGGAGAATTTTT-GAATGAACTTCTAAGGCTGCT

CTG40749b        TTAGCAGAATAGTGAAGAAAATAATTTTTGCT-GGTGCTGGACAATGTATTTATCCATCACACATTAT-AAATTTTGGAT
IES77            TTAGCAGAATAGTGAAGAAAATAATTTTTTCTTTGTCCTGGAAAATGCATCTATCCATCGCATCTC---AAATTTATGAT
CTG23427         --------------------------------------------------------------------------------
CTG35975         --------------------------------------------------------------------------------
CTG21499         TTAGCAGAATAGTGAAGAAAATAATTTTTGCTCGGTGCTGGACGATGCATCTATTCATCGCACATCGC-AAATTTTGGAT
CTG40749         --------------------------------------------------------------------------------
IES128           TTAGCAGAATAGTGAAGAAAATAATTTTTGCTTGGTGCTGGACGATGCATATATCCATCGCACATCAC-AAATTTTGGAT
CTG28766         TTATTAGAATAGTGAAGAAAATAATTTTTACTTTTTGCTGGACAATGCATCTATCCATCTTACATCACAAAATTTTGGAT
IES98            --------------------------------------------------------------------------------
CTG10063         --------------------------------------------------------------------------------
AnchoisA    2908 TTAGCAGAATAGTGAAGAAAATAATTTTTGCTTGGTGCTGGACAATGCATCTATCCATCGCACATCAC-AAATTTTGGAT

CTG40749b        TAATTGTCTTATGTGAACTATCTATTTCTCCTACAATATAGTCCTGA-GCTTAATTGCATTGAAA-GCTCTGGGGGAGT-
IES77            TAATTGTCTTATATGAATTATTTATTTCTCCCACCATATAGTCCTTT-GCTTAATTGCATTGAAAAGCTATGGGG-AGTA
CTG23427         --------------------------------------------------------------------------------
CTG35975         --------------------------------------------------------------------------------
CTG21499         TAATTGTCTTATGTGAACTGTTTATTTCTCCCATCATATAGTCCTTAGGCTTAATTGCATTTAAAAGCTATGGGG-AGTA
CTG40749         --------------------------------------------------------------------------------
IES128           TAATAGTCTTATGTGGACTATTTATTTTTCCCACCATATAGTCCTTA-GCTTAATTGCGTTGAAAGGCTATGGGG-AGTA
CTG28766         TAATTGTATTATGTGAACTATTTATTTCTCCCACCAGATAGTCCTTA-GATTAATTGCATTGAAAAGTTATGGGG-AAAA
IES98            --------------------------------------------------------------------------------
CTG10063         --------------------------------------------------------------------------------
AnchoisA    2987 TAATTGTCTTATGTGAACTATTTATTTCTCCCACCATATAGTCCTTA-GCTTAATTGCATTGAAAAGCTATGGGG-AGTA

CTG40749b        -------------------------ACATCGTAACAACATATTAAGAATTTCACAACATTA-------------------
IES77            GCAAAACAAAAACTCTCCAAAATGTACTTCGCAACATCATTTTAAGAACTTCACAATATTCATTTTGTGCTTAACTCAAT
CTG23427         -----------------------------------------------------CAACTTTCATTATTTTCTTAACCCAAT
CTG35975         --------------------------------------------------------------------------------
CTG21499         AC-AAACAAAAGCTCTCCAAAATGCACTTCACAATAACATTTTAAGAATTTCGCAAAATTCGTTTTGTTTTTAACTAAAT
CTG40749         --------------------------------------------------------------------------------
IES128           GCAAAACAAAAGCTCTCCAAAATGCACTTCGCAACAACATTTTAAGAATTTCACAACATTCATTTTGTGCTTAACTCAAT
CTG28766         GCAAAATAAAAGCTATCTAAAATACACTTCGCAACAACATTTTAAGAATTTCACAAAATGCATTTTGTGCTTAACTCAAT
IES98            --------------------------------------------------------------------------------
CTG10063         --------------------------------------------------------------------------------
AnchoisA    3065 GCAAAACAAAAGCTCTCCAAAATGCACTTCGCAACAACATTTTAAGAATTTCACAACATTCATTTTGTGCTTAACTCAAT

CTG40749b        -----------------------------------------------------------TACATAGGCAGCCTTTTTGAT
IES77            TACAGCTAATTAAGTGCAAAATATTTGCACCTTTAACTTGAAATTCTTGCAAGCTTACTTACATAGACAGCAGTTTTGAT
CTG23427         TGCAACAAATTAAGTGCAAAATATTTCCACTTTTAGCTTGAAATTCTTGCAAGCATGCTTGCATAGACAAAAGTTTTGAT
CTG35975         -----------------------------------------------------------TA-ATCGACAACAGTTTTTAT
CTG21499         TACAGCTAATTAAGGGCAAAATATTTGCACCTTTAACTTGAAATTCTTGCAAGCTTGCTTACATAGACAGCAG-TTTGAT
CTG40749         --------------------------------------------------------------------------------
IES128           TACAGCTAATTAAGTGCAAAATATTTGCACATTTAACTTGAAATTCTTGCAAGCTTGCTTGCATAGACAATAGTTTTGAT
CTG28766         TACAGCTAATTAAGTGCAAAATATTTGCATCTTTAACTTGAAATTTCCGAATGCTTGCTTACGTAAACAGCAGTTTTTAT
IES98            --------------------------------------------------------------------------------
CTG10063         --------------------------------------------------------------------------------
AnchoisA    3145 TACAGCTAATTAAGTGCAAAATATTTGCACCTTTAACTTGAAATTCTTGCAAGCTTGCTTACATAGACAGCAGTTTTGAT

CTG40749b        TAATTATATTTTCATTAAAAACATAAGGTATTCAATGCAAACAATTTACAATAAAAA-TCTCTTCACTTTTGATA---GT
IES77            TAGTTATATTTTAATTTGA-------------------------------------------------------------
CTG23427         GAATTATATTTTCATTTAAACCATAAAATATTCAATAAAAGCAATTAACAATAAAAA-TCTCTACACTTTTCTTATA-GC
CTG35975         TAATTATATTTTCGTTTAAA------------------------------------------------------------
CTG21499         GAATTATATTTTCATTTAAACCATAAAACATTCAATGCAAGCAATTAACAATAAAAAATCTTTACTCTTTTCGTAAAGGC
CTG40749         --------------------------------------------------------------------------------
IES128           TAATTATATTTTCATTTAA-------------------------------------------------------------
CTG28766         TAATTATATTTTCATATTA------AAATATTCAATGCATGCAATTAACAATAAAAATTCTCCACTATTTTCTTTTC-GA
IES98            --------------------------------------------------------------------------------
CTG10063         --------------------------------------------------------------------------------
AnchoisA    3225 TAATTATATTTTCATTTAAACCATAAAATATTCAATGCAAGCAATTAACAATAAAAATTCTCTACTCTTTTCTTATA-GC
```

```
CTG40749b       GACCGTGTGCGAAAGCAGACTTTCGTCAGCTATACTTAATACTATCGAGCGGATAG---TAAGAG-AGAGTTTAGAATCG
IES77           --------------------------------------------------------------------------------
CTG23427        GATCGTGTGCTAAAGCAGACTTTCGTCAGTTATACTTAACGCTATCGAGAAGA----------------GTTTCGAAT-G
CTG35975        --------------------------------------------------------------------------------
CTG21499        GACATTGTGCAATAGCAGACTTTCGTCAGTTATACTTAACACTATCGAGCAGATATTAATAAGAGCACAGTTTCGTATCG
CTG40749        --------------------------------------------------------------------------------
IES128          --------------------------------------------------------------------------------
CTG28766        AACAGAGTGCAAAAGCAGACTT------GTCAGACTTAACACTATCGAGCAGATATTAATCAGAGTAGAGTTTCGAATCT
IES98           --------------------------------------------------------------------------------
CTG10063        --------------------------------------------------------------------------------
AnchoisA   3304 GACAGTGTGCAAAAGCAGACTTTCGTCAGTTATACTTAACACTATCGAGCAGATATTAATAAGAGCAGAGTTTCGAATCG

CTG40749b       GGGGACAGAATGGGATCCAAAACAATCGCTCCTTAACCTTTCCAGCCTATTCTCAATACTATTAGACTCTGATTTAATTA
IES77           ----------------------------CTCCTTAACCTTTCCAGCCTATTCTCAATACTATTAGACTCTAATTTAATTA
CTG23427        GGGGACAGGATGGGATTTAAAACACTCGCTCCTTAACCTCTCAAGCCTATTCTCAATACTATTTAACTCTGATTTAATAT
CTG35975        --------------------------------------------------------------------------------
CTG21499        G-------------------------------------------GCCTATTCTCAATACTATTAGACTCTGTTTTAATTA
CTG40749        --------------------------------------------------------------------------------
IES128          ----------------------------------GACCTTTCCAGCCTATTTTCAATACTATTAGACTTTGATTTAATTA
CTG28766        AGGTACAGGGTGGGATCTACAACACTTGCTCCTTAACCTTTCCGGCCAATTCTCAATACTATTAGACACTGATTTAATTT
IES98           --------------------------------------------------------------------------------
CTG10063        --------------------------------------------------------------------------------
AnchoisA   3384 GGGGACAGGATGGGATCTAAAACACTCGCTCCTTAACCTTTCCAGCCTATTCTCAATACTATTAGACTCTGATTTAATTA

CTG40749b       TATATTTTAAAATTATGATAAAATTAAATTCTGAGTTTGGTGTAACTCATTAAATTTTATACAACCATATATGATTTATA
IES77           TATATTTTTAAATT-TCATAAAATTAAATTATGGTTTTGGGGTAAGTCATTAAATATTGTATCATTATATATAATTTATA
CTG23427        TATATTTTAAAGTT-TGATAAAATTAAAA-ATGGGTTTGGGGTAATTCATTAAAT------------ATGTATAATTTAT-
CTG35975        --------------------------------------------------------------------------------
CTG21499        TATATTT-AAAATT-TGATAAAATTAAATTATAGGTTTGGGGTAATTGATTGAATATTATATAATTATTTATAATTTATA
CTG40749        --------------------------------------------------------------------------------
IES128          TATATTTTAAAATT-TGATAAAATTAAATTATGGGTTTGGGGTAATTCATTAAATATGATATAATTATATATAATTTATA
CTG28766        GACATTTATAAATT-TGATAAAATTAAACTATGGGTTTGGGGTAATTCATTAAATATTATATAATCATATATAATTTATA
IES98           --------------------------------------------------------------------------------
CTG10063        --------------------------------------------------------------------------------
AnchoisA   3464 TATATTTTAAAATT-TGATAAAATTAAATTATGGGTTTGGGGTAATTCATTAAATATTATATAATTATATATAATTTATA

CTG40749b       TTGTTATAAA-TTTATTTAATCCTGCAAAAGCAGAAAGTCAAATTGTATTTGATTATA
IES77           TTATTATAAA-TGTATATATTCCTGCAAAGTCAGAAAGTC------------------
CTG23427        ----------------------------------------------------------
CTG35975        ----------------------------------------------------------
CTG21499        TTATTTTAAATTTATATAATCCTGCATAAGCAGAAAGTCAAATTGTATTTGACTATA
CTG40749        ----------------------------------------------------------
IES128          TTATTATAAA-TTTATATAATCCTGCAAAAGCAGAAAGTCAAATTGTATTTTACTATA
CTG28766        TTATTATATATTT---------------------------------------------
IES98           ----------------------------------------------------------
CTG10063        ----------------------------------------------------------
AnchoisA   3543 TTATTATAAA-TTTATATAATCCTGCAAAAGCAGAAAGTCAAATTGTATTTGACTATA
```

D

```
CTG36069      TATAGTC--TAAAAAAATTCAGCCAAAGTTTACTTTGAGTTGTAGCTAA--TCAATTTTATTTTGCTA-T-----AA-AC
IES85         TATAGTCA-AGAACAATTTCAGCCAACATTTACTTTGAGTT---ACAAAATTCAATTTTATTTT-CTA-TGGCAAAA-GC
IES57         TATAGTCGAAAAACAATTTCAGCCAACATTTACTTTTAGAT---TCAAAATTCAA-TTTATTTTGCTA-TTGCAAAAAGC
IES214        TATAGTCG-AAAACAATTTCAGCCAACAGTTACTTTGAGTT---GCAAAATTCAATTTTATTTTGCTT-TCACTAAA-GC
IES105        TATAGTCG-AAAACAATTTCAGCCAATATTTGCTTTGAGTT---GCAACATTCAATTTTATCTTGCTT-TTGTTAAA-GC
IES47         TATAGTC--AAAATAATTTCAGCCAACATTTACTTTGAGTT---GCAAATTTTAATTTTATTT--CTAATTGCAAAA-GC
IES80         TATAGTCG-AAAACTTTTTCAACCAACATTCACTTGGAGTT---CCAAAATTCAATTTTATTTTGATA-TAACAAAA-AC
CTG4797       --------------------------------------------------------------------------------
IES77b        TATAGTAG-AAAAAAATTTCAGCCAACATTCACTTTGAGTT---GCAAAAGTCAATTTTATTTTGCTA-TTGCAAAA-AC
CTG826        --------------------------------------------------------------------------------
IES132        TATAGCCG-AAAA-AATTTCAGCCAACATTAACTTGGAGTT---GCAAAATTCAATTTTATTTTGCTA-TCTCAAAA-GC
CTG7743       --------------------------------------------------------------------------------
CTG43276      AACATTCA-AAAAAAATTTCAGTCAACATTCACTTTGAGTT---GAAAATTGGAATTTTATTTTACTA-TTGCAAAA-GC
IES42b        TATAGTTG-AAAAAAATTTCAGCCAAAATTAACATTGAGAT---GAAAAATTGAATTTTATTTTGTTA-TCGCAAAA-AC
IES35         TATAGTCG-AAAAAAACTTGAGCCAACATTTAATTTGGGTT---CCAAAATTAAATTTTAATTTGTTA-TTGCAAAA-GG
IES104        TATAGTCG-AAATCAATTTCAGCCAACACTCACTTTGAGTT---GCAAAATAC---------------------------
IES76         --------------------------------------------------------------------------------
CTG13940      --------------------------------------------------------------------------------
IES42         TATAGTAG-AAAACAATTTCAGGCAACATTCACTTTGAATT---GCAAAGTTCAATTTTATTTTTTTA-TTACTAAA-TC
CTG34100      --------------------------------------------------------------------------------
IES169        TATAGTCG-AAAACAATTTCCGTCAACATTCGGTTTTAATT---GCAAAATTCAATTTTA-TCTGCTA-TCACAAAA-GT
IES103        TATAGTCG-AAAACAATTTCAGCCAACATTCACTTTGGGTT---GCAAAATTCAATTTTATTTTGCTA-TTGCAAAA-GC
CTG2630       --------------------------------------------------------------------------------
CTG45590      TATAGTCG-AAAAAAATTTCAGCCAACATTCACTTTGAATT---GCAAAATTCAATTTTATTTTTCTA-TCGCAAAA-GC
AnchoisB    1 TATAGTCG-AAAACAATTTCAGCCAACATTCACTTTGAGTT---GCAAAATTCAATTTTATTTTGCTA-TTGCAAAA-GC

CTG36069      AAAGT-ATATATTGAACCAA-GTTG--TTTA-------TGTTTAAAAAAAT---------TATAATAATTCTTTTTATAA
IES85         CAAGT-AAAAGTATAACCAA--TTGA-TTTATAA-GCA--TTTGAAAAAAA----TTGTT--TAATGATTTTTCTTATAA
IES57         TAAGT-AAA-------------------------------TTTGAAAGAATATTATTTTTAAT--TTATTTTTCT-ATAA
IES214        TATGT---AAATTAAACCAAAGTTTA-TTTA----------TTAAAGGGT-----TTTTTG-TAGAATTTTTTATTTTAA
IES105        TAATA-AGAATTTGAACCAA-GTTTA-TTTAAAA-GCAAGTTTGAAAAATC----TTTTTAAAAATAATTTTCTTTTTAA
IES47         TAAGT-AAATATTGAACCAA-----------------------------------------------------------T
IES80         CAA---------TGAACCAAGGTTAA-TTTAAAA-GTAGATTTGAAAGAAATTTTTTTTTGATAATAATTTTTCTTTTAA
CTG4797       --------------------------------------------------------------------------------
IES77b        CAAGT-AAAAATTGCACCAA-GTTTA--CTAAAA-GTAGGTTTGAAGGAAA----TTTTTGATGCTAATTTTTCTT-TAA
CTG826        --------------------------------------------------------------------------------
IES132        GAAGT-AAAA-TTGAACCCC-GTTAA-TTTAGAA-GCAGGTTTGAAGGAA----------TATAGTCATTTTTCTTATAA
CTG7743       --------------------------------------------------------------------------------
CTG43276      AAAGA-AAAAATTGATCCAA-GTTTA-TTTAAAA-GCAGATTTGAAGGAAA----TTCTTAATCATAATTCTTCTCATAA
IES42b        CAAGT-AAAAATTGTACCAA-GTTT-------------------GAAGGAAA----TTTTTGATAGTAAT-TTTCTTATAA
IES35         CAAATAAAAAATTGAACCAA-ATTTA-TTTAAAA-GCAGGTTTGAAAGAAC---ATCATTTATAATTATTTTTC-----A
IES104        --------------------------------------------------------------------------------
IES76         --------------------------------------------------------------------------------
CTG13940      --------------------------------------------------------------------------------
IES42         CAAGT-AAAAATTGAACCAA-GTTAA-TTTAAAA-GCAGGTTTGAAGGAAA----TTTTTGATAATAATTTTTCTTATGA
CTG34100      --------------------------------------------------------------------------------
IES169        TAAGT-AAAAATTGAACCAA-GTTTA-TCTAAAA-GCAGGTTT-AAGGAAA----TTTTTGATAATAATTTTTCTTATAA
IES103        CCAGT-AAAAATCCAACCAA-GTTTAATTTAAAAAGCAGGTTTGAAGAAAA-----TTTTGATAATAATCTTTATTATAA
CTG2630       --------------------------------------------------------------------------------
CTG45590      CAAAT-ATAAAT--------------------------------------------------------------------
AnchoisB   75 CAAGT-AAAAATTGAACCAA-GTTTA-TTTAAAA-GCAGGTTTGAAGGAAA----TTTTTGATAATAATTTTTCTTATAA

CTG36069      ATAAATTAACTTCTAAAATTTTTTGTTCGCTATTATAT--TTTGCCAATA-T--AATTAATTTCGCACTATATAATA---
IES85         ATATTTTAAATTCTAATA-TTTTTATACACTTTT-TATCTATTGCTAATGGT--AGTATAT-TTGTATCATATGATA---
IES57         ATAATTTAACTTCAAGTCTTTTT-ATGGAATATT-TATAAA--GCTTGTTAT--AATATATTATGAATTATATAAAACAT
IES214        T--ATTTCAAAACCAGCATATTTTTAGCTCTTTT-TATATATTGCTTCGTTT--AGTAT---------------------
IES105        ATAATTTAACTGCTAGTATTTTTTGTGCACTTCTTTACATTTTCCTTGTAAT----------------------------
IES47         TGATATTAAATTATAATATTT-----------------------------------------------------------
IES80         TTATTTTATCTTCTCGTATTTTTTGTGCACTTTAATATATATTACTAATAAT--AATATAATTTACATTATATGATATAT
CTG4797       --------------------------------------------------------------------------------
IES77b        TTAATTTAACTCCTTGTATTTTTTGTGCTCCTTAATATATATTGCT-ATTAT--AATATATGTTGCATGATATTATATAT
CTG826        --------------------------------------------------------------------------------
IES132        ATAATTTAACTTTTAGTATTTTTTGTGCACTTTTTTAT-TATTGCTTAGAACAAAATATATTTTGCATTATCTGATATAT
CTG7743       --------------------------------------------------------------------------------
CTG43276      TTAATTTA----CTAATATACTTTGTGCACTTTTATCTATAATACTTATAGT--AATTTAGTTTTCATTATATTATACAT
IES42b        TAAATTTGACTTCTTGTATTTTTTGTGCACTTTT---------------------ATATATTTTGCATATTATGATATAG
IES35         ATAATTTA-------------------------------------TTAC---------TATAATGC-------GAT----
IES104        --------------------------------------------------------------------------------
IES76         --------------------------------------------------------------------------------
CTG13940      --------------------------------------------------------------------------------
IES42         TTAATTTAATTTCTAATATTTTTTGTGCACTTAAATCCATATTGTTTATAAT--AATATATTTTCTATTAT-TGATATAT
CTG34100      --------------------------------------------------------------------------------
IES169        TTAATTTAACTTTTAATATTTTTTGTGCACTTTTATCTATATTGCTTATAAT--AATATATTTTGCATTATA--------
IES103        TTAATTTAACTTCTAATA--TTTCGCGCACTTTTACCTGTGTTGCATATAAT--AATATATTTTGCATGATAAGAGATAT
CTG2630       ------TAACTTCTAATA-TTTTTGTGCACTTTTAA------------------ATAATATTTTGCATTAATTGAAATAT
CTG45590      --------ACTTCTATCA-TTTTTGTACACTTTCATTTATATTGCTTATAAT--ACTATATTTTGCATTATATGATATAT
AnchoisB  147 TTAATTTAACTTCTAATATTTTTTGTGCACTTTTATATATATTGCTTATAAT--AATATATTTTGCATTATATGATATAT
```

```
CTG36069        AAAACA--GTAATGCAATTATACAGCTAC-AAATATCATTCAGGATATTT-ATTAATTTAAATCATGTAAGTCTA--TTT
IES85           -AAGCAAACTAAAGCAATTTTATTGCAGC-AAATTTTTTTCAGGATCTTAAATT-------ATTATGTATGTCTA-TTTT
IES57           T-----AAGTAACGCAATTTAATCTTAGC-AAATTTTTTTCAACATAATTC-TTAATTTATAGTATGTATGTCAATTTTT
IES214          -----------------------------------TTTTTGTGGACAATCTTC---------------------------
IES105          -------------------------------------------ATTATTTTTATAATTTAAATTTTGTATGTCTATTTTT
IES47           -------------------------------------------GATATTTTTGT--------------------------
IES80           AAAGCAAA-CATCTCAATTT-ATTGCAGC-AAA-TTTTTTGAGGATATTT-ATTATTTTAAATTATGTCTGCCTATTTTT
CTG4797         --------------------------------------------------------------------------------
IES77b          AAAGCA-TGTAACTCAATTTTATCGCAGC-AA---TTTTTCAGGATATTTTAATAATTTAAATTAT----ATTTATTTTT
CTG826          -----------------------------------------------------------AAATTATGTATGTCTA-GTTT
IES132          -AAACAAAGTAAAGAAATTTTATCACAAT-AA-CTTTTTTCAGGAGATTTTCTTAATTTAAATTATTTATATCTA-TTTT
CTG7743         --------------------------------------------------------------------------------
CTG43276        TAAGCA----------------------------------------ATTTTAT---------------------------
IES42b          GAAACAAAGTAACGGAATTTTATCACAGC-AAA--TTTCTCAACATATTTTA-TAAATTAAATTATGTATGTCTATTTTT
IES35           ------------------------------------------GGATATTTTATTATTTTA-ATTATTTAGATCTA-----
IES104          --------------------------------------------------------------------------------
IES76           --------------------------------------------------------------------------------
CTG13940        --------------------------------------------------------------------------------
IES42           AATGCAAA-----------------------------------AGTATTTTGGTAATTGAAA------------------
CTG34100        --------------------------------------------------------------------------------
IES169          ---GCAAAGTAAAGCAATTTTAGCGCAGC-AAATCTTTTTCAGGATATTTTATTAATTAAAATTACGTATGTGT------
IES103          AAGGCAAAGTAACGCAATTTTATCGCAGT-AAATTTTTTACAGGATATTTTGTTAATTTAAATTATGTATGTCT------
CTG2630         AAAGTAATGCAA------TTTATAGCAGCAAAATTTTTTTCAGGATATTTAATTAATTTAAATTATGAATGTATATTTTT
CTG45590        CAATCAAAGTAACGCAATTTTATCGCAGC-ATATTTTTTTCAGGATATTTTATTAATTTAAATTATGTATGTCTATTTTT
AnchoisB    225 AAAGCAAAGTAACGCAATTTTATCGCAGC-AAATTTTTTTCAGGATATTTTATTAATTTAAATTATGTATGTCTATTTTT

CTG36069        CGATTTCA-TTATTATTTTC-AAAGTGTTTT-AATATAACCTAAATAGCCTATAGTT--TACCAATTTTTCC-AAAAAGC
IES85           TGTATTCC-TTA----TTTC-AAAATGCCTGAAATATAACCCAACTAGCCCATTACT--TGCCAATAATACC-AAAATGT
IES57           TGT-GTCA-TTA---TTTTT-AAAATGTCTGAAAAATAACTCAACTAGTCCATAATTTCTGCCAATTTTGGC-AGAAATT
IES214          --------------------------------------------------------------------------------
IES105          TATTTACA-TT-------------------------------AACTAACCCACAATT--T-ACAATATTGCC-AAAAAGA
IES47           --------------------------------------------------------------------------------
IES80           T-TTTTCA-TTGTTATTTC--AATATGTCTGAAATATTCCCCAACCAGCCCTTAACT--TGGCAATATTGCCAAAAAAAT
CTG4797         --------------------------------------------------------------------------------
IES77b          TTTTTTCT-TTGTTATTTTC-AAAG----------------CAACCAACCCATAACT--TG-CAATATTGGC-AGAAAAT
CTG826          TGTTTTCA-TTTTTATTTTC--AAATGTCTCAAATATAATCCAATTAGCCCATAACT--TTCCAATA-------------
IES132          CGTTTTCA-TTATTATTTTC-AAACTGTCCTAAATATAAACTAATTAGCCCTTAACT--TGGTGTTATTGCC-AGAAAGT
CTG7743         --------------------------------------------------------------------------------
CTG43276        --------------------------------------------------------------------------------
IES42b          TGTTTTCAATTGTTATTTTC-AAAATGTCTGAAATATTCCCATACTAGCCCATAACT--TGCCAATATTGCC-AGAAAAT
IES35           -----------------------------------------------------------TGCCAATATTGGC-AGAAAGT
IES104          --------------------------------------------------------------------------------
IES76           --------------------------------------------------------------------------------
CTG13940        --------------------------------------------------------------------------------
IES42           --------------------------------------------------------------------------------
CTG34100        --------------------------------------------------------------------------------
IES169          ----TTCA-TTGTTATTTTC-AAAATGTCTGAAATATTCCCCAACTAGCCCATAACT--TTTCAAAATTGCC-AGAAAAT
IES103          --------------------------------------------------------------------------------
CTG2630         TGGTTTCA-TTATTATTTTCAAAAATGCCCGAAATACTCCCCAACCAGCCCATAAAT--TGCCAATATTGTC-GGAAAGT
CTG45590        TGGTTTCA-TTGTTATTTTC-AAAATGTCTGAAATATC-CCCAACTAGCCCATAACT--TGGCAATATTGCC-AAAAAGT
AnchoisB    304 TGTTTTCA-TTGTTATTTTC-AAAATGTCTGAAATATAACCCAACTAGCCCATAACT--TGCCAATATTGCC-AGAAAGT

CTG36069        ACTGTTTAGATAATTGAACATTAAGG-CTCATCGTTTTATTT-T-ATTAAAAAG-TTAAAG----AAACATTTGGAA---
IES85           ATCGTTTTGATGATCAA--ATCAAGG-CCTATCGT-TTATTCGT-ATTGAAAAA-TTGAAG-----AAAATTTGGAA---
IES57           AATGTTTTGGTAATTGGACATCAAGG-CCCATTGTTTTTTTTT-ATTAAA-AA-TTGAAA----ATAAATTTCGAA---
IES214          ---------------------------------------------------------------------ATATGGAA---
IES105          ATTGTTTGGGTATTTGAACATCCAGG-CCCATCGTTTAATTTGT-ATTGAATAA-TTGAAG----AAGAATTTGGAA---
IES47           --------------------------------------------------------------------------------
IES80           ATTGTTTGGGTAATTGAACATCAAGG-CCCATCGTTTTATTTGT-A----------------------------------
CTG4797         --------------------------------------------------------------------------------
IES77b          ATTTTTC--ATAATTGAACATCGAGA-CCCATCTTTTTATATGT-ATTGAATAACTTGAAGTTTTTAAAATTTTGAAATG
CTG826          ------------------CATCA------------TTTATAT---ATTGAAAAA-TTGGGG----AAAAATTTGGAA---
IES132          ATTGGTTTGGTAATTGAACATCATCG---------TTTTTTTAC-ATCCAACAA-TTGAAG----AAAAATTTGGTA---
CTG7743         --------------------------------------------------------------------------------
CTG43276        --------------------------------------------------------------------------------
IES42b          ATTGTTTGGGTAATTGAACATCAAGG-CCCATCGTTTTATTCGT-ATTGAACAT-TTGAAG-----AAAATTTGGAA---
IES35           ATTGTTTGGGTAATTAAACATCAAGTCCCCATTGTTTTATTTGT-ATTAAACTA-TCGAAG----AAAAA-TTGGAA---
IES104          --------------------------------------------------------------------------------
IES76           --------------------------------------------------------------------------------
CTG13940        --------------------------------------------------------------------------------
IES42           -------------------ATCAAGG-CCGATT-TTTTATTTGT-GTTGAATGA-TTGAGG--AAAAAAAATTGGAA---
CTG34100        --------------------------------------------------------------------------------
IES169          ATTGTTTGGGTAATTGAACATCAAGG-ACGATCGT----------GTTGAACTA-TTGAGA----AAATATTTAGAA---
IES103          --------------------------------------------------------------------------------
CTG2630         ATTGTTTGGGTAATTGAACATCAAGG-CCCATCGTTTTATTTAT-GTTGAACAA-TTGAGA-----AAAATTTGGAA---
CTG45590        ATTATTTGGATAATTGAATATTAAGG-CCAATCTTTGTATTTGTAATTTAACAA-TTGAAG----TAAAATTTTGGA---
AnchoisB    379 ATTGTTTGGGTAATTGAACATCAAGG-CCCATCGTTTTATTTGT-ATTGAACAA-TTGAAG----AAAAATTTGGAA---
```

```
CTG36069        -----TTATAATTATAGTTTGGCTAAGAA---AAACCAACATAGAAGAACA-AAAAATTATT-AGAAAATGT--TATAGC
IES85           -----TTAGAAATATGGGTTGGCAAACGAA--AAATCAACACTGAAGAATA-AAATAGTTAATCTTAAGAGTAT----GC
IES57           -----TTAGAATTATAGATTGGCTAAGGAAACAA-------CAGGAGAACA-AAACAGTTTA------------------
IES214          --------------------------------AAATCAACGCAAGAGACCA-AAAC-GTTTACAGATAA-GCATTTTAGC
IES105          -----TTAGAATTATAGTTTGGGTAAGGAAATAAATCAACGAAGGACAACA-AAAAAGTTTATCAAAAAAGTATTGGAAC
IES47           ----------------------------------------------------------TTTATAAAGAA-----------
IES80           --------------------------------------------------------------------------------
CTG4797         --------------------------------------------------------------------------------
IES77b          GTTTTTGAATATTTTGAGTTGGATA---CAT-AAA-----------GAACATAAAAAGTTTATAGAAAAAGTATTGC-GC
CTG826          -----TTAAAATTATAGGTCGGCTAAGGAAA-AAATCAACGCAAGAGAACA-ATAAAGTTTATAGAAAAAGTATTGTATC
IES132          -----TTAGAATT---GGTTAACTACGGAAA-AAATCAAAGCTGGAGAACA-AAAAA-TTTATAGAAAA-ATATTTTTGC
CTG7743         --------------------------------------------------------------------------------
CTG43276        --------------------------------------------------------------------------------
IES42b          -----TTAGAATGATAGGTTGGCTAAGGAAA-AA-TCAACCCAGGAGAACA-AAAAAGTATATAGAAAA-GGACTGTAGC
IES35           -----TTA-AATTATAGTTTGGCTAAGGAAA-AAATCAACTTAGAAGAACA-ACAAAGTTTATAGAAAAAGTATTGTAGC
IES104          --------------------------------------------------------------------------------
IES76           --------------------------------------------------------------------------------
CTG13940        --------------------------------------------------------------------------------
IES42           -----TTAGAATTATAGGTTGGCTAAGGAAA-AAATCAACAAAGCAGAACA-AAAAAATTTTTAGAAAA-------TAGC
CTG34100        --------------------------------------------------------------------------------
IES169          -----TTAGAATTATAGGTTGGCTAAGGAAA-AAATCAACGCAGGA-AA-A-AACAAAGTTATAGAAA--GTATTGTAGC
IES103          --------------------------------------------------------------------------------
CTG2630         -----TTAGAATTATAGGTTGACTAAGGAAA-AAATCAACGCATGAGAACA-AAGAAGTTTAAAGAAAATGCATATTAGA
CTG45590        -----TTAGAATTATAGGTTGGCTAAGGAAA-AA--TCACGCA-GAGAACA-AAAAAGTTTATAGAAAAAGTATCATAAA
AnchoisB    449 -----TTAGAATTATAGGTTGGCTAAGGAAA-AAATCAACGCAGGAGAACA-AAAAAGTTTATAGAAAAAGTATTGTAGC

CTG36069        CAGGTTT-AGCAAGCA--AATTAAAAATAA-CATAAAGATA-TTAAGATAATAAACAAAGGATCTAATAATTTGATGGAA
IES85           A----------AAACTTCTTTTAAAATGATACCAAATCAAA-TTCAGATCATAAGCAAAGAACCTAATGCTTAGATGAAA
IES57           --------------------------------------------------------------------ACTTAAATG--A
IES214          AAGGCTT-AGCAAATTTCTAATAAAATGAATCTAGATTAAA-TTCAGATCATAAATAAAGGACCTAACATTCAGATG-AA
IES105          AAGGTTA-A--CAACATCTAATAAAATGAATCTAAAAGAAA-TTCAGTTCATAAGGAAGGAACCTAACACTTAGATGGAA
IES47           --------------------------------------------------------------------------------
IES80           --------------------------------------------------------------------------------
CTG4797         --------------------------------------------------------------------------------
IES77b          AAGGTTTTAGCAAACTTCTAATAAAATAAATAGAAAA-AAA-TTAAGATTATAAGCAAAGGAACTAATACTTAGATAAAA
CTG826          AAGTTTA-ACCAACCTT----------GGATCTAAAAGAAA-TTCAGATCATAAGCAAAGGACCTATTATTAATATGAAA
IES132          AAGGTTT-AGCAAACTTCTAATAAAATGAATCTCAAAGAAATTTCTGATCACAAGCAAAGGACCTAACAGGAAGATG-AA
CTG7743         --------------------------------------------------------------------------------
CTG43276        --------------------------------------------------------------------------------
IES42b          AAGGTTT-AACAAAATTCCATT-TAATGAATCTAAAAGAAT-TTCAGATCATAAGCAAAGGACCGAACACTTAAATG--A
IES35           AAGGTTT-AGCAAACTTGCAATAAAATGAATCTAAAAGAAA-TTCAGATCATAAGTACTGGACCAAACACTTAAAT----
IES104          --------------------------------------------------------------------------------
IES76           -----------AAACTTCCAA-AAAATGAATTTAAAAGAAA-TTCAGATCATATGCAAAGGACCTAACACTTA-------
CTG13940        --------------------------------------------------------------------------------
IES42           AAGGCTT-GGATGACTTCCAATAAAATGAATCTAAAAGAAA-TTCAGATCATAAGCAAAGGACCTAACACTTATATG-AA
CTG34100        --------------------------------------------------------------------------------
IES169          AAGGTTT-AGCCATTTTTCAATAAAATGACTCTAAAAGAAA-TTCAGATTATAAGCAAAGGACCTAATACTTAGATG--A
IES103          --------------------------------------------------------------------------------
CTG2630         AAGGTTT-AGCCAACTTTCAATAAAACGAAT-TAAAAGAAA-TTCAGATCATAAGCGAAGGACCCAAAACTTAGATGAAA
CTG45590        AAGGTGT-AGC-AACTTCCAAT-AAATGAACCTAAAAGAAA-TTCAGATCATAAGCAAAAG-TCTAACACATAGATG-AA
AnchoisB    522 AAGGTTT-AGCAAACTTCCAATAAAATGAATCTAAAAGAAA-TTCAGATCATAAGCAAAGGACCTAACACTTAGATGAAA

CTG36069        AAAGAGCTGAAGATATTTTTTAGCAATAGCAATTTAAAA-TTTCAGAAAA--TAAGCAAAAGCTGGAAATAAAATAACCA
IES85           AAAATGCTAAAGACAATATTTATCAATAGCAATTAAAAA-TTTGAGAAAA--TTAGCAACAGCAG---------------
IES57           AAAATGTTATAGATAATATATGGTCATAGCAATTAAAAT-TT-CATAAAA--CTGACAACAGCAGTAAAGAAAATCAATA
IES214          AGAATATTAAAGCCAATACTTAACAATAGCATATAAAAA-TTTCAGAAAAA-CTAGCCATTGCAGGAAATAAAATAAACA
IES105          AAAATGCTAGAGTTAATATTCAAAAATAGCAA---------------------------CAGCAGGAAATAAAATCAATA
IES47           --------------------------------------------------------------------------------
IES80           --------------------------------------------------------------------------------
CTG4797         --------------------------------------------------------------------------------
IES77b          AAAATGCTAAATAAAACATTCTGCTAAAGCAAT--AAAA-TTTTAGAAGA-----------GTAGGAAATAAAATAAACA
CTG826          AAAATGCTAAAGACATTATTTAGCAAAAGAAATTAAAGA-TTTCA-AAAA--CTATCAGCAGCAGGAAATAAA-----CA
IES132          AAAAAGTAAAAGACAACATTTAT---------------------------------------------------------
CTG7743         --------------------------------------------------------------------------------
CTG43276        --------------------------------------------------------------------------------
IES42b          AACATGTTTTAGACAATATTTAGCAATAGCAAAAAAAAA-TTTCAGAAAAGCCTAGCAACAGCAGGAAATAAAATATGCA
IES35           ATAATTCTTAAGACAATATTTTGCAATAGCAATTAAAAAATTTCATAAA---CTAGCATCAGCAGAAAATAAAATAAATA
IES104          --------------------------------------------------------------------------------
IES76           ----------------------GCAATAGCAATTAAAAA-TTTTAGAAAA--CGAACAATAGCAGGTAATAAA--TAACA
CTG13940        --------------------------------------------------------------------------------
IES42           AAAATGCTAAAGACAATATTTAGCAATAGCAATTAAAAA-TCTCAGAAAA--CTAGCAATAGCAGGAAACAAAATAAACA
CTG34100        --------------------------------------------------------------------------------
IES169          AAAATGCTAAAGA----------------------------TTCAT----------------------------------
IES103          --------------------------------------------------------------------------------
CTG2630         AATATGCTAAAGACAATTTTTAGCAATAGCAA------------------------------------------------
CTG45590        AAAATGCTAATGACAATATTTAGCAATAGCAATTAAAAA-TTTCAG-AAA--CTATCAACAGCAGGAAATAAAATAAACA
AnchoisB    600 AAAATGCTAAAGACAATATTTAGCAATAGCAATTAAAAA-TTTCAGAAAA--CTAGCAACAGCAGGAAATAAAATAAACA
```

```
CTG36069        GAATAA-TAAGAT-AAGAAATAGTATCAGAAAAAAATCAAGAT-TTAGAGTAGAATAATAGTATTTTGAAGAAAGTTAAG
IES85           --------------------------------------------------------------------------------
IES57           C-GAGAATAAGCTGAAGAAATAGTATCAGAAAATAATTAAGGT-ATAGAGCAG---AA-ATTATGTTGAAGAAGGTTAAG
IES214          G-AAAAAAAAGCTG------TGGTATC----AAAAATCAAAAT-ATCGAACAT---CATATTA-TTTGAAGAAAGTTAAG
IES105          G--AAAATAAACAGAAGAAATAGTATTTG--AAAAATCAAGAT-GTAGAGCAG---AATATTAATTTGAA-AAAGTTAAA
IES47           --------------------------------------------------------------------------------
IES80           --------------------------------------------------------------------------------
CTG4797         --------------------------------------------------------------------------------
IES77b          G-AAAGATAGGCTGAAGAAATAGTAGCAGAAAA-----------------------------------------------
CTG826          G-AAAAATAAGCTGTACAAGTAGTATC----AAAGATCAAGAC-GTAGAGCAG---AACATTATTTTGAAGAAATTTAAG
IES132          -------------------------------------------------GCAG---AATATTA-TTTGAAGAAAGTTAAA
CTG7743         --------------------------------------------------------------------------------
CTG43276        --------------------------------------------------------------------------------
IES42b          G-AAAAA---------------GTTGTAGAAAAAAATTAACAT-ATAGAACAG---AACATTATTTTGAAGAAGATTAAG
IES35           GAAAAAATAGACTGAGGAAATATTAGCAGAAAATAATCAAGAT-GTAGAGCAG---AATATTATTTTGAAGAAGATTAAG
IES104          -------------------------------------------------------------------------------G
IES76           G-AAAAATAGGCTGAAGAAATATTAGCAGAAAAAAATCAAGAA-GAAAAGCAT---AATATTATTTTGAAGAAAGTTAAG
CTG13940        --------------------------------------------------------------------------------
IES42           G-AAAATCGACTGAAGAAATATTAACAGAAAAAA-TCAAGATTGTGGAGTAG---GATATTATTTTGAAGAAGGTTAA-
CTG34100        --------------------------------------------------------------------------------
IES169          --------------------------------------------------------------------------------
IES103          --------------------------------------------------------------------------------
CTG2630         --------------------------------------------------------------------------------
CTG45590        G-AAAAATAGGCTGAAGAAATAGTAGCAG--AAAAATCAAGGT-GTAGAGTAG---CGTATTATTTTTAAGAAGGTTAAG
AnchoisB    677 G-AAAAATAGGCTGAAGAAATAGTAGCAGAAAAAAATCAAGAT-GTAGAGCAG---AATATTATTTTGAAGAAAGTTAAG

CTG36069        AAAATAGTT-CAGAAATGGAAATG-T-AAAAATGAATTAAAGAATTAGTAA-GGGAACA-TGATGAATATCGACA--TTT
IES85           --------------------------------------------------------------------------------
IES57           AGAATAGTT-GAGAAATAGAGATGCT-AAAAATGCATTAAAGAATTCATAAAGAGG-CAAGGA-GGATATCGACA-TTTT
IES214          AGAATAGTT-GAGAAATGTAAATGCTAAAAAATACATTAAAGAATTTATTA-GGGAACA-GGATGAATTTCGACA-----
IES105          AGAATAGTT-GAGAAATGGAAATGCT-AATAATGCATTAAAGAATTCATAG-TGGAACA-GGATAGATATTGGTA-TTTT
IES47           --------------------------------------------------------------------------------
IES80           --------------------------------------------------------------------------------
CTG4797         --------------------------------------------------------------------------------
IES77b          --------------------------------------------------------------------------------
CTG826          AGAATAGTT-GAGAAATGGAAATGCT-AGAACTGCATTAAAG-ATTCACAA-GGGAACA-GGATGGA---------TGTC
IES132          AGAAAAGTT-GAAAAATGG-AATATT-AGAAATGCATTAAAAAATTCATTA-GGGAGTA-G-------------------
CTG7743         --------------------------------------------------------------------------------
CTG43276        --------------------------------------------------------------------------------
IES42b          AGAATAGAT-GAGAAATGGAAATGCT-AGAAATGCATTAAAGAATTCATAG-GGAAACA-GGGTGTATATCTACATTTTT
IES35           AGAATAGTT-GAGAAAAAGAAATGCT-AGAAATGCATTAAAGTATTAATAA-GGGAACA-TGATGGATAAGGACA-TCTT
IES104          AGAATAGTT-GAGAAATGGAAATGCT-AGAAATGTA-TAAAGAATTCGTAA-GGGAACA-TGATGGATATCGACA-TTTT
IES76           AGAATAGTT-GAGAAATAAAAATGCT-AGAAATGCATTAAAGAATTCATAA-GG-AACA-GGATGAATATCGACA-TTTT
CTG13940        --------------------------------------------------------------------------------
IES42           -AAATAGTTTGAGAAATGGAAATGCT-AGAATTGCATTAAAGAATTAACAA-GGGAATA-GGAAGGATATCTACA-TTTT
CTG34100        --------------------------------------------------------------------------------
IES169          --------------------------------------------------A-AGGAATA-AGATGGATATCGACA-TTTT
IES103          --------------------------------------------------------------------------------
CTG2630         --------------------------------------------------------------------------------
CTG45590        AGAATAGTA-GAGATATGGAAATGAT-AGAAATGCATTAAAGAATTCATAA-GGGAACA-GGATGGATCTCGACA-TTTT
AnchoisB    752 AGAATAGTT-GAGAAATGGAAATGCT-AGAAATGCATTAAAGAATTCATAA-GGGAACA-GGATGGATATCGACA-TTTT

CTG36069        GAGCACAATTCTAAAGATTTGTATTGAAGAGGAGAAAAAAT---ACTTTAAGCAGAAAAG-----AAAGAA---GGTTGA
IES85           --------------------------------------------------------------------------------
IES57           GAGTACCATAATAAAGA-TTGTAACGAAGAGGACAAAA-TT---TCAGTGAA-AGAGAAG-----AAAGAA--TAGTAGC
IES214          -----TCATTATAAA-A-TTGTATGGAAGAGGAGAAAA--C---TCATTGAGCAAAGAAG--------AAAAAAAGTTGC
IES105          GAGCACAAACATTAAGA-TCATAACGAAGAGGAGAAAAAAATAACTCTTTAAGAAGAGAAGATAATAAAGAAATAAGTTAC
IES47           --------------------------------------------------------------------------------
IES80           --------------------------------------------------------------------------------
CTG4797         --------------------------------------------------------------------------------
IES77b          --------------------------------------------------------------------------------
CTG826          GAGCATCATCAAAAAGA-TTGTAACGATGATTAG-AAAAAT---TCTTTTAGCAGAGAAG-----AAAGAAAAAAGTTGC
IES132          --------------------------------------------------------------------------------
CTG7743         --------------------------------------------------------------------------------
CTG43276        --------------------------------AGCAGAAAT---TTTCAGAATATA------------------------
IES42b          GAGCACCATCATAAAGA-TTGTAACAAAGAGGAGAAAAAAT---TCTTTAAACAGAGAAG-----AAAGAAAAAAGTTGC
IES35           GAGCACCATTAGAAAGA-TTGTAAAGAAGAGGAGAAAAA-T---TCTTTGAGTAGAGAAG-----AAAGAAAAAGGTTGC
IES104          GAGCACCATCATAAAGA-TTGTAATGAGGAGGAGAAAAAGT---TCTTTGAGCAGAGAAG-----AAAGAAAACA-TTGC
IES76           GAGCA-----------------------GAAGAGAAAAAAT---TCTTTGAGCA-AGAAG--------------------
CTG13940        --------------------------------------------------------------------------------
IES42           GAGCACCATCATAACGA-TTGTAACGAGGAGGAG-AAAAAT---TCTTTGAGGAGAGAAG-----AATGAAAAATGTTGC
CTG34100        --------------------------------------------------------------------------------
IES169          GAGCACTATAATAAAGA-TTGTAACGAAAAGGAGAGAAAAT---TCTTTGAATAGAGAAG-----GAAGAAAAAAGTCGC
IES103          --------------------------------------------------------------------------------
CTG2630         --------------------------------------------------------------------------------
CTG45590        GAGCACCATCATTAAGA-TTGTAAAGAAGAGGAG---AAAT---TTTTTGAGCAGAGAAG-----AATGAAAAAAATTGC
AnchoisB    827 GAGCACCATCATAAAGA-TTGTAACGAAGAGGAGAAAAAAT---TCTTTGAGCAGAGAAG-----AAAGAAAAAAGTTGC
```

```
CTG36069         AGAATCATAGCAATAGCAAGTAGAAAAATGATACATATATATAGATTAAAT-TAAATAATATTT--GATCTTTTTGTTTT
IES85            ----------------------------------------AAATATAAC-------------------------------
IES57            A----CA--GCCATAGCAAATTGGAAAATGATGTATATATAGATATAAAAT-TATGCATTATTT--TATCTTTTTGTTTC
IES214           AGAATC---GTAATAGT----AGCATAATGAT-TAT-TATAAATATAAAAA-TAAATAATATTT--GATCTTCTT-TTT-
IES105           AGAATCGTCACA-TAGCAAGAAGAAAAATAATATCTGTATATATATAACAT-TATATAATATTT--GATCATTTTTATTT
IES47            --------------------------------------------------------------------------------
IES80            --------------------------------------------------------------------------------
CTG4797          --------------------------------------------------------------------------------
IES77b           ----------------------------------------AAAATTAAGAT-----------------------------
CTG826           AGAATCATAGCAATAGCAAG-GGAAAAATGATATATATA--AACATAAAAT-TATACTTTGATT--GATCTTTTTGTTTT
IES132           --------------------------------------------------------------------------------
CTG7743          --------------------------------------------------------------------------------
CTG43276         ------------------------------------TTATAATTATAAAAC-TAAAGATTATTT--GATCTTTTT-TTTA
IES42b           TGAATCATAGAAACAGCAAGAAAAAACATGATATATTTATAAATAAAAA-T-TAGAGATTATTT--GATCTTTTATTTTT
IES35            AGACTCACAGCAATAGCAAGAAGAATCATTATATATTTATAAATATAAAAT-TAGAGAATATTT--GATCGTTTTGTTT-
IES104           AGAATCATAGCAAAAGCAAGAAGAAACATGATATATATTTAAATATAAAAGTTAGAGAATATTT--GATCTATTT-TTTT
IES76            ------------------------AAAATGAAATATATATAAATATAAA-T-TAGAGAATGTTTTTGATCTTTTTGTTTT
CTG13940         --------------------------------------------------------------------------------
IES42            AAAATCATTGCAATAGCAAGTAGAAAAATGATATATATATAAATATAAAAT-TAGTGAATATTT--GATCATTTAAAAAA
CTG34100         --------------------------------------------------------------------------------
IES169           AGAATCCTAGCAGTAGCAAGATGAAAAATGATATATATATAAATATAAAGT-TAGAGAATATTT--GATTTTTTT-TT--
IES103           --------------------------------------------------------------------------------
CTG2630          --------------------------------------------------------------------------------
CTG45590         AGAATCATAGCAGTAGCAGGAAGAAAAATG------ATATAAATATAAACT-TAGAGAATATTT--GATCTTTTT-TTTT
AnchoisB     898 AGAATCATAGCAATAGCAAGAAGAAAAATGATATATATATAAATATAAAAT-TAGAGAATATTT--GATCTTTTTGTTTT

CTG36069         -TTATC-ATT-----AATAATTATTCAATTTTCATCTTT-AAAATGCCTGCGCGTTTATTAAACTTCTTTTTAAATTTCC
IES85            --------------------------------------------------------------------------------
IES57            -GTTTA-CG------AATCTTTATTCAATTTTCTTCTTT--AAATGAAAGCGC--TTATTAAACTTCTTCTTAAA-TTCC
IES214           -GTTTTCATTTT---AATAATTATACAATTG-CTTCTTT-AAAATGACTATGC--TCAT-AAAC-TCTTTTTAAA-TGAC
IES105           TGAACT-ATT-----AATAATTATTCAA-------CTTT-AAAATGATTGCTC--TTATTAAATTTCCACTCAAA-TTCC
IES47            ------------------------TCAATTT-CTTCTTT-AAA-----TGCAC--TTATTGAACTTCTTCTTTTA-TTCC
IES80            --------------------------------------------------------------------------------
CTG4797          --------------------------------------------------------------------------------
IES77b           ----------------------------------------------------C--TTATTAAACTTCTTCTTAAA-TTTC
CTG826           -GTTTT-GTTTTATTAATAATTCTTTAATTT-CTTCTTT-AAAATGACTGTGT--TTATTAAATATCTCCTTAAA-----
IES132           --------------------------------------------------------------------------------
CTG7743          --------------------------------------------------------------------------------
CTG43276         -A-----ATT-----AATAATTATTCAATTT-CTCCTTT--AAACGACTGCGC--TTATTAAACTTCTTCTT--------
IES42b           -AT----------------AATAATCAATTT-TTTCTTT-AAAACAAATGCGC--ATATTAAA---ATTCTTAAA-TTCA
IES35            -GTTT--ATT-----AATAATTATTCAATTT-CTTTTTT-AAAA-AACTGTGG--TTATTAAACATCTTCTTAAA-TTCC
IES104           -----T-ATT-----ACTAATTATTTGATTT-CTTCTTTAAAAACGAATGCCC--TTATTAAACTTCTTCTTAAA-TTCC
IES76            -GTTTT-ATTAATAAAAAAATTATTCAATTT-CTTCTTT-ACAACGACTGCCC--TTATTAAACTTCTTCTTAAA-TTCC
CTG13940         --------------------------------------------------------------------------------
IES42            ---------------AATAATTATTCAATTT-CTTCTTT-GAAATGACTGCGC--TTATTAAACTTCTTATTAAA-TTCC
CTG34100         --------------------------------------------------------------------------------
IES169           ---------------AATAATTATTCAATTT-ATTCTTT-AAAACGACTGCGC--TTATTAAACTTCTTTTTAAA-CTCC
IES103           --------------------------------------------------------------------TCTTAAA-TTCC
CTG2630          --------------------------------------------------------------------------------
CTG45590         -----T-ATT-----AATAATTATTTAATTT-CTTCTTT-GAAACGAACGCGC--TTATAAAACTTCTTCTTAAA-TTCC
AnchoisB     975 -GTTTT-ATT-----AATAATTATTCAATTT-CTTCTTT-AAAACGACTGCGC--TTATTAAACTTCTTCTTAAA-TTCC

CTG36069         TTAATTTATTCGTTCTAGCTCCTCGGTTAGCTTTGATAATTTAAGCAATTTTTT-TGCTTAAA--T--GACCTCTTTACA
IES85            --------------------------------------------------------------------------------
IES57            TTAATTTATTTGTTCAAACCCTTCAATTACTTTTGACAATATATGCAATTTTTTTTGCATTAA--T--GAGCTCTTGGCA
IES214           TTAGTTTATTAGTTATAACTAGTCAGCTAACTTTGATAATTTAAGTAGTTTTTT--GCTTAAT--T--------------
IES105           TTAATTTATTTGT-------------------------------------------------------------------
IES47            -TAATTTATTTGTTCTAGCTCTTTAGTTAACTTAGATAATTTCAGCCATTTTCT--GGTTAAA--T-----------GCG
IES80            --------------------------------------------------------------------------------
CTG4797          --------------------------------------------------------------------------------
IES77b           GTAA----TCTATTCTAACTCATTAGATAACTTTGATAATTTAAGCAATGTTTT-TGCTTAAA--T--AAGCTCTTTGCG
CTG826           -----TTATTTCTTCTAACTCTTCGGTTAACATTGATAATTTAAGCAATTTTTT--------------------------
IES132           ----------TATTTTATTTCTTCAGTTAACTTTGATAATTTAAGTAATTTTTT-TGCTTAAA--T--GAACTCTTCACA
CTG7743          --------------------------------------------------------------------------------
CTG43276         ---------------------------------TGATAATATAAGCAATTTTTA--GCTTAAA--T--AAGATCATTGCA
IES42b           TTAATGTATTTATTCTAATTCTTCAGTTAACTTTGATAATTTCGGCAATATTTT--GCATAAAAATAAAAGTTCTTTGCT
IES35            TTAATTTATTAATTCTAACTCTTCAGTTAACTTTAATAATTTAAACAATTTTTC--GCTTAAA--T--AAGCTCTTTGC-
IES104           TTAATTTATTTATTCTAACTCTTGAGCTAACTTTGATAATTTAA--AAATTATTTTGTTTAAT--T--AAGTTCCTTACA
IES76            TTAATTTATTTATTCTAATTCTTGAGCTAACTTTGATAATTTAAGCAACTATTT-TGTTTAAA--T--AAGCTCTTTTCG
CTG13940         --------------------------------------------------------------------------------
IES42            TTAATTTATTTATTATAACCCTTCAGTTAACTATGATAATTAGAGCAAATTTTT-TGCTTAAA--T--AAGCTCTTTGCG
CTG34100         --------------------------------------------------------------------------------
IES169           TTAA-TTATTTATTCTAATTCTTCAGTTAAATTTGATAATTTATGCAATTTTTT-TGCTTAAG--T--AAGATCTTTGTG
IES103           TTAATTTATTTATTCTAACTCTTTAGTTAACTTTGATAATTTAAGCAATCTTTCTTTTTTAAA--T--AAACT-------
CTG2630          --------------------------------TTAATAATTTAAG-----------------------------------
CTG45590         TTAATTTATTTATTCTAACTC-CCAGTTAACTTTGATAATTTAAGCAATTTTTC-TGCCTAAA--T--AAGCTCTTTGCG
AnchoisB    1043 TTAATTTATTTATTCTAACTCTTCAGTTAACTTTGATAATTTAAGCAATTTTTT-TGCTTAAA--T--AAGCTCTTTGCG
```

```
CTG36069          ----TGTTTTTTTTTTGC-TACATCT------------------------------------------------------
IES85             --------------------------------------------------------------------------------
IES57             ATCCTGCATTTTCTTTGC-AGCATATTATTTAAATTTCTCAACTTGATCCTTCGTTCTGCAA------------------
IES214            --------------------------------------------------------------------------------
IES105            --------------------------------------------------------------------------------
IES47             ---CT-CTTTCGCTTTGC-TGCATCTCATTTAAATTTCTCAAATTGCTCCTTCGTTCTGTGTAAACATCTCATTCCATCC
IES80             --------------------------------------------------------------------------------
CTG4797           --------------------------------------------------------------------------------
IES77b            AG---------------C-TGCATCTTATTTAAATTTCTTAACTTGCTCCTTCTTTCTGCAATAATATCTCA----ACT-
CTG826            --------------------------------------------------------------------------------
IES132            AACCTGCTTTTTCTTTAA-TTCACCTTA---AAATTTCTAAACTTGCTCCTTCGTTCTGCTGAAATATCTCA----TCT-
CTG7743           --------------------------------------------------------------------------------
CTG43276          AGCTTGCTTTTTATTTGC-TACATCTTACTTAAATTTCTCAACTTTCTCCTTCATTCGGAGGAAATATCTCA----ACT-
IES42b            AACCTGCTTTTTCTTTGCCTGTTTCTAA-TTAAATTTCTCAACTTGCTCATTTATTCTGCAAAAATATCTCA----ACT-
IES35             ----------------------------------TTAC---ACTTGCTCCTTCTTTCTGCGGAAATATCTCA----AAT-
IES104            AGTCTGCTTTTTCTTTGC-TGCATCTTATTTTAATTTCTCAACATGCTCCTTCGTTCTGCGGTATTATCTCA----ACT-
IES76             AGGCTGCTT-----TGGC-TGCATCTTATTTAAATTTCTCAACATGATCCTTCGTTTTACAAAATATTTTCA----ACTA
CTG13940          --------------------------------------------------------------------------------
IES42             AGCA-GGTTTTACTTAGC-TGCATCTTCTTTAAA-TTCTCAACTCGCTTCTTCGTTCTTCGGAAATATCTCA----ACT-
CTG34100          --------------------------------------------------------------------------------
IES169            AGCCTGCTTTTTCTTTAC-TGCATCTTATTTAAATTTCTCAACTT-----------------------CTCA----AC--
IES103            --------------------------------------------------------------------------------
CTG2630           --------------------------------------------------------------------------------
CTG45590          AGAC-GCTTTTTCGTTTC-TGCATCTTATTTAAATTTCTCAACATGCTCCTTCGTTCTGCGGAAATATCTCA----GCT-
AnchoisB     1118 AGCCTGCTTTTTCTTTGC-TGCATCTTATTTAAATTTCTCAACTTGCTCCTTCGTTCTGCGGAAATATCTCA----ACT-

CTG36069          -------------------------TAATTAATGAATGAAACTTTTAAATTCAAATAACCTTTCAAACAG-ATCATCTTA
IES85             -------------------------------------------TCTTAATTGAAATAACTTTTTCATTAG-ATAATTTTG
IES57             --------------------------------------------------------------------------------
IES214            --------------------------------------------------------------------------------
IES105            --------------------------------------------------------------------------------
IES47             TTTTGTTTCTTCAGATAGATATATCTAATTAATGAAT-ATAT-TTTGAATTGAAATAGCTTTTATATTAG-ATCATCTTG
IES80             --------------------------------------------------------------------------------
CTG4797           --------------------------------------------------------------------------------
IES77b            TTTTGTTTCCTCCAATATATGTATCTAATTAACGAATCAAACTTTTTAATTGGAATAACTGTTACATTGG-ATTATCTTG
CTG826            --------------------------------------------------------------------------------
IES132            CTTTGTTTCATCCAATAAATGGATCTAATTGACAAATCAAAC-TTTTAATTGAAATAACTGTTACATTAGAATTATCTTG
CTG7743           --------------------------------------------------------------------------------
CTG43276          T-TAGTTTCTTCCAATAGATGTATCTAATTAACGAATCAAAC-TTTTAATTGAAATAACTGTTATATTAG-AATATCTTG
IES42b            TTTTGTTTCTTAGAATAGATGTATCTAATTAATGAATCAAAC-TTTTAATTAAAATAACTGTTACATTAG-ATCATCTAG
IES35             TTTTGTTTTTCCCAATAGATTTTTCTAATTAATTAATCAAAC--TTTAATTGAAATAACTGTTATATTAG-ATCATATTG
IES104            TTTTGTTTCTTCCAATATATTTATCTAATTAATGAATCAATC-TTTTAATTGGAATAACTGTTATATTAG-ATCATCTTG
IES76             CTTTGTTTTTTCCAATAGATGTATCTAATAAAGGAATCAAAC-TTTTAATTGAAATAACTTTTACATTAG-ATCATCTTG
CTG13940          --------------------------------------------------------------------------------
IES42             ATTTGTTTCTTACGATAGATATATCTAATTAACGAATCAAAC-TTTT-----AAATAACTGTTAGATTAG-ATCATCTTG
CTG34100          --------------------------------------------------------------------------------
IES169            TTTTGTTTTTTCCAATGAATGTATCTAATTAACGAACCAAAC-TTTTAATTGAAATAATTGTTAAATTTG-ATCATCTTG
IES103            --------------------------------------------------------------------------------
CTG2630           --------------------------------------------------------------------------------
CTG45590          TTTTGTTTCTTCCAATAGATGTATCTAATTAACGAATCAAAC-TTTTAATTTAAATAACTGTTACGTTAG-ATCATCTTG
AnchoisB     1192 TTTTGTTTCTTCCAATAGATGTATCTAATTAACGAATCAAAC--TTTAATTGAAATAACTGTTACATTAG-ATCATCTTG

CTG36069          TGTAA-ATTTGATTAATACTTCATTTT-GGGTTCTTGAATAAAGAATGATTAGGATCTATCG-TTTTAAGAATATTGACA
IES85             CGTAAGATTTTATTAATTTTGTGCATTTGGGTTGTTGAATTAAGAATAATTCGGGTCTATTG-TTTTAAGAATATTGACT
IES57             --------------------------------------------------------------------------------
IES214            --------------------------------------------------------------------------------
IES105            --------------------------------------------------------------------------------
IES47             CGAATGA-TTGATGAATCTTGCAATTTTGAGTTCTT-AGTAAAGAATAATTCGGATCCATCA-TTTTAAGATAATTGACT
IES80             --------------------------------------------------------------------------------
CTG4797           --------------------------------------------------------------------------------
IES77b            CGTAAGATTTGATTAATCTTGCATTTTAGGGTTTTTGAATAAAGAATGATTCGGATCTATCG-TTTTAAGAATATTGACT
CTG826            --------TGGATTAAACTTACGCTTT-GGG-TTTTGAATAAAGAATGATTTTAATCTATCGCTTTTATTTATATCGACT
IES132            GGAGAAATTTGATTAATCTTGCACTTT-TGGGTCTCGAATAAAGAAAAATTTGGATCTATCG-TTTTAC-AATATTGACT
CTG7743           --------------------------------------------------------------------------------
CTG43276          CTTAAGATCTGATTTATCATACACTTTTTGGTTCTTGAATAGAGAATGATTTGGATCTATCT-TTTTAAGAATATTGACT
IES42b            CTTAAGACTTGATTAATCTTGCACTTT-GGGTTCTTGAATAAAGAAGGTTTTGGATCTATTG-TTTTAAGGATATTGACT
IES35             CATTAGATTAGATTAATCTTGCGCTTTTTGGTTCTTGAATAAAGAATGATTTGGATCTATCG-CTTTAAGAATATTGACT
IES104            TGGAAGGTTTGATTAATCTTGCATTTTTTGATTCTTGAATAAAGAATGATTCGGATCTATCG-TTTTAAGAATGTTGATT
IES76             TGTATGATTTGATTAATCTTGCACTTG-AGGTTCTTGAATAAAGAATGATTTGGATTTATCG-TTTTAAGAAAGTTTAGT
CTG13940          --------------------------------------------------------------------------------
IES42             TGTAAGGTTTGATTAATCATGCACTTGGGGGTTCTTGAATAAAGAATGATTCGGATCTATCG-TTTTAAGAATATTGAAT
CTG34100          -------------------------------------AATAAACAATGATTTGGAATTATTG--TTTTAAAATATTGACT
IES169            CGTAAGATTTGATTAGCCATGCACTTTGGGGTTCTTGAATAAAGAATGAATCGGATCTACCG-TTTTAAGAATTTTG---
IES103            --------------------------------------------------------------------------------
CTG2630           --------------------------------------------------------------------------------
CTG45590          ATTAAGA-TTGATTAATGATGCACTTTGGGGCTCTTGAATAAAGAATGATTCGTATCTATCG-TTTTAAAAATATTG-CT
AnchoisB     1269 CGTAAGATTTGATTAATCTTGCACTTT-GGGTTCTTGAATAAAGAATGATTCGGATCTATCG-TTTTAAGAATATTGACT
```

```
CTG36069        G-A-CATTTATAATCATGGTGTTTTC-ATGGG-AAA---------------------------------TTTAAAT----
IES85           GTC-CATGTTTTATCAGAGTATCTTCAATCAGCAAAAGCCA------AGCTCCTAGCCATTTTGTTTCTTTTAAATCTTG
IES57           --------------------------------------------------------------------------------
IES214          --------------------------------------------------------------------------------
IES105          --------------------------------------------------------------------------------
IES47           GCC-CAATATTTATCAGTGTAGTTTCCATCAACAAA-ACCA------A-CACTTTGCCATTTAGCTCCTTTTAAGTCTCA
IES80           --------------------------------------------------------------------------------
CTG4797         --------------------------------------------------------------------------------
IES77b          ACC-CATATTTTATCAGAGTAGTTTCCATCACCATC-ACCATTGCCGAGCACTTAGCCAT-TAGTTTCAATAAAATCTCG
CTG826          GGC-TATGTTTTATCAGGTTAGTTTTCATCAGCAAA-GCCA------GCCTCTTAGACATATAGTTTCTTTTAAATCTGG
IES132          GTT-CTTGTTTTATCAGGGTACTTTCCATGAGCAAA-GCCA------AGCTCTTAGCCATTTAGTTTCTTT-AAATCTCG
CTG7743         --------------------------------------------------------------------------------
CTG43276        GCC-CATGTTTTATCAGAGTAGTTTCCATCATCAAA-GCCA------AGATCTTAGCCATTTAGTTTCTCTTAAATCTCG
IES42b          GCC-AATGTTTTATCAGATTAGTTTCCATCA-CAAA-GCCA------AACTCTTAGCCATTTAGTTTCTTTAAAATCTCG
IES35           CCCTTTTGTTCTATCAGGGTAGTCTCCATCAGTAAA-GCCA------AGCTCTTAGCCATTTAGTTTCTTTTCAATCTCG
IES104          GCC-CATATTTTATCAGGGTATTTTCCATCACCAAA-GCTA------AGCTCTTAGCCATTTAGTATCTTTTAAATTCTT
IES76           GCC-AATGTTTTATCAGGGTAGTTTAAATTGGCAAA-GTCA------AGCATTTAGCCATTTAATTTCTTTT-AATCTCT
CTG13940        --------------------------------------------------------------------------------
IES42           ACC-CATGTTTTATCAGGATGGTTTCCATCACCAAA-CCCA------AG--CTTAGCTATTTAGTTTCTTCTAAATCTCG
CTG34100        GCC-CATATTTTATCAGGGTAGTTTCCATCAGCAAA-GCCA------AGCTCCTCACCATTTAGTTTAATT-AAATCTTG
IES169          -----------------------------------------------AGCTCTTTGACATTTTGTTTCTTTTAAATCTCG
IES103          --------------------------------------------------------------------------------
CTG2630         --------------------------------------------------------------------------------
CTG45590        GCC-CAAGTTTTGTCAGGGTTGTTTCCATCACCAAA-GCCA------AGCTCTAAGCCATTTAGTTTCTTTTAAATCTAG
AnchoisB   1347 GCC-CATGTTTTATCAGGGTAGTTTCCATCAGCAAA-GCCA------AGCTCTTAGCCATTTAGTTTCTTTTAAATCTCG

CTG36069        --------------------------------------------------------TACTTGATTCCAACCTAAAAAATT
IES85           TGTAAA---TTTC-CTCTTTTGTTGTTGATCTTAGCAATATTCTTATTTGACCTTTTTTTTTATTCAAAACTAAACAATT
IES57           --------------------------------------------------------------------------------
IES214          -------------------------------------------TTATTTGAAC-TTTTTTTGATTCTAAGCTAAAATATA
IES105          ---------TTTCTCTCTTAATT---------------------------------------------------------
IES47           TGTAAAA--TTAC-CACTTTAGTTGCTGATCTTAGCAGTTATCCTAATTGA-C-CCTTTTTGATTCTAAGC---------
IES80           --------------------------------------------------------------------------------
CTG4797         --------------------------------------------------------------------------------
IES77b          TGTAAAA--CTTC-CTCTTTAGTTGCTGATCTTACCAATTCTCCTATTTGAAC-TTTTTTTGATTCTAAGCGTTAGCATT
CTG826          TGTATAA--ATTTCCTTCTTAGTTGCTGATCTT------------ATTTGAGC-TTTCGTAG--TATTAGCTTAAGCATT
IES132          TATAAAA--TATC-CTCTTTAGTTGCTAATCTTAGCAATTATCATATTTGAAC--TTTTTTGATTCTAAGATAAAACTTA
CTG7743         --------------------------------------------------------------------------------
CTG43276        TGTAAAT--TTTC-CTCTTTAGTTGCTTATCTTAGCAATTATCCTA------------TTTGATTCTAAGCTATAGCATT
IES42b          AGTAAAA--TTAC-CTCTTTAGTTACTGATCTTAGCAATTATCCTAATTGAAT-CTTTTTTGATT--------TAGAATT
IES35           TGTAAAA--TTTC-CTCTTTAGTTGTTGATCCTAGCAATCACACTATTTGAAC---TTTTTGATTCAAAGCTAAAGCATT
IES104          GTTAAAATTTTTTCCTCTTAATTTGTTGAACTT-----------------------------------------------
IES76           TGTAAAA--TTTC-CTCTTTATTTGCTGATCTTAGCAATTATCCTATTTGAAC-TTTTTTTGATTTTATGCTAAAGCAAA
CTG13940        --------------------------------------------------------------------------------
IES42           TGTAAAA--TTTTACTCTTTAGTTTCTGATCTTAGCATTTATCCTATTTTAAC-TATTTTTGATTCTCAGCTATAGCATT
CTG34100        TGTAAA---TTTT-CTCTTTAGTCGTAGATCATAGCTATTATCCTATTTGAAC-TTTTTTTGATTCTAAGCTAAAGCATT
IES169          TGTAAAA--TTTC-CTCTTTAGTTTATGAACTTAGCTATTATCCAATTTGAAC-TTTTTTTGATTCTAAGCTATAGCATT
IES103          TGTA--A--TTTC-CTTTATAATTGCTGATCTTAGCAATTATCCCATTTGATC-TTTTTTTGATTCTAAGCTATAGCATT
CTG2630         TGTAAAA--TTTT-CTCTTTTATTGCTGATCTTAGCAATTATCCTATTTGAAC--TTTTTTGATTCTAAGCTATACCATT
CTG45590        TGTAATA--TTTC-CTCTTTAGTTGCTGATCTTAGCAATTATCCTATTTTAAC-ATTTTTTGATTCTAATCTATAGCATT
AnchoisB   1419 TGTAAAA--TTTC-CTCTTTAGTTGCTGATCTTAGCAATTATCCTATTTGAAC-TTTTTTTGATTCTAAGCTATAGCATT

CTG36069        TT---GAAGTAACT-------ATTA-TATGC--TGTTACATCTGATGAATGGATAAAG-GGCAAGTAATATTCTGCAAAG
IES85           TTATTGTAGCAACTCTGAGCCGATATTACGC--TGTTACACATGAAGAATGGATCGCG---------ATATCCCGCTAAT
IES57           --------------------------------------------------------------------------------
IES214          TTTT-GTACAAACCCCGAGCAAAAGTTACG--------------------------------------------------
IES105          --------------------------------------------------------------------------------
IES47           --------------------------------------------------------------------------------
IES80           --------------------------------------------------------------------------------
CTG4797         --------------------------------------------------------------------------------
IES77b          TTTA-ATAGCAACTCCGAGCAAATATTATTC--TGTTACATCTGAAGAATGAATCGCG-GGCAAATAATATCCCGCTAAG
CTG826          TTTT-GTAGTAACTCTGAGCAAATATTACACTTTTTTTACACCTGAAGAATGGATCTCT-GGTATAAAATAT-CGACTAAA
IES132          ATTTTGTAGCTACCCCTAATAAATATTAATC--TGTTACACCTGAAGAATTGATCGCG-GGCAAATAATATCCCGCTAAG
CTG7743         --------------------------------------------------------------------------------
CTG43276        TTTT-GTAGCAAAACTGAGCAGATATTATGC--TGCTACACCTAAAGAATGGATCTC--AACAAATAATATCCCGCTTAG
IES42b          TATTT-TAGTAACTCTGAACAA-TGTTATGC--TGTTACTCATGAAGAATGGATCGCG-GACAGATAA------------
IES35           GTT--GTTGCAACTCTGAGCAAATCTTACT---TGTTACACCTGAAGAAGGGATCGAG-GGAAAATATTATCCTGCAAAG
IES104          -----------------------------------------------------TCAGG-GACAAATAATATCAAGCTAAG
IES76           TTTT-GTCGTTGCTCTGAGCAAATATTAAGC--TGTTACACCCGAAGAATGGATTGTA-GGCAAATAATGTACTGCTAAG
CTG13940        --------------------------------------------------------------------------------
IES42           TTTT-GTAGTAACTCTGAGCAA-TAGTAAGC--TGTTACAACTGAAGAATGGATTGCG-GGCAAGTA--------CTTAG
CTG34100        TTGTTGTAAAAACTCTGAGCAAGTAGTTCGC--TGTTTAATCTGAAGAATGGATCGCGAGGCAAATATTATCCTACTAAG
IES169          TTTT-GTAA-AACTCTGAGCAAATATTAAGC--TGTTCCACCAGAAGAAAGGATCGC--TGCAAATAATATCCCACTAAG
IES103          TTTTTATAGCAAATCTGAGCAAGTATTACGC--TGGTACACCTGAAGATAGGATCGCG-GGCAAATAATAT---------
CTG2630         TTTT-GTCGCAACTCTGAGCACGTATTAGGC--TTTTACACCTTAAGAATGGATCAAG-GGCAAATAACATCCCGTTAAG
CTG45590        TTTTT-TAGCAACTCTGAGCAAATATTACGC--TGTTATATCTGAAGAATGAATCGCG-GGCAAATAATATCCCGCTAAG
AnchoisB   1495 TTTTTGTAGCAACTCTGAGCAAATATTACGC--TGTTACACCTGAAGAATGGATCGCG-GGCAAATAATATCCCGCTAAG
```

```
CTG36069        ATTAATCG--GTAAAACATAAA----------------------------------------------------------
IES85           ATTAATCCATATAATAGATAGTTTCAACAGAATCAGTTGCTTAATTTTCTTGTAAAATGGAAATCATAAAGAATTAGAGA
IES57           --------------------------------------------------------------------------------
IES214          --------------------------------------------------------------------------------
IES105          ------------------------------------------------------------------------------GA
IES47           -----------TAATACATTGATT-AACAGAACCAGTTGATTAATTTGCTTGTAAAA-GGGAATCATAAAGAATTAGAGA
IES80           --------------------------------------------------------------------------------
CTG4797         --------------------------------------------------------------------------------
IES77b          ATTATTCC--ATT-TACATGGATTCTTCAGAACCAGT-------------------------------------------
CTG826          AATAATCC--TTAATATATAGATTCAAATGAACCAGTTGTTTAATTTGCTTGTAAAATGAAACT----------------
IES132          ATTAATCC--ATAATACATAGATTAAACAGAACCAGTTTCTTAGTTTATTTGTGAAACGAAATCATAAAGAATTAGA-A
CTG7743         --------------------------------------------------------------------------------
CTG43276        ATTAATCC--ATAATATATAGATTCAACAGAGCCAGTTTTTTGATTT-TTTGTAAAATGAAAATTATAAAGAATTCTAAA
IES42b          --------------------------------------------------------------------------------
IES35           ATAAATCC--ATAATACATAGATTCAACTGAAACAGTAGCCTAATTTGCTTGTAAAATGGAAATCATAAATAATTTTAGT
IES104          ATTAATCC--ATTATTCATATAGCCAACAAAACCAATTGCTTAATT-GCTAGTAAAATGGTAATCATAAAGAATTTAAAA
IES76           ATTAATCT--ACAATAAAAAGATTC------ATCAGTTGCTTAATTTGCTTGTAAAATGGAACTCATAAAGAAACCTAGA
CTG13940        --------------------------------------------------------------------------------
IES42           ATTAATCC--ATAATACATAGATTCAATTGAACCAGTTGCTTAATTTGCTTGTAAAATGGAAGTCATAAAGAATTCTAAA
CTG34100        ATAAATCC--ATAACACATAGATTCAACAGAACTTGT-------------------------------------------
IES169          ATGAATCC--ATAATATATGGATTGAACAGAACCAGTTGCTTATTTTGCTTTTAAAATGGAAATCATCAAGAATTCTAAA
IES103          --TAATCC--ATAATACATAGTATCATCAAAACCAGTTGCCTAATTTGCTCGTAAAATAGAAATCATAAAGAATTCTAAA
CTG2630         ATTAATCC--ATAATATATAGAATTAACAGAACCAGTTGCTTAATTGGTTTGTAAAATGGAAACCATAAAGAATTCTAAA
CTG45590        AGTAATCC--ATAATACATTGATTCAACAGAAGCAGTTGCTTAATTTGCTCGTAAAATGGAAATCATAAAGAATTCTAAA
AnchoisB   1572 ATTAATCC--ATAATACATAGATTCAACAGAACCAGTTGCTTAATTTGCTTGTAAAATGGAAATCATAAAGAATTCTAAA

CTG36069        --------------------------------------------------------------------------------
IES85           TCTTTGTTCAACTAATTTAAAAATGCAAATTCAAAA-TTTGTG-TTGAAAA-AAATATT--TTTCCAAC--CAAACAAA-
IES57           --------------------------------------------------------------------------------
IES214          --------------------------------------------------------------------------------
IES105          TTTTTATTCAATTAAATTTAAAATGCTAATGC-AAA-TTTAGA-TTGAAAA-AATTAT-CTTTTCCAAT--TAATTAAA-
IES47           TCTTTGCTCAATTACTTTAAA-ATGTAAAAGCAAAA-CTTATA-TTGAAAA-AAAAATTCTTCTCCAAC--CAATCAAA-
IES80           --------------------------------------------------------------------------------
CTG4797         --------------------------------------------------------------------------------
IES77b          --------------------------------------------------------------------------------
CTG826          --------------------------------------------------------------------------------
IES132          TCTTTGTTCAATTAATTTAAAAGTGCAAAAGCAAAT-TTTATA-TTGAAAA-AAA--CTCGTTTCCAAT--TAATCGGA-
CTG7743         --------------------------------------------------------------------------------
CTG43276        TCTTTGTTCAATTAATTTAAATATGCAAAAGTAAAA-TTTAAA-TTGAAAA-AAAAATTCTTTTCCAACCACAATCAAAA
IES42b          --------------------------------------------------------------------------------
IES35           TCTTTGTTCAATCAATTTATTAATGCAAAAGAAATG-TTTATA-TTGAAAATAAAAATTCTTTTCCAAC--CAATCAAT-
IES104          TCTTTGTTCG-TTAATTTAAA-ATTCAAAAGCATAA-CATATT-TTGAAAA-AAAAAATCTTTTCCAAC--CAATCAAA-
IES76           TCTTTTTTCGATCAATTTAAA-ATGTAAAAGCAAAA-TTTATATTTGAAAA-AAAACTTCTTTTCCAAC--CACTCAAA-
CTG13940        --------------------------------------------------------------------------------
IES42           TCTTTGTTCAATTAATTTAAAAATGC-AAAGCAATA-TTTTTA-TTAAAAA-AAAAATCCTTTTCCAAT--CAATCAAA-
CTG34100        ----TGTTCAATTGATTTAAAAATGAAAAAGGAAAA-CTTATA-TTGAAAA-AAAAATTCTTTGCCCAC--CGTTCATA-
IES169          TCTTTGTTCAATTAATTTAAAAATGCAAAAGCAAAT-CTTATA-TTGAAAA-AAAAT---TCTTTCAAC--CAATTAAA-
IES103          TCTATGTTCAATTAATTTAAAAATGCTAAAGCGAAA-TTTAT----------------TCTTTTCCAAC--CAATCAAA-
CTG2630         TTTTTGTTCAATTAATTTAAAAATGCAAAATCAAAAATTTATA-TTGAAAA-AAAAAAA-TTTTCCAAT--AAATCAAA-
CTG45590        TCTTTGTTCAATTAATCTAAATATGCAAAAGCAATA-TTTATA-TT-AAAA-AAAAATTCTCTTCCAAC--CGATCAAA-
AnchoisB   1650 TCTTTGTTCAATTAATTTAAAAATGCAAAAGCAAAA-TTTATA-TTGAAAA-AAAAATTCTTTTCCAAC--CAATCAAA-

CTG36069        --------------------------------------------------------------------------------
IES85           TTG-AAAGCA------ATTAACCCCAAATTGAAAA--------------ATAGAAATAACAAAGAATAAAATAA--TTA-
IES57           ----AAAGTA----------------------------------------------------------------------
IES214          --------------------------------------------------------------------------------
IES105          TTG-AAAGCAGTTGCAATTTATACCATAATAAAAA------------TAATATAAATAATAAAGTATAAAAATAACAAAA
IES47           TTG-AA----------ATTTATCGCAAATTAAAAA--------------ATATAAATAATAAAGAACAAAA-AAACAAA-
IES80           --------------------------------------------------------------------------------
CTG4797         --------------------------------------------------------------------------------
IES77b          --------------------------------------------------------------------------------
CTG826          --------------------------------------------------------------------------------
IES132          TTG--AAGCT------ATTTAACCCAATTAAAAAA-------------------AATATTAATGAACAAAA-AAATT---
CTG7743         --------------------------------------------------------------------------------
CTG43276        TTG-A--GCTTA----ATTTATCGCAAATTAAAAT--------------ATACATATAATAAAGAGTAAAA-AAAAAAA-
IES42b          ----AAAGTA----------------------------------------------------------------------
IES35           TTA-AAGGCA------ATTTATCACAAATTTAAA----------------TATTAATAATGAAGAATATACAAATTA---
IES104          TTG-AAAGCA------A--TATCTCCAATTAAAAT---------------TATAAAAAATAAAGAATAAAACGAACAAA-
IES76           TTG-AAAGCAT-----ATTTTAGGAAAATTAAAAA--------------ATATAAA-AATAAAGAATAAAACGAACAAA-
CTG13940        --------------------------------------------------------------------------------
IES42           TTC-AAAGCA------ATTTATCTCAAATTAAAAA---------------TATAAATAATAAAGAATAAAAAAAACAAA-
CTG34100        CTGAAAAGCA------ATTTATCTCAAATTAAAAAATATAAATAATAGAGAATAAAGAATAAAGAATAAAGCAAACAAA-
IES169          TCG-AAAGAA------ATTTATCGCAATTTAGAAA-------------CTTATAAATAATAAAGAATAAAA-AAACAAA-
IES103          TTG-AAAGCA------ATTTATCGCAAATTAAAAA--------------ATATAAATAATAAGGAATAAAACAAAT----
CTG2630         GTG-AAAGCA------ATCTATCGCAAATTAATAA--------------ATATAAATAATAAAGAATATAACAAACAAT-
CTG45590        TTG-AAAGCA------ATTTATTGCAAATT-ATAA--------------ATATAATTACTAAAGAATAAAATAAATAAA-
AnchoisB   1724 TTG-AAAGCA------ATTTATCGCAAATTAAAAA--------------ATATAAATAATAAAGAATAAAACAAACAAA-
```

```
CTG36069        --------------------------------------------------------------------------------
IES85           TTC----ATTTTTG-CTTTTAAGTATAAATT-ATATCTTAC----ATTTTTATTGGAACATATTTCTAATTTCA----TT
IES57           --------------------------------------------------------------------------------
IES214          ---------------------------AATT-ATAACTATC----ATTTTTATTGGA----TTTTTTAAATTTAAGATTT
IES105          TTA----ATTATTATTTTTTCAATTCAA--G-ATAAC-AGCTTATATTATTATTGGA--TCATTTTTAAATTCAAGATCG
IES47           TTAA---ATTATTATTTTGTAA-TATAAATTCATAACTTAC--------------------ATTTTTCCATTCAA-----
IES80           --------------------------------------------------------------------------------
CTG4797         --------------------------------------------------------------------------------
IES77b          --------------------------------------------------------------------------------
CTG826          ---------------------------------------------GTTTTTATTGTA--TTATTTTTAAATTCAAGA-TT
IES132          TTA----ATT-TT-TTTTTTAAATATAAATT-ATAATTAAC----ATTCATATTGGA--TTAT-GTTAAATTCAAGATTT
CTG7743         --------------------------------------------------------------------------------
CTG43276        TCA----ATTAGTG-CTTTTAAATATAAATT-ATAACTAGC----TTTC-----GGA--TAA-TTTTAAATTTAAGATTT
IES42b          --------------------------------------------------------------------------------
IES35           -------ACTATTATTTTATAGATACAAATT-ATATCTA----------------------------AAATTTAA-----
IES104          TTAATTAATTATTATTTTTTAAATATAAATT-ATAACTAA-----ATTCTTATTGGA--TTATTTTTAAATTCAAGA--T
IES76           TTA---------------TAAAATATAAATT-A-AATTAGC----ATTCTTATTGGA--TTAATTCTAAATTCAAGATTG
CTG13940        --------------------------------------------------------------------------------
IES42           TTA----ATTATTATTTTTTAAATAGAAATT-ATAACTAGC----ATTCTTATTGGA--TTATTTATAAATTCAAGATCT
CTG34100        TTA-TTTATTATTATTTTTTAAATATAGATT-ATAACTAAC----ATTGTTATTGGA---GTATTTTAAATTAAAGTTTT
IES169          TTA----ATTAATA-TTTTTAAATTAAAATT-ATAACTATC----CTTCTTATTGGA--TTATGTTTAAATTTAAGAGTT
IES103          TTA------TAGTATTTTTTAAATATATATT-ATAATTAGTA---ATTCTTATTTGA--TTAT-TTTATATTCAAGTATT
CTG2630         TAA----ATTACTATTTTTTAAATTTAAATT-ATAACAAGC----ATTCTTATTGGA--TTATTTTTAAATTCAAGATTT
CTG45590        TTA----ATTGGTATTTTTTAAATATAAATT-ATAATTAGC----ATTCTTATTGGA--TTATATTTAAATTCAAGATTT
AnchoisB   1782 TTA----ATTATTATTTTTTAAATATAAATT-ATAACTAGC----ATTCTTATTGGA--TTATTTTTAAATTCAAGATTT

CTG36069        --------------------------------------------------------------------------------
IES85           TTTTCATAAATTGACAAATATTTTTC-----------AGTCAATTTTTATTATTGGATGTTAGTGAGAATTTTAAAATAT
IES57           --------------------------------------------------------------------------------
IES214          TTTTTTTAAATTGAAAAAATATTTTTTC---------ATTCAATTTTTATCTTTGGATATTCATATGAATTTTGGAATAT
IES105          TTTTCAAAAATTTACAAAATATTTTTTC--------------ATTTAAATTATTGGATGTTGGCATGAATTTTAGAAAAT
IES47           --------------------------------------------TTTTATAATTGAAAGATATT-TGAATTTTGAA----
IES80           --------------------------------------------------------------------------------
CTG4797         --------------------------------------------------------------------------------
IES77b          --------------------------------------------------------------------------------
CTG826          TTTTCAAAAATTGACATTCTA-----------------------TTTTATAATTGGATTTTGCTATGAATTTTGGAATAT
IES132          TTT-----------------------------------------------------------------------------
CTG7743         -----------------------------------------------------------------------TTAGTATAT
CTG43276        TTTTTTTAATTTGACAAAATA-TTTTTCCATTCTATGATTCTATTGTTATTATTGGATTTGGTTATGAATTTTAGAATAT
IES42b          --------------------------------------------------------------------------------
IES35           ---------------------------------------------------ATTGGATATATATGTGAATTTTAGGATAT
IES104          TTTTCAAATAATGAAACAATA-TTTTTTC--------ATTCGATT-TTATAATTGGATGTTGGAATGAATCTTTTAATAT
IES76           TTTTCAAAAATTGACATTATA-TTTTTTC--------ATTTAATT-TTATTTTTGGAAATTGTTATTA------------
CTG13940        --------------------------------------------------------------------------------
IES42           TTTTT--AAATTGACAAATTA-TTATTCC--------TATCCATTGTTACTATTAGATGTTACTATCAATTTTAGAATAT
CTG34100        TTTTCAAAAATTGACAAAATATTTTTCCA---------------------------------------------------
IES169          TTTTTT-AAATTTACAAAATATTTTTTCC--------AATCAATTGTTAATATTGGATGTTGGTATGAATTTTAGAAAAT
IES103          TTTT--AAAATTGACAAATTA--TTTTCC--------ATTCAATTGCTATTATTGGATGTTGGTATAAATTTAAAAATAT
CTG2630         GTTTTT-AAATTGACAAAAT-----------------TTACAATCGTTATTATTGGATGTTGGTATGAATTT-AGAATA-
CTG45590        TTTTTTTAAATTAACAAAATA--ATTTCC--------ATTCAATTGTTATTATTGGATGTTTGTATGTATTTTAGAATAT
AnchoisB   1851 TTTTCAAAAATTGACAAAATA-TTTTTCC--------ATTCAATTGTTATTATTGGATGTTGGTATGAATTTTAGAATAT

CTG36069        --------------------------------------------------------------------------------
IES85           -CAAATCTTATTCATTGTAAATATTTCTATCATTTCATATTTGAATGTAAA-TAGAAACTCCAGCTTCAA---CTTTTAT
IES57           --------------------------------------------------------------------------------
IES214          AAAAATCATACTCATTGCAAATAATTCTATTATATCATAATTAAATGTAAA-TAGAAACTCAAGCCTCAACTTCTTCTCT
IES105          -CAACACTTATTTATTGCAAGTAATTCTATTGTTTCTTCATTTAA-----A-TTGAA-C-----------CTTCTTCTAT
IES47           --------------------------CTATTACTTCAAAATTTGATGTAAA-TCGAAGCTCTAGGTTCAAATTCTTCTCT
IES80           --------------------------------------------------------------------------------
CTG4797         --------------------------------------------------------------------------------
IES77b          --------------------------------------------------------------------------------
CTG826          --------------------------------------------------------------------------------
IES132          --------------------------------------------------------------------------------
CTG7743         -GAAATCTTATTTATTGCAAATACATCTATTATTCTATAATTTA-TGTAAAATGGCAACTCCAGCTTCAACTTCATCGCT
CTG43276        -GAGATCTTATTAATTGCAAATAATTCTATTATTTCATAATTTAATGTAAA-TAG-ATCTCCAGCATTAACTTCTTCTCT
IES42b          --------------------------------------------------------------------------------
IES35           -CAAATCTTATTTATTGCAAATAATTTT-TTATTTCATAATTTAATGTAAA-TAGAAACTCCAGAATCAAATTCTTCTCT
IES104          -GAAATCTTATTTATTTCAAATTATCCTCTTATTTCATAATATAATATAAA-AAGAAACTTCAGCTTCAACTTTTTCTTT
IES76           ---AATTTTATT-ATTACAAATAATTCTATAATTTCATTATTTAATGTAAA-TAGGAACTCCACCTTGAACTTATTCCCT
CTG13940        --------------------------------------------------------------------------------
IES42           -GAAATCTTATTAATTGTAAATAATTCTATTATTAAATAATATTTTGTAAA-TAGAAACTCCAGCATCAACTTCTTCACT
CTG34100        -TTTAATTTATTTAATGCAAATAATTCTATAATTTCATAATTTAATGTAAA-TAGAAACTCCAGCTTCTAAATCTTCTTT
IES169          -G-AATCTTATTTATTG-AAATAAGCCTATTATTTCATAATTTAATGAAAA-AAGGAACTCTAGCTTCAACTTCTTCTCT
IES103          -GAAATCTTATTTATTGCAAATAATTCTATTATTTTATAATTTAAAGTAAA-TAGAAACTCCAGCTTCAATTTCTTCTCT
CTG2630         -AAAATCATATTTATTGCAAATAATTCTATTATTTCATAATCTAATGTAAA-TAGAAACTCCAGCTTCAACTTCTTCTCT
CTG45590        -GAAATTTTATTTATTGCA--------TATACTTTCATAATTTAATGCAAA-TAGAAACATCAGCTTCAACTTCTTCTCT
AnchoisB   1922 -GAAATCTTATTTATTGCAAATAATTCTATTATTTCATAATTTAATGTAAA-TAGAAACTCCAGCTTCAACTTCTTCTCT
```

```
CTG36069         --------------------------------------------------------------------------------
IES85            GTAATAGTATTATTTACTATAGCAGGCCCGACATTTAAAATAAATTAAATAGATATGCAAACAATGATGACTGTCCATAA
IES57            --------------------------------------------------------------------------------
IES214           GTAATAATATTATT-GTTATAGTAGGCCTTACATGTAAATCAGATTGTTT-----------------------------A
IES105           ATAA-AATATTATTCGCTATA---GGCCTTACTTATAAACTACATTATATAAATATAGATGCAATGATCATTACCAATAA
IES47            GAAATAATATTATTTGCTGTCGTAGGCCTTACACTTAAACTACATAATGTAGATGCACATGAAAAGATGACTGTCACTAA
IES80            --------------------------------------------------------------------------------
CTG4797          --------------------------------------------------------------------------------
IES77b           -------------------TAGCTGGCCTTACACTTAAACTACATTATGTAGATGTATATGAAATGACGACTGTCAATAA
CTG826           --------------------------------------------------------------------------------
IES132           --------------------------------------------------------------------------------
CTG7743          GTAATGATATTATTTGCTATAGCAGGCCTTGCAATTAAACTTCATTAAATAGACATGCACACAGAGATGACTATCAATAA
CTG43276         GTAATAATATTCTTTGCTATAGCAGGCC-TACACTTAAATAACATAATGTAGACATACATGCAATAATGACTGTCGATAA
IES42b           --------------------------------------------------------------------------------
IES35            GAAGTAATATTATTTGCTATAGTAGGCCTTACACTTCAACTACATTATGTAGACATATTTGCAATTATAACTGTCAATAA
IES104           ATAAGAAGAAATTTTGCTGTAGTAGGCCTTACACTTAATCCAAATTCTGTAGACATACTTACAATGATGACTGCCAATAA
IES76            TTAATAATATTTTTTGCAATAGCAG-CCTTATGTTTAAACTACAAAATGTAGACGTAAATGCAATGATGACTGTCAATAA
CTG13940         --------------------------------------------------------------------------------
IES42            GTAATATTATACTTTGATATA-CAGGTCTTACACTTTTGTTACATTATGTAGATATACATGCAATGA---CTGTCAATAA
CTG34100         GTAATAATATTATTTGCTATAGCAGGC-----------------------------------------------------
IES169           GTAATAATATTCTTTGCTATAGCAGGCCTTAAACTTAAGCTACATTATGTAGACATACATGTAATGATGTCTTTCTATAA
IES103           GTAATAATATTCTTTGCTATTGAAAGCCTTACACTTAAGCTACATTATTTAGACATACATGCAATGATAATTGTCAATAA
CTG2630          GTTATCATATTCTTTGCTATAGCAGTCCTTACGCTTAAGCTACGTTATATAGACATACATGATATGATGATTGTCAATAA
CTG45590         GTAATAATATTCTTTGCTATAGCAGGCCTTAAACTTAAGTTCCATTATGTAGACTTACATGCAATGATGACTGTCAATAA
AnchoisB    2000 GTAATAATATTATTTGCTATAGCAGGCCTTACACTTAAACTACATTATGTAGACATACATGCAATGATGACTGTCAATAA

CTG36069         -------------------------------------------------------------------------GA-----
IES85            TATAGTTAA---ACTATGTATATGAAAAAACGA----TTTTCT-TCAAAA--AACCAGA-AAATTCAAGAGTTGATAACT
IES57            --------------------------------------------------------------------------------
IES214           TGTTATCAA--ACCTATGTGTATGAAAAATGA------TTTCC-TCTAAACAAAATAGC-AGATGCAGGATTTGATTACT
IES105           ATTTATTAA--ATCTATATGTACGAATAAAAAAAAA--TTTCA-TCAAAATAAA-CAGG-AAATGCATGAATTGATTACC
IES47            ATTTATTAA--ATCTATGCGTATGTAGAAAAAAGA--TTTT-----------AAACGGC-ACATGCAGTAATTGATTACT
IES80            --------------------------------------------------------------------------------
CTG4797          --------------------------------------------------------------------------------
IES77b           TTTTATTAA--ATCTATGTGTATAAAAGAAAAAA---TTTTCA-TCAAAACAACATAGC-AATTACAAGAATTGATTATT
CTG826           --------------------------------------------------------------------------------
IES132           --------------------------------------------------------------------------------
CTG7743          TTGTATTAA--ATCTAATTGAATGAAAGAAA------TTTTAG-CCAAAACAAAACA---ATATGCATGAATTCATTTCT
CTG43276         TTTTATTAA---CAAATGTGTATGAAAAAAAAGA---TTTTCA-TCAAAA-ACAACAGC-AACTGCAGGAATTGATTACT
IES42b           --------------------------------------------------------------------------------
IES35            TTTTATTAA--ACCTATGCGCAAAAAAAAACAA----TTTTCA-TCAAAGCATAGCAGC-AATTTCAGGAA---------
IES104           CTTTATTAA--ACCTATGCGGATGAAAAAAAAGA---TTTTTA-TCAAAACAAAACAGC-AATTCGATTAATTCATTACT
IES76            TTTTATTAA--ACCTATG----TGAAAGAAAAAAAACTTTTCA-TC-AAACAAAACAGC-AATTACAGGAGTTGATTACT
CTG13940         --------------------------------------------------------------------------------
IES42            TTTTGTTAA--ACCTATGTGTATGAAAAAACAAAAGATATTTA-TCAAAACAAAACAGC-AATTGCAGGAATTGATTACT
CTG34100         ---------------------------------------TTCA-TCAAAACAAAAAATTAAAATGCAGGAATTGATTATT
IES169           TTTTATTAA--ATCTAAATGTATGAAAAAAAAAAGA-TTTTCAAAAAAAATAATACAGC-AATTGCAGGAATTGATTACT
IES103           TTTTATTAACCCCCCATGTGCATGAAAAAATAAATA-TTTTCA-TCAAAACAAATCAGC-AATTGCAGGAATTGATTACT
CTG2630          -TTTATTAA--ACCTGTGTGTATGAAAAAAAAAGA--TTTTCA-TCAAAACAAAATAGCAAATTGCAGGAAATGCTTACT
CTG45590         TTTTATTAA--ACCTATATGAATG-AAAAAGAAATA-TTTTCA-TCAAAACAGAACAGC-AAT--------TTGATTGAT
AnchoisB    2080 TTTTATTAA--ACCTATGTGTATGAAAAAAAAAAAA-TTTTCA-TCAAAACAAAACAGC-AATTGCAGGAATTGATTACT

CTG36069         ------------------ATTATATA------------------------------------------------------
IES85            TTCTATGACGC---CATAAATAACTAG-CAAAA-TTATAAAT--AATAAGGGATGTAATACTAAGAAAAA---GTTTGGT
IES57            --------------------------------------------------------------------------------
IES214           TTGGATAATGCACTTAAAACTATATAG-CAAA-TTTATAAAC---AATAAGGAAGTACCACTGAGAAAAA-G-ATTCACT
IES105           TTGTATTATAAACTT-AAATTATATAC-CAAAAATTAGAAA---GTAAAGGGAAGAAACCCTAC----------------
IES47            TTGGATGATGCAAAT-AAATTATATAG-TAAAACTTATAAA---AACAAGAGAAGAAAGCCTTAAGAAAAGCGATTAACA
IES80            --------------------------------------------------------------------------------
CTG4797          --------------------------------------------------------------------------------
IES77b           TTGGATGTTGAGCCTAAAATTA------TAAAAA----------AAAAAGGAATGAGACTCTAAGATAAAGG--------
CTG826           ----------------------------AAAAAATTATCAAC--AAAAAGGGAAGAAATGTAGAGAAAAAAAACTACCAA
IES132           ----------------------------CAAAAACTAAAAT---------------------------------------
CTG7743          TTGGATGATGCACTTAAAATTAAATAATCAAAAATTCTAAAC--AAAAAGGGAAGAAAACTTAAGAAAAAAG-ATAAACA
CTG43276         TAGGATGATGTGCTTAAAATAATAT----AAAAATAATAAA---AAAAAGGTAAAAAAGGCTA----------ATTGACA
IES42b           --------------------------------------------------------------------------------
IES35            ----------------------------------------------------GAAGAAACACTAAGAAAAAAG-ATTAACA
IES104           TTGGATGATGCGCTTAAAATTAACTAG-CAAAAATTATAAACAAAAAAAGGGAAGAAGCGCTAAGAAAAA-G-ATGAACA
IES76            TTGGATGATACGCTTAAAATTATATAG-CAAAA-TTATAAAC--A----------------------------ATTAA-A
CTG13940         --------------------------------------------------------------------------------
IES42            TTGGATGATGCGCTTAAAAATATATAC-CAAAAATTATAAAC--AAAAAGGGA-------------AAAAAG-ATTT-CA
CTG34100         TTGGATAACGGGCTT-AAATTATA----GCAAAAATATAAAC--AAAGAGAGAAGAAACTCTAAGAAAAATA-GTTAATA
IES169           TTGGATGATGAGC-TAAAATTATATAG-CAAAATTATAAACC--AAAAAGGGAAGAAACGATAAAAAAAAAAGATTAACA
IES103           TTAGACGATGAGCTT-AAATTATATAG-CAAAATTTCTAATC--AAAAAGGGAAGAAATGCTAAGAAAAAAGAATTAACA
CTG2630          TTTTATGATACACTTAAAATTATATAG-CAAAAATTATAAAC--AAAAAGAGATGAAACGCTAAGAAAAA-G-ATTAACA
CTG45590         TTGGATAATGAGCTTAAAAATATATAG-CAAAATTTATAAAC--ATAAAGGGAAGAAACTTTAAGAAAAAAG-ATTAAC-
AnchoisB    2155 TTGGATGATGCGCTTAAAATTATATAG-CAAAAATTATAAAC--AAAAAGGGAAGAAACGCTAAGAAAAAAG-ATTAACA
```

```
CTG36069        --------------------------------------------------------------------------------
IES85           AACATAAGAGAGTAAATATA-TTTATATC-TAAGTAAAATTCAAGTTAATGTAAATTGATTCAGCCTTTTAAACTCCTAA
IES57           ---------------------TCTCAATC---------------------------------------------------
IES214          AACCCAAGAGAGCAAATATA-TTTAAATCTTAACTAATTTTCAAGATAAAGTAAATAGGTTTAGAGTTAGGAACTTCCAA
IES105          ---------------------TTAAAAT----------------------------------------------------
IES47           -----------------ATA-TTAAAATCTTAAATAACTTTCAAGTTTAGGCAAATTATTTAGAATTCAGGAACTTCTAT
IES80           --------------------------------------------------------------------------------
CTG4797         --------------------------------------------------------------------------------
IES77b          --ATTAAAAAGTAAGTAAA-TTTAAATCTTAACTAGCTCTCAAATTCAATTAAATTGCTTAAGAATTAAGAACTTTTAT
CTG826          AAACTAAGATACCGAATATA-TTTAATTCTTAACTAACTTTCAAGCCAAAGTAAATTACTTCAGGATTAGAAACTTCTAT
IES132          --------------------------------------------------------------------------------
CTG7743         AATCTAACAGAGCAAATATATTTAAAATCATATCGAACTTTTAATTTAAAATAAATCGCTTCAGAATTAAGAACTTCTAT
CTG43276        AGCCTAAGTGAGCAAATATA-TTTAAACCTTAACTAACTTTCCAGTCAAAGTAAATTGCTTAAGAATTAGGAATTTCTAT
IES42b          --------------------------------------------------------------------------------
IES35           AACCTAGGAGAGAAAATATA-TTTAAATCTAAACTAACTTTCAAGTTAAAGTAAATCGCTTCAGAATTAGGAACTTCTAT
IES104          AACCTAAGAGAGCAAATATA-TTTAAATTTTAACTAAATTTTAAATTAAAGTAAGTTGCTTAAGTATTAAGAACTTCTAT
IES76           TACCTAAGAGAACAAATATA-TGCAAATCTTAATTAACTTTCAAGTTAAAGTAAATTGCTTCAGAATTAGGAACCTCTAT
CTG13940        --------------------------------------------------------------------------------
IES42           AACCTA----AGCAAATATA-TTTAAATCTTAACTAACTTTCAAGTTAAAGTAAATTGCCAACGAATAAGGAACTTCTAT
CTG34100        AACCTAAAAGAA-AAATATATTTTAAATCATAACTAATATTCAAGT-----TAAATTGCTTTAGATTTAGGAAATTCTAT
IES169          AACCTAAGAGAGCGAATATC-TTTAAATCTTAACTAACTCTCTTGTTAAAGTAAATTGCTTACGAATTAGGAACTTCTAT
IES103          AACCTAAAAGAGCAAATATA-TTAAAATATTAACTAACTTTCAAGTTAAAGTATATTCCTTAGGAATTGGGAACATCTAA
CTG2630         AACCTAAGAGAGCAAATATA-TT-AAATCTTAACTAACTTTAAAGTTAACGTAAATTGCTTACGAATTAGGAACTTCTAT
CTG45590        AATATAAGATAGCAAATATA-TTT-AATCTTAACTAACTTTCAAATTAAAGTAAATTTCATCAGAATTAGGAACTTCTAT
AnchoisB   2231 AACCTAAGAGAGCAAATATA-TTTAAATCTTAACTAACTTTCAAGTTAAAGTAAATTGCTTAAGAATTAGGAACTTCTAT

CTG36069        --------------------------------------------------------------------------------
IES85           AAAACCTG-CAATTT-AGCTGGATATTTAGGAATTTCAATTGGAAGGACTAAATATCTAT-AAAATTAAATTCTATAGTA
IES57           --------------------------------------------------------------------------------
IES214          ---------TGATTTTGGTAGGATATTTTGGAGTCTTAATTA-------------------AAAAGTAAGTTAGAGATTA
IES105          ---------CAAATTTTACAAGATATTTGGGAATTTCAATTGGATAGGCTAAATATATAT---AAGTAAATTTGAGAGTA
IES47           AAAACCTA-TCATTTTTGCAGGATATTGAGGAATGTCAATTGGAAGGGC-GAATATAAAT-AAAAGAACGTTCTAGAATA
IES80           -----------------------------------------------------------------------------GT-
CTG4797         --------------------------------------------------------------------------------
IES77b          AAAACTTG-CAATTT-AGCAGGATACTTAGGAATTTCACTTCAAAGGGCTAAATATCTCT-AAAAGCATTTTCAAGAGTA
CTG826          AAAACCTGCAATATTTGGCAGAATATTTAGGATTTTCAAATAGGAGGGCTAAATATATAT-AAAAGTAAGTTCCAGAGTA
IES132          --------------------------------------------------------------------------------
CTG7743         AAAACCTG-TAATTTCTGCAGGATA-TTAGGAATTTCAATTTGAAATGCTAAACATCTAT--AAAATAAGTTCGAGAGTA
CTG43276        AAAACCTT-CAACTTTAGTAGGATATTTTGGAATTTCAAATGGAAGGGCTAAATATCTCT-AAAAGTAAATTCAAGAGTA
IES42b          --------------------------------------------------------------------------------
IES35           AAAACATG-CAATTTTAGTAGGATACTAAGGAATTTCAATTTTAAGTGCTAAATTTCTAT-AAAAGTAAGTTCGAGAGTA
IES104          AAAAACCTGCAATTTTAGCAGGATAAAAAGGGATTTCAATTGGAAGGCCTAAATATCCCT-GAAATCAAGTTGGAGAGTA
IES76           A-AACCTG-CAAATTTAGCAGAATAGTTAAGAATTTCAATTGGAAGG-CTAAATATCTATAAAAAGTTAGTTCAAGATCT
CTG13940        --------------------------------------------------------------------------------
IES42           ATAACCTG-CAATTTTAGCAGGATATTT--GAGTTTCAATTGGAAGGGCTAAATATCTCT-AAGAGCAAGTTCGAGAGTA
CTG34100        AAAACCTGAAAATTTTAGCAGAATATTTAGGAATTTCAATTGGAAAGGCTAAATAGCTAT--------------------
IES169          AAAACCTG-CAATTTTAGCAGGATATATAGTAATTTCAATTGGAAGGACTAAATATTTCT-AAAAGAAAGTTCGAGATTA
IES103          AAAACCTG-AAATTTCAGTAGGATATTTAGGAATTTCAATTGGAAGGGCTAAATATCTT---AAGGTAAGTTCTAGAGTA
CTG2630         A-------------------------------------------------------------------------------
CTG45590        AAAACATG-CAATTTTAGCAGGATATTTAGGAATTTTAATTGGAAGGGCTAAATATCTCT-AAAAGTAAGTTCGAAAGCA
AnchoisB   2310 AAAACCTG-CAATTTTAGCAGGATATTTAGGAATTTCAATTGGAAGGGCTAAATATCTAT-AAAAGTAAGTTCGAGAGTA

CTG36069        --------------------------------------------------------------------------------
IES85           TCCTCATTCTCT---------AATTTACTTTTTTATTT-GATA--------AATAGCACAAATTATGGATTGCGA-ATTT
IES57           --------------------------------------------------------------------------------
IES214          TCCCTATTCTCTGAGTCGAT-AACTTA-TTTTTCATCT-AAAG--------AATACTACAAATTATGGATTGAATCATTT
IES105          TCCCGAATCTCTGAGTCAAC--------------------------------ATAGAACAAATTATAGAATAAAAACCTG
IES47           CCCAGGTTCTTTGAGTCAAT-AATTTGCATTT-CATTTTGATG--------AATAGCATGAATTAAGGATAGAAAATTTG
IES80           TTCCGATTCTCTGAGTTAAC-GAG-TGCTTTTTCATTT-GATG--------AATAGCACTAATTATGGATTGGAAAATTT
CTG4797         --------------------------------------------------------------------------------
IES77b          TCCCGATTCTCTGTG------------------------------------AATAGCAAAAATTATGGATTGTAAAATTC
CTG826          TCCAGATTCTCTGATTCAAT-AATTTGCTTTT-CATTT-GATG--------TATAGCACATG------------------
IES132          --------------------------------------------------------------------------------
CTG7743         TCCCAATTCTCTGATTCAAC-GATTTGCTTTTTCATTT-TATG--------AATAGCATAA---------------ATTT
CTG43276        T----GGATTCTCACTCAAT-AATTTGCTTTATCATTT-GATG--------AATAGCACAAATTATGGATTGAAAATATA
IES42b          --------------------------------------------------------------------------------
IES35           TCCCGATCCTCTAAGTCAAT-AATTTGCTTTTACAGTT-GATG--------AATAGTGCAAGTTATGAGTTGAAA--TTT
IES104          TGCCGATTTTCTGAGTCAAT-AATTTGGTTTTTCATTT-GATT-------TAATAGCACAAATTATGGATTGAAA-ATTT
IES76           CCC--ATCCTCAGAGTCAAT-AATTTGCTTTTTCATTT-TATG--------AATAGCACAAATTATGAATTGAAAAATTT
CTG13940        --------------------------------------------------------------------------------
IES42           TCCCGATTCTCTGAGTCAAT-AATTTGCTTTTTAATTT-GATG--------AATAACACAGATTATTGATTGAAAAATTT
CTG34100        --------------------------------------------------------------------------------
IES169          TCCCGATTCTCCGAGTCAAT-AATTTGCTTTTTCATTT-AATG--------AATAGCATAAATTATGGATCCAAAGATTT
IES103          TCCCGCTTTTCTGAGTCAGTAAATTTGCTTTTTTATTT-GAAG--------AATAGCTCAAATTATGGACGGAAAAATTT
CTG2630         --------------------------------------------------------------------------------
CTG45590        TCCCGATT---TGAGTCAAT-AATTTGCTTTTTCATTT-GATGATTTGATAAATAGAAC-AATTATGGATTGAAAAATTT
AnchoisB   2388 TCCCGATTCTCTGAGTCAAT-AATTTGCTTTTTCATTT-GATG--------AATAGCACAAATTATGGATTGAAAAATTT
```

```
CTG36069         --------------------------------------------------------------------------------
IES85            CTAGCTTTAGATAATGTTGCACCCTTGGG---TGTAAAACAAATATAATCAGAATTCA-TTGTAAATTTTTCACATAATT
IES57            --------------------------------------------------------------------------------
IES214           GTAGCATAAGATAGTGTAACGCAATTGAACAGTACAAAACAAATATATAAAGAATTCGTTTATAAATTTTAATATTAATC
IES105           ATAGACTAATTTAATTTTACACAATTGAGCA-TG-AAAA--AATACAAGAAG-ATTCA-TTGTTAATTTTAATATTAATA
IES47            GTAGTCTAAGATGATGTTGCACACTTGAGCAGTGCAAAACATATGGATTAGAAATTCA-TGGTAAATTT-CATATTAATA
IES80            GTAGTTTAAGATAATGTTGCACACTTGAGCAGTGCAAAAT-AATATATTAGGAATTCG-TTGTAAATTTTCAAATTAATA
CTG4797          --------------------------------------------------------------------------------
IES77b           GTAGCATAAGATAATGTTGCACACT-----------------------AAAGAATTCG-TTGTGAATTTTCATATTAGCA
CTG826           ------------------------------------------------TAAGAATTCA-TTGTAA---TTCATATTAA--
IES132           --------------------------------------------------------------------------------
CTG7743          TTACTTTAAGATAATGTCGTACACTTGAGCAGAGCTATACAAATATATTAAGAATTCA-TAGTATATTTTCATATGAATC
CTG43276         GTAGCTTAAGATAAAGTTGCACACTTGAGCAGTGCAAAATAAATACTGTAATAAATCA-TTGAAAATTTTCATTTTCATC
IES42b           --------------------------------------------------------------------------------
IES35            TCAGCTTATAATAATGTTGCACACTTGAGTAGTGCAA-ACAAATATATAAGTATTTCA-TTGTAAATTTTCATATTAATC
IES104           TTAGCTTAAGTTAATGTCGCACACTTGAGCAATGAAAAACAAA-----TAAGAATTTG-TTGTAAATTTTTCACA-----
IES76            GTAACTTAAGATAACGTAGCACACTCGAACAGTG-GAAACAAATATATTAAGAATTCA-TTGT-AATTTTCAAATTAATT
CTG13940         --------------------------------------------------------------------------------
IES42            GTAGTT--------TGTTGCACATCTGAACATTGCAAAATAAATATATTAAGAATTCA-TTGTAAATTTTCATATTAATC
CTG34100         --------------------------------------------------------------------------------
IES169           TTAGTTTAAAATGATGCTGCACACTTGAGCAGTGCAA----AATATGTTTAGAATTCA-TAGTAAATTTTCATATTAAT-
IES103           GTAGCATAAGTTAATGTTACACACTTGAGCAATG------------ATTAAGAATTCA-TTGTAAATTT-CATATTAATC
CTG2630          --------------------------------------------------------------------------------
CTG45590         GTAACTTAAGATAATGTTGCACACTTGAGCAGTGCGAAAT-AATATATTAAGAATTCA-TTGTAAATTTCCATATTA--T
AnchoisB    2458 GTAGCTTAAGATAATGTTGCACACTTGAGCAGTGCAAAACAAATATATTAAGAATTCA-TTGTAAATTTTCATATTAATC
```

```
CTG36069         ----A------CAACTGCAATTTCATTAGTTTTTTTAATGGTTTAAGCAACTTGGGTTGTATAATTATCGCTTACTTAGT
IES85            AATAA------CAACTGCAATTTTAATAGCCTTTTTAGTGATTTAAGAAACTTATGCTGCATACTTATCGCCTACTTTG-
IES57            --------------------------------------------------------------------------------
IES214           AATAA------CAACTGCGATTTCATTCG-TTTTTTAGTCATTTAAGCAACCTGGGTTGTATATTGATGGCTTACTTAA-
IES105           AATAA------AAACTACAATTGTATTCATTCTTTTATTGGTTTAGGGAACTTGGGTTGTATATTAATCGCTAATTTTAG
IES47            AAATA-----ACAACTGCAATTTAATTATCTCATTTAGTAGTTTAAGTAACTACGGTTGTATACTTATAGGTTACTTAA-
IES80            AATAA------CAATTTTAATTCCATTCA-TTTTTCAATGGTTTAAACAACTTGAGTTGTATACTAATTGCTTACTTAG-
CTG4797          --------------------------------------------------------------------------------
IES77b           AATAA------CAACTGCAATTTTATTCA-TTTTTTAGTGTTTCAAACAACTTGGGTTGTATATTTATCACTTACTTAG-
CTG826           -----------------CAATTTTATTCA-TTTTTTAGTTGTTAAAGCAACTTGGGTTTTCTACTTATCGCTTACTTAG-
IES132           --------------------------------------------------------------------------------
CTG7743          AATAA------CAACTGCAATATCTTTCATATTGTTAGTGG-GTAAGCGACTTGGGTTGCATACATATCACTCACTTAG-
CTG43276         AATAA------CAAGTGCAATTTCATTAATTTTCTTAGTCGTTTAAG---------------------------------
IES42b           --------------------------------------------------------------------------------
IES35            AATAACAATTGCAATTGCAATTTCATTCTTTTTTTTAGTGGTATAAGGAACTTGGATTGT--------------------
IES104           --------------------------------------------------------------------------------
IES76            TATAA------CAATTGCCATTTAATTCACTTTTTTAGTGGTTTAAGCAAATTGGGTTGTATACTTATTGTTTATGTAG-
CTG13940         --------------------------------------------------------------------------------
IES42            AATAA------CAACTGCAATTTCATTAA-TTTTTTAGTGGCTTAAGCAACTTGGGTTATTTGCTTATTGCTTACTTAG-
CTG34100         --------------------------------------------------------------------------------
IES169           -----------------CAATTTCATTCATTTTTTTAGGGGTTAAAGTAACTTGGGTTATATACTTATCGCTTACTTAG-
IES103           AATAA------CAACTTCAATTTCA-AAATTTTTTTAGTGGTTTAAGACACTTGTTTTATATATTTATCACTTACTAAGT
CTG2630          --------------------------------------------------------------------------------
CTG45590         AATAA------CAACTGCAATTTCATTAA-TTTTTTAGTGGTTTAGGCAACTTGGGTTATATAATTATCGCTTACTTAG-
AnchoisB    2537 AATAA------CAACTGCAATTTCATTCATTTTTTTAGTGGTTTAAGCAACTTGGGTTGTATACTTATCGCTTACTTAG-
```

```
CTG36069         TTTGTTTATTTGGAGTTATAAAACC--AATGCTATTTAGAAAGTTGATTTGAGTTTGCAAAATAATTAACTAATCTTTTA
IES85            TTGCTTTAATTTTA-ATCTCAAACA-AAATGAACTTGAGTTAGAAGATTTTAATTTGCAAAATAATTAACTACCCATCTA
IES57            --------------------------------------------------------------------------------
IES214           TTGATTTAATTGTAAAACTCAAACC-AAATGCTCTTTAGAAAGATGTTTTGAATTTACA-AATAATTAACTAATCGTCTA
IES105           TTGCTTTAATTTTAAATCTCAAACCAAAATTTTCTTTAGAAAGAAGA-TTGGATTTGCAAAATAATTAA-TACTGTTCTA
IES47            TTGAATTAATTT-AAATTTCAAGCC-AAATGCTCTTTACAAAGAAGATTTGAATTCGCAAAATAATT-GCTACTATTTTA
IES80            TTACTTTGATTTTAAATCTCAAATC-AACTGTGCTTTAAATAG-AGATTTGAATTTGCAAAATAATTAACTAATCT---A
CTG4797          --------------------------------------------------------------------------------
IES77b           TAGCATTAATTTAATATCTTAAATT-AAATTCTCTTTAGAAAATAGGTTTGATTTTGGAAAATAATTAACTACTCTTCTA
CTG826           TCGCTTTAATCTTAAATCTCAAACC-AAATTCTCTTTAGAAAGAAGTTTTGAATTTGTAAAATAATTAACTACTCTTCTA
IES132           --------------------------------------------------------------------------------
CTG7743          TTGCGTTATTTTTAAGTCTCTAACC-AAATGCTCTTTAGAAAGAAGATTTGAATTTGCAAAATAATTAACTTCTCTTCTA
CTG43276         --------------------------------------------------------------------------------
IES42b           --------------------------------------------------------------------------------
IES35            --------------------------------------------------------------------------------
IES104           --------------------------------------------------------------------------------
IES76            TTGTGTTAATTTTAAATCTCAAAC--AAATGCTGTGTAGAAAGAAGATTTGAATTTGCAAAATAAT---CTACCCATCT-
CTG13940         --------------------------------------------------------------------------------
IES42            TTGAGTTAATTTTAAATCACAAACCAAAATGCTCTTTAGAAAGAAGATTTGAATTTACAAAATAATTTACCACTCTTCAA
CTG34100         --------------------------------------------------------------------------------
IES169           TTGCATTAATTTTAAATCTCAAACA-AAATGTTCTTTAGAAAGAAGATTTGAATTTGCAAAATAGTTTAT--CTCTTCTA
IES103           TTGCGTTTATTTTAAATCTCAAACC-AAGCGCTCTTTAGAAAAGGGATTTGAATTTGCAAAATAATTTACTACTCTTCTA
CTG2630          --------------------------------------------------------------------------------
CTG45590         TTACGTTAATTTTTAATCTCAAGCCAAAATACTCTTTAGAAGGAAGATTTGAATTTACAAAATAATTTACTACTCTTCTA
AnchoisB    2610 TTGCTTTAATTTTAAATCTCAAACC-AAATGCTCTTTAGAAAGAAGATTTGAATTTGCAAAATAATTAACTACTCTTCTA
```

```
CTG36069         AGTTAAAATCAAAA-ATTACTTTAC---GATAAATATAACTTTGGGAGAAATTTAAA-----------TGCT--CAAAA-
IES85            AGTACAAATTAAAA-ATTAGTCTTCATTGATGAAGGTAGCCTTTGAAGAAACTTAAA-AACTGGAGGACATTGGC-AAAA
IES57            --------------------------------------------------------------------------------
IES214           AATTCAAATTATAA-ATTAATCTCTATTAAAGAATACAACTTTCGTAGAAACTTTAAGAGCTGGACGATGTTGCC--AAA
IES105           AGTTAAAATGATAA-ATTAGCCTACATTGATGAATGTAGC----------------------------------------
IES47            AGTTCATATTAAAA-ATTAGTCTGCATTGATGAATCTTGCTTT-GGAGAAACTTTAA-AGTTGGACGATGTTAGC-AAAG
IES80            AGTTAAAATTATAA-ATTAGTCTACATTGATGAATGTAAATTTTGGAGAAACTTACA-AGCAGGACGATGCTGGCAAAAA
CTG4797          --------------------------------------------------------------------------------
IES77b           AGTTTAAATTAAAA-ATTAGTCTACATTGATGAATTCAGCTTTTGGAGAAACTT-AA-AGCTGGATGATGCTTGC-AAAA
CTG826           ATTTCAAATTAAAA-TTTAGTCTACATTGATGAATCTAATTATTAGGAGAAACATAA-AGCTGGACGATG---GC-AAAA
IES132           --------------------------------------------------------------------------------
CTG7743          AGTTCAAATTTAAACATTAGTCTACATTGATGAATGCAGCTTTGGGAGAAACTTAAA-T-CTGGACGATGCTGGCAAAAA
CTG43276         --------------------------------------------------------------------------------
IES42b           --------------------------------------------------------------------------------
IES35            -------------------------------------AACTTTGGGAGAAACAATAA-AGCTGCACGATGCCAGCAAAAA
IES104           --------------------------------------------------------------------------------
IES76            ------------AA-ATTATTCTACATTGATGAATGTAAATTTGGGAGAAACTTAAA-AGCTGGATGATGCT-GCGAAAA
CTG13940         --------------------------------------------------------------------------------
IES42            AGTAAAAATTAAAA-ATTAGTCTATATTGATGGATGTAACTTTAGGAGAAACATAAA-TGCAAGACGATGCTGGCAAAAT
CTG34100         --------------------------------------------------------------------------------
IES169           AGTT-ACATTAAAA-ATTAGTCTATATTTATTAA--------TGGGAGAAACTTAAA-AGCTGGACGATGCTGGCAAAAA
IES103           AGTTAAAATT-AAA-ATTAGTCTACATTTATGAATTTAGCTTTGCGAGAATCTTAGA-AGCTGGACGATGCTGACAAAAA
CTG2630          --------------------------TTGATGAATTTAGCTTTGGGAGAAATTTAAA-AGCTG---------TGCAAAAT
CTG45590         AGTTAAAATT-AAA-ATTAGTCGATATTGATGAATGTAGCTTTGGGAGAAACTTAAA-AGCTGGGCAATGATGGCAAAAA
AnchoisB    2689 AGTTAAAATTAAAA-ATTAGTCTACATTGATGAATGTAGCTTTGGGAGAAACTTAAA-AGCTGGACGATGCTGGCAAAAA

CTG36069         --------------------------------------------------------------------------------
IES85            AACAATAGT--TATCCATTATTATAAATAGAATCG-AACTCCATAAAAA-GTAACTGTGTAAA---TGATGCTATAGGTT
IES57            --------------------------------------------------------------------------------
IES214           AAGAATATT--TATGCATCGCTGTAAATAAAATCG-AATTCTTCAAAA---TAAATGTGTAAT---TGGTGCCATCTTTT
IES105           --------------------------------------------------------------------------------
IES47            AAGAATAGT--TATCCATTACT-TTAACAAAATCG-AACTACTTAAAAA-ATAAATGTGTAAT---TAGTGCTATCGGTT
IES80            ATGAA-AGT--TATCTATTGCTGTAAGTAAAATCG-AGCTCATCAAAGA-GTAA--------------------------
CTG4797          --------------------------------------------------------------------------------
IES77b           AAGAATATT--TATCCAATGGTGTAAATAAAATCGGAA-TCCTC-AAAA-GTAAATGTGGGAT---TGGTGCCATCTTTT
CTG826           AAGAATATT--TATCCATTGCTATTAATAAAATCG-AACTCCT-AAAAA-GTAAATGTGTAATTGGTGGTGTTAACTGTT
IES132           --------------------------------------------------------------------------------
CTG7743          AAGAATATT--TAACAATTGCTGTAAATAAAATCG-AACTCCTTAAAAA-TTAAATGTAGAAT---TGGTTCAATCTTTT
CTG43276         --------------------------------------------------------------------------------
IES42b           -----TAGT--TACCCTCTACCGTAAATAAAATCG-AACTCTTCAAAAA-GTAAATGCGTATT---TGGTGCTATCTGTT
IES35            TAGAATGGT--TATCCATTGCTGTAAATAAAATCA-AACTCCTCAAAAAAGTATATATGCAAT---TGGTACCAAATGTT
IES104           --------------------------------------------------------------------------------
IES76            AAGAATAGT--TACCCATTTCTGTAAATAAATTCG-AACTCCTTAAAAA-GTAAATGCGTAAA---TGGTACCATCTGTT
CTG13940         --------------------------------------------------------------------------------
IES42            AA-AATAAT--TATCCATTGCTGTAAATAAAATCG-AACTCCTCAAAGA-GTAAATATGTATA---T-ATGATATCTGTT
CTG34100         --------------------------------------------------------------------------------
IES169           ATGAATAGTTATATCCATTGCTGTAAATAAAATCG-AACTCCTCAAAAT-TTATAGGTGTGAA---TGGAGCTAACTGTT
IES103           AATAATAGT--TATCAATTGCTGTAAATGAAGTCC---------------------------------------ATCTGTT
CTG2630          AAGAATAAT--TATCCATTGCTGTAAATAAAATCGAAACTCCTCGAAAA-GTAAATGTGTAAT---TGGTACTATCTGTT
CTG45590         AAGAATAGT--TATCTATTGCTGTAAAT-AAATCG-AACTCCTCAAAAT-GTAAAGGAGTAAA---TGGTAATATCCGTT
AnchoisB    2767 AAGAATAGT--TATCCATTGCTGTAAATAAAATCG-AACTCCTCAAAAA-GTAAATGTGTAAT---TGGTGCTATCTGTT

CTG36069         --------------------------------------------------------------------------------
IES85            CTTAC--TGGTTTATG-GCTTATAAATGCGTAATTGGAAATGTAAATCAATAGGTA-----TCTGGAGAACTTTCGCTTG
IES57            --------------------------------------------------------------------------------
IES214           GTGAT--AGGTTTATTTGAT-ATAAATGTGTTATCTGAAATGTAAAACAATAGGTA-----CTTTGGGAATTTTTGAATG
IES105           --------------------------------------------------------------------------------
IES47            TTGAT--GGAATTATT--TTTATAAATTTGTTATCGGAATTGTAAAACACTAGGTA-----TTT--AGAATTCTTGAATG
IES80            ------------------------AAGCGAGTTATCAGAAATGTAAATCAATTGGTA-----TTTAG-GAAATTTTGAATG
CTG4797          ------------------------------------------------------------------------TTTGAATG
IES77b           GTGAT--GGATTTTTT-TCTAATAAGTGTGTTATCGGAAATGTAAATTAATAGGTA-----TTTGGAAAATTTTTGAATG
CTG826           GTGAT--GGGTTTATT-GCTTATAAATGTGTTATAGGAAATGTAAATTAATCTGCATTTTTTTTGGAGAATTTTTGAATG
IES132           --------------------------------------------------------------------------------
CTG7743          GTGAT--TGGTTTATT-TCTTATCAATTTCTTTTCGGATACGTAAATTAATTGGGA-----GTTGGAGAA-TTTTGAATG
CTG43276         ----------------------------------CAGAAATTTAAAC---------------------------------
IES42b           GTGAT--GGATTTATT-GATTATAAATGTGTTATCGGAAATGTAAATCAATATA-A-----TTTGGAGAATTTTTGAATG
IES35            GTGAT--GGGTTTATT-GCTTATAAATATGTTATCAGAAATGTAAATCAATAGGTA-----TTTGGAGAATTTTTGAATA
IES104           -TGAT--GGATTTATT-GCTTAT-ATTGTGTCATCGGAAATGTAAATCTATAGGAA-----TTTGGAGAATTTTTGAATG
IES76            GCGAT--GGTTTTATT-GCTTATAAATATGTTATCGGAAATGTAAATCAATAGGTG-----TTTGGAGAATTTTTGAATG
CTG13940         --------------------------------------------------------------------------------
IES42            GTTATGGGGGTTAATT-ACTTATAAATGCTTTATCGGAAATGTAAATCAATAGGTA-----TTTGAAGAATTTTTGAATC
CTG34100         --------------------------------------------------------------------------------
IES169           GTGAT--GGGTT-ATT-GTTTATAAATGTGATTTCGGAAATGTAAATCAATGGGTA-----TTTAGAGAATTTTTGAATG
IES103           TTAAT--TGGTTTATT-GCTTATAAATATGTTATCTGAAATGTAAATCAATAGGTA-----TTTGGAGAATTTTTGAATG
CTG2630          GTGAA--GGGTTTATT-GCTTATAAATGTATCATCGGAAATGTAAATCAATAGGTA-----TTTGGAGAATTTTTGAATG
CTG45590         GTGAT--GGGTTAATT-ACTTATAAATGTGTTATCGGAAATGTAAATCAATAGGAA-----TTTGGAGAATTTTTGAATG
AnchoisB    2840 GTGAT--GGGTTTATT-GCTTATAAATGTGTTATCGGAAATGTAAATCAATAGGTA-----TTTGGAGAATTTTTGAATG
```

```
CTG36069          ------------------------------------------------------------------------TCCATCCA
IES85             AACAATT---AAGTTGCTTTGGCAA--AGTACT-GAAGATAA-TAAAATATAC-TTAGTGCTGGATAATACCTCCATCCA
IES57             --------------------------------------------------------------------------------
IES214            AACTTTT--AAAGCTGCTTTAGCAA--AGTAAT-GAAAAAAAATAACTTT-GC-TTAGTGCTGGATAATGCCTCCATCCA
IES105            --------------------------------------------------------------------------------
IES47             AATTTAT--AAAGCTGCTTTAGCAA--AATAAA-GAAGACAA-TAATTTT-GC-TTATCGCTTGATATTGCCTCCACCCA
IES80             AACTTTT--AAAGTTGCTTTTGCAA--AATAAA-GAAGAAAA-TAATTTT-------------------------GTTCA
CTG4797           AACTTT---AAAGCGGCTTTAGAA--------------------------------------------------------
IES77b            AACGTTT--AAAGCTGCTTTAGCAA--AGTAA--AAAAGAAA-TAATTTTT-C-TTAGTGCTGGATTGTGCCTCCATCCA
CTG826            AAATTTT--TAATCTG--TTAGCAA--AATAGCAGAACTAAA-TAATTTTTCC-TTGGTGCTGGATTATGC---------
IES132            --------------------------------------------------------------------------------
CTG7743           TTCTTTT--AAGGCTGC-TTAGCAA--AATAAT-GAAGGAAA-TAATTTTTGT-TTAATGCTGCATAATGCTTTCATCCA
CTG43276          --------------------------------------------------------------------------------
IES42b            AACTTTTTAAAAGTTTCTTTTGCAA--AACAAT-GAA-AAAA-TACTATATGC-TTAAT-CTGGA-AATTCCCCCATCCA
IES35             AACTTTA--AAAG-----GAAG--A--AATAAT-GTGGAAAA-TAATTTTTGC-TAAGCCCTGGATAATGCCTCCATCCA
IES104            AACTTTT--AAAGCTGAATTAGAAAATAATAAA-CAAAAAAA-TAATTTTTG--TTAGTGCTGGATAATAT-TACATCTA
IES76             AACTTTT--AAAGA-GCTTTAGCAA--AATAAT-GAAGAAAA-TAATTTT-GC-TTAG---AGGATAATGCCTTCATCCA
CTG13940          --------------------------------------------------------------------------------
IES42             AACTTTT--AAAGCTGCTTTAGCAA--AATAAT-AA--------AATTTTTAC-TAAGTGCTTGATAATTCCTCCATCCT
CTG34100          --------------------------------------------------------------------------------
IES169            AACTTTT--GAAGCTGCTTTTGCAA--AATAAT-GACGAAAA-CAATTTTTGC-TTAATGCAATGTAATGTCTCCATCCA
IES103            AACTTTT--ATAGCTGCTTTAGCAA--CATAAT-GAAGTAAA-TAATTTTTACTTTAGTGCTGGATAAT-TCTCCAT---
CTG2630           AACTTTA-------------AGCAA--AATAAT-GGAGAAAA-TAATTTTT-C-TTAGTGCTGGATAATGCCTCCATCCA
CTG45590          TAATTTC--AAAGCTGCTTTAGCAA--AATAAT-GAAGAAAA-TAATTTTTGC-TTAGTGCTGGATAATGCCTCCATCCG
AnchoisB    2912  AACTTTT--AAAGCTGCTTTAGCAA--AATAAT-GAAGAAAA-TAATTTTTGC-TTAGTGCTGGATAATGCCTCCATCCA

CTG36069          CTGGACATAT--AATTCTGAA-TCAAATATCATCTGTTAACTATTTATTATCTTCC-CAATAGAGCCA--TCAG-CTTAA
IES85             TTAGACACAATAAATTCTGAA-CCATTTATCACTTGCTTATCATTTATT-TTTTGC--------------TTAA-CTTAA
IES57             -------------------------------------------TTTGTT-TCTTCC------------------------
IES214            ATAGACTCAATAATTTCTTAT-TGAGTTATCATTTATTAATTATTTATT-T-TTCTTCTATATTGCCC--TTAGCCTTAA
IES105            ----------------------------------------------------------------------TTAA------
IES47             CTATACATA-TAAATTCTGAA-TTAATTATCATTTGTTAATTATTTA-T-TCTTCCTCCATATAACCC--TTAG-CTTAA
IES80             CCTAATATAATAAATTCTGAA-TCAATTATGATTTGTTAATTATTTATT-TCTTCCTCCAAATAGCCCTTTTAG-CTTAA
CTG4797           ----------------------TCATTTATCTCTTGTTAATCATTTAAT-TCAACATCCAAATAGCCT--TTAG-CTTAA
IES77b            CCGAACATAATAAATTAAAAA-TCAATTATCATTTGTTAGTTTTTTATT-TCTTCCTCTATGTAGCCC--TTAG-CTTAA
CTG826            ---AATATAAAATATTCTGAGTTCAATTATGATTTTTTAATTAGTTATT-TCTTCCTCCATCTAGCAC--TTTG-TTTAA
IES132            --------------------------------------------------------------------------------
CTG7743           CCATACATAATAAAATCTGAA-TCAATTATCATTTGTTAATTATTTATT-TCTTCCTCCATAGAGTCC--TTAA-GTTTA
CTG43276          --------------------------------------------------------------------------------
IES42b            CCGAACATAATA------------AATTATTATTTGTTAATTATATATT-TCTTCCTCCATATCACCC--TTAG-CTTAG
IES35             TCTAACGTAATAAATTCTGAA-TCAATTATCATGTGTTAATTATTTATT-TTTTCCTCCATATAGCCC--TTAG-TTTAA
IES104            CAGAACATATTAATTTCTGAA-TTATTTATCATTTGTTAAATATTTATT-TCTTCTTCCATATAGCCC--TTAG-CTTAA
IES76             CTGGAAATAATAAATTCTGAA-TCAATTATCATTTTTTAATTATTTATT-TCTTCCTCCATAGAGCCC--TTAA-CATAA
CTG13940          --------------------------------------------------------------------------------
IES42             TCAAACATAATA-GTTCTGAA-TCAATTATCATTTGTTAATTATTTATT-TCTTCCCCCATATAACCT--TTAA-CTTGA
CTG34100          --------------------------------------------------------------------------------
IES169            CCGAACATAATAAATTCTGAA-TCAATTATCATTTGGAAATTATTTATT-TCTACCTCCATATAGCCT--TTAG-TTTAA
IES103            ------ACAATACATTCTGAA-TCAATTATCATTTGTTAATTATGTATT-TCTTCCTCCAAATAGCCT--TTAG-CTTAA
CTG2630           TCGAACATAA----------------------------------------------------------------------
CTG45590          CAGATCGGAATAAATTCTGAA-TCAATTATCATTTCTTAATTATTTATT-CCTTCCTCCTTATAGCCC--TTAG-CTTAA
AnchoisB    2985  CCGAACATAATAAATTCTGAA-TCAATTATCATTTGTTAATTATTTATT-TCTTCCTCCATATAGCCC--TTAG-CTTAA

CTG36069          CTGCATTGTAAGGCTTTGGGGAATTGCAAAGTGATAGCTCTCTAATATGTATGTTGCAA-------AAGCTTCCTAA-GA
IES85             CTGCATCAAAAGGTTATTTGGAATTGC-AAGCAACAGCCTTCCAAAATACATCTTACAT-------CAATTTATTCA-GA
IES57             --------------------------------------------------------------------------------
IES214            TTGCATAGAAAGGCTGTGGGTAATTGCAAAGCGATAGCTCTCTAAAATGTATGTTGCAA-------CAAAGGCTTAACAA
IES105            -----------------GGAGAATTGCAAAGCGAAACTTCTTTAATACCCATATTGCAA-------AAACGCCATAA---
IES47             TTGAATCGAAATGCTGTGCGGAATTGCAAAGAGATAGCTTTCTAAAATACATATTGCAA-------CAACTGCC------
IES80             ATGCATAGAAATGATGTG-AGAATTGCTTAGCGATA--TCTCTAAAGAATCTATTGCAA-------CTACTGTCTAA-GA
CTG4797           CAGCATCGAAACGTTGTGTGGAATTACTCAGCGATAGCTCTCTAAAATGCTAGTTGCAA-------CATCTGGGTAA-TA
IES77b            TTGCATCGAAATTATATGGGTAATTGTTAAGCGATAGCTCTCTGAAATGCATATCGCAA-------CTACTACCTAA-GA
CTG826            CTGTATCGAAAAGGTGTGCGGAATTTCAAAGCCA-AGCTCTCTAAAATTCATGTTGCA--------CAACTGCCTAA-GA
IES132            --------------------------------------------------------------------------------
CTG7743           CTGCATAGGAAGGCTGCGGGGAATTGCAAAGCGATAGCTCTCTAAAATGAATGTTTTAA-------CAATTACCTAA-GA
CTG43276          --------------------------------------------------------------------------------
IES42b            CTGCATAGAAATACTGTGGGGAATTGTTAATTGATAGCTCTCTAAAATGCATATTTCAA-------GTACTGCCAAA-GA
IES35             TTGCATCGAATTACTTTGGAGAATTGCTA---------------------------------------------------
IES104            CTGCATCGAAATGTTTTGGGGAATTGCTAAGTGATAGCTC----------------------------------------
IES76             CTGCATCGAAATGCTCTGGATAATTGCTAGGCGATAGCTC--TAAAATGCAAATTGCAA-------CTACTGTCTAA-GA
CTG13940          --------------------------------------------------------------------------------
IES42             CTGCATCGAAATGCTGTGAGGAATTGCTAAGCGATAGCTCTCT-TAATGCATAGTGCAA-------CTACTACCAAA-GA
CTG34100          --------------------------------------------------------------------------------
IES169            CCGCATCGAAA-------------TGCTAAGCGATAGCTCTCTAAAATGCATAGTACAA-------CTATTACCTAA-GA
IES103            CTGCATCGAAATGCTGTGGGGAATTGCTAAGCGATAGCTCCTTAAAATGCATAGTTCAA-------CTACTACCTAA-GA
CTG2630           --------------------------------------------------------------------------------
CTG45590          CTGCATCGCAATGCTGTGGGGAATTGCTAAGCGATAGCTCTCTAAAATGCATAGTGAAACTACTATCTACTACCTAA-GA
AnchoisB    3060  CTGCATCGAAATGCTGTGGGGAATTGCTAAGCGATAGCTCTCTAAAATGCATATTGCAA-------CTACTGCCTAA-GA
```

```
CTG36069        ATTTGATAATATAC-GTGTTGTGCTAAACCCTATTACATCAACACAAGCTTA----AAA-CAAATGGATTTCAAACCTAA
IES85           ATTTTATAATACAC-ATGTCATGCTTAACTCTATTACATCAACTCTAGTTAAGTTCAAAACATCTGCAAATCAAACCTAA
IES57           --------------------------------------------------------------------------------
IES214          ATTTCATAACATAA-GTGTTGTACTTAACTATATAA--------------------------------------------
IES105          ATTTTATAATTTACAATGTTGTAAT-------------------------------------------------------
IES47           --------------------------------------------------------------------------------
IES80           ATTTTATAATTTAA-ATGTTGAGCATAACTCTATTACAACAACTCAAGTTCA----AAT-CATCTGCAATTCAAACCTAA
CTG4797         ATTTTAT-ATATAC-ATATTGTGCTTAATTCTATTAAATCAACTTCAGTTTA----AAA-CATCTGCAATTCAAA-CTAA
IES77b          ATTTTATAATATAT-ATGTTGCGCTTAACTCTATTACAACAACTCGAATTCA----AAA-CATTTGCACTTCAAACCTAA
CTG826          ATTTCATAACATAC-GTGTTGTGCTTAACTCTATTACTTCCAATCAAGCTCA----GAA-AATCTGCACTTAAAATCTAA
IES132          --------------------------------------------------------------------------------
CTG7743         ATTTCATAATATTC-ATTTTGTGCTTTACTCTATTACATCAACCCAAGTTCA----AA----------------------
CTG43276        --------------------------------------------------------------------------------
IES42b          -TTTTATAATATAC-ATTTTGCTCTTAACTCTATTATAATAAAACAAGTTCA----AAA--ATCTAAACTTCAAACCTTA
IES35           --------------------------------------------------------------------------------
IES104          ----------------------------------------------------------------------------CTAA
IES76           ATTTTATACTAAAC-AAGTTGCGCTTAATTCTATTACAATAATTCAAGTTGA----AAA-CATCTGTACTTCACCCCAAA
CTG13940        -----ATAATATAC-ATGTTGTGCTTAATTCTATTAAAAAAACTCAAGTTCA----AA--CATCTGCACTTTTAACCCAA
IES42           AGTTTA-AATACAC-ATGTTGTGCTTAACTCTATTACAATAACTCAAGTTAA----AAA-CATCTGCAAATCAAACTTAA
CTG34100        --------------------------------------------------------------------------------
IES169          ATTTTATAATATAC-ATGTTGTGCTTAACTCTATTACAACAACTCAAGCTCA----AAACCATCTCTACTTCAAATTTAA
IES103          ATTTTATAATATAC-ATGTTGTGCTTAACTCTATTACAACAGCTCAAGTTCA----AAA-AATCTGCACTTCAAACTTAA
CTG2630         --------------------------------------------------------------------------------
CTG45590        GAATTAT-ATATAC-ATATTGTGCTTAACTCTATTACAACAACTCAAGTTC-----AAA-CATCTGCACATCAAACTTAA
AnchoisB   3132 ATTTTATAATATAC-ATGTTGTGCTTAACTCTATTACAACAACTCAAGTTCA----AAA-CATCTGCACTTCAAACCTAA

CTG36069        AATTCCTAGAAGTTTGCTTGTTACAAAAATAATTCTGATGGTA-TAA--------------------------TTTCATA
IES85           A-TTCCTAGAAACTTTCCTGTTACAAAAATATTTCTGATG-AA-TTATATACAGTGCTAATTTTAAAATTAAGTATCACA
IES57           --------------------------------------------------------------------------------
IES214          --------------------------------------------------------------------------------
IES105          -------------------GTTG-GACAATAATTCTGATGCTA-ATATATACAATTCAAATTTTAATATCAAATTTCACA
IES47           --------------------------------------------------------------------------------
IES80           AATTCTTAAAAGCTTGCTTATTAC-AAAATAATTCTGATGCTA-TTATATACAATTTAAATTTTAATATATAGTTTCAAT
CTG4797         AATTCATAAAAGCATGCTTGTTACGAAAATAATTTTGATGCTA-TTGTATACAATTCAAATTTTAATCTCAAATTTCACA
IES77b          AATT---AGA---TTGATTGTTAAGAAAATAATTCTGATGCTA-TTATATACAATTTAAATTTTAATACAAAATTT-AAT
CTG826          AATTCC-AAAAACTTACTTATTAA-AAAATGATTTTGAAACTA-TTAGATACTAGTCGAATTTTAATATCAAGTTTCAGA
IES132          --------------------------------------------------------------------------------
CTG7743         ----------------CATATTACGAAAATAATTCTGATGTTA-TTAT--------------------------------
CTG43276        -------------------------------------------------------------TTTAGTATAAAGTTTTAAT
IES42b          AATTCCTAGATGATAGCTTATTACGAAAATAATTCTGATACTA-TTA---------------------------------
IES35           --------------------------------------------------------------------------------
IES104          AATTCCTAGA----------------------------------------------------------------------
IES76           AACACCTGGAAGTTTTCTTGTTAAGAAAATAATTCTGATGCTATTTATATACAATTCAAATTTATTATAAAGTTTCACA
CTG13940        A-TTCCTAGATGCTTGCTTGTTATGAAAATAATTTTGATACTA-TTATATACAATTCAAATTTTAATATAAAGTTTCACA
IES42           AATTCCTAGAAGTTGGCTTTTTACGAAAATAATTCTGATGCTA-TTATATACAATTTAAATTATAATGTAAAGTTTCAAT
CTG34100        --------------------------------------------------------------------------------
IES169          AATTCCTAGAAGCTTGCTTGTTAC-AAAATAATTCTAATGCTA-TTTTATACAATTTAAATTTTAATATAAAGTTTCAAT
IES103          AATTCCTAGAAGCTTACTT-TTAAGAAAATAATTCGGATGCTT-TCATACAAAATTTAAATTTTAATATTATGTTTCAAT
CTG2630         ------------------------------AACTCTGATACTA-TTATATAGAATTTAAATT-TAATATAAA-TTTCAAT
CTG45590        ACTTCCTGGAAGCTTGCTTGTTTCGAAAATAATTCTGATACTA-TTATATACAATTTAAATTTGATATAAAGTTTCAAT
AnchoisB   3206 AATTCCTAGAAGCTTGCTTGTTACGAAAATAATTCTGATGCTA-TTATATACAATTTAAATTTTAATATAAAGTTTCAAT

CTG36069        AGTAAATGAGCAAA-AGGTGTCCGTTTATTTCTAACAGTGATGGTGTAGCAATTACATATTGTTAGTCTGTAAAGTTTA
IES85           AGT-AATGAGCAAA-AGGTCTCTGATTCTTTCTAATAGTGATGGTGTAACAACTGCAGATTGGTATTCTGGAATGCTTAA
IES57           --------------------------------------------------------------------------------
IES214          --------------------------------------------------------------------------------
IES105          AGTAATTGAGCAAA-AAGTCTCC-----TCTCGAATAGCTATTTTATAGAATCTGCAGATTGTTAGTCTGGAAAACTCTT
IES47           --------------------------------------------------------------------------------
IES80           AGTAAATTAGTAAA-AGTTCTCCGATTCTTTTGAAATGAGATGGTGTAGCAACTACAAATTGTTAGTCTGGAATGCTTTA
CTG4797         AATAAATAATCGAA-ATTTCTCCGATTCTTTCGAATAGGGATGTTGTAGCCACTGCAGATTCTTATTCTGGAATGCCTAA
IES77b          AATAAACGAACAAA-AATTCTCCCATTTTTTCGAATGGCT------------TAGCAGATTGTTAGTCTGGAATGCTTTA
CTG826          AATTAATAAACAAA-AGGTCTCCTCTCTTTTCGAATAGCTATCCTGTAGTAACTGTAAATTGTTAGTCAGGAATGCTTTA
IES132          --------------------------------------------------------------------------------
CTG7743         --------------------------------------------------------------------------------
CTG43276        AGTAAATGAGCAAA-AAGTCTCCGATTCTTTCGAATAGC-ATGGTGTAGCAACTGCAGCTCGTTATTCTAGAATGCTTTA
IES42b          -GTAAATGAGCAAA-ACGTTTCCGATTCTTTC-----------------CACCTGCAGTTTGTTAGTCTGGAACGCTTTA
IES35           --------------------------------------------------------------------------------
IES104          --------------------------------------------------------------------------------
IES76           AGTAAATGAGCAAA-ATGCCTCCGATTCTTTCGAATAGCGATGCTGTAGCAACTTTAGATTGTTAATCTAGAATGCTTTA
CTG13940        ACTAAATAAGCAA--AAGCCTCCTATTTTTTCGAATAGCCATGGTGTAGCAACTACAAATTGCTAGTATGGTATTCTTTA
IES42           TTT-AATTAGCAAA-ATGTCTCAGATTCTTTCGAATAGCGATGGCGTAGCAACTGCAGATTGTTAGTCTGGAATCCTTCA
CTG34100        --------------------------------------------------------------------------------
IES169          TGTAAATGAGCAAATAAATCTCCAATTCTTTCTAATATTGAGTGTGTAACAACTGCAGATTGTAAGTCTGGAATGCTTTA
IES103          AGTAAATAAGCAAA-AAGCCTCCGATTCTTCCTAATAGCGATGGCGTAGAAACTGCAGATTGTTAGTCTGGAATGCTTTA
CTG2630         AGTAAATGAGCAAT-AAGTCTCCTAATCTTTTGAATAGTGATGAAGTATCAACTGCAAATTGTTAGTCTGGAATGCTTTA
CTG45590        AGTAAATGAGCAAA-AGGTCTCCGATTCTTTCGAATAGTGATGGTGTAGCAACT--------------------------
AnchoisB   3285 AGTAAATGAGCAAA-AAGTCTCCGATTCTTTCGAATAGCGATGGTGTAGCAACTGCAGATTGTTAGTCTGGAATGCTTTA
```

```
CTG36069         ACAC---CAAGCAGAATTTATTCAAAGCAGAGCTTCAAATTGGG-GACCGAAGGAGAGCTAAATCACTTACTCAAACATC
IES85            ACACTTTCAAGCAAATTTTAGTCGAAGCAGAGTTTCAAATTTGGGGATAGAAAGGGAGTTGAATCACTCACTCCTTAATC
IES57            --------------------------------------------------------------------------------
IES214           ---CTTTCAAACAGATCTTA-TCGAAGAAGAGTTTTGAATTGGGAAACAGAAAGTTAGGAAAATCACTCTATCGTTAATC
IES105           ACACTTTTCA---------------AGCATACTTTCTAATTGGGGGATTGAAAGGTC-----------------------
IES47            --------------------------------------------------------------------------------
IES80            ACACTTTCAAGCAGA----------------GTTTCGAATTGGGGAGCGGAAAGGGGGCTAAATCATTCTCTCCTTAATC
CTG4797          ACGCTTTCAAGCAGATTATAATCGAGGCAGAGTTTCGA---TAGGGACCAAAAGGGAACTAAATCATTCGCTCCTTAATC
IES77b           ACAATTTCAAGCAGATTTTAGTCAAAGCAAAGTTTTGAATTCGTGGACCGAAAGAGAGCTAAATCGCTCACTCCTTAATT
CTG826           AAAC-TTCAAGCAGATTTTAGTCTAAGAAAGGTTTCTAACTGGAGGACTGAAAAAGAACTA-------------------
IES132           --------------------------------------------------------------------------------
CTG7743          --------------------------------------------------------------------------------
CTG43276         ACACTTTCAAGCAGA----------------GTTTCGAATTGGG-GACCTAAAGGGAGCTAAATCACTCACTCCTTAATC
IES42b           ACACTTTCAAGCAGATTTTAGTCGAAGCAGAGTTTCCAATTGAGGGAC----------CTAAATCACTCGCTCCTTAATC
IES35            ---------------------------------------------GACCAAAAGGAAACTA-------------------
IES104           ------------AGATTTTATTCGAAGCAGAGTTTCGAATCGGGGCACAGAAAGGA--CTAAATCACTCGCTCCTTAATT
IES76            ACACTTTTAAGCAGAT-TTAGACGAAGCAGAGTTTCGAATTGGACGGCCAAAAGGGAG-TAAATTGCTCACT-TTTGATA
CTG13940         ATATTTTCAAACAGATTTTAGTCGAAGTAGAGTTTCGAATTGGGGAATCGAAAGGGAACTAAATCACTCG---------C
IES42            ACAATTTCAAGCAGATTTTAGTCGAAGCAGAGTTTCGAA-TAGGGGACAGAAAGGGAGCTGTTTCCCT--GTCCTCATTC
CTG34100         --------------------------------------------------------------------------------
IES169           ACACTTTCAAGCGGATTTTAGTTGAAGCAGAGTTTCGAATTGGGGGACCGAAAG-GAGCTAAATCAGTCGCTCTTTAATC
IES103           ACCCATTCAAGTAGAT----------------TTTCGAATTGGAGGACCTAAAGGGAGCTAAATCACTCGTTCATTAATC
CTG2630          ACGCTTTCAAGCAGATTTTAGTCA---TAGAGTTTCGAATTGGGGAACAGAGAGTAAGCTAATTCACTCACTCCATAATC
CTG45590         ----------GCAGATTTTAGTCGAAGCAGAGTTTCGAATTGGGGGACCGAAAGGGAGCTAAATCACTCGCTACTTAATC
AnchoisB    3364 ACACTTTCAAGCAGATTTTAGTCGAAGCAGAGTTTCGAATTGGGGGACCGAAAGGGAGCTAAATCACTCGCTCCTTAATC

CTG36069         TATCCAACC---AACTCTCAATATAACA-AGCCTTTCATTTTTATTTATAG-TTTATT--TTATAAAAAATAACATTATG
IES85            TACTGAGCCA--GCCACTTAGTATAAAACAGTCTTTCACCTTTATTTAT-G-TTTTTT---------AA-TAAAAATATG
IES57            --------------------------------------------------------------------------------
IES214           --------------------------------------------------------------------------------
IES105           ------------AAATCTCACTAGAATA-AGCCTTTCATTTTTA------G-TTTTTCTATTGTAAAAA---AAATTATG
IES47            --------------------------------------------------------------------------------
IES80            TATCCAGTC---AACTCTCAATATAACT-AGCCTTTGATTTTTATTTATTG-TTT-------------------------
CTG4797          AATCCAGCC---AACTCTCAATATCACC-AGTCCTAAATTGTTATTAATAG--TGTTTTATTATAACAA-TAAAATTATG
IES77b           TCACCAGCC---AACTCTCAGTATTACA-AGCCGTCCATTTTAATTTATCGTTTTTTGATTAT-AAAA-TAAAATGATG
CTG826           ------------ATCGTTCAATATAATA-AGCCTTTCATTTAT-----------------TTACAAAT----AAATTTTG
IES132           --------------------------------------------------------------------------------
CTG7743          --------------------------------------------------------------------------------
CTG43276         TATCCAGCT---AAATCTCAAGATAACA-AGCCTTTCATTTTTATTTATAG--TTTTTGATTATAAAAA-TAAAATTATG
IES42b           TATCGAGCC---AACTCTCAATATAACA-CTTATTTCATTTTTATTTATAG-TTTTTTGATTATAAAAA-TAAAATTATG
IES35            ---------------------TATAACA-AGCCTC-----------------CGTTTTATTTATAAAAA-GAAAATTATG
IES104           CATTCAGCCAAAAACTCCAAATATGACA-AGCCTTTCATTTTTATTTATAGTTTTTTTGATTATAAAAA-TAAAATTATG
IES76            TATCCAGCA---AACTCTCAATATAA-A-AACCTCTTATTTTTACTTATA--TTCTTTGATTATAAAAA---ACATTATG
CTG13940         TATCCAGTC---AACTTTCAATATAAAA-TGGCTGTCATTTTTATTTTTAA--TTTTTGATTATAAAAA-TAAAATTATG
IES42            TATCCAGCC---AACTCTCAATATCATA-AGCCTTTCATTTTTAATTATAG-TGTTTTGATTATAAAAT-AAATTTTAAG
CTG34100         --------------------------------------------------------------------------------
IES169           TATCCAGCC---AACTCTCAATATAACA-AGCCTTTCATTTTTATTTATAG-TTTTTTGATTAT-AAAA-TAAAATTATG
IES103           TATCCAGC----------CAATTTAACA-AGCC-TTCATTTTTTTTTATAG-ATTTTTTATTATAAAAA-TAAAATTATG
CTG2630          TATCCAGCA---AAATCTCAATAGAACA-AGCCTTTCATTTCTATTTCTAG-TTTTTTGATTATAAAAA-TAAAATTATG
CTG45590         TATCCAGCA---AACTCTCAATATAACA-AGCCTTTCATTTTTA-TTATAG-TTTTTTGATTATAAAAA-T-AAATTATT
AnchoisB    3444 TATCCAGCC---AACTCTCAATATAACA-AGCCTTTCATTTTTATTTATAG-TTTTTTGATTATAAAAA-TAAAATTATG

CTG36069         GGTTTGTGTAATTCATTATAGATTATATGTAAAATTAGATTAAT-CTT------ATAATAAAAATAATATCGAGAAGTCT
IES85            GGTTCGTGTGATTAATGATTGATTATAAATAAAAATAGATTTATGATTTATGTCACGATAAAAATAATATAATAAAGTCT
IES57            ---------------------------------AATCGATATA----------------------------------TCT
IES214           -----------------------TAGATATAAAAATAGATTTATAATT------ATTATAAAAATATTATCAGGCAGTCT
IES105           AGTTTTTGTTACACTTGAGTAATAATATATAAAAGTAGATGTAG-TTA------ATAATAAAAATAATAACAAGTAGTTT
IES47            --TTTGTGTGATTCTTTTGTGATAATATATAAAAATAGATTTAT-ATT------ATAATAAAAATAATATCAGGAAGTCT
IES80            GGTTTGTTTGATTCGTGATTGATTATAT---AAAATAGATTTA-----------------GAAACA----CAGGAAGTTT
CTG4797          AGTTTGTGTGATTCATGAGTGATTATAAATAAACATAGATTTAT-ATT------ATAAAAAAAATAATATCAGGAGTTCG
IES77b           GGTTTTTGTGATCTATGAGTGTTTTTATAT-AAAATAGATTTAT-CTAT-----TCTAAAGAAATAATATC--TAAGTTT
CTG826           GG--------------GAGTAATTATTTATAAATATAGATTTAT-ATT------ATAAA--AATTAATACAAAGAAGTCT
IES132           --------------------------------------------------------------------------------
CTG7743          --------------------------------------------------------------------------------
CTG43276         GGTTTGTGTGATTAATGACTGATTAAATATAAAAATCGATTTAT-ATT------ATGAAAGAAATAATATTAAGAAGTCT
IES42b           GGTTTGTGCGATTCATGAGTGATTATATATAGAATAGAATTTAT-TTT------ATAAAAGAAATAAATTCAGGATTTTT
IES35            GGTTTATGTGATACATGAGTGATTATATTTAAAAATAGATTTTT-ATT------ATAATAAAAATAATATCAGGAAGTCT
IES104           GGTTTGTGTGATATATGAGTGATTTTCGATAGAAATAGATTTAT-ATA------ATTAAAGAAATATTATCAGGAAGTTT
IES76            GGTTTGTATGATTCATGAATGATTATATAT-GAAATAGATTTAT-ATT------CTAAAA-AAATAATATCAGGAAATCT
CTG13940         GGTTTATGTGATTCAAGAGTGATTATATATAAAAATCAATTTAT-ATT------ATAAAAGAAATAATATCAAGAAGTCT
IES42            GGTTTGTGCGATTCATGAATGAGTATATATAAAAATCGATTTAT-ATT------ATA-ATGAAATAATATCAGGAAGTCT
CTG34100         --------------------------------------------------------------------------------
IES169           GGTTTAAGTGATTCATGAGTGACTATTTATAAAAATCGATTTAT-ATT------ATATAAGAAATAATATTAAGATG---
IES103           AGTTTATATGATTCATGAGTTAGTATTTATAGAAATCGATTTAT-ATT------ATAAAAGAAATAATATCAGGAAATCT
CTG2630          GGTTTGTGTGATT-ATGAGTGATTATATATAATAATCGATTTAT-ATT------ATAAAAGAAATAATATCAGGATATCT
CTG45590         GGTTTGTGTGATTCTTAAGTGATTATATAT-AAAATCGATTTAC-ATT------TTAAAAGAAATAATATCAGGATGCCT
AnchoisB    3518 GGTTTGTGTGATTCATGAGTGATTATATATAAAAATAGATTTAT-ATT------ATAAAAGAAATAATATCAGGAAGTCT
```

```
CTG36069        GAAGAAGTATATTAAATCGTATTTGACTATA
IES85           GAAGGAGTAGATGAAATTGTATTGGACGATA
IES57           AATTAAGGAGTCAAACTTTAATTGA------
IES214          GAAGAAGT----GCCATTGTTTTGAACTATA
IES105          GAAAAAGTGTTTGAAATTTTATTGGACTATA
IES47           GAAGTAGTAGATAAAATTGAATTCAACTATA
IES80           GAAGAAGTAGATGAGATTGTTTTGGACTATA
CTG4797         GAAATAGTAGATGAAATTGCATTGGACTATA
IES77b          GAAAAAGTAGATGAAATTTAATTGGTCTATA
CTG826          TAAGAGGTAGATGAAATT-TATTGTACTATA
IES132          -------TAGATGAAATTGGATTGGACTATA
CTG7743         -------------------------------
CTG43276        ---GAAGTAGAT-AAACTATATTGGACTATA
IES42b          GAAGAAGTAGATGAAATTTTATTGGACTATA
IES35           GAAGAAATAGATGAAATTGTATTGGACTATA
IES104          GA-GAAGTAGATGAAATTTTATTAGACTATA
IES76           GAAGAAGTAGATCAAATTTTATTGGACTATA
CTG13940        AGAGAAGTAGATAAAATTGTATTGGACTATA
IES42           AAAGAAGTAGATAAAATTGTATTGGGCTATA
CTG34100        --AAAAGT-----------------------
IES169          GATGAAGTAGCTAAAACTGTATTTGACTATA
IES103          GAAGAGGTAGATAAAACTGTATTGGACTATA
CTG2630         GAAGAAGTAGATAAAATTGTATTGGACTATA
CTG45590        GAAGAAGTAGATAAAATTGTATTGGACTATA
AnchoisB   3591 GAAGAAGTAGATGAAATTGTATTGGACTATA
```

E

IES (ParameciumDB Accession)	IES Size (nt)	Element	Alignment Label	Alignment Length (nt)
IESPGM.PTET51.1.98.309432	2003	A	IES98	1226
IESPGM.PTET51.1.128.254421	3392	A	IES128	3394
IESPGM.PTET51.1.77.209216	4154	A	IES77	3258
IESPGM.PTET51.1.103.177611	2462	B	IES103	2464
IESPGM.PTET51.1.104.49056	2238	B	IES104	2240
IESPGM.PTET51.1.105.239361	2483	B	IES105	2295
IESPGM.PTET51.1.132.167159	1500	B	IES132	1502
IESPGM.PTET51.1.169.56908	3272	B	IES169	3274
IESPGM.PTET51.1.214.11549	2317	B	IES214	2319
IESPGM.PTET51.1.35.111752	2820	B	IES35	2766
IESPGM.PTET51.1.42.397702	2473	B	IES42b	2262
IESPGM.PTET51.1.42.72890	3389	B	IES42	3391
IESPGM.PTET51.1.47.408041	2125	B	IES47	2127
IESPGM.PTET51.1.57.48117	1257	B	IES57	1099
IESPGM.PTET51.1.76.220822	3048	B	IES76	2890
IESPGM.PTET51.1.77.311405	3470	B	IES77b	2755
IESPGM.PTET51.1.80.84925	1513	B	IES80	1515
IESPGM.PTET51.1.85.45587	3479	B	IES85	2959

IESs used to build the final *AnchoisA and AnchoisB* consensus sequences.
