# Supplementary material for: The Paramecium Germline Genome Provides a Niche for Intragenic Parasitic DNA: Evolutionary Dynamics of Internal Eliminated Sequences
Source: PLoS Genet. 2012 Oct 4;8(10):e1002984. doi: 10.1371/journal.pgen.1002984 (PMC3464196; doi:10.1371/journal.pgen.1002984)
Supplement: Text S2 — Alignment of homologous IESs inserted at non-homologous genomic sites. The IESs of each cluster of homologous IESs (cf. Table 3 and its legend) and 200 bp of 3′ and 5′ flanking sequences were aligned using Muscle [89]. The IESs are in uppercase type and the flanking sequences are in lowercase type. For cluster5, consisting of IESs homologous to a solo TIR of the Thon transposon, the consensus sequence and the Thon TIR are included in the alignment and the palindromic repeats are highlighted. (PDF) [file pgen.1002984.s014.pdf]

CLUSTER 2
MUSCLE (3.7) multiple sequence alignment

```
IESPGM.PTET51.1.25.381101      agtaagagtagatatactgatcttttgttatt---ataaatgtattagtttctcatttaa
IESPGM.PTET51.1.25.389332      ----------catgt-ttgatatattattactcaaccaaatgctcctaatatgcattctt
                                          ** *  **** * ** *** *     *****       *   ****

IESPGM.PTET51.1.25.381101      ttaattttctgtatcatctttctcgattttaaatatagcat--------aatatggcgat
IESPGM.PTET51.1.25.389332      taaaatataagtaggagatttcataatattatttatattatttataatcaatat-acaat
                               * ** * *  ***  *  ****   ** ***  ****  **        *****  * **

IESPGM.PTET51.1.25.381101      ctaaactat----atctcttattgaagttcataaatagtaaa---attcaaatt------
IESPGM.PTET51.1.25.389332      ctaattaattttgaatttttatttgatatcttgaatcatagggttattcaaaataatata
                               ****   **    *  * *****  *  ** * ***  **     ******* *

IESPGM.PTET51.1.25.381101      ---attaattcttctttcctgtgacttttcaaaattttcttattcatTATAGTGCTCACA
IESPGM.PTET51.1.25.389332      cgaattacttctcttttgctgtaac------agattt---------aTATAGTGTTCACA
                                  **** ****  *** **** **      * ****          ******* *****

IESPGM.PTET51.1.25.381101      TAATACATTTCGATGTTAAGAGTATATAT-AAGAGAAGGATTTAAATATATAAAGTAAAA
IESPGM.PTET51.1.25.389332      TAATAGATTTTGTAATTAAGGATATATATAAAGTGAAAGATTTAAATAGATTGAGTTAAA
                               ***** **** *   *****  ******* *** *** ********** **  *** ***

IESPGM.PTET51.1.25.381101      ATGTTTAA---------------TTAAAATATGAAATCA-TAAAAAATTAAATGTTATTA
IESPGM.PTET51.1.25.389332      ATATTTGAAATCAGAAACTATACTTAAAATGTGAAAGCATTAAATACTTAAATTTTATAA
                               ** *** *               ******* ***** ** **** * ****** **** *

IESPGM.PTET51.1.25.381101      TTAATAATACCATTCATTAATTAATGTTCATTCCTTCTATAAATTTATGTATTTCATATA
IESPGM.PTET51.1.25.389332      CTAA-------------TAATTAATCTTCATTCCTTCTATAAATTTATATATTTCATATG
                                ***             ******** ********************** **********

IESPGM.PTET51.1.25.381101      TCCCTTCACATAAAAGATTTTATTTAATAAGTAtat-tagttgtttaacatatttacttt
IESPGM.PTET51.1.25.389332      TCCCTTCACATAAATGCTTTTATTTAACAACTAtatatatattttgaatacgttaaatat
                               ************** * ********** ** ***** **  * ** ** *  ** * * *

IESPGM.PTET51.1.25.381101      tttttctctatttattatctattcacaaattttctattctcagaataggtatttgcatca
IESPGM.PTET51.1.25.389332      gttaacatcatcca--agctattcagcagtcaatcgttattagaa--------tgtatca
                                **  *   **  *  * *******  * *      ** * ****        ** ****

IESPGM.PTET51.1.25.381101      ggatagttgc-ataaattttgaaaattgtttgctgtctccctatcattttattcaagttt
IESPGM.PTET51.1.25.389332      ----aattgcaattaatatttagaa--------tgtcttatggatatttcttccaaaatt
                                   * **** ** *** ** * **        *****       ****  * ***  **

IESPGM.PTET51.1.25.381101      ttaaatgaaatcatcttttaattttaattg-------atcatcattatccttagc--aat
IESPGM.PTET51.1.25.389332      ttacttg-actctactattgaatcaaatagggattcaaaaaacatcatcttcagcagaat
                               ***  ** * **  ** ** * *  *** *       *  * *** *** * ***  ***

IESPGM.PTET51.1.25.381101      aa------------ttag
IESPGM.PTET51.1.25.389332      aaaggagatcactcttga
                               **            **
```

TextS2 - Alignment of homologous IESs found at non-homologus sites - page 2

CLUSTER 3
MUSCLE (3.7) multiple sequence alignment

```
IESPGM.PTET51.1.160.10020      -----tctaattg---------------taagaaagagtgtatacctaaatgtatttctt
IESPGM.PTET51.1.117.944        -----ttttaatacctcagcaagttaattaagaaagagttg----ttaa--------tct
IESPGM.PTET51.1.44.7711        agacgatttagtg----------aaaaagaaaaaaaaactaaaatttaa--------ctt
                                      * * *                 ** *** *         ***          *

IESPGM.PTET51.1.160.10020      caa---------tcatactgaattcttataaattgtcaattaatttgatttatttgatta
IESPGM.PTET51.1.117.944        gaaagcgcatcgtagtagtgaaatttca----------------ttaaga-----gcgca
IESPGM.PTET51.1.44.7711        caaaggat----taatagttagtgttta----------------ctaaaatgtatgatca
                                **         *  ** * *    * *                 * *       *   *

IESPGM.PTET51.1.160.10020      aaatattaataatgtaataattagttttttgattgatttctattagatgaggggagaatg
IESPGM.PTET51.1.117.944        acataataata---------------------------tatattattc---aagtgattg
IESPGM.PTET51.1.44.7711        aaatgttact----------------------------ttcattacttaggaagaaaatt
                               * **  ** *                            *  ****        *  * *

IESPGM.PTET51.1.160.10020      ctcttttagatattattggaagctatatgccttaatat--aaaataaaaaa---------
IESPGM.PTET51.1.117.944        t--------aaatcgctgaacatgacaa--ttctatactaaaaatcgaaaatttaa----
IESPGM.PTET51.1.44.7711        c--------atacctttgggtagtataaactttgaaatgggaaatcgaaaatctaaagta
                                        * *    **      * *  *    * *    ****  ****

IESPGM.PTET51.1.160.10020      --------------------------------------------TATAGTCGATAGTAGA
IESPGM.PTET51.1.117.944        --ccctaatttgattgtgtcatcttcttattgaattaattctcaTATAGCTGATATCAGA
IESPGM.PTET51.1.44.7711        ttccataaattgtttata--attatctccatgaatt--------TATAGCTGATATTAGA
                                                                           *****  ****  ***

IESPGM.PTET51.1.160.10020      AATTATTTTTAAATAAATA-AAAATATATCATTATAATTTATAGGTTTTGAAGGCAAAAA
IESPGM.PTET51.1.117.944        AATTCTTTTAA---AGATATAATATATATTGATATAATTTATCAGTTTGGGAG---ATAA
IESPGM.PTET51.1.44.7711        AATTATTATTA---AAATATAATATATAATAATATAATTTATCAGTTTGGGAG---ATAA
                               **** ** * *   * *** ** *****    **********  **** * **   * **

IESPGM.PTET51.1.160.10020      AATAGGTCCAATTTATATCAAGTGAATTT---ATCT------TTAATTTTTACTTATATG
IESPGM.PTET51.1.117.944        AATAAGCCCATTTTATCTCCCGTGAATATCCAGTTTAGGAAATTAACCCAAATATGCATT
IESPGM.PTET51.1.44.7711        AAT------ATTTTATCTCCCGTGAATTTCCAGTTTAGGAAGTTAACTTAAATATATATT
                               ***      * ***** **  ****** *    * *      ****     *  *  **

IESPGM.PTET51.1.160.10020      AGTGAATTTCACTTATTGGCAATTCACCATTTTATATCTTGCAAGTAGAGTAAAGAATAA
IESPGM.PTET51.1.117.944        AGTGAATTTCCTTTATTGGCAATTCACCATTTCATATTTTGGAAG----ACAAGGAGTAA
IESPGM.PTET51.1.44.7711        GGTGAATTTCCTTTATTGACAATTCACCATTTCATATTTTGGAAA----ACAAAGAATAA
                                *********  ****** ************* **** *** **       ** ** ***

IESPGM.PTET51.1.160.10020      TTTTTTGTGATT--ATTATGAAATT--CAATATATTTAT-AATCTCCTAACATAAAAATT
IESPGM.PTET51.1.117.944        TTTTTTGTAATTTAATTATAAAATGTCAAATATATTGGTGAATTTCCAAATTTAGAAATT
IESPGM.PTET51.1.44.7711        TTTTTTGTAATTT--------------AAATATATTGGTGAATTTCCTAATTTAGAAATT
                               ******** ***                ********  * *** *** **  ** *****

IESPGM.PTET51.1.160.10020      CACGAAACATCTATGTCATTTTTGATATAAGCT--AAATAAAGTTAAAAAC---------
IESPGM.PTET51.1.117.944        CACCGAAAACAC-TTTTTTTTGTAAACTTTATTAATATGCAGGATAAGAACACAACATAT
IESPGM.PTET51.1.44.7711        CACCAAATATATATATAATCCCTGATTTTTACT--TAAGAATGATAAAAAC---------
                               ***  ** *    * *  *   * *  *    *   *   * * *** ***

IESPGM.PTET51.1.160.10020      ----------TTTTATTTAACTTTAAGAAATCATCTTATTTTTTGAAGAGTACTTTTCGG
IESPGM.PTET51.1.117.944        ATTATGATGTCTTCAAAGGTTATTAAGTAAATATCAGAGATGTTATATATATTAGGTGAA
IESPGM.PTET51.1.44.7711        ----------CTTTAGATGCCTTGAAGACATCATAATATATGTTATACAG----------
                                          ** *       * ***  *  **   *  * **  * *

IESPGM.PTET51.1.160.10020      TCTATCAATAATG--ATTTCCCAATCTATTTTATATTTTGTTATTAAAATAAAACAAATT
IESPGM.PTET51.1.117.944        TTTCTAAATTTGGAAATTCACCAGTATATTTGACATTTTAAAATTAAAATAC-AAAAATT
IESPGM.PTET51.1.44.7711        -TTCTAAATTAGGAAATTCGCCAATATATTTTATATTTTATAATTAAGTTACAAAAAATT
                                 * * ***   *  ***  *** * ***** * *****   *****  **  * *****
```

```
IESPGM.PTET51.1.160.10020      GTTCTTTACTCTGCTTGCAAAATATGAAATTGTGAATTGCCAATAAATGAAATTCACTCA
IESPGM.PTET51.1.117.944        ACTCCTTATT----TTCCAAAATATGAAATGGTGAATTGACAATAAAGGAAATTCACCAA
IESPGM.PTET51.1.44.7711        ATTCTTTGTT----TTGCAAAATATGAAATGGTAAATT-CCAATAAAGGTTATTCACCAA
                                 ** **  *    ** ************* ** ****  ******* *  ******  *

IESPGM.PTET51.1.160.10020      TATAAATAAAGA---------TATAAGATAAATTCACTTGATATCAATTGGACCTA--TT
IESPGM.PTET51.1.117.944        TGTATATTTGGGTTAATTTCCTAAACTGGAAATTCACGGGAGATAAAATGGGCCTATTTT
IESPGM.PTET51.1.44.7711        TATATATTTGAGTTAATTTCCTATACTGAAAATTCACGGAAAATAAAATGGGCCTA--TT
                               * ** **              ** *    ********   * ** ** *** ****  **

IESPGM.PTET51.1.160.10020      TTGCCTTCAAAACCTATAAATTATAATGATATAT-TTTTATTTATTTAATAATAATTTCT
IESPGM.PTET51.1.117.944        TTATCTCCCAAACTTATAAATTATATCAATATATATTATAT---CTTTAAAATAATTTCT
IESPGM.PTET51.1.44.7711        CTATATCCCAAACTGATAAATTATATTAATATATATTATAT---TTTATTAATAATTTCT
                                *   * * ****  **********   ****** ** ***    **   **********

IESPGM.PTET51.1.160.10020      AACATCAGCTATAtcattg----ttgattgttgagttgaaattatgatggataaatataa
IESPGM.PTET51.1.117.944        TATATCAGCTATA------------aattgttgatattacatttgggtgaaaa-------
IESPGM.PTET51.1.44.7711        GATATCAGCTATAttacagacaaataattatataatttacaatttattgaata-------
                                * **********             *** *  *  * * * *    ** * *

IESPGM.PTET51.1.160.10020      actggcactaacattggtgaaggcacatatattggaatctgataaaggcaattctaataa
IESPGM.PTET51.1.117.944        --------cagcgttacttagaggaaatatttt-----------aacttagttttggtgt
IESPGM.PTET51.1.44.7711        ------------atttctttg-----atttttt------------------atttgaaaa
                                            **  *        ** * **                    * *

IESPGM.PTET51.1.160.10020      agttgaa----cataagcaggtgcttactaaactgatgattgttaaattggttgttgaat
IESPGM.PTET51.1.117.944        aataaaaataccattagttgtcgattatatcat--actatttttgatttccttcttgtat
IESPGM.PTET51.1.44.7711        actaaaag---cactgaaaatcaatcatttaac--aatataatttgtacgatttgaatat
                               * *  **    **           * *    *   *  **  **       **     **

IESPGM.PTET51.1.160.10020      tgcaggaatat-------------------------------------------attatt
IESPGM.PTET51.1.117.944        ctcaactctgttttctt------------tgtttcaatttactttatctttttggatatt
IESPGM.PTET51.1.44.7711        ttaaaaattgttaacatacgaaaagaaaatgccttaat----------------aataaa
                                  *    * *                                             **

IESPGM.PTET51.1.160.10020      gctcttaaacatcttgattctgct------------
IESPGM.PTET51.1.117.944        tgtcattaagattattattattttc-----------
IESPGM.PTET51.1.44.7711        agtagtagattgttatattatgcattgataaagcat
                                 *  *  *       *** *
```

```
CLUSTER 5
MUSCLE (3.7) multiple sequence alignment


ThonS6b:1..566                ------------------------------------------------------------
IESPGM.PTET51.1.34.280841     ------------------------------------------------------------
IESPGM.PTET51.1.131.262422    ------------------------------------------catgagttaattaactta
IESPGM.PTET51.1.109.40673     ------------------------------------atgctaaaggcgctaattgatgat
IESPGM.PTET51.1.58.302214     -----------------------------atggtcttattt-------tttgttagaaca
IESPGM.PTET51.1.18.127217     ggccattaattggcagtattcctccttaaattattgaacttgaaaagaagaattttaata
IESPGM.PTET51.1.128.266698    -------------------------------------------gtgcttccactaataaa
consensus                     ------------------------------------------------------------


ThonS6b:1..566                ------------------------------------------------------------
IESPGM.PTET51.1.34.280841     aatatat---ttcgaggataaacta------attttaaatat-aatataa--------aa
IESPGM.PTET51.1.131.262422    aatttgtggaaatcaaaataatgaa------ataaggaatatcaaaataaaagtatcttg
IESPGM.PTET51.1.109.40673     aacattc---ttcaaatacaaacta-------tcaaaaatat-gtattgatct-----aa
IESPGM.PTET51.1.58.302214     gtgtttc---ccctaaaataatagattattcgtaactaacat-ttaaaatttg-----aa
IESPGM.PTET51.1.18.127217     aatttgt---acctatgatcaagga---ttcgtaataaattt---gctaaattttaatat
IESPGM.PTET51.1.128.266698    atcaggctttgtttttaaggattgcttttttttaataaattt-aataaattctacaagaa
consensus                     ------------------------------------------------------------


ThonS6b:1..566                ------------------------------------------------------------
IESPGM.PTET51.1.34.280841     tgaatattctgttggatattaataaaaaaaatatttagttt-----ctattatatttcat
IESPGM.PTET51.1.131.262422    taaactctttttaatctaattaaataaaactgattttgatt-----------------ga
IESPGM.PTET51.1.109.40673     tggttgcattttaatcttttcagacagttcatcatcagtcaaatcctcataacaataaaa
IESPGM.PTET51.1.58.302214     taagcatttattagcc-----------aagatcatttgact------------agttaag
IESPGM.PTET51.1.18.127217     tgtccatttatatattcccagcaatcaaatatactttagttctttccgataaca----aa
IESPGM.PTET51.1.128.266698    tataactcctgttatattcaaactcagatcataattagtat-----taattgatattaaa
consensus                     ------------------------------------------------------------


ThonS6b:1..566                ------------------------------------------------------------
IESPGM.PTET51.1.34.280841     taggtgatgaaaggtcttcttatatgagg--------aataatcgaaaattataaatcaa
IESPGM.PTET51.1.131.262422    tttgtgattggtagtgtattattttgggaaa------aacagtgtagaataata------
IESPGM.PTET51.1.109.40673     tacatcagcatcactataaattcctaaagcttagcttaaaaataggaagtagattatcgc
IESPGM.PTET51.1.58.302214     tatatcttcgacagttaatctattttata--------agaa------aatttaaaaacga
IESPGM.PTET51.1.18.127217     tagttttttaatagtttaactgcataataataaTACAATTAATTCGTACTTGTTAAATCA
IESPGM.PTET51.1.128.266698    gatactttttcatatttatttacttgatgta------aaatgttagaggaaaaattgcaa
consensus                     ------------------------------------------------------------


ThonS6b:1..566                ---------------------------------------------------TATAGCTGA
IESPGM.PTET51.1.34.280841     tttgaatttataatacgttggcaaattctctttgaattcctaatatttataTATAGCCAA
IESPGM.PTET51.1.131.262422    tttccctatatagatcaacttcaatg--------------caatcaacataTATAGCCAA
IESPGM.PTET51.1.109.40673     ttttatcatata---------------------------------------TATAGCCAA
IESPGM.PTET51.1.58.302214     tttaatgttttaacacaactactcta---------aagttcattgaagtaaTATAGCCGA
IESPGM.PTET51.1.18.127217     TTTATTTATATTTGAC---------------------------------TATATAGCCAA
IESPGM.PTET51.1.128.266698    ttgataaatacaaat------------------------------------TATAGCCAA
consensus                     ---------------------------------------------------TATAGCCAA
                                                                                 ******  *

ThonS6b:1..566                TTTCATCAGCAAAATATACTTAATATTACTACAATATATATAAATTCA-ATTAAAATTTT
IESPGM.PTET51.1.34.280841     ACT-ATTTGCCAAATAAAAATTATATAATATAAATATACAATATTTAATAGAAAA-----
IESPGM.PTET51.1.131.262422    TCTCATTTACAAAATATAAATAATTTTATATCAATATACAATAATTAACAGAAACACTCA
IESPGM.PTET51.1.109.40673     TCTCATTTGCAAAATATAAA-AATATTATATCAATATACAATAATTAACAGAAAAACTCA
IESPGM.PTET51.1.58.302214     TCTCATTTGCAAAATATAAATAATATTATATCAATATAC--TAAATTACAGAAAAACTCA
IESPGM.PTET51.1.18.127217     TCTCATTTGCAAAATATAAATAATATTATATCCATATACAATAATTAACAGAAAAAATCA
IESPGM.PTET51.1.128.266698    TCTCATTTGCAAAATATAAATAATATTACGTCAATATAAAAGAATTAACAGAAAAACTCA
consensus                     TCTCATTTGCAAAATATAAATAATATTATATCAATATACAATAATTAACAGAAAAACTCA
                                * **   * ***** *    ** * *     *****    *  * * *  **
```

```
ThonS6b:1..566                  GAATTAAAGTTAATATATTGATA--ATGTATTAAGTCAATAAAATTCAACTATTTTATTG
IESPGM.PTET51.1.34.280841       ----------CAAAATATTTGA---ACATATTAAGTCAATAAAATACAACTATTTTATTA
IESPGM.PTET51.1.131.262422      TAATTTTAACTAATATATTTGAA--ATATATTAAGTCAATAAAATACAACTATTTTATTG
IESPGM.PTET51.1.109.40673       TAATTTTAATTAATATGTTTGAAAAATATATTAAGTCAATAAAATACAACTATTTTATTG
IESPGM.PTET51.1.58.302214       TAATTTTAATTAATATATTTGAA--ATATATTAAG-CAATAAAATACAACTATTTTATTG
IESPGM.PTET51.1.18.127217       TAAATTTAATTAAAATATTTGAAATATATATTAAGTCAATAAAATACAACTATTTTATTG
IESPGM.PTET51.1.128.266698      TAATTTTAATTAATATATTTGAA--ATACATTAAGTCAATAAAATACAACTATTTTATTG
consensus                       TAATTTTAATTAATATATTTGAA--ATATATTAAGTCAATAAAATACAACTATTTTATTG
                                 ****  *   ** ** **      *   ****** ********* *************

ThonS6b:1..566                  ACTTAATATAAAGTTTCTATTGGTGAAAAGATAAATA--TCTTTCACGGTGAAAAGAAAA
IESPGM.PTET51.1.34.280841       ACTT-GTATATAATTAACATGGT----------------------------GAAAGAAAT
IESPGM.PTET51.1.131.262422      ACTTAGTATATAATTAACACTGG--------------------------TGAAAAGAAAT
IESPGM.PTET51.1.109.40673       ACTTAATATATAATTTACACTGG--------------------------TGAAAAGAAAT
IESPGM.PTET51.1.58.302214       ATTTAGTATATAATTAACACTGGTGGAAGGAAAAATTATCTTTTTGCTGTGAAAAGAAAT
IESPGM.PTET51.1.18.127217       ACTTAGTATATAATTAACACTGGTGAAAAGAAAAATTATCTTTTTATGGTGAAAAGAAAT
IESPGM.PTET51.1.128.266698      ACTTAGTATATAAT-------------------------CTTTTTATGTTGAAAAGAAAT
consensus                       ACTTAGTATATAATTAACACTGGTGAAAAGAAAAATTATCTTTTTATGGTGAAAAGAAAT
                                * **  **** * *                                      *******

ThonS6b:1..566                  TTTTTCTTTTCACAATACT---------------------------------------TT
IESPGM.PTET51.1.34.280841       ATTTT-TTTTTACCTTCCAAGTTATTTGTAATTGTAAAAATAGTTTCCTACCTGAACTCT
IESPGM.PTET51.1.131.262422      ATTTTCTTTTTACATGCCA---------------------------------------TT
IESPGM.PTET51.1.109.40673       ATTTTCTTTTTACATACCA---------------------------------------TT
IESPGM.PTET51.1.58.302214       ATTTTCTTTCAACATACCA---------------------------------------TT
IESPGM.PTET51.1.18.127217       ATTTTCTTTTTACATACCA---------------------------------------TT
IESPGM.PTET51.1.128.266698      ATTTTCTTTTTACATACCA---------------------------------------CT
consensus                       ATTTTCTTTTTACATACCA---------------------------------------TT
                                 **** ***  **    *                                         *

ThonS6b:1..566                  TTTACACAAAACCAAAGAACAATTTTTTGATTTGTAAATATAATAATATAAAAATTATG-
IESPGM.PTET51.1.34.280841       TAAAGAGAAAAAGAAGATCATAATTTTTGATTT----------TAATATAAAACTTATGT
IESPGM.PTET51.1.131.262422      TCAAGAGAAAA----------GATTTTTGATTT----------TAATAT-AAATTTATAT
IESPGM.PTET51.1.109.40673       TCAAGAGAAGATGAAGATTAAAATTTTTGATTT----------TAACAT-AAATTTATAT
IESPGM.PTET51.1.58.302214       TGAAGAGAAAATGAAGATTAAAATTTTTGATTT----------TAATAT-AAATTTTTAT
IESPGM.PTET51.1.18.127217       TCAAGAGAAAATGAAGATTAAAATTTTTGATTT----------TAATAT-AAATTTTTAT
IESPGM.PTET51.1.128.266698      TCAAGAGAAAATGAAGATTAAAATTTTTGATTT----------TAATAT-AAATTTTTAT
consensus                       TCAAGAGAAAATGAAGATTAAAATTTTTGATTT----------TAATAT-AAATTTTTAT
                                *  * * ** *            **********          *** ** *** ** *

ThonS6b:1..566                  ---------GTGAAAAGAAAAAATTTCTTTTCACCAAATAAT-ATGCTTTTATAGAAACC
IESPGM.PTET51.1.34.280841       ATAATATGAGTAAAAAGAAAATGTTTCTTTTCAACATTTAATAATGTTTTCTCAAAAATC
IESPGM.PTET51.1.131.262422      ATATTAAGTGTG--------------------ACCGTTGAATAATG-TTTCTTAAAAATC
IESPGM.PTET51.1.109.40673       -TATTAGGCATGAAAAGAAAAAATTTCTT-TCACCTTTGAATAATTTTTTCT--------
IESPGM.PTET51.1.58.302214       ATATTAAGCGTGAAAAGAAAAAATTTCTTTTCACCATTTAATAATGTTTTCTCAAAAATT
IESPGM.PTET51.1.18.127217       ATATTAAGCGTGAAAAGAAAAT-TTTCTTTTCACCGTTGAATAATTTTTTCTCAAATATT
IESPGM.PTET51.1.128.266698      ATATTAAGCGTGAAAAGAAAAAATTTCTTATCACCATTTAATAATGTTTTTACAAAAATT
consensus                       ATATTAAGCGTGAAAAGAAAAAATTTCTTTTCACCATTTAATAATGTTTTCTCAAAAATT
                                          *                     * *    *** **  ***

ThonS6b:1..566                  AAAAATAACACATAGAAATGTGTTAATACTATATTTCTTCTAT--ATATAATCAAAAATT
IESPGM.PTET51.1.34.280841       G----------ATTCATTCTATTTGGAACTTAATCTATTGAGATG---------------
IESPGM.PTET51.1.131.262422      G----------ATTCATTTGATTTAAAACTGAATATATTAAAAAAATGGAATCGTAAGTA
IESPGM.PTET51.1.109.40673       --------------CATTCGATTTAAAACTGAATATATTTAATTGAAAGAGTCGTAAGT-
IESPGM.PTET51.1.58.302214       G----------ATTCATTTGATTTAAAACTGAATATATTTAAATGATAGAGTCGTAAGT-
IESPGM.PTET51.1.18.127217       G----------ATCCATTTGATTTAAAACTGAATATATTTAAATGATAGAGTCGTAAGT-
IESPGM.PTET51.1.128.266698      G----------ATCCATTTGATTTAAAACTGAATATATTTAAATGATAGAGTCGTAAGT-
consensus                       G----------ATTCATTTGATTTAAAACTGAATATATTTAAATGATAGAGTCGTAAGT-
                                               *      **   ***  ** * **
```

```
ThonS6b:1..566                  AAGATG-----------TTTGTAATGATTCCTGAACTTTTAGAAAAATAAAACGAATTAT
IESPGM.PTET51.1.34.280841       GAAGGTATAAAGTAAATCTT-----------------------------------ACTTT
IESPGM.PTET51.1.131.262422      AAAAGAAAACCGCAAATTTTAGAGCCATTTCTGAA----TAATAGAAAATAACGAATTTT
IESPGM.PTET51.1.109.40673       AAAAGAATACCGCAAATTTTAGAGCCATTTTTGAA----TAGTAGAAAATAACTAATTTT
IESPGM.PTET51.1.58.302214       AGAAGAATACCGCAAATTTTAGAGCCATTTCTGAA----TAATAGAAAATAACTAATTTT
IESPGM.PTET51.1.18.127217       AGAAGAATACCTCAAATTTTAGAGCCATTTCTGAA----TAATAGAAAATAACTAATTTT
IESPGM.PTET51.1.128.266698      AGAAGAATACCGCAAATTTTAGAGCCATTTCTGAA----TAATAGAAAATAACTAATTAT
consensus                       AAAAGAATACCGCAAATTTTAGAGCCATTTCTGAA----TAATAGAAAATAACTAATTTT
                                                  **                                   * * *

ThonS6b:1..566                  T-GGTGAAAAGAAATTTTTTCTTTTCACCATAATTTTTATATTATCATATATACAAATCA
IESPGM.PTET51.1.34.280841       TCTATGTAAAAAGATTATTTTT--------------------------------------
IESPGM.PTET51.1.131.262422      TTGGTTAAAAGAAATTTTTTCTTTTCACGCTTA-ATATATATAAATTTATATTAAAATCA
IESPGM.PTET51.1.109.40673       TTGGTGAAAAGAAATTTTTTCTTTTCCTATTT----------------------------
IESPGM.PTET51.1.58.302214       TTGGTGAAAAGAAATTATTTCTTTTCACGCTTA-ATATATA-AAATTTATATTAAAATCA
IESPGM.PTET51.1.18.127217       TTGGCGAAAAGAAATTATTTCTTTTCACACTTA-ATATATAAAAATTTATATTAAAATCA
IESPGM.PTET51.1.128.266698      TTGGTGAAAAGAAATTTTTTCTTTTCACACTTA-ATATATAAAAATTTATATTAAAATCA
consensus                       TTGGTGAAAAGAAATTTTTTCTTTTCACACTTA-ATATATAAAAATTTATATTAAAATCA
                                *      *** * *** *** *

ThonS6b:1..566                  AAAAATTGTTCTTTGTTTTTGTGTAAAAAAGTATTGTGAAAAGAAAAATTTTCTTTTCAC
IESPGM.PTET51.1.34.280841       ------------------------------------------------------------
IESPGM.PTET51.1.131.262422      AA---------------------------TGGTATGTTTAAAGAAAATATTTCTTTTCAC
IESPGM.PTET51.1.109.40673       ------------------------------------------------------------
IESPGM.PTET51.1.58.302214       AAAATTTTAA--------------------------------------------------
IESPGM.PTET51.1.18.127217       AAAAATTTAATCTTCATTTTCTCTTGAAATGGTATGTAAAAAGAAAATATTTCTTTTCAC
IESPGM.PTET51.1.128.266698      AAAATTTTAATCTTCATTTTCTCTTGAAATGGTATGTAAAAAGAAAATATTTCTTTTCAC
consensus                       AAAATTTTAATCTTCATTTTCTCTTGAAATGGTATGTAAAAAGAAAATATTTCTTTTCAC

ThonS6b:1..566                  CGTGAAAGA--TATTTATCTTTTCACCA--------------------------------
IESPGM.PTET51.1.34.280841       ------------------CTTTTCACCAGTGTTAATTATCTACTAATTAATAAATTAATA
IESPGM.PTET51.1.131.262422      CTTAAAATGATAATTTT-TCTTTCACCAGTGTTAATTATATAC-------TAAGTCAATA
IESPGM.PTET51.1.109.40673       ------------------CTTTTCACCAGTGTTAATTATATAT-------TAAGTCAATA
IESPGM.PTET51.1.58.302214       ------------------TCTTCCATCAGTGTTAATTATATAC-------TAAATCAATA
IESPGM.PTET51.1.18.127217       CATAAAAAGATAATTTTTCTTTTCACCAGCGTTAATTATACAC-------TAAGTCAATA
IESPGM.PTET51.1.128.266698      CATAAAAAGATAATTTTTCTTTTCACCAGTGTTAATTATATAC-------TAAGTCAATA
consensus                       CATAAAAAGATAATTTTTCTTTTCACCAGTGTTAATTATATAC-------TAAGTCAATA
                                                    ** ** *** *********  *        *** * ****

ThonS6b:1..566                  ------------------------------------------------------------
IESPGM.PTET51.1.34.280841       AAATAG----------TTTTATTTTATTGACTAAATATA--TTTCTTATATATTAATTAA
IESPGM.PTET51.1.131.262422      AAATAT----------TTTTATTCTATTGACTTAATATC--TTTCAAATATATGAATTAC
IESPGM.PTET51.1.109.40673       AAATAG----------TTGTATTTTATTGACTTAATATATTTTTCAAATATATTAATCAA
IESPGM.PTET51.1.58.302214       AAATAG----------TTGTATTTTATTGACTTAATATA--TTTCAAATATATTAATTAA
IESPGM.PTET51.1.18.127217       AAATAGTTGCATTTTATTGTATTTTATTGACTTAATATA--TTTCAAATATATTAATTAA
IESPGM.PTET51.1.128.266698      AAATAG----------TTGTATTTTATTGACTTAATATA--TTTCAAATATATTAATTAA
consensus                       AAATAG----------TTGTATTTTATTGACTTAATATA--TTTCAAATATATTAATTAA
                                *****           **  ****  ******** *****   ****  ****** *** *

ThonS6b:1..566                  ------------------------------------------------------------
IESPGM.PTET51.1.34.280841       AA--------------------------------------------ATATATCTTTCGC
IESPGM.PTET51.1.131.262422      AATTATGTGTTTTTC---TGTTCATTATTGTATATTGATATAATGTTATTTATATTTTGT
IESPGM.PTET51.1.109.40673       AATTATGAGTTTTC----TGTTAAATATTGTATATTGATTTAATATTATTTATATTTTGC
IESPGM.PTET51.1.58.302214       AATTATGAGTATTTC---TGTAATTTA--GTATATTGATATAATATTATTTATATTTTGC
IESPGM.PTET51.1.18.127217       AATTATGAGTTTTTC---TGTTAATTATTGTATATTTATATAATATTATTTATATTTTGC
IESPGM.PTET51.1.128.266698      AATTATGAGTTTTTC---AGTTAATTATTGTATATTGATATAATATTATTTATATTTTGC
consensus                       AATTATGAGTTTTTC---TGTTAATTATTGTATATTGATATAATATTATTTATATTTTGC
                                ******* ** ** *    *T     *********** ** ** * ********  ****
```

```
ThonS6b:1..566              ------------------------------------------------------------
IESPGM.PTET51.1.34.280841   AAATGAAATTGGCTATA---------tttc-catacttcttatttttaaacaaaattttg
IESPGM.PTET51.1.131.262422  AAGTGAGATTGGCTATA---------atat-ctaatttgttttttaaaaggaattctcaa
IESPGM.PTET51.1.109.40673   AAATGAGATTGGCTATA---------taat------------tataaaaaaaaattcgaa
IESPGM.PTET51.1.58.302214   AAATGAGATCGGCTATA---------ttgt-------------------aaaatacgcaa
IESPGM.PTET51.1.18.127217   AAATGAGATTGGCTATA---------atttgcagaattttaatagttcaagaaaccttaa
IESPGM.PTET51.1.128.266698  AAATGAGATTGGCTATAtgagctaatttatgaaagcatttgaatttcaaataaaactgaa
consensus                   AAATGAGATTGGCTATA-------------------------------------------
                            ** *** ** *******

ThonS6b:1..566              ------------------------------------------------------------
IESPGM.PTET51.1.34.280841   ttcaattagaga-------aaagaatgattaatatgact--------tttgaatatttgt
IESPGM.PTET51.1.131.262422  ttttttgttata------tgtaatgtcatagatattttc--------gtttattttggaa
IESPGM.PTET51.1.109.40673   t----cgaaattagcttaccaaatttgaggagtactt-----------------------
IESPGM.PTET51.1.58.302214   ttacttaatacaatttgaaaaaatgtcatatctattg------------tcattagttac
IESPGM.PTET51.1.18.127217   t-------------------aagtgtcctgaatactaataccctaaagtctaaatttcta
IESPGM.PTET51.1.128.266698  tcatataatata----------atatcagtgatatttgt---------------------
consensus                   ------------------------------------------------------------

ThonS6b:1..566              ------------------------------------------------------------
IESPGM.PTET51.1.34.280841   gttgtttacaat----------tttcaacagtaaa------------------attcaca
IESPGM.PTET51.1.131.262422  ttcatttcaaataggaaatgaatttaatgagtatatatt-----tta------attaaaa
IESPGM.PTET51.1.109.40673   tgcaattccaacaaatacatattttcgtcattatttaac-----ctataatttattcaaa
IESPGM.PTET51.1.58.302214   taattcaaaaattcacaaaaattattaacgatgaaggat-----ata------gtaggaa
IESPGM.PTET51.1.18.127217   attagttagattataactagaaatttatcgatataagatggccccttttaagctataaca
IESPGM.PTET51.1.128.266698  ---atttgaaacataagtaaattagctattttataaatt-----cataagggtattgtac
consensus                   ------------------------------------------------------------

ThonS6b:1..566              ------------------------------------------------------------
IESPGM.PTET51.1.34.280841   aaaatatatatatatatttt-------tatattgt-------------attttgggaatc
IESPGM.PTET51.1.131.262422  atcatat-----tatatctt-------catactgc-----------caattctaatgaag
IESPGM.PTET51.1.109.40673   aaatatggaacatatattttttggttacatactttcta-----cttttgttttaattgat
IESPGM.PTET51.1.58.302214   aacatttttaaaaagcattt-------catatctaataaaagataggagtttttctaaac
IESPGM.PTET51.1.18.127217   attgttcaatgtcaaatttcctaattacaatttaa---------ttgaattttaattatc
IESPGM.PTET51.1.128.266698  atgaactaattgaatagttg-------tatattac------------attcttaattatt
consensus                   ------------------------------------------------------------

ThonS6b:1..566              -----------------------------------
IESPGM.PTET51.1.34.280841   ttttactc---------------------------
IESPGM.PTET51.1.131.262422  cgatactgaattaaaggcttgtttgcctctgtcaa
IESPGM.PTET51.1.109.40673   ctttacttcatcctaaatttatatgtgctttctcg
IESPGM.PTET51.1.58.302214   ttataaacattagagatgaatgtaaaaaaaaacaa
IESPGM.PTET51.1.18.127217   gtttagatagagca---------------------
IESPGM.PTET51.1.128.266698  aaatagacttttaaaataatacttttcatgcaata
consensus                   -----------------------------------
```

```
CLUSTER 9
MUSCLE (3.7) multiple sequence alignment


IESPGM.PTET51.1.19.475992      taat-aacttgaaatagtgcttttaaactttctttcaatatctatttatcataaaaacat
IESPGM.PTET51.1.96.236752      aaataaaaaagacaaaatgatcataa-------ttagatcaccatt-----caaaaacct
                                *** **   ** * * ** *  ***       **  **  * ***      ****** *

IESPGM.PTET51.1.19.475992      aaaggcagaccta-----aatttag----tctaatctatttgctagatccactttta---
IESPGM.PTET51.1.96.236752      ata-----acttatttacaatttaactattctaactcctctccttcatgttttcttaaga
                               * *     ** **     ******     *****    * * **  **    * ***

IESPGM.PTET51.1.19.475992      -tctaat-tgatagccaaattaagta-ataagtttttcttatattaaaatta-----aga
IESPGM.PTET51.1.96.236752      ttctactataatacataaaatatgtatgccatttatttttgtatgatacttatagttata
                                **** * * ***   *** ** ***    * ** ** ** *** * * ***     * *

IESPGM.PTET51.1.19.475992      attgtttatttcaattgtgttttttcttttagaaccaacacTACAGTCCTCGCAGAAGAT
IESPGM.PTET51.1.96.236752      aaagctttccttaattttcctttgactatc----ttactgcTACAGTCCTCGCAGGGAAT
                               *  * **   * **** *  ***  ** *       *   ***************   **

IESPGM.PTET51.1.19.475992      TATTTGGAAAAAATAATATTACATGCCATTCCTATCGATATAATAATGGCATAGGGATCC
IESPGM.PTET51.1.96.236752      TATTTTAAAAAAATAATATTACATGCCATTCCTATAGATATCAAAATGGCATAGGGATCC
                               *****  **************************** ***** * ****************

IESPGM.PTET51.1.19.475992      CTAACAGTCATCTTCGATGAATATACATACTAATTAATTTAATTTTTTTTTTATAAGAAT
IESPGM.PTET51.1.96.236752      TAAAAAGTGATCTTCGATGAATATACATACTAATTAA------------TTTATAAGAAT
                                 ** *** ****************************            ***********

IESPGM.PTET51.1.19.475992      TCAATTTCTTTTTAATAATAGAATAGGGGAGCATATAAATTGAGAAAAAGATGTCTTAAC
IESPGM.PTET51.1.96.236752      TCAA-TACCTTTTAATAATAGAATAGGGGAGTATATATATTGAGAAAAAGATGTCTTAGT
                               **** * * ********************** ***** ********************

IESPGM.PTET51.1.19.475992      TTAGCTT--TTAATTTAAGATTTTTGTATTGATAATGAGGTGGAGTAAACAATTTTAATA
IESPGM.PTET51.1.96.236752      TTGACCTAGCTAATTAATGA-TATTGCATTGATAATGAAGTGGAGTAAA-AAGTTTAATG
                               **  * *   ***** * ** * *** *********** ********** ** ******

IESPGM.PTET51.1.19.475992      ATATTTGGAATAACATAACTTTATTAAA-TAAAATTAAACATCTATTTAAAAATAAGA--
IESPGM.PTET51.1.96.236752      ATAATTGGAAT-ATATACCTTGATCAAATTAAATTTAAATATCTATTTAAAAATACAAGG
                               *** ******* * *** *** ** *** **** ***** ***************  *

IESPGM.PTET51.1.19.475992      ----ATTTCATTATATAAATATTCAAAAGCCTTCA--------ATCACAATAAAAAAATT
IESPGM.PTET51.1.96.236752      TTTGATTTCAGTATAGAAATATTAAAAAGCCTTCAATATATTCATCACAAT-AAAAAATT
                                   ****** **** ******* ***********        ******** ********

IESPGM.PTET51.1.19.475992      TAAATTTCATCTTTGGAAATATGAATTCTATATTGTGCGCTTAAAGTCCTCAATTTAACC
IESPGM.PTET51.1.96.236752      GATATTTCGT-TCTGGAAATATGAGTTCTATATTGTGAACTTAAAGTCCTCAATTTAAAA
                                * ***** * * *********** ************  *******************

IESPGM.PTET51.1.19.475992      TTATAGAAAACATTTTGGGAATAATGAAATAAAGTGTTATAAAAATGGGACAGAGAATAA
IESPGM.PTET51.1.96.236752      CAATAGAAAACATTTGGGGAATAATCAAATGAAGTGATATAAAAAGTGAACCGAGAACAA
                                 ************* ********* **** ***** ********  * ** ***** **

IESPGM.PTET51.1.19.475992      ATTAAGCTTTCTAA-ATTTAATTATAAAACACTGACAGCGATGAAACCATTTAGTATTTA
IESPGM.PTET51.1.96.236752      ATTAAGCTTTGTAACATTCAATTAAAAGGCACTTATAGCGATGAAGCCATTTTGTGTTTA
                               ********** *** *** ***** **  **** * ********* ****** ** ****

IESPGM.PTET51.1.19.475992      GAGAACCATAAAGGATTAATTCAAATTAGTTTTAAAATAATTGCTGAAAAATTGAATTTT
IESPGM.PTET51.1.96.236752      GAGAATCATAAATGATAAATTCACATTAA-TTTAGAATAATTGCTGAGAAAATGAATATT
                               ***** ****** *** ****** ****  **** ************ *** ***** **

IESPGM.PTET51.1.19.475992      AAATCAATTTAATTATCTAATCTATCTGAATATTCCAGTATGCAAATAATTCCCTGCGAG
IESPGM.PTET51.1.96.236752      AAATCAATTTAATTATCTAATCTATCTGAATATTCCATTTTGCAAACAATTCCCTTCGAG
                               ************************************* * ****** ******** ****
```

```
IESPGM.PTET51.1.19.475992      GACTGTAccatcaaaattaattaaggctttgccgagattgtaactgaaaggagttaaaat
IESPGM.PTET51.1.96.236752      AACTGTActatgagattggtctttctttttacttaattcatctc---------ttaatcc
                                ******* ** * * *    *     *** *  *  *  *  *         ****

IESPGM.PTET51.1.19.475992      tttctagaattataactgcaatttgacatgcaaatgactaaattcttaaatgttaatttt
IESPGM.PTET51.1.96.236752      tcttaacaattata--------ttaatatttata-aatttaattctatcataataatatt
                               * *  * *******        ** * **  * *  * * ******   **  **** **

IESPGM.PTET51.1.19.475992      taatcat-------aacttaaacccttttttacataattgaatctagaatttataaataa
IESPGM.PTET51.1.96.236752      aaagaatttgagagaatacaaagtcaagtttaagtaa----------aatttat--attt
                                **  **       **   ***  *   ****  ***          *******  **

IESPGM.PTET51.1.19.475992      aggattttttaatattaaaaagtagatgtttaga-----------------------
IESPGM.PTET51.1.96.236752      attatactttaatatctcaaagtacattattattctatagttcttctttctttatct
                               *  **  ********   ****** **  ***
```

CLUSTER 12
MUSCLE (3.7) multiple sequence alignment

```
IESPGM.PTET51.1.124.248174      ----acttaagggtggttttactatag-attaa---tttagttcactgtttattctctta
IESPGM.PTET51.1.27.275392       gtataccctcaaattatacttttatagcattaaaatttccacttattgttttattactta
                                    **       *  *  *  ***** *****   **    * * *****  *  ****

IESPGM.PTET51.1.124.248174      att--tttatcatagt-----agctttcaaattatttgcaaacattgg------------
IESPGM.PTET51.1.27.275392       attacctaattataggttgaaagatttaaagtgattt-taattattaatgttattactcg
                                ***   * ** ****      ** *** ** * ****  **  ***

IESPGM.PTET51.1.124.248174      -------atttataaattaaagcatttcctttctattataaattgtagatatggcttaat
IESPGM.PTET51.1.27.275392       tttgactattgattaattgaa------ttttccaagtataaatt---aaaatcagataat
                                       *** ** **** **        ** * * ********    * **    ****

IESPGM.PTET51.1.124.248174      ttatttatgtattttaagagatcttaaaaaatcgttttaaaaccattatacaatTACAGT
IESPGM.PTET51.1.27.275392       atgcttaattatttt------------ggatttattt------catta------TACAGT
                                 *  ***  ******              * *  ***      *****      ******

IESPGM.PTET51.1.124.248174      TGAACTATATATTATTAAACA-TTTTTGAGTTGAAC-TTTTTTTACTCTAATTAATTTTG
IESPGM.PTET51.1.27.275392       CGAGCTGAATATTATTAAACATTTTTTGAGTTGAACTTTTTTTTAGTTTGATTATTTTTG
                                 ** **  ************* ************** ******** * * **** *****

IESPGM.PTET51.1.124.248174      ACAATTTAATAAATCCTTTTTTTATATACATATTTTTAATAGAAATTGTAAAATCAAAAT
IESPGM.PTET51.1.27.275392       ACAATTTACCAAATCCTCTTTTTATCTACTAATTTATATTAGAAATTGTAAATTCATAAT
                                ********  ******* ******* ***  **** ** ************* *** ***

IESPGM.PTET51.1.124.248174      CAAAGTTTAAAAAAATTATAGTTGATA-TTTAATGAA--TGTTTGTAAGATACTTAATCT
IESPGM.PTET51.1.27.275392       CCAAGTTT---AATATTATAGTTGATATTTTAATGAAAGTGTGTGTAAGAAACGTAATCT
                                * ******   ** ************* *********  *** ******* ** ******

IESPGM.PTET51.1.124.248174      ACATTGATTAATCCATTATCTTTTATGTTATTAAAAAAATATTATCTTTAAATCAAAAAT
IESPGM.PTET51.1.27.275392       ACATTGATTAATCCGTTAACTTTAAGGCTATCA---------------------------
                                ************** *** **** * * *** *

IESPGM.PTET51.1.124.248174      TATTTTGCAGCTTTTAAATCAAAATAATGACATTTTAAATTACTAAATGCATTATTAATG
IESPGM.PTET51.1.27.275392       -----------------------------------TAAATTACATAGTGTATTATTAATG
                                                                   ********  * ** **********

IESPGM.PTET51.1.124.248174      AC---GAATATATATTGAATATGATAAATAATTCTTAATTTCG-AAATTATTTTC--TAT
IESPGM.PTET51.1.27.275392       ACTTAATATATATATTGAATTAGATAAATAATTCTTTATTTCGAAAAATATTTTCTATAT
                                **     *************  ************** ****** *** *******  ***

IESPGM.PTET51.1.124.248174      ATAGATTTTTAGAATGAATATAAGCAAGCCTAATTATTTTTTAGTTGATTAATATAATAT
IESPGM.PTET51.1.27.275392       ATAGATTTTTAGAATGAATATAAGCAATTATAAAC-TATTTTAATTGATTAATATAATAT
                                ***************************   ***   * ***** ****************

IESPGM.PTET51.1.124.248174      ATTGATAGTTATAAACAATATTGAAACTTGGATTATGAATTTGGAATCTCTAATATAAAT
IESPGM.PTET51.1.27.275392       ATTGATAGTTATTAACAATAAATAAACTTGGATTATGAATTTAAAATTTCTTATATAAAT
                                ************ *******   *******************  *** *** ********

IESPGM.PTET51.1.124.248174      TAGTATATAAAATGAGGATTTATAAAATTGTCAAAAATAATCAGAGTAAAAAAAGTTCAA
IESPGM.PTET51.1.27.275392       TAGTAGATAAAATGAGGATTTGGTAAATTGTCAAAAATAATCAT-------AAAGCTCGA
                                ***** ***************   *******************        **** ** *

IESPGM.PTET51.1.124.248174      CTCAAA------AATCATTAATAATATAAAACTCGACTGTAtgataatacttta---ttc
IESPGM.PTET51.1.27.275392       CTGTAgctttgtaaatattgagaataaaaattttcaaaatctttttatgccttaaagtcc
                                **  *       **  *** * **** ***  *  *   * *  * ** * ***   * *

IESPGM.PTET51.1.124.248174      tattataatgattaatta--tatgattttcagaatagacagttattaatctcttccaaac
IESPGM.PTET51.1.27.275392       t----taatttctaaatatttatgtagttaaggataaaaggct-tgaatttttt------
                                *    ****   *** **  ****   ** ** *** *  * * * *** * **
```

```
IESPGM.PTET51.1.124.248174      taaagattaatactaataacgccatcattaacttt----attatt-aacatctctaggta
IESPGM.PTET51.1.27.275392       ------------ctaattctttttttattagtttttcaaataattgaataagtctagata
                                            *****       * ****  ***    ** *** ** *  ***** **

IESPGM.PTET51.1.124.248174      aagggttataatcagttgtgtcaccgtttattatttgtaaatttatca---tttgaaatt
IESPGM.PTET51.1.27.275392       aatatttattctca-----------------tattt-taagtttgttaaatattaaagaa
                                **   ****  ***                 ***** *** *** * *    ** **

IESPGM.PTET51.1.124.248174      tattttaatctatt
IESPGM.PTET51.1.27.275392       catttt--------
                                 *****
```

```
CLUSTER 13
MUSCLE (3.7) multiple sequence alignment


IESPGM.PTET51.1.155.211807      -----------------------tatcagtttctaaagaatattcacctcctatctagac
IESPGM.PTET51.1.20.46790        ataa---agaaggtatcttactttttttatttta----------catttttgtttcag-t
IESPGM.PTET51.1.27.294496       ataacctataaacta-atgatattttttatttgaagaaattgatcatccctaatccaact
                                                       * *   ***            **       *  *

IESPGM.PTET51.1.155.211807      aggaaataagagatttagtatttatttcatgt-------ctagagtaaacccaaaaaaaa
IESPGM.PTET51.1.20.46790        acagtttgaatcagttaagctttcttttctttggtaatatgacaatagtctctt------
IESPGM.PTET51.1.27.294496       agtgactataaaaaataagatgtgtatatatt--taatattgaagtagtgatatagaaaa
                                *     *     *  **   * * * *    *           * **

IESPGM.PTET51.1.155.211807      ttagataaagtgatagga---tgaaa---taaaaaaattattttgtagttaata-----g
IESPGM.PTET51.1.20.46790        -tttattcttg-gttgaa----ggactctttcacatatattcatggacctagtttttacg
IESPGM.PTET51.1.27.294496       gtatatgcaagaattgaaaattggac-----------tgttcttagattaagtgt----g
                                 *  **       * * *    * *            *  *  *  *   * *      *

IESPGM.PTET51.1.155.211807      ctcagaagatgttagatcatatagaattctaatttaggaagatttatgaaataattgtta
IESPGM.PTET51.1.20.46790        ccgatcacactttcactgctattcatttaagattt---------------atattttata
IESPGM.PTET51.1.27.294496       ttaaccagaagataagagtcgtaaagttgcaatgt---------------at--------
                                   *  * *   *        *  * **   ** *               **

IESPGM.PTET51.1.155.211807      aTATAGCTGATATCAGAAATTATTTTTAAA------------ATATAATAT-----ATCA
IESPGM.PTET51.1.20.46790        tTATAGCTGTTATTAGAAAATATTTA-AAAATATATTGAAATATATAATATAATTAATCA
IESPGM.PTET51.1.27.294496       tTATGGCTGGTATTAGAAAATATTTATAAAATATAATGAA--ATATAATATAATTAATCA
                                 *** **** *** ***** *****  ***            *********     ****

IESPGM.PTET51.1.155.211807      GTTTGGAGGATAAAA-TAGGCCCATTTTATTTCCTTG------------------TGAAT
IESPGM.PTET51.1.20.46790        GTTGAGGAGATAAAATTATTCCTAT------------------------------TATAT
IESPGM.PTET51.1.27.294496       GTTGATGA-ATTAAATTATGCCTATTTAATAACCGTATTATAAAATTTATATTAGTGAAT
                                ***      ** *** **  ** **                              *  **

IESPGM.PTET51.1.155.211807      TTCCAGTTTAGGAAATTAACCCAAATATATATTGGTGAATTTCCTTTATTTACAATTCAC
IESPGM.PTET51.1.20.46790        CTCCCCT------TATTATC---AAA--ATATTGGTGAAGATGCTGAATTGGAAATTCAC
IESPGM.PTET51.1.27.294496       TTCCACTTTTGGATATTCAC---AAAATATATTGGTGAAGATCCTGAATTGGAAATTCAC
                                 ***  *       ***  *   **   ***********  * **  ***   *******

IESPGM.PTET51.1.155.211807      CATTTCATATTTTGTAAAACAAAGAATAATTTTTTGTATTTTTTAATTATAAAATGTAAA
IESPGM.PTET51.1.20.46790        T-TTTTTCATTTTTTAGTTTAAAGATTAATTTTTTGTA--TTTTAGTTATAA-TTTTATA
IESPGM.PTET51.1.27.294496       --TTTTTCATTTTTTAGTTTAAAGATTAATTTTTTGTA--TTTTAGTTATAATTTTTATA
                                  ***   ***** **    ***** ************  ***** ******  * ** *

IESPGM.PTET51.1.155.211807      TATATTGGTGAATTTCCT-AATTAAGAAATTCACCAATATATTT----------------
IESPGM.PTET51.1.20.46790        TAATAGCTTGAATTTGACAAATGAAATATTTTTTTATTATATTCAAATAGAAGCTGGTAA
IESPGM.PTET51.1.27.294496       CAATAGTGTG---------AATGAAGTATTATTTC-TTAGATTCACATAGTAGATGGTAT
                                 *      **         *** **  * *       ** ***

IESPGM.PTET51.1.155.211807      -TACATTTTAAAATTAAA------ATA--CAAAAAATTATTCTTTGTTTTACAAAATATG
IESPGM.PTET51.1.20.46790        ATACATCAAAAAAAGAAACAATTGATAG-TGAGAGGCAGACGTATAATTTTTAATGAATA
IESPGM.PTET51.1.27.294496       GTATAT--AAAAAAGAAATAATTGATAGTTTAGAGACTGACCTATAATTTATAATGAATA
                                 ** **   ****  ***      ***    * *        * *  ***  **   **

IESPGM.PTET51.1.155.211807      A-----------AATGGTGAATTGT-----------------------------------
IESPGM.PTET51.1.20.46790        AGTCAGTATTTTAGTGGAAATTTGCTTATTATAATATTTATATAATTCAATATTAATCTG
IESPGM.PTET51.1.27.294496       AG-------TTTAG----AATTTGT-----------------------------------
                                *           *      * ***

IESPGM.PTET51.1.155.211807      ------AAATAAAGGAAATTCACCAATATATATTTGGGTTAATTTCCTAAACTGGAAATT
IESPGM.PTET51.1.20.46790        AATATCCAAAAGTGGAAATCCACTTATATATAT---------ATTTATAA----------
IESPGM.PTET51.1.27.294496       -----------GTGGAAATTCACTTATATATA-----------TTTATAA----------
                                             ****** ***  *******           **  ***
```

```
IESPGM.PTET51.1.155.211807      CACAAGGAAATAAAATGGGCCTATTTTATCTCCCAAACTGATTAATTATAATAATATATA
IESPGM.PTET51.1.20.46790        TAAGGGGATGTAAAATAGACATATTTTATCTCCTAAACTAATTAATTAT--TATAATTCA
IESPGM.PTET51.1.27.294496       TAAGGGGAGATAAAATAGGCATATTTTAATTCTTCAACTGATTAATTATAATATATTTCA
                                 *   ***  ****** * * *******  **   **** *********  **   *  *

IESPGM.PTET51.1.155.211807      TTATAATTTAAAAATAATTTCTGATATCAGCTATA-------------ata---------
IESPGM.PTET51.1.20.46790        TTATATTTTATAAATATTTTCTAATAACAGCTATAtaatgttaatggaataatacgagat
IESPGM.PTET51.1.27.294496       TTATATTTTATAAATATTTTATAATAACAGCTATA-------------acaatatcacat
                                ***** **** ***** *** * *** ********             * *

IESPGM.PTET51.1.155.211807      --------------aaattttact-------attcaaatgtaattaccttaaa-------
IESPGM.PTET51.1.20.46790        ttatatgtatcaacataatttgctcaaaca-gtataattacaattatcg-----------
IESPGM.PTET51.1.27.294496       t-------------agattttacttggatatgaataattggaattaccaaaaggccgctc
                                              * * *** **           ** *  ***** *

IESPGM.PTET51.1.155.211807      tatatgatataaatttaataattgattaaatcttttattc------tcttaaagaatctt
IESPGM.PTET51.1.20.46790        taatttatataga--taattatctactgatattaatcttgaaat--gttaataa------
IESPGM.PTET51.1.27.294496       tagagtatataga--ggattacaaattcgaattgctataaaataagccaagaaaacatc
                                **    ***** *    ** *   * *     *  * *            * *

IESPGM.PTET51.1.155.211807      tttgttt----atcgttactgaatctttt-ttaaatgatatcatttataagagacactac
IESPGM.PTET51.1.20.46790        --tgcttgcaaataatt--tgatttcttcgatgtataacttc------------------
IESPGM.PTET51.1.27.294496       tttacctaaaggttattagtgattctttt------taagttc------------------
                                  *   *     *  **  *** *  **       * *  **

IESPGM.PTET51.1.155.211807      gatgaaagaaaaacacaaggttcaatccaaaaaacat-aattttaaaattaaaacat
IESPGM.PTET51.1.20.46790        -------gtaatagatcagtgctcactaattaaata--agttctagga---------
IESPGM.PTET51.1.27.294496       -------caaaaatataacgattatgtaattatatatctattctaaaaatggaa---
                                         ** * *  *          *  * * *    ** **  *
```

```
CLUSTER 14
MUSCLE (3.7) multiple sequence alignment


IESPGM.PTET51.1.184.21279      -ttgattcattttaattcttgccattttaaaaac------------aataatcattataa
IESPGM.PTET51.1.21.430950      ----at--actatgatggccactattaaaaaatt-----------gattattgattcttg
IESPGM.PTET51.1.58.200038      gtcgat--agttcgatgttatttaatggaagatttggacccacagtattataagttgtgg
                                   **  * *   **       * *  ** *              * **    ** *

IESPGM.PTET51.1.184.21279      tagg----tatattgggcttaccactaattctacaa-agaataaca-------agtggat
IESPGM.PTET51.1.21.430950      gaga----actagcagattt--cagagattatttaactgaattgcacttcttgaattaat
IESPGM.PTET51.1.58.200038      gaggagtttatgcaggatttttatggagttgtacagtacaattata-----tatactaat
                                **       *    *  **        ** *  *    ***   *            **

IESPGM.PTET51.1.184.21279      ataaaata-ataaaaagtgctttgacagttaaaa-------------------cattgtc
IESPGM.PTET51.1.21.430950      ttggattc-acattagattacttttcatttaacactagtttttatttttttttccactacc
IESPGM.PTET51.1.58.200038      ataaaatagattagatctgttgtatcaatccaaa----------------------tatt
                                *  * *  *    *  *    *  ** *  * *                      *

IESPGM.PTET51.1.184.21279      aagtagc----cattatggtttgaagagaatatccaaattaa-------tagagaagtat
IESPGM.PTET51.1.21.430950      aaatcacttgatgctctatttttttaaattctattg-ttttga-------ttcTACAGTTC
IESPGM.PTET51.1.58.200038      ggatcac----------atttcccagagagtattgacactgatttcctttgagcattgT
                                  *  *            **   * *   ***      * *       *     * *

IESPGM.PTET51.1.184.21279      aaaat-agtataattgaTACATTAAAT--TTATTATTATTATTATATTTTTTTTATCATA
IESPGM.PTET51.1.21.430950      ATTTTTTGTTTGTTTATTTAATTAAATAATTATTATTATTATTATATAATTTTTATCTTA
IESPGM.PTET51.1.58.200038      ATTGTAAGTATTTTT---------AATATTTATTATTATTA--------TTTTTATCTTA
                               *   *  ** *  **         ***  ************        ******** **

IESPGM.PTET51.1.184.21279      AAAATTCATGCAGGAAAAAATAGACAATGAGTAAATCCA-AGCCTTTCAA-AAATTTCCT
IESPGM.PTET51.1.21.430950      TTAAAAAATGCAGGGGAAAATAGACGAAGAAGAGCTTTTCAATCTTTCAGCAAATTTCCT
IESPGM.PTET51.1.58.200038      TTAAAATATGCAGGAG-AAATAGAAGAAGAAGTGATTTATCCTATTTCAGCAAATTTCTT
                                 **   *******   *******  * **     *        *****  ******* *

IESPGM.PTET51.1.184.21279      TTGTGAGTCTCCAGTCAATGGCTGACCAATCTTTTAGGGTTTATTTATAACCTTCTACAA
IESPGM.PTET51.1.21.430950      TCCTGAGTATCCAGTCAATGGCTGGCCAATCTTCTAGGGCCTATTTAATACCTTCTACAG
IESPGM.PTET51.1.58.200038      TCCTGAGTATCCAGCCAATGGCTGGTCAATCTTCTAGGGCCTATTTAATACCTTCTACAG
                               *  ***** ***** *********  ******* *****  ******  **********

IESPGM.PTET51.1.184.21279      TGCCTTAGATGATTTGTGTGAATTTAAAGCATCAAATATTCTTTAGCTTGGAGTTGGAAG
IESPGM.PTET51.1.21.430950      TGCCCTGGATGATTTGTGTGAGTTTAAAGCTCCAAATGTTCTTTAATGTGGAGTTGGAAG
IESPGM.PTET51.1.58.200038      TGCCCTGGATGATTTGTGTGAGTTTAAAGCACCAAATGTTCTTTAATGTGGAGTTGGAAG
                               **** * ************** ********  ***** *******   ************

IESPGM.PTET51.1.184.21279      GAGAGTTAAAGACATCCTACCAACACTTAATAGGGATCTATCTAGGATGGGCCTAATTAC
IESPGM.PTET51.1.21.430950      GAGGGTCAAAGACATCCTTCGCGCATTGAAGAGGTACCTTTCTAGGATGGGCCTAATTAC
IESPGM.PTET51.1.58.200038      GAGGGTTAAAGACATCCTCCGCACATTGAAGAGGTACCTTTTTAGGATGGGCCTAATTAC
                               *** ** *********** *   ** * ** *** * ** * ******************

IESPGM.PTET51.1.184.21279      CTGA----------GAATTTTGTCTT-GGTTTGAGTTTTGAGA--AAAAATAGAAATTCA
IESPGM.PTET51.1.21.430950      TTGGTCACCTGAGCAACTTTAGTCTT-GGTTTGAGACTCGGGGGAAAAAATAGGAATTAG
IESPGM.PTET51.1.58.200038      CTGTTCACCTGAGCAACTTTAGTCTTGGGTTTGAGACTCGGGAG-AAAAATAGGAATTCG
                                **            * *** ***** ********  * * *   ******** ****

IESPGM.PTET51.1.184.21279      GAAATTCCAGAAAGCTAGGTAGGAAAAATGATGAAAATTAAAAGGG----------TGCA
IESPGM.PTET51.1.21.430950      GAAAATCCTAAAAGATACACGGGAAACATGATAAAACTTCAAAAGGATAAATTCAATACA
IESPGM.PTET51.1.58.200038      GAAAATCCTAAAAGATACGTGGGGAGCATGATAAAACTTCAAAAGGATAAATTCAATACA
                               **** ***  **** **    ** *  ***** *** ** *** **          * **

IESPGM.PTET51.1.184.21279      CTAGGAAAAGAAAAGATAAAACTGAAGACTTTTAAGATGCACCAGGGATTAAATCTAAGT
IESPGM.PTET51.1.21.430950      CTAACAAGAAACGAAGATAAAATGAGACCTTTAGTGAAGCGCCAGGGGTGAAACCTAAGT
IESPGM.PTET51.1.58.200038      CCAACAAGAGATGAAGATAAACTGAGACCTTTAGTGAAGCGCCAGGGGTGAAACCTAAGT
                               * *  ** * *  *    *** ***   ****   ** ** ****** * *** ******
```

```
IESPGM.PTET51.1.184.21279      TAAAATCTTAGCCACAGTAATC---AAAAGGCAGTTTCAAAATTTCATCTCAGCCCAAAT
IESPGM.PTET51.1.21.430950      TAGAGACTTAAGCTTAGTAGCCTATTAAAGGTAATTTTGCAACTATGACTCAGCCCTCTT
IESPGM.PTET51.1.58.200038      TAGAGACTTAGGCTAAGTAGTC---CAAAGGCAGCTCTGCAGCTGTGACTCAGCCCTCTT
                               ** *  ****  *  ****  *    ***** *  *    *  *    ********   *

IESPGM.PTET51.1.184.21279      CTGGGAATAAAA---GGGAAAAGAAAGATAAGCATAGACAAAATGAAATTACAAAG---C
IESPGM.PTET51.1.21.430950      AGGGGAATAATAAGGTGAAAAAGAAAGATAAGGAAGGATAGCATGGTGTTCCTAAGAATT
IESPGM.PTET51.1.58.200038      AAGGGAATAATAATGGGAAAAAGAAAGATAAGGAAAAACACCGTAGTGTTCCTAAGAATT
                                 ******** *    * ************** *   * *   *    ** * ***

IESPGM.PTET51.1.184.21279      CTTTTTTAACACAGCCTTTAGTATGTGGTCAATATATAAAATCATATAAGAACGTTCA--
IESPGM.PTET51.1.21.430950      CTTCTCCAACACAGCCTCTTGTATGTGGCCATTGCCTCAGGCAATATAAAAACATACGGT
IESPGM.PTET51.1.58.200038      CTTCTCCAACACAGCCTCTTGTATGTGGCCATTGCCTCAGGCAATGCAAGAACATACGGT
                               *** *  ********** * ******** ** *   * *    **  ** *** * *

IESPGM.PTET51.1.184.21279      -ATTTAGGACCCAAATGTACCAGGTCCATCATAACAAAGCCACTGAAGGCTCTTCTCATT
IESPGM.PTET51.1.21.430950      CATATAGAACTCACATGTATTAGGTCCACATCAAGTAAGTTCCTGATGGTTCCTCTCATA
IESPGM.PTET51.1.58.200038      CATATAGAA--CACATGTATTAGGTCCACATGAAATAATTTCCTGAAGGCTCCTCTCATA
                                ** *** *  ** *****  *******    **  **    **** ** ** ******

IESPGM.PTET51.1.184.21279      AATTAATAGGTAAACCTTGTTTTAGAGCTGTGAATTTAAAGCCAAACCCTTGTACTAAGG
IESPGM.PTET51.1.21.430950      TGTTACTAGGATAACCTTTAGTAAGAGCTGTGCCTCTTAAACCTAACCCATCTATCAGGT
IESPGM.PTET51.1.58.200038      CGATATTAGGATAACCTTTAGTAAGAGCTGTGCCTCTTAAAACTAACCCATCTATCAGGT
                                  ** ****  ******   * *********  * * **  * ***** * **  * *

IESPGM.PTET51.1.184.21279      CTGACCAACCATGGTAGTCAAAACAGGACAAAGACAAAATATTCTCATTAGAACCAAGTC
IESPGM.PTET51.1.21.430950      CTGACCAACCTCGATAGCCAAGACAGGACACTCTCCACATTCCGTCATTAGGACCAATTC
IESPGM.PTET51.1.58.200038      CTGACCAACTTTGATAGCCAAGACTGGACATTCTCCACATTCCGTCATTAGGACCAATTC
                               *********   * *** *** ** *****    * * **    ******* ***** **

IESPGM.PTET51.1.184.21279      AGAGACATATTGAAATCATTGAGGAAACACCTTAGGGCAATATAGTCCATCACCGGAAGG
IESPGM.PTET51.1.21.430950      AGAGAGACTCTGAGATGATAGAGGAGACACCTTAGGGCAATATTGTCCATGGCCGAAAGG
IESPGM.PTET51.1.58.200038      AGAAATATTCTGAGATGATAGAGGAGACACCTTAGGGCAATATTGTCCATGGCCGAAAGG
                               *** * *   *** ** ** ***** ***************** ******  *** ****

IESPGM.PTET51.1.184.21279      CAACTAGAACTCTTAGTGCTCTGAATCTTGATCCACCTAAGTGGAAAG---------GAA
IESPGM.PTET51.1.21.430950      C------------TCATACTACCGTTCCTGGTCCACCTAAGGGGAAAAAGGGGAGGTAAA
IESPGM.PTET51.1.58.200038      C------------TCATACTACCGATCCTGGTCCACCTAAGGGGAAGAAGGGAAGGTAAA
                               *            *  * **     ** ** ********** ****            **

IESPGM.PTET51.1.184.21279      AATGAT-TGAATTATTATTATTTTTTTAATTAATCAAATTA-GACTAATAAGGTTGATTT
IESPGM.PTET51.1.21.430950      AATGATAAGGGTTATCA----TTTAATAAATAAATAAATTATTATTATTATTATTATTAT
IESPGM.PTET51.1.58.200038      AATGAT-TGGGTTTTCA----TTTCATAATAAATAAAATTATTATTATTATTATTATTAT
                               ******  *  ** * *    ***  ***  **  ******  * ** **   **  * *

IESPGM.PTET51.1.184.21279      GCTTATTCTAAAATTTG---TGAATTATAATTAATAATCTC--CAAGAATT---CCTAAA
IESPGM.PTET51.1.21.430950      TATTATTATTA-------------TTATTATTAATAGCTATAacaagaactaatcctaat
IESPGM.PTET51.1.58.200038      TATTATTATTAATTTTAAATAAATTTACAATAaattattatcataacaattaggggtaaa
                                 ***** * *             ***  ** ***         ** ** *     ***

IESPGM.PTET51.1.184.21279      TTCAACCACTAACTTATTGTAgcaagattactactattgcaaattcaatatctttattct
IESPGM.PTET51.1.21.430950      ataaa-----gggttaatgtaattgaa-------gaactcaaacaaatcatctt------
IESPGM.PTET51.1.58.200038      ---aaacaatgggtagatttgtttcagttaacgtggatttgactaagacatcca-----t
                                  **        *   * *                     *       ***

IESPGM.PTET51.1.184.21279      ggaagaatcttgaattaatggatatagcccttttttggtgggtcataagaaactgcagga
IESPGM.PTET51.1.21.430950      ---aaggtttcaaatctccagtttcatttctctaattaaa----ttcagcaac-------
IESPGM.PTET51.1.58.200038      taaagattgtcata-----agttgcagagcttcatttgtg----ataagcaat-------
                                  *   * *   *      * *  *   **    *         * ** **

IESPGM.PTET51.1.184.21279      atctatctcttaatatactgagatgaaggatcaaagttaataatagaataa-ctaaaaaa
IESPGM.PTET51.1.21.430950      atccacctttaagaattct----ccatgaatatgagtttcatagatatcgatctcaatag
IESPGM.PTET51.1.58.200038      ------------gattatt----taataaatatgattcaattataaattaataccattaa
                                              *  *      *   **   * *     * * *   *    *  *
```

```
IESPGM.PTET51.1.184.21279      acaataatagcaagcgaggtatcccatattttcagattagta--
IESPGM.PTET51.1.21.430950      gcatttctatttgtttatttttttatagattcccaattt------
IESPGM.PTET51.1.58.200038      acaattcaagta------------cagttttacagtttatcttt
                                ** *   *                *  **  **  **
```

CLUSTER 15
MUSCLE (3.7) multiple sequence alignment

```
IESPGM.PTET51.1.28.278632     gtcccatgaattgacttaataagataaaactaccctcctgattcgtgtatgagtaagtat
IESPGM.PTET51.1.4.361312      ------------gccatgatgttgtaatacttatcttc-----------ggaataaatac
                                          * * * **    *** ***   ** *            ** *** **

IESPGM.PTET51.1.28.278632     tt-----ccaacattgtgctttattgtttatacatattgtggtaa---------atcatt
IESPGM.PTET51.1.4.361312      ttcctaaccaacaaaatatgcatttgtataatcttcctttaataatctttttccatcagt
                              **     ******   *      **** **  * *  * *  ***         **** *

IESPGM.PTET51.1.28.278632     tacaatctgaatagatatatcctctttag------cgt-tgtaaaatcctata--aggga
IESPGM.PTET51.1.4.361312      tccaatactatcagctgggtctacagtaggagctatgtgtatcacattcgaaactaaagg
                              * ****   *  ** *   **  *  ***       ** * * * ** * * *  *  *

IESPGM.PTET51.1.28.278632     ttatcttaaaatattgtcaaaataacaataaaacattatccat--TATAGTTGATATCAG
IESPGM.PTET51.1.4.361312      ttaaagcagttcatttggaacttcatatggtaccccaatttattaTATGGTTGATATCTG
                              ***    *    ***   **  * * *    * *   **  **  **  ********* *

IESPGM.PTET51.1.28.278632     AAATTATTATTAAAATATAATATATATTAATATAATTTATCAGTTTAGGAGATAAAATAG
IESPGM.PTET51.1.4.361312      AAATTTAAAATAAAATTTAATAGATATTAATATAATTTATCATTTTGGGAGATAAAATAG
                              *****   * ****** ***** ******************* *** *************

IESPGM.PTET51.1.28.278632     GCCATTTTATCTCCCGTGAATTTCCAGCTTAGGAAATTAACCAAAGGAAATTCACCAATA
IESPGM.PTET51.1.4.361312      GTTCATTTACCTCTTGTGAATTT-CAGTTTCTGAAATT--CCTA---AATTTCATT--TA
                              *    **** ***  ******** *** **  ******  ** *   ** ****    **

IESPGM.PTET51.1.28.278632     TATATAAATAAGCTAATCTCCTAAACTGGAAATCCACGAGTGATAAATTGGGCCCTAATT
IESPGM.PTET51.1.4.361312      TCTACATCTTAGTTAATTTCAGATATTGGAAA---------GGTAAATGAG--CCTATTT
                              * ** *  * ** **** **  * * ******         * *****  *  **** **

IESPGM.PTET51.1.28.278632     TATCTCTCAAACTGATAAATTATATTAATATATGTTATATTTTAATAATAATTTCTGATT
IESPGM.PTET51.1.4.361312      TATCTCCCAAAATGATAAATTATGTGAATATACAGAATATTTTAATTA-AATTTCAGGTA
                              ****** **** *********** * ******    ********** * ****** * *

IESPGM.PTET51.1.28.278632     TCAGCTATA----tccttcatataaaatattatcaaattcaaatgacaa-agtgttagtt
IESPGM.PTET51.1.4.361312      TCAGCTATAtagatccagtttattagtcagtaactactgtgattaatggtagtgtta---
                              *********    ***    *** *   * ** * * *   * * *    *******

IESPGM.PTET51.1.28.278632     aaagttttgaaagagtaaaatactttgtgggtaattatttatttatttggaTCTATATCA
IESPGM.PTET51.1.4.361312      aaatttctg---------tatatattgtacctcatt-----tacatttccaCATTCAGCT
                              *** ** **          ***  ****   * ***     *  ****  *  *  * *

IESPGM.PTET51.1.28.278632     gcagaaacaaccaaccaattcaccttatcattggc------------acgttcaatggaa
IESPGM.PTET51.1.4.361312      gttgaaattcc-------ttcacataaattttgacctccagttgatgatgttgcttgtaa
                              *  ****   *       ***** * *   *** *            * ***   ** **

IESPGM.PTET51.1.28.278632     ta-----gataaattgttgtccttgtataataatttaatcttttaatatac
IESPGM.PTET51.1.4.361312      tatccttaagagtttcttaattttttattctatttctgtattttaatcaat
                              **      * *  ** **    ** ***  ** **   * *******  *
```
