# Supplementary material for: The Paramecium Germline Genome Provides a Niche for Intragenic Parasitic DNA: Evolutionary Dynamics of Internal Eliminated Sequences
Source: PLoS Genet. 2012 Oct 4;8(10):e1002984. doi: 10.1371/journal.pgen.1002984 (PMC3464196; doi:10.1371/journal.pgen.1002984)
Supplement: Text S3 — PCR approach to validate IESs with homology to Thon solo TIRs. (PDF) [file pgen.1002984.s015.pdf]

TextS3.

PCR validation of IESs from cluster 5, homologous to solo TIRs of the *Thon* transposon

We used 2 different approaches to validate the size and sequence of IESs with homology to *Thon* solo TIRs. The first approach (A in schema, below) involved PCR primers anchored in MAC-destined sequences flanking the IES, and were used to amplify PGM DNA using the Expand Long Template PCR System (Roche Applied Science) with buffer 3 that contains detergents to help denature foldback structures that might be formed by the palindromic TIR of Thon (cf. TextS2), according to the recommendations of the supplier.

The second approach (B in schema, below) involved PCR primers anchored in flanking IESs, allowing amplification of total cellular DNA, possible for two of the IESs in cluster 5 (cf Table, below). In order to sequence the products, two primers internal to the solo TIR were designed in the central region between the palindromic repeated sequences (cf. Text S2 and schema, below). The same Long Template PCR System was used for the amplifications.

In the case of the three IESs that were completely sequenced, no differences were found with the predicted IES sequences obtained using PGM DNA assembly and the MICA pipeline.

The sequences of the primers are available on request.

Scaffold	Position of IES	Expected Size (nt)	Observed Size (nt)	IES size	Sequence	PCR Method
scaffold51_109	40673	921	~900	571	ND	A
scaffold51_128	266698	1131	~1100	689	Confirmed IES	B
scaffold51_131	262422	980	~950	630	ND	A
scaffold51_18	127217	1189	~1100	770	Confirmed IES	B
scaffold51_34	280841	925	~950	512	Confirmed IES	A
scaffold51_58	302214	1006	~1000	640	ND	A

Table of PCR and sequencing results for cluster 5 IESs. ND, not done. The positions of IES insertion are relative to the MAC strain 51 reference genome.

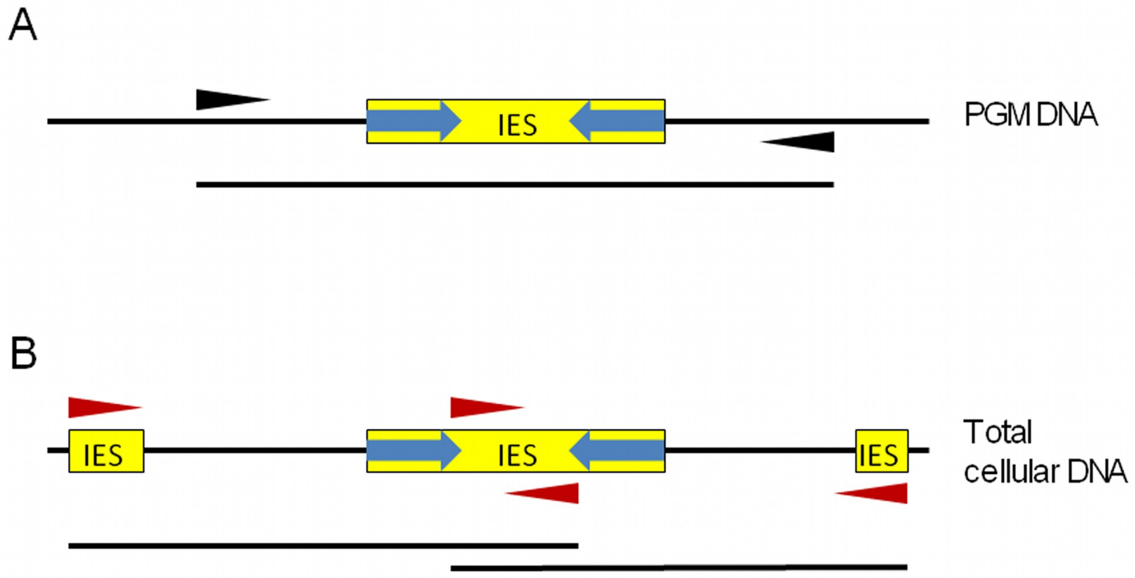

Schema. PCR methods A and B. The blue arrows represent the palindromic repeats of *Thon* TIRs.
