# Supplementary material for: The Paramecium Germline Genome Provides a Niche for Intragenic Parasitic DNA: Evolutionary Dynamics of Internal Eliminated Sequences
Source: PLoS Genet. 2012 Oct 4;8(10):e1002984. doi: 10.1371/journal.pgen.1002984 (PMC3464196; doi:10.1371/journal.pgen.1002984)
Supplement: Text S4 — A maximum likelihood framework for testing hypotheses about IES evolution. (PDF) [file pgen.1002984.s016.pdf]

A maximum likelihood framework for testing hypotheses about IES evolution

Notation

- WGD1: recent whole genome duplication
- WGD2: intermediate whole genome duplication
- N_q: count of IES quartets with a particular quartet pattern q of present and absent IESs.
- ρ_3: fraction of quartets with origin before WGD2.
- ρ_2: fraction of quartets with origin between WGD2 and WGD1.
- ρ_1: fraction of quartets with origin between WGD1 and the present.
- $\rho_1 + \rho_2 + \rho_3 = 1$
- $\delta_{3,2}$: survival rate for IESs created before WGD2 during the period between WGD2 and WGD1.
- $\delta_{3,1}$: survival rate for IESs created before WGD2 during the period between WGD1 and the present.
- $\delta_{2,1}$: survival rate for IESs created between WGD2 and WGD1 during the period between WGD1 and the present.

Assumptions

- Independence of IES decay processes within quartets.
- Independence of IES decay processes across quartets.
- Negligible probability of two insertion events occurring at the same place after a WGD event.

Observability

There are 16 types of IES quartets, but of these, several are observationally equivalent and one is unobservable:

N_q	Observable	Types
N_{1111}	Yes	1111
N_{1110}	Yes	1110,1101,1011,0111
N_{1010}	Yes	1010,1001,0110,0101
N_{1100}	Yes	1100,0011
N_{1000}	Yes	1000,0100,0010,0001
N_{0000}	No	0000

Model

We treat the observable quartet type counts as the sum of the number of counts arising from each of three decay processes: one for IESs created before WGD2 that survived to WGD2 (g=3); one for those created between WGD2 and WGD1 that survived to WGD1 (g=2); and one for those created after WGD1 that survived to the present (g=1). Under these assumptions, the probability $p_{g,q}(\delta)$ of observing each of the IES patterns q, given that an IES originated in generation g, is given in the following table:

$p_{g,q}(\delta)$	$g = 1$	$g = 2$	$g = 3$
$q = 1111$	0	0	$(\delta_{3,2})^2(\delta_{3,1})^4$
$q = 1110$	0	0	$4(\delta_{3,2})^2(\delta_{3,1})^3(1-\delta_{3,1})$
$q = 1010$	0	0	$4(\delta_{3,2})^2(\delta_{3,1})^2(1-\delta_{3,1})^2$
$q = 1100$	0	$(\delta_{2,1})^2$	$2(\delta_{3,2})^2(\delta_{3,1})^2(1-\delta_{3,1})^2$ $+2(\delta_{3,2})(1-\delta_{3,2})(\delta_{3,1})^2$
$q = 1000$	1	$2\delta_{2,1}(1-\delta_{2,1})$	$4(\delta_{3,2})^2(\delta_{3,1})^1(1-\delta_{3,1})^3$ $+4(\delta_{3,2})(1-\delta_{3,2})(\delta_{3,1})(1-\delta_{3,1})$
$q = 0000$	0	$(1-\delta_{2,1})^2$	$(1-\delta_{3,2})^2$ $+\ 2(\delta_{3,2})(1-\delta_{3,2})(1-\delta_{3,1})^2$ $+\ (\delta_{3,2})^2(1-\delta_{3,1})^4$

The final row of the table is the case of IESs that were eliminated entirely by decay, and are censored observations. Thus, the relative frequency of the other patterns increases from the values in the table above by a factor of $1/(1 - p(q = 0000|g))$

Therefore, the relative frequency of the five observables as a function of ρ_2, ρ_3, $\delta_{3,2}$, $\delta_{3,1}$, and $\delta_{2,1}$ is:

$$p(q = 1111|\rho, \delta) = \rho_3 \left(\frac{(\delta_{3,2})^2(\delta_{3,1})^4}{1 - ((1-\delta_{3,2})^2 + 2(\delta_{3,2})(1-\delta_{3,2})(1-\delta_{3,1})^2 + (\delta_{3,2})^2(1-\delta_{3,1})^4)} \right) \quad (1)$$

$$p(q = 1110|\rho, \delta) = \rho_3 \left(\frac{4(\delta_{3,2})^2(\delta_{3,1})^3(1-\delta_{3,1})}{1 - ((1-\delta_{3,2})^2 + 2(\delta_{3,2})(1-\delta_{3,2})(1-\delta_{3,1})^2 + (\delta_{3,2})^2(1-\delta_{3,1})^4)} \right) \quad (2)$$

$$p(q = 1010|\rho, \delta) = \rho_3 \left(\frac{4(\delta_{3,2})^2(\delta_{3,1})^2(1-\delta_{3,1})^2}{1 - ((1-\delta_{3,2})^2 + 2(\delta_{3,2})(1-\delta_{3,2})(1-\delta_{3,1})^2 + (\delta_{3,2})^2(1-\delta_{3,1})^4)} \right) \quad (3)$$

$$
\begin{aligned}
p(q=1100|\rho,\delta) &= \rho_2\left(\frac{(\delta_{2,1})^2}{1-(1-\delta_{2,1})^2}\right)\\
&+ \rho_3\left(\frac{2(\delta_{3,2})^2(\delta_{3,1})^2(1-\delta_{3,1})^2}{1-((1-\delta_{3,2})^2+2(\delta_{3,2})(1-\delta_{3,2})(1-\delta_{3,1})^2+(\delta_{3,2})^2(1-\delta_{3,1})^4)}\right)\\
&+ \rho_3\left(\frac{2(\delta_{3,2})(1-\delta_{3,2})(\delta_{3,1})^2}{1-((1-\delta_{3,2})^2+2(\delta_{3,2})(1-\delta_{3,2})(1-\delta_{3,1})^2+(\delta_{3,2})^2(1-\delta_{3,1})^4)}\right) \quad (4)\\
p(q=1000|\rho,\delta) &= (1-\rho_2-\rho_3)\\
&+ \rho_2\left(\frac{2\delta_{2,1}(1-\delta_{2,1})}{1-(1-\delta_{2,1})^2}\right)\\
&+ \rho_3\left(\frac{4(\delta_{3,2})^2(\delta_{3,1})^1(1-\delta_{3,1})^3}{1-((1-\delta_{3,2})^2+2(\delta_{3,2})(1-\delta_{3,2})(1-\delta_{3,1})^2+(\delta_{3,2})^2(1-\delta_{3,1})^4)}\right)\\
&+ \rho_3\left(\frac{4(\delta_{3,2})(1-\delta_{3,2})(\delta_{3,1})(1-\delta_{3,1})}{1-((1-\delta_{3,2})^2+2(\delta_{3,2})(1-\delta_{3,2})(1-\delta_{3,1})^2+(\delta_{3,2})^2(1-\delta_{3,1})^4)}\right) \quad (5)
\end{aligned}
$$

Given these expressions, we can write out a likelihood function for the observed data, given the underlying parameters:

$$
\mathcal{L}(\rho,\delta|N_q) = \frac{(\sum_q N_q)!}{\prod_q N_q!}\prod_q p(q|\rho,\delta)^{N_q} \quad (6)
$$

The parameters in the model are not fully identified by the data. As a result, there are many maximum likelihood estimates (MLEs).

Where, $N_{1111} = 190$, $N_{1110} = 64$, $N_{1010} = 10$, $N_{1100} = 1304$, and $N_{1000} = 558$, the following table provides MLEs under several constraints that fully identify the model (constrained parameters bolded):

	Model 1	Model 2	Model 3	Model 4
ρ_1	**0**	0.15	**0**	0.16
ρ_2	0.28	**0**	**0**	0.69
ρ_3	0.72	0.85	**1**	0.15
δ	-	-	-	0.91
$\delta_{2,1}$	0.57	-	-	-
$\delta_{3,1}$	0.91	0.91	0.85	-
$\delta_{3,2}$	0.29	0.25	0.22	-
$log(\mathcal{L})$	-13.52	-13.52	-35.95	-13.52

Model 4 is constrained with respect to the decay rates, as it assumes that there is a constant rate of decay over time, δ. In this Model, a unique set of parameter values gives the maximum value of the likelihood (-13.52). The justification for this assumption is provided by measurement of the same protein divergence between WGD2 and WGD1 as between WGD1 and the present (cf. Materials and Methods). The parameter values $\rho_1 = 0.16$, $\rho_2 = 0.69$, $\rho_3 = 0.15$ are consistent with a wave of insertions that peaked in the period between WGD2 and WGD1. The value for δ is the same as that obtained for $\delta_{3,1}$ in the less constrained models (see below).

The three other models are constrained with respect to time of IES insertion, and there are a continuum of parameter values which share the maximum value of the likelihood (-13.52), some of which involve quite different values of several of the parameters. For example, the same value of the likelihood results from $\rho_1 = 0.13$, $\rho_2 = 0.71$, $\rho_3 = 0.15$, $\delta_{2,1} = 0.90$, $\delta_{3,1} = 0.91$, and $\delta_{3,2} = 0.87$. These are quite different parameter values than some of the constrained models, but they fit the data just as well. In particular, there are MLEs involving a wide range of values for all parameters except $\delta_{3,1}$, which is well-identified by the data to be in the vicinity of 0.91.

Fortunately, even without the assumption of a constant decay rate over time, we can rule out one hypothesis of interest, which is that that all IES quartets arose before WGD2. Model 3 above corresponds to that hypothesis, and it is strongly rejected by the data. A likelihood ratio test comparing that hypothesis to Model 1, Model 2, or any of the other models that result in $log(\mathcal{L}) = -13.52$ has $\chi^2_{df} = 43.48$, where df is equal to 1, 2, or 3, depending on which model is used as a comparison. These correspond to p-values of 2.1×10^{-11}, 1.8×10^{-10}, and 1.0×10^{-9}, respectively. Thus, we can quite confidently reject the hypothesis that all the IES creation events occurred before WGD2.
